# Varlociraptor: enhancing sensitivity and controlling false discovery rate in somatic indel discovery

**DOI:** 10.1186/s13059-020-01993-6

**Published:** 2020-04-28

**Authors:** Johannes Köster, Louis J. Dijkstra, Tobias Marschall, Alexander Schönhuth

**Affiliations:** 1 Algorithms for Reproducible Bioinformatics, Genome Informatics, Institute of Human Genetics, University Hospital Essen, University of Duisburg-Essen, Essen, Germany; 2 Dana-Farber Cancer Institute, Harvard Medical School, Boston, USA; 3 Centrum Wiskunde & Informatica, Amsterdam, The Netherlands; 4 Leibniz Institute for Prevention Research and Epidemiology - BIPS, Bremen, Germany; 5 Institute for Medical Biometry and Bioinformatics, Medical Faculty, Heinrich Heine University, Düsseldorf, Germany; 6 Genome Data Science, Faculty of Technology, Bielefeld University, Bielefeld, Germany

**Keywords:** Variant calling, Tumor/normal, Somatic, Bayesian latent variable model, FDR control

## Background

The foundation of cancer is the modifications of the genome; somatic mutations turn originally healthy cells into a heterogeneous mix of aberrantly evolving cell clones [1]. Global consortia have launched population-scale sequencing projects concerned with the discovery and annotation of somatic variants in cancer genomes [2, 3]. Potential benefits of the systematic analysis of somatic mutations include improved diagnosis, staging, and therapy protocol selection in the clinic.

The routine use of tools for the discovery of *somatic* variants naturally is at the core of such large-scale projects. However, only the discovery of somatic single nucleotide variants (SNVs) can be considered sufficiently addressed so far. The situation differs quite substantially for somatic insertions and deletions (indels; here, we refer to insertions and deletions of all possible sizes, ranging from 1 to thousands of base pairs, because we will focus on such variants in the following. Note that elsewhere, insertions and deletions larger than 50 bp are often subsumed as structural variants) and for other types of structural variants, such as translocations, duplications, and inversions.

For the discovery of *germline* indels and SVs, a plethora of approaches have been available in the meantime. These approaches vary in terms of benefits and drawbacks, but address the issue sufficiently well, with only a few open problems remaining, see [4] for a recent review.

Methods for the discovery of *somatic* variants can be classified into those targeting SNVs and small indels (e.g., Mutect2, Strelka, NeuSomatic, SmuRF [5–8]) and larger indels and SVs (e.g., Delly, Manta, LUMPY, GRIDSS, novoBreak [9–12]). To the best of our knowledge, the only method so far that is able to handle both small and large indels in a unified way is Lancet [13]. However, none of the methods is able to assess all involved uncertainties in a statistically sound way. This is reflected by the fact that suggested filtering operations rely on combinations of hard thresholds on various scores like, e.g., *p* values, read depth, strand bias test statistics, and allele counts.

As a result, given the amount of somatic variants discovered so far, there is room for improvement across the whole range of possible variant types [14], documented by the rather small amounts of variants discovered so far. In this, somatic insertions and deletions (indels) have proven to pose particular challenges when belonging to certain classes or length ranges [15, 16]. Indels of length approximately 30–250 bp, previously termed “the next-generation sequencing (NGS) twilight zone of indels” [17–19], have resisted their discovery in particular also in somatic variant calling: while the COSMIC database (https://cancer.sanger.ac.uk/cosmic; Release v88, data retrieved on 20 March 2019) counts 1,879,044 indels of length 1–30 bp, it only counts 17,793 indels of length 31–60 bp, 3758 indels of length 61–100 bp, and 2483 indels of length 101–250 bp. The drop by two orders of magnitude from 1–30 to 30–60 bp, not followed by any such drops in further length bins, has so far not been supported by any reasonable biological interpretation. Further supported by our benchmark experiments, the most likely explanation is that the majority of somatic indels in that size range have remained undiscovered so far, hence correspond to a blind spot in somatic mutation discovery.

Therefore, the application of more sensitive somatic indel calling strategies most likely will induce striking changes in the spectrum of somatic indels so far detected. As indicated in earlier work [16], this has the potential to deepen our understanding of the origin and effects of somatic indels, beyond just balancing count statistics.

Here and in the following, *sensitivity or recall* denotes the ratio of true indels discovered over the overall amount of true indels, whereas *precision* denotes the ratio of true indels discovered over the overall amount of indels discovered. *False discovery rate (FDR)* is the ratio of mistaken discoveries over the overall amount of indels discovered (so, precision = 1 − FDR).

In this paper, we suggest a sensitive strategy and prove that it substantially reduces the somatic indel discovery blind spot. To understand the issues that are characteristic of this blind spot, consider that somatic variant discovery (unlike germline variant discovery) is a two-step procedure: in the first step, one discovers putative variants in both the cancer and healthy (or control) genome of the individual analyzed; in the second step—which is unique to somatic variant discovery—one runs a differential analysis that classifies putative variants into somatic, germline, healthy somatic, or just noise. Here, somatic variants only appear in the cancer genome, germline variants occur already in the healthy genome, and healthy somatic variants appear in the healthy genome but at subclonal levels.

Already the first step (which also applies for generic germline variant discovery) is affected by major issues, where for example gap wander and annihilation (see [20]) are well-known and notorious examples when determining gapped alignments in general. Issues become further aggravated when dealing with indels of 30–250 bp due to particularities of NGS read alignment and indel discovery tools. This explains why one needs to make particular methodical efforts already when seeking for “twilight zone” germline variants [17–19, 21].

In somatic indel discovery, an additional layer of issues due to cancer heterogeneity has to be considered. The variant allele frequency (VAF), here the fraction of genome copies in the (tumor or control) sample affected by the variant, is either 0.0, 0.5, or 1.0 for germline variants, reflecting absence, heterozygosity, or homozygosity, respectively. In contrast, allele frequencies of somatic variants vary across the whole range from 0.0 to 1.0, depending on the clonal structure of the tumor sample and its impurity (the ratio of healthy genome copies in the tumor sample). Usually, there is no prior information about the clonal structure available at the time of variant calling. Low-frequency variants (i.e., having a VAF close to 0) yield particularly weak, statistically uncertain signals. Of course, there are limits to somatic variant discovery, relative to VAF and sequencing depth. The methodical challenge is to not miss any discoveries that a statistically sound approach is able to reveal. The purpose of this paper is to understand the limits of somatic variant discovery in theory and considerably push them in practice.

Certainly, there are still also improvements for the first step conceivable; however, the second (differential analysis) step has never been treated before with statistical rigor. So, the second step may have left room for improvements in particular. We recall that dealing with the various statistical uncertainties due to (as abovementioned) cancer heterogeneity, gap placement, strand bias, etc. is the major challenge in the second step. For successful operation, one needs to accurately quantify all relevant uncertainties in the first place. As a consequence, one has a sound statistical account on whether putative variants are somatic, germline, healthy somatic, or just errors. In consequence, statistically sound *false discovery rate (FDR)* control becomes possible: the user specifies the maximal ratio of false discoveries she/he is willing to deal with. Subsequently, a maximal set of discoveries is reported that ensures that the FDR specified by the user is not exceeded.

The desired accuracy in uncertainty quantification, however, comes at a cost: if data is uncertain, one deals with an exponential amount of possibly true data scenarios. This prevents naive approaches to work at the desired level of accuracy without serious runtime issues, which constitutes a common computational bottleneck in uncertainty quantification. In our setting, we will be dealing with at least 3^*n*^ possible scenarios for one putative indel where *n* corresponds to the sum of read coverages in the cancer and control genome at a particular locus. Nowadays, *n* around 60 is not uncommon. This number of arithmetic operations is prohibitive when processing up to hundreds of thousands putative indel loci.

In the model presented in this paper, we overcome this bottleneck by presenting a Bayesian latent variable model whose conditional dependencies point out how to compute all of the relevant probabilities in time *linear*, *and not exponential* in the coverage of a putative indel locus. Thanks to its computational efficiency, the model captures and accurately quantifies all uncertainties involved in somatic indel discovery in a comprehensive manner.

In summary, we are able to compute all probabilities required for enforcing reliable FDR control at both the necessary speed and accuracy. Reliable, sound, and accurate FDR control, in turn, gets us in the position to substantially increase the recall in somatic indel discovery, without having to deal with losses or even improving in terms of precision. As was to be expected, improvements in the twilight zone turn out to be the most dramatic: in comparison with the state-of-the-art tools, we double or even triple recall, while preserving (often better than just) operable precision.

## Results

We have designed *Varlociraptor*, as a method to implement the improvements in the differential analysis step outlined in the introduction. To the best of our knowledge, Varlociraptor is the first method that allows for accurate and statistically sound false discovery rate control in the discovery of somatic indels. As a consequence, the application of Varlociraptor leads to substantial increases in recall in somatic indel discovery. Varlociraptor doubles or even triples the number of true discoveries in comparison with the state-of-the-art tools, while often also further improving on precision, or, at any rate, not incurring any kind of loss in precision. Varlociraptor also accurately estimates the variant allele frequency (VAF) for all somatic indels.

In the following, we provide a high-level description of the Varlociraptor workflow. We provide a brief explanation of how one can quantify all relevant uncertainties in linear runtime, as the key methodical breakthrough and the fundamental building block that underlies all that follows. We briefly illustrate how the Bayesian latent variable model that enables rapid quantification of uncertainties further immediately gives rise to the computation of all probabilities that are crucial in somatic variant calling. We further briefly address how Varlociraptor estimates variant allele frequencies (VAFs). Finally, we explain how accurate FDR can be established. For details, we refer to the “Methods” section.

Subsequently, we analyze Varlociraptor’s performance in comparison with the current state-of-the-art tools on simulated and real data. As pointed out above, we show that Varlociraptor indeed achieves (sometimes drastic) increases in recall, often accompanied by further increases in precision. We notice that the probabilities used for classifying putative variant calls allow for a clear distinction between true and false positives, which is of considerable value in classification practice. We then demonstrate that Varlociraptor indeed reliably controls FDR, thereby also providing the theoretical explanation for why Varlociraptor achieves superior performance rates in terms of recall and precision. Varlociraptor further accurately estimates all VAFs. Turning our attention to real data, we conclude that Varlociraptor achieves superior concordance for variants of VAF at least 20%. For variants of VAF of less than 20%, Varlociraptor is the only tool that discovers considerable amounts of variants. The low coverage of reads supporting such calls delivers stringent statistical explanations for why concordance cannot be reached at rates that apply for calls above 20%. To corroborate that the majority of Varlociraptor’s calls are correct—just as we experienced on simulated data—we demonstrate that Varlociraptor’s count statistics agree with the theoretical expectation under neutral evolution.

### Workflow

We first discuss how Varlociraptor embeds in a workflow for somatic variant calling and highlight the central difference to classical approaches.

**Fig. 1:**
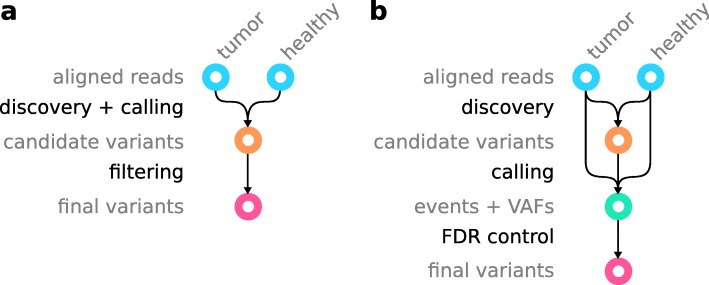
Difference between **a** the classic somatic variant calling workflow and **b** the Varlociraptor approach

The classical workflow for calling somatic variants (Fig. 1a) starts with aligned reads from tumor and corresponding healthy sample of the same patient in BAM (https://samtools.github.io/hts-specs/SAMv1.pdf) or CRAM (https://samtools.github.io/hts-specs/CRAMv3.pdf) format. First, variants are discovered, and a differential analysis is performed to call variants as somatic or germline. Candidate variants are reported in VCF or BCF format (https://samtools.github.io/hts-specs/VCFv4.3.pdf). In the following, we will refer to VCF as a placeholder for VCF/BCF, and BAM as a placeholder for BAM/CRAM. Second, the candidate variants are filtered, usually by applying thresholds for various scores (e.g., variant quality, strand bias, coverage, minimum mapping quality, minimum number of supporting reads in healthy sample), in order to obtain final variant calls. If not just relying on some suggested defaults, finding those thresholds is often a tedious, study-specific effort.

With Varlociraptor, we provide a new approach for calling and filtering, thereby separating variant discovery from calling (Fig. 1b). The input for Varlociraptor are candidate variants from an external discovery step. Here, any variant calling tool can be applied. At the time of writing, the Varlociraptor implementation supports SNVs, multiple nucleotide variants (MNVs), insertions, and deletions. However, the model presented here is agnostic of the variant type, and we are actively working on adding support for all other types of variants in Varlociraptor. Therefore, while we are writing about indels in the following, keep in mind that the presented model can straightforwardly be applied to other variant types. Similarly, Varlociraptor currently supports single-end or paired-end short reads, while the model itself is agnostic of the sequencing protocol and technology. The implementation will be extended in the future. There are plenty of approaches that thoroughly deal with the discovery step. In particular, as it is already common practice within the state-of-the-art somatic variant calling pipelines (e.g., the Sarek pipeline, [22]), it is possible to combine the candidate variants of different callers in order to obtain maximum sensitivity across all variant types and length ranges. However, instead of having to perform ad hoc filtration of finalized calls (e.g., Sarek performs majority voting), Varlociraptor provides a unified mechanism for assessing all candidate variants. During calling, Varlociraptor classifies variants into somatic tumor, somatic healthy, germline, or absent variants, while providing posterior probabilities for each event (see the “Classification” section) along with maximum a posteriori estimates of the variant allele frequency (VAF) (see the “Estimating allele frequencies for somatic tumor variants” section), reported in BCF format. Finally, using the posterior probabilities, Varlociraptor can filter variants by simply controlling for a desired false discovery rate (FDR, see the “False discovery rate control” section), instead of requiring the adjustment of various thresholds. This becomes possible because Varlociraptor, as the first approach, integrates all known sources of uncertainty into a single, unified model.

In this work, we will evaluate Varlociraptor’s performance in direct comparison with the (usually ad hoc) differential analysis routines provided by other tools. We will use the indels that are output by the respective tool to compare against in its first (discovery) step as input for Varlociraptor. This way we ensure that all tools receive input they are supposed to deal with and therefore ensure maximum fairness.

### Foundation of the approach

#### Efficient computation of the fundamental likelihood function

Let us fix a particular variant locus, as given by an entry in the VCF file that lists all candidate variants from Fig. 1b. By *θ*_*h*_ and *θ*_*c*_, we denote the true but unknown allele frequency of that (putative) variant among the healthy (*θ*_*h*_) and the cancerous (*θ*_*c*_) genome copies. While for *germline* variants, *θ*∈{0,1/2,1} reflecting absence (artifacts or noise) and hetero- and homozygosity of the variant, and *θ*∈[0,1] for somatic variants. We model that *somatic healthy* variants usually show at subclonal rates by allowing only *θ*∈(0,1/2), i.e., the exclusive interval between 0 and 1/2. A variant evaluates as *somatic tumor* if and only if *θ*_*c*_>0, while *θ*_*h*_=0. Since we are most interested in these variants, one of the central goals is to conclude that *θ*_*c*_>0,*θ*_*h*_=0 for a particular putative variant with sufficiently large probability.

By $\boldsymbol {Z}^{h}=\left (Z_{1}^{h},...,Z_{k}^{h}\right)$ and $\boldsymbol {Z}^{t}=\left (Z_{1}^{t},...,Z_{l}^{t}\right)$, we denote the read data being associated with the variant locus in the healthy (h) and the tumor (t) sample. Note that we distinguish between the tumor sample, which is a mixture of healthy and cancer cells, and the cancer cells themselves. When referring to the latter, we use the subscript *c*, and for the former, we use the subscript *t*. Each of the $Z_{i}^{h},Z_{j}^{t}, i=1,...,k, j=1,...,l$ represents one (paired-end) read that became aligned across or nearby the given variant locus. This further means that *k* and *l* correspond to the sample-specific read coverages at that locus. For selecting reads via alignments, we use BWA-Mem [23] in the following, although the choice of particular aligner is optional, as long as the aligner outputs a MAPQ value [24], which quantifies the certainty by which the sequenced fragment (represented by the read pair) stems from the locus under consideration.

Let further $\beta \in \{0,\frac 12,1\}$ denote the *strand bias* affecting the particular variant locus. Thereby, *β*=0 and *β*=1 denote that evidence about the putative variant occurs only in the reverse (*β*=0) or in the forward (*β*=1) strand. Both cases are indicators of sequencing or mapping artifacts. Therefore, no strand bias, i.e., *β*=1/2 will subsequently be used to select for non-artifact variants.

We present a *Bayesian latent variable model* that enables to *efficiently compute*:

1 $$  L(\theta_{h}, \theta_{c}, \beta \mid \boldsymbol{Z}^{h}, \boldsymbol{Z}^{t}),  $$

the likelihood of allele frequencies *θ*_*h*_,*θ*_*c*_ and strand bias *β* given read data ***Z***^*h*^,***Z***^*t*^ (see the “The model” section). Straightforward approaches to compute (1) via *fully Bayesian inverse uncertainty quantification* [25], which is the canonical and approved way to compute (1) fail due to requiring exponential runtime (also see section S1 (Additional file 1)). The conditional dependency structure of the statistical model we raise (see the “Methods” and the “The model” sections), points out a way to compute (1) in runtime linear in *k*+*l*, as summarized by the following theorem.

##### **Theorem 1**

*L*(*θ*_*h*_,*θ*_*c*_,*β*∣***Z***^*h*^,***Z***^*t*^) can be computed in *O*(*k*+*l*) arithmetic operations.

Note that this is the best one can hope for; the insight is crucial in somatic variant calling practice, beyond establishing also a theoretical novelty, because Theorem 1 establishes that *L*(*θ*_*h*_,*θ*_*c*_,*β*∣***Z***^*h*^,***Z***^*t*^) can be evaluated fast at any (*θ*_*h*_,*θ*_*c*_,*β*). This in turn renders integration over *L*(*θ*_*h*_,*θ*_*c*_,*β*∣***Z***^*h*^,***Z***^*t*^) computationally feasible, which facilitates solving the following three essential problems.

#### Classification

Statistically sound classification in somatic variant calling requires, when given ***Z***^*h*^,***Z***^*t*^, to compute the posterior probabilities for the following four cases, which refer to different combinations of *θ*_*c*_,*θ*_*h*_ (see also Fig. 8a in the “Methods” section).

- Somatic tumor (st); *θ*_*c*_>0,*θ*_*h*_=0: the variant is somatic in the tumor and does not appear in the healthy genome.
- Germline (ge); *θ*_*h*_∈{1/2,1}: a germline variant, where *θ*_*h*_=1/2 reflects a heterozygous and *θ*_*h*_=1 a homozygous variant.
- Somatic healthy (sh); *θ*_*h*_∈(0,1/2): a variant that is somatic but appears in the healthy genome, reflected by subclonal, non-germline variant allele frequencies.
- Absent (ab); *β*∈{0,1} or *θ*_*c*_=0,*θ*_*h*_=0: the variant reflects (strand bias) artifacts or noise.

Note in particular that cases (st), (ge), and (sh) imply that there is no strand bias, i.e., *β*=1/2. Given the respective read data ***Z***^*h*^,***Z***^*t*^ from the healthy and the tumor genome, the corresponding posterior probabilities compute as:

2 $$ {}\begin{aligned} P\left(\text{st} \mid \boldsymbol{Z}^{h}, \boldsymbol{Z}^{t}\right) & = \frac{1}{P(\boldsymbol{Z}^{h}, \boldsymbol{Z}^{t})} \int_{0}^{1} h(0, \theta_{c}, \nicefrac{1}{2}) L\left(0, \theta_{c}, \nicefrac{1}{2} \mid \boldsymbol{Z}^{h}, \boldsymbol{Z}^{t}\right) d\theta_{c},  \end{aligned}  $$

3 $$ {}\begin{aligned} P\!\left(\!\text{ge} \!\mid \boldsymbol{Z}^{h}, \boldsymbol{Z}^{t}\right) & \,=\, \frac{1}{P(\boldsymbol{Z}^{h}\!, \boldsymbol{Z}^{t})}\! \int_{0}^{1} \!\sum_{\theta_{h} \in \{\nicefrac{1}{2}, 1\}} \!h(\theta_{h}, \!\theta_{c},\! \nicefrac{1}{2}) L\left(\!\theta_{h}, \theta_{c},\! \nicefrac{1}{2} \!\mid\! \boldsymbol{Z}^{h}\!, \boldsymbol{Z}^{t}\!\right) \!d \theta_{c},  \end{aligned}  $$

4 $$ {}\begin{aligned} P\!\left(\!\text{sh}\! \mid \boldsymbol{Z}^{h}\!, \boldsymbol{Z}^{t}\!\right) & \,=\, \frac{1}{P(\boldsymbol{Z}^{h}\!, \boldsymbol{Z}^{t})} \!\int_{0}^{\nicefrac{1}{2}} \!\int_{0}^{1} h(\theta_{h}, \theta_{c},\! \nicefrac{1}{2}) L\!\left(\!\theta_{h}, \theta_{c},\! \nicefrac{1}{2} \!\mid\! \boldsymbol{Z}^{h}\!, \boldsymbol{Z}^{t}\!\right) \!d\theta_{c} d\theta_{h},  \end{aligned}  $$

5 $$ \begin{aligned} P\left(\text{ab}\mid \boldsymbol{Z}^{h}, \boldsymbol{Z}^{t}\right) & = 1 - \sum_{\{st,ge,sh\}} P\left(\cdot\mid\boldsymbol{Z}^{h},\boldsymbol{Z}^{t}\right). \end{aligned}  $$

*h*(*θ*_*h*_,*θ*_*c*_,*β*) is the prior distribution of the three readouts *θ*_*h*_,*θ*_*c*_, and *β*, which can be used to integrate prior knowledge about clonal structure or zygosity rates, if available. We consider the choice of *h* as an open question, that is most important for sparse data (i.e., very low coverage). It can be guided by the work of [26], for example. For the evaluation conducted here, we use a uniform *h*. *P*(***Z***^*h*^,***Z***^*t*^) is the marginal probability of the data, which acts as a normalization factor.

The integrals lack an analytic formula but, supported by the efficient computation of (1), as established by Theorem 1, can be approximated numerically using quadrature.

#### Estimating allele frequencies for somatic tumor variants

Upon having determined that a variant is a somatic tumor, implying *θ*_*c*_>0,*θ*_*h*_=0 and $\beta =\frac 12$, we would like to determine maximum a posteriori estimates for *θ*_*c*_, given the fragment data ***Z***^*h*^,***Z***^*t*^. When using a uniform prior this is the same as computing the maximum likelihood estimate:

6 $$  \widehat{\theta_{c}}\equiv \underset{\theta_{c}\in [0,1]}{\arg\,\max} \ L\left(0, \theta_{c}, \nicefrac{1}{2} \mid \boldsymbol{Z}^{h}, \boldsymbol{Z}^{t}\right).  $$

The likelihood function (1) is a higher-order polynomial in *θ*_*h*_ and *θ*_*c*_ for given *β*, as follows from the computations in the “Statements” section, which makes it infeasible to derive its maximum analytically. We can nevertheless prove the following theorem.

##### **Theorem 2**

Let the following conditions hold:

1. The likelihood of *θ*_*h*_,*β* given the data from the healthy sample must be non-zero, i.e.:
  $$\prod_{i=1}^{k} P(\boldsymbol{Z}^{h}_{i} \mid \theta_{h}, \beta) > 0. $$
2. The subset:
  7 $$ I := \{\theta_{c} \in [0,1]: P(Z_{j}^{t} \mid \theta_{h}, \theta_{c}, \beta) > 0\ \text{for}\ j =1,\dots,l\}  $$
  is connected and non-empty.
3. The purity is greater than 0, i.e., *α*>0 (otherwise the “tumor” sample would not contain any cancer cells).
4. There exists read data $Z_{j}^{t}$ for which the alignment probability $\pi _{j}^{t}$ is strictly larger than 0, and *p*_*j*_≠*a*_*j*_ (i.e., there must exist an observation that with non-zero probability stems from the locus of interest and provided information about the presence or absence of the indel of interest).

Then, for fixed *θ*_*h*_ and *β*, the logarithm of the likelihood function *θ*_*c*_→*L*(*θ*_*h*_,*θ*_*c*_,*β*∣***Z***^*h*^,***Z***^*t*^) is concave on the unit interval [0,1]. Hence, the likelihood function attains a unique global maximum $\widehat {\theta }_{c}$ on [0,1].

Conditions 1–4 are technical and always apply in practice. A proof for the theorem can be found in section S2 (Additional file 1). Since the logarithm of the likelihood function is strictly concave, its maximum can be easily determined numerically. This allows to report the corresponding global maximum $\widehat {\theta }_{c}$ for *θ*_*h*_=0 and $\beta =\frac 12$ as a reasonable estimate for the VAF of a somatic cancer variant.

#### False discovery rate control

Once the event probabilities defined in the “Classification” section are available, FDR can be controlled in a statistically sound way. In an ideal setting, the user specifies an FDR threshold *γ*, upon which a maximal amount of variants is output such that the ratio of false discoveries among the discoveries overall does not exceed *γ*. Only if the underlying model is statistically sound, maximal increases in terms of true discoveries among the output (sensitivity or recall) can be expected. Note that ad hoc style FDR control procedures such as “call merging” (raising only discoveries simultaneously supported by several variant callers), while indeed controlling FDR, usually incur serious losses in terms of discoveries and tend to lead to discovery blind spots, thereby (while not being the main issue) contributing to the lack of “somatic twilight zone indels” so far discovered. Moreover, it becomes next to impossible to fine-tune an analysis to a particular FDR acceptable in the specific context. First tries on FDR control procedures for variant calling have been reported before [21, 27]. However, in this work, we present the first fully Bayesian approach, and also the first for the calling of somatic variants.

For a given set of putative somatic tumor variants *C*, each of which is annotated with $p_{i}, i=1,\dots,|C|$, the posterior probability (2) that variant *i* is indeed somatic tumor, we can calculate the expected FDR [28] as:

$$\begin{array}{*{20}l} \text{FDR}_{C} = \frac{1}{|C|} \sum_{i=1}^{|C|} 1 - p_{i} \end{array} $$

In order to both control FDR at *γ* and raise maximal output, one searches for the largest set of variants *C*^∗^⊆*C* such that ${FDR}_{C^{*}} \leq \gamma \phantom {\dot {i}\!}$. One can efficiently implement this by sorting variants by 1−*p*_*i*_ in ascending order, and summing up 1−*p*_*i*_ in that order until this sum divided by the number of summands collected has reached the threshold *γ*.

### Data analysis reproducibility

The evaluation performed in this paper is available as a reproducible Snakemake [29] workflow archive (10.5281/zenodo.3361700). In addition, we provide a *Snakemake report* (Additional file 2) that allows to interactively explore all figures shown in this article in the context of the workflow, the parameters, and the code used to generate them.

### Data

A central challenge when evaluating somatic indel calling is to sufficiently cover all relevant length ranges. Furthermore, the occurrence of already discovered true twilight zone indels is biased towards easily accessible, hence lesser uncertain regions of the human genome. However, somatic variant databases contain only few twilight zone indels (see the “Background” section). Hence, we chose a dual approach based on designing a simulated dataset with the desired properties for assessing precision and recall, complemented by a concordance analysis on real data.

#### Simulated data

We used a real genome (Venter’s genome [30]), previously applied for NGS benchmarking purposes [18, 21] as the control genome.

**Fig. 2:**
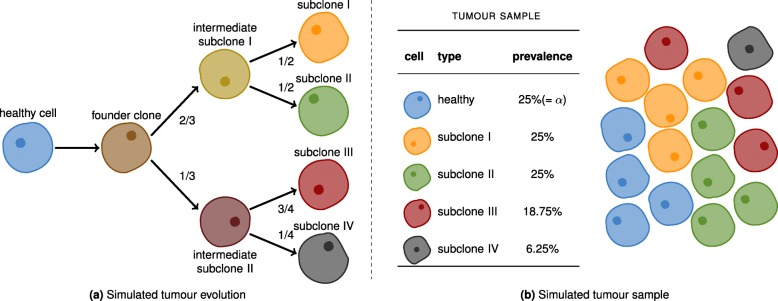
Simulated cancer clones. **a** The evolution of the cancer clones. **b** The simulated tumor sample. Each cell is assumed to be diploid. Cells of the same type share the same genetic code. The relative prevalences of the various cell types are shown in the table. The level of impurity (*α*) is 25%

Our goal was to simulate a cancer genome that qualifies for statistically sound benchmarking, while reflecting a scenario that is realistic in terms of tumor evolution. We therefore simulated 300,000 somatic point mutations, 150,000 insertions and 150,000 deletions, of which 279,509, 139,491, and 139,532 in the autosomes. Following the clonal structure described in Fig. 2, we sampled the mutations into four subclones. For example, 2/3 were assigned to intermediate subclone I. Of these, the first half was assigned to subclone I and the other half to subclone II. This simulates an evolutionary process, where mutations are inherited from parental intermediate subclones. The combination of the subclones at different abundances with an impurity caused by contamination with healthy cells constitutes an artificial tumor sample.

To account for realistic proportions in terms of length, indels follow the length distribution of Venter’s germline indels (which roughly follows a power law distribution). Allele frequencies range from 0.042 to 0.667 for the somatic indels. The majority of indels are of relatively low frequency, and they were randomly placed across the genome. Both low frequencies (making indels hard to distinguish from noise) and random placement (rendering the calling particularly difficult in repetetive regions), lead to a particularly challenging benchmarking dataset.

Reads were sampled according to the above model using the Assemblathon read simulator SimSeq [31]. For the tumor sample, we chose a coverage of 40 ×, and for the healthy sample, we simulated a 30 × coverage. Subsequently, reads were aligned using BWA-MEM [32]. The simulated reads are publicly available via Zenodo (10.5281/zenodo.1421298).

#### Synthetic data

In addition to the simulated reads above, we created a synthetic tumor/normal dataset based on the synthetic diploid benchmark from [33]. Using a combined assembly of both long (PacBio) and short (Illumina) reads, [33] provide high-quality variant calls for the two haploid cell lines CHM1 and CHM13. While not originally designed for this, the dataset can be slightly abused to approximate a tumor/normal situation as follows. First, we use publicly available short reads from CHM13 (SRR2088062, https://www.ncbi.nlm.nih.gov/sra/?term=SRR2088062) to define an artificial healthy or normal sample. Let *n* be the number of reads in SRR2088062, and *f*∈[0,1] be a mixture rate. Then, we create a synthetic tumor sample, by taking *f*·*n* read pairs from CHM1 (SRR2842672, https://www.ncbi.nlm.nih.gov/sra/?term=SRR2842672 and (1−*f*)·*n* read pairs from CHM13 (SRR2088062, our healthy sample). By this, variants exclusive to CHM1 can be seen as somatic variants in the synthetic tumor sample. Thereby, the mixture rate *f* defines the allele frequency of those variants in the synthetic tumor sample. In contrast, variants in both CHM13 and CHM1 can be seen as (synthetic) germline variants. We provide a reproducible Snakemake workflow conducting all necessary steps to generate the data (10.5281/zenodo.3630241).

The upside of this approach is that one obtains a tumor/normal dataset based on real sequencing reads, with a known ground truth. However, the following caveats that should be kept in mind. First, the variant calls in CHM1 and CHM13 are mostly small, not evenly covering all length ranges at sufficiently high numbers. Manual inspection reveals that in particular, many larger indels seem to come from repetitive regions, which are notoriously hard to map. Second, all synthetic somatic variants generated by this approach have the same allele frequency *f*, violating the assumption in the Varlociraptor model that allele frequencies in tumors are distributed in the unit interval [0,1]. This renders a fair assessment of FDR control and allele frequency estimation on this dataset impossible.

#### Real data

To demonstrate the applicability in practice, we also evaluated real cancer-control genome pairs. Since, as of today, there are no real datasets with known ground truth available (where the lack of real twilight zone indel discoveries is of course an important factor), we opt for a concordance analysis, as a procedure that has been used previously. Namely, we analyzed the concordance of reported variants with respect to four replicates of cancer-control genome pairs, all of which have been sampled from the same tumor cell line (melanoma cell line COLO829), albeit in different institutions [34] (https://ega-archive.org/datasets/EGAD00001002142). If discoveries referring to the four replicates agree to a sensible degree (considering that differences due to batch effects and independent progression are conceivable), one can conclude that performance is also of high quality on real datasets.

### Tools

For generating lists of candidate indels in form of VCF files (see Fig. 1), we chose Delly 0.7.7 [9], Lancet 1.0.0 [13], Strelka 2.8.4 [6], Manta 1.3.0 [35], BPI 1.5 (https://github.com/hartwigmedical/hmftools/tree/master/break-point-inspector), and NeuSomatic 0.2.1 [7, 36] as representative state-of-the-art callers covering all length ranges. All these callers provide their own “ad hoc” method of annotating somatic variants from tumor/normal sample pairs, which we applied with default parameters for comparison. We provided BWA-MEM alignments with marked duplicates (via Picard-Tools, https://broadinstitute.github.io/picard) as input for all tools. When subsequently running Varlociraptor (version 1.1.1), we used the output VCF files of the tools in combination with the BAM files that were the basis for generating the candidate variants.

### Experiments

We first consider the simulated dataset (see the “Data” section). Here, the true somatic variants are known from the simulation procedure. To classify predicted variants of the different callers into true and false positives, we matched them against the known truth using the vcf-match subcommand of RBT (https://github.com/rust-bio/rust-bio-tools) with parameters —max-dist 50 — max-len-diff 50. This means that we consider a predicted somatic variant to be a true positive if its position and length are within 50 bases of the true variant (which reflects approved evaluation practice, see [37] for further reasoning). Thereby, the position for a deletion is defined as its center point, i.e., ⌊(*e*−*s*)/2⌋ with *e* being the end position and *s* being the start position of the deletion. In the following, we show the results for deletions. If results for insertions essentially deviate from the deletion results, we mention it in the corresponding section of the text. All results for insertions can be found in the supplement.

#### Varlociraptor achieves substantial increases in recall without notable losses in precision

**Fig. 3:**
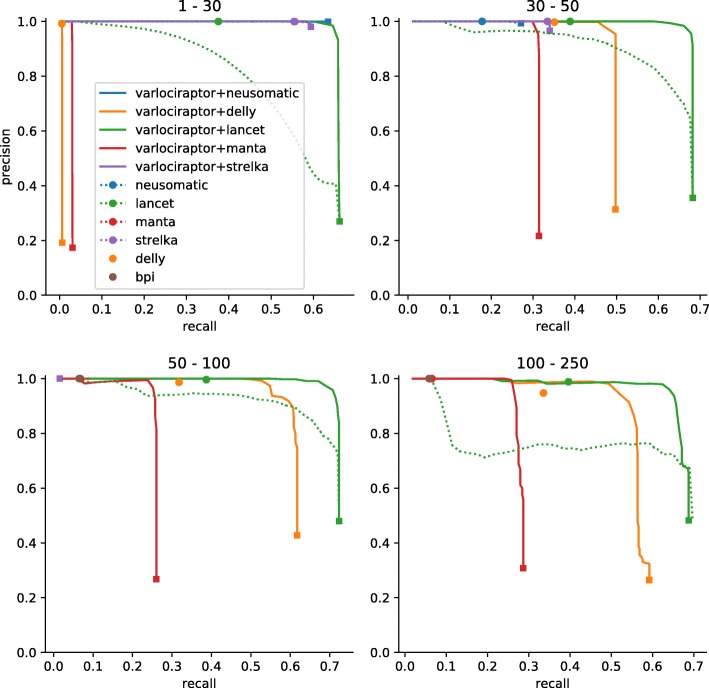
Recall and precision for calling somatic deletions on simulated data. Results are grouped by deletion length, denoted as interval at the top of the plot. For our approach (Varlociraptor+*), curves are plotted by scanning over the posterior probability for having a somatic tumor variant (for readability, each curve is terminated by a square mark). For other callers that provide a score to scan over (e.g., *p* value for Lancet), we plot a dotted line. Ad hoc results are shown as single dots. Results are shown if the prediction of the caller did provide at least 10 calls. The sharp curves for our approach reflect the favorable property of having a strong separation between the probabilities of true and false positives, see Fig. 4

In the following, *recall* is defined to be the ratio of true variants that became predicted, while *precision* is the ratio of correct predictions among the predictions overall. Figure 3 and S1 (Additional file 1) show recall and precision for all tools considered on the simulated data. In each plot, we juxtapose the tools’ recall and precision when run in stand-alone modus (dots or dotted lines) with recall and precision when postprocessing the respective output sheets of the tools with Varlociraptor (lines). The lines referring to Varlociraptor result from varying the posterior probability threshold: the greater the threshold, the smaller the recall. While the reduction in recall is merely a consequence of reducing the output, a simultaneous increase in precision only shows if posterior probabilities make sense, as (first) essential evidence of the quality of the approach. Note that the only caller that offers to vary an output-specific threshold is Lancet (dotted green line in Figure 3 and S1 (Additional file 1)), by scoring variants with *p* values. However, Lancet’s *p* values are only one component of the ad hoc filtering procedure performed by the tool, which relies on multiple scores. This explains while filtering based on the *p* values alone (dotted green line) yields suboptimal performance compared to the ad hoc calls provided by Lancet (green dot).

The first, fundamental observation is that Precision indeed increases on increasing the posterior probability cutoff, across all size ranges and inputs. This points out that Varlociraptor’s posterior probabilities for a variant to be somatic indeed make sense.

When further comparing Varlociraptor’s (continuous) lines with the dots or dotted lines referring to the stand-alone modus of the other tools, it becomes immediately evident that Varlociraptor improves on the tools’ results: Varlociraptor’s lines are upper right of the tools’ dots or dotted lines. This means that Varlociraptor achieves better combinations of recall and precision. The most striking observation however is that Varlociraptor achieves substantial increases in recall in comparison with the stand-alone tools: in particular, in the twilight zone (30–250 bp), Varlociraptor is able to double (Lancet, Delly) or even more than double (Manta) the recall, while never incurring notable losses in precision (sometimes even improving the latter). The synthetic data (Figures S8, S9, S10, S11 (Additional file 1)) in general confirms all findings from the simulated data, however, in a more noisy way, due to the caveats of the dataset mentioned in the “Data” section.

#### Posterior probabilities allow for a clear distinction between true and false positives

**Fig. 4:**
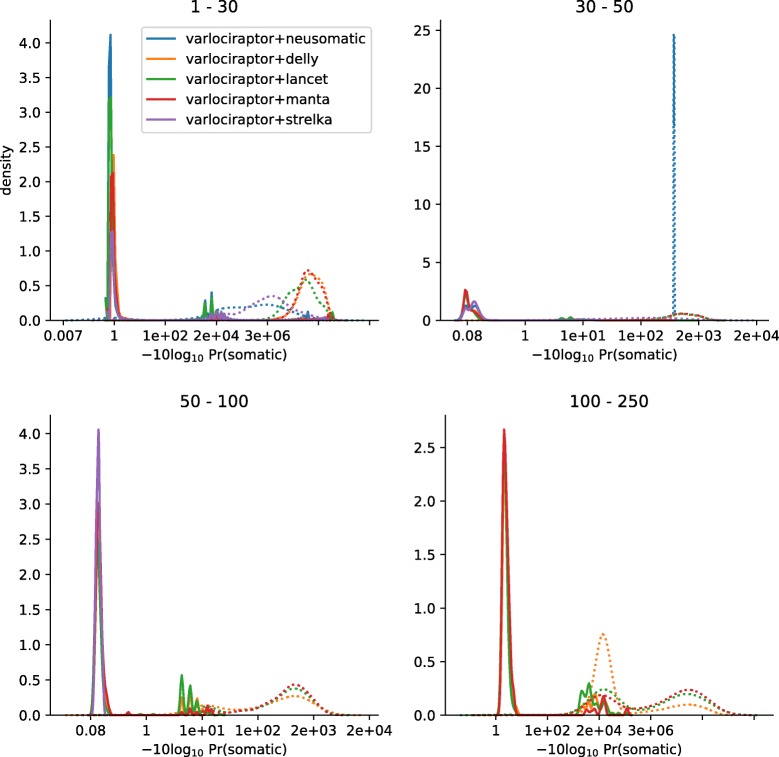
Posterior probability distributions for somatic deletions. Results are grouped by deletion length, denoted as interval at the top of the plot. The *x*-axis indicates the (PHRED-scaled) probability, and the *y*-axis indicates relative amounts of calls with this probability. The distributions of posteriors for true-positive calls are shown as solid lines; the distributions of posteriors for false-positive calls are shown as dotted lines

The precision-recall curves of Varlociraptor show a sharp turning point at their upper right. This indicates that Varlociraptor’s posterior probabilities allow for a clear and fine-grained distinction between true and false positives. Note that the vertical parts of these lines therefore also indicate the maximum recall one can achieve, the limit of which corresponds to the list of variant calls provided by the caller. In other words, Varlociraptor is able to identify all, or nearly all of the true variants that are provided as candidates. To further solidify this observation, we investigated the posterior probability distributions of Varlociraptor. Figure 4 and S4 (Additional file 1) show that Varlociraptor’s probabilities are indeed clearly separating true from false positives.

#### Varlociraptor reliably controls false discovery rate

Varlociraptor summarizes the uncertainty about a putative variant in terms of a single and reliable quantity: an estimate of the posterior probability for the putative variant to be somatic in the tumor. It remains to determine which of the variants to output. This is a ubiquitous issue in sequencing-based variant calling: optimally, one outputs maximum amounts of variants while ensuring that the mistaken predictions among the output variants do not exceed a preferably user-defined ratio. In other words, we would like to control the *false discovery rate (FDR)*.

Note that establishing statistically sound FDR control in variant discovery, to the best of our knowledge, amounts to a novelty: all of the state-of-the-art methods that we benchmarked against either cannot control FDR, or establish it through ad hoc methods, such that theoretical guarantees cannot be provided.

**Fig. 5:**
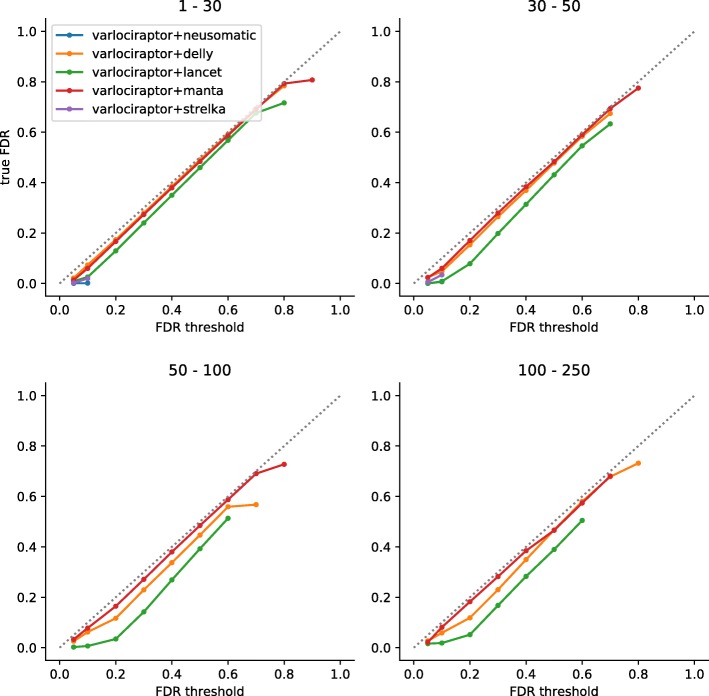
FDR control for somatic deletions. Results are grouped by deletion length, denoted as interval at the top of the plot. The axes denote the desired FDR, provided by the user as input (*x*-axis) and the true achieved FDR (*y*-axis). A perfect FDR control would keep the curve exactly on the dashed diagonal. Below the diagonal, the control is conservative. Above the diagonal, the FDR would be underestimated. Importantly, points below the diagonal mean that the true FDR is smaller than the threshold provided, which means that FDR control is still established; in this sense, points below the diagonal are preferable over points above the diagonal

For Varlociraptor, it is rather straightforward to establish FDR control: well-known theory [28] points out how to achieve maximally large output at a desired FDR, when working with Bayesian type posterior probabilities. See Fig. 5 and S2 (Additional file 1) for the evaluation of how Varlociraptor’s FDR control performs. In general, the closer to the diagonal, the better FDR control can be established by the user.

For deletions (Fig. 5), Varlociraptor controls FDR, in the sense that across all deletion length ranges the curve is on or below the diagonal, and that deviations from the diagonal are small. The latter means that Varlociraptor tends to be slightly conservative in some combinations of length range and FDR threshold provided. One reason contributing to this is that, induced by too coarse MAPQ values or base quality scores, the resolution of posterior probabilities may be limited.

As for insertions (Fig. S2 (Additional file 1)), which, as we recall, are generally more challenging, Varlociraptor equally achieves high-quality FDR control, across all length ranges and FDR thresholds. There is one caveat: for insertions of length 30–100 provided by Lancet [13], Varlociraptor’s FDR is slightly greater than the threshold specified by the user (and, although deviations are tiny, in this sense does not control FDR). An explanation for this is the modus operandi of Lancet: Lancet bases insertion calls on microassemblies that are computed from all reads mapping to the variant locus. This approach is reasonable for large insertions, which do not fit into single reads, because their length either exceeds the read length or are too long to show in single reads at full length. However, microassembly may also lead to false positives in repetitive areas where both alignments and assemblies can lead to ambiguities; note that Lancet only reaches a precision of 90% for larger insertions. Varlociraptor, at this point in time, cannot quantify all uncertainties emerging from microassemblies—we consider it a highly interesting future work to also quantify uncertainties that are associated with microassemblies (see the “Discussion” section).

#### Varlociraptor accurately estimates variant allele frequency

**Fig. 6:**
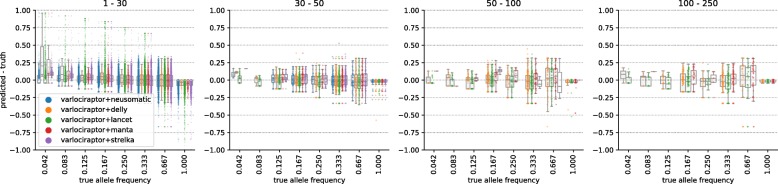
Allele frequency estimation for somatic deletions. Results are grouped by deletion length, denoted as interval at the top of the plot. The horizontal axis shows the true allele frequency; the vertical axis shows the error between predicted allele frequency and truth. The observable true allele frequencies are a result of the subclone structure used in the simulation process outlined in Fig. 2. Note that there are only few data points for allele frequencies below 0.125 with variant sizes above 30 bp. In consequence, those box plots have to be considered with care

Figure 6 and S3 (Additional file 1) show the difference between the true VAFs and the ones predicted by Varlociraptor, henceforth referred to as prediction error. The first observation is that the prediction error is approximately centered around zero in all cases, which is the desired scenario. We further investigated the effect of sequencing depth on the accuracy of the VAF estimates. Figures S6 and S7 (Additional file 1) show how the prediction error varies relative to sequencing depth (number of non-ambiguously mapped fragments overlapping the variant locus), denoted by *n* and true VAF, denoted by *θ*^∗^. For each combination of *n* and *θ*^∗^, we determine an expected baseline error, modeling that one samples *n* fragments each of which stems from a variant-affected genome copy with probability *θ*^∗^. In other words, the expected baseline error is governed by a binomial distribution *B*(*n*,*θ*^∗^). Accordingly, we determine:

$$\frac{1}{n} \sqrt{n\theta^{*}(1-\theta^{*})}, $$

that is the standard deviation of *B*(*n*,*θ*^∗^), divided (normalized) by the depth, as the expected baseline error. This establishes the theoretical optimum of a VAF estimation procedure. In other words, no sound estimator can achieve smaller error in prediction. We see that, on average, our prediction is close to this theoretical optimum. Further, the accuracy of VAF estimates increases on increasing sequencing depth, which is the desired, logical behavior. In summary, these results point out that the estimates are sound, and even close to what one can optimally achieve in theory. A possible explanation for the remaining small deviations from the theoretical optimum are reads that stem from different loci and because of ambiguity in placement get aligned to the variant locus. If the read mapper is unable to reflect such ambiguity in the MAPQ score, for example, because the true locus of origin is due to the variation not properly represented in the reference sequence, our model is as well not able to properly reflect this in the allele frequency estimation. We expect such problems to mitigate with longer reads and more accurate (even non-linear) reference genomes in the future.

#### Varlociraptor achieves superior concordance above VAF of 20%

We applied all callers (see the “Tools” section) on the four replicates of cancer-control genome pairs described above (the “Data” section), using their default parameters. In all analyses, because of the lack of prior knowledge available, Varlociraptor assumed a purity level *α* of 100%. We then performed a concordance analysis in the following way.

For each caller, we collected calls for each of the four replicates, both when run in stand-alone fashion and when postprocessing calls with Varlociraptor. For each of the calling strategies, we then computed matchings across the four replicates as described at the beginning of the “Experiments” section. For each calling strategy, we then constructed a graph where each node represents one variant call in one replicate, and edges indicate that two calls (from different replicates) are matched. We then consider the connected components of this graph: any non-trivial connected component (that is any connected component consisting of more than one node) counts as *concordant call*. We then determine *concordance* as the ratio of concordant calls over all connected components.

**Fig. 7:**
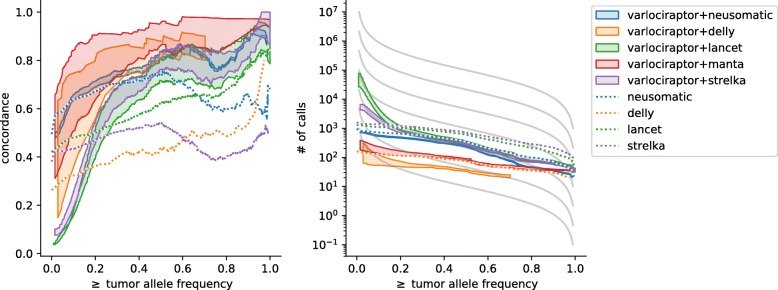
Concordance of somatic deletions on real data. For Varlociraptor, the interval between all calls with a posterior probability of at least 0.9 and at least 0.99 is shown as shaded area. Left: concordance vs. minimum allele frequency. Right: number of calls vs. minimum allele frequency. The different gray lines depict the theoretical expectation at different effective mutation rates according to Williams et al. [26] (see text)

In Fig. 7 and S5 (Additional file 1), we display for each possible VAF, the concordance of all calls with an at least as high VAF. In other words, for each VAF threshold *t*∈[0,1], we display the concordance all calls with a VAF ≥*t*. To allow for a fair comparison, we use Varlociraptor’s VAF estimates for all calling strategies. It becomes immediately evident that Varlociraptor achieves superior concordance for VAFs of 20% and higher.

**Fig. 8:**
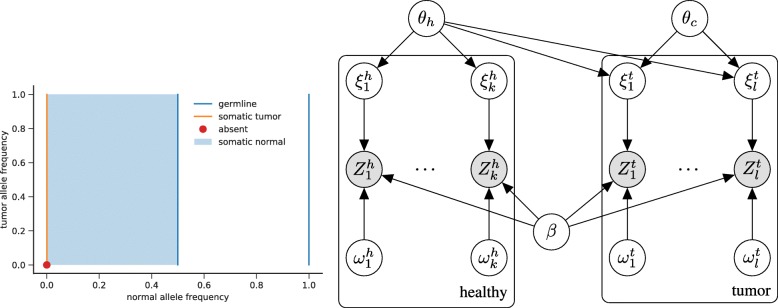
Left: visualization of the parameter space *Θ* of the VAFs. *Orange*: somatic variants agree with (*θ*_*h*_,*θ*_*c*_)∈{0}×(0,1], which means that no healthy cells have the variant (*θ*_*h*_=0), while some cancer clones do have the variant (*θ*_*c*_>0). Germline variants (*blue*) are described by $\theta _{h}\in \{\frac 12,1\}$ and absent variants (*red dot*) by *θ*_*h*_=0,*θ*_*c*_=0. Subclonal somatic variants in the healthy (normal) tissue are described by *θ*_*h*_∈(0,0.5). Right: diagram of the model presented in the “The model” section (white circles, latent variables; gray circles, observable variables). Each column corresponds to one alignment ($Z_{i}^{h}$ or $Z_{j}^{t}$) with its hyperparameters $\xi _{i}^{h},\omega _{i}^{h}$ or $\xi _{j}^{t},\omega _{j}^{t}$. Due to (potential) sample impurity (denoted by *α* in the text), *θ*_*h*_ has an influence on the alignments $Z_{j}^{t}$ from the tumor sample

#### Varlociraptor’s variant counts of VAF below 20% agree with the theoretical expectation under neutral evolution

When inspecting all calls with a minimum VAF below 20% (i.e., *t*<0.2, see above), Varlociraptor’s concordance drops below the rates achieved by callers run in a stand-alone fashion. This does not mean, however, that Varlociraptor’s performance is worse. Note first that Strelka and Lancet, which appear to have superior concordance, raise only very few (and, as we will point out below, according to evolutionary models too little) discoveries. This can be seen in the right panels of Fig. 7 and S5 (Additional file 1). In contrast, Varlociraptor raises substantially more discoveries at these frequencies. Second, for low-frequency variants, data may not reach the necessary degree of certainty (which leads to a call by Varlociraptor) in sufficiently many of the four samples, and, since the four replicates were raised in different laboratories, low-frequency variants unique to samples can be expected [34].

To explore this further and assess whether the larger numbers of low-frequency calls provided by Varlociraptor are potentially correct, we compared the somatic variant count distributions with the theoretical expectation under neutral evolution (see gray lines in the right panels of Fig. 7 and S5 (Additional file 1)). For this, we employ the tumor evolution model by Williams et al. [26] and calculate the expected number of somatic variants of at least allele frequency *f* as:

$$M(f) = \frac{\mu}{\lambda} \left(\frac{1}{f} - \frac{1}{f_{\text{max}}}\right).$$

Here, *μ* is the somatic mutation rate, *λ* is the growth rate, and *f*_max_ is the maximum clonal allele frequency. Because there are no reliable estimates for *f*_max_ available, we set *f*_max_=1.0. We then plot the expected counts at various values of $\frac {\mu }{\lambda }$ (the effective mutation rate). It can be seen that the counts of low-frequency variants provided by Varlociraptor (shaded violet and green areas) are closer to the theoretical expectation than those of Lancet and Strelka (dotted violet and green lines). This is an indication that, by evolutionary principles, it is likely that many low-frequency calls that were not recognized as concordant are still correct.

## Discussion

We have chosen to rely on external tools for the discovery of variants and focus on providing a sound and rigorous statistical treatment of the differential analysis (or the classification) of variants into the events relevant for somatic variant calling. A benefit of this strategy will be that Varlociraptor can be conveniently integrated in large-scale projects, as a postprocessing step in production-quality somatic variant calling pipelines. This particularly applies when projects are run by large consortia that manage various, often heterogeneous combinations of variant callers: as per its design, Varlociraptor offers the first approach that is able to analyze sets of variants raised by different callers, all of which come with their own strengths, weaknesses, and blind spots, in a *statistically unifying* way. Because Varlociraptor preserves and combines the individual strengths of the callers while eliminating any of their particular weaknesses, it is able to provide substantially improved variant calls. In summary, the application of Varlociraptor has the potential to lead to substantial increases in true somatic indel discoveries—possibly even overwhelming in certain size ranges—in large-scale matched tumor-normal genome sequencing projects.

The technical challenge has been to overcome a well-known and notorious computational bottleneck that relates to the quantification of the uncertainties affecting the differential analysis. Thereby, not only ambiguities in terms of gap placement and aligning reads in general, which had been dealt with in the literature abundantly before, but also effects such as cancer heterogeneity (implying uncertain variant allele frequencies), purity of tumor samples, bias in terms of sampling indel-affected fragments, and strand bias are major factors. We have presented, to the best of our knowledge, the first model that allows to capture all relevant effects and quantify all inherent uncertainties computationally efficiently. In particular, we want to stress that the presented model is the first approach for somatic variant calling that takes strand bias—as a major source for systematic artifacts—into a statistically comprehensive account, which is not possible if strand bias is quantified through independently raised auxiliary scores. Note that for germline calling, integration of strand bias into the statistical model is implementated in Freebayes [38], albeit not yet mentioned in the corresponding preprint and not considering mapping quality as done in the integration presented here.

An important aspect is the fact that the presented model can be considered as a “white box,” in the sense that all parameters have a direct biological interpretation. Hence, it supports the investigation of variant calls at different levels of detail, depending on the research question. First, one applies global filtering via FDR control. The remaining calls can optionally be investigated more closely, by looking at the estimated allele frequencies and their posterior distribution, the estimated sampling bias (see the “Sampling probability” section), the likelihoods and strand support of each fragment with associated mapping qualities, and, finally, the read alignments themselves (if necessary). This becomes particularly important in the era of personalized medicine, where variant calls for individual patients might lead to therapy decisions, requiring utmost certainty and transparency in the decision process.

Because the statistical model comprehensively addresses all relevant effects and related uncertainties, filtering the posterior probabilities derived from the model gives immediate rise to accurate and statistically sound (fully Bayesian) FDR control, for the first time in the variant calling field.

The advantage of such a straightforward and statistically interpretable filtering procedure becomes apparent when comparing it with the methodology of prior state-of-the-art approaches: so far, they have been relying on a variety of (often independently raised) scores, where combinations of (often manually) fine-tuned default thresholds are supposed to ensure that predictions contain reasonably little amounts of false discoveries. Modifying these combinations of scores in order to change the default FDR, provided by the developers through the default settings, is tedious, difficult, and error-prone, if not entirely impossible.

The analysis of somatic variants often serves the purpose to yield further insight into the clonal structure of a tumor which includes to assess the allele frequencies of somatic variants appropriately. Varlociraptor, again as per its design, does not only assess the probability of individual putative somatic variants to be true discoveries, but also equips them with an estimate for their allele frequency. We have demonstrated that the corresponding VAF estimates are of almost optimal accuracy.

Still, as always, there is room for improvements. First, Varlociraptor so far only deals with insertions, deletions, and single nucleotide variants (which are not analyzed in this work). Note however the framework presented is generic in terms of its dependency structures. Therefore, efficient computation of the central likelihood function is also warranted for other variant classes; the only thing required is to adapt the computation of the typing uncertainty (see “Computing *a*_*i*_,*p*_*i*_, and *o*_*i*_” section).

Second, we have been focusing on second-generation paired-end reads in this treatment, motivated by the fact that the vast majority of reads sequenced to date belong to this class. However, our model is entirely agnostic to any particular choice of sequencing platform and can be used without any further adaptations: the only basic requirement is that the sequencing/mapping protocol in use yields (sufficiently reliable) MAPQ values. Any particular sequencing/mapping-specific issues will be taken care of by the re-alignment step that Varlociraptor routinely makes use of for accurately quantifying alignment-related uncertainties. When re-parameterizing the pair HMM that underlies the re-alignment step (which reflects a straightforward adaptation) to emerging long-read technologies like Nanopore (https://nanoporetech.com) or SMRT sequencing (https://www.pacb.com/smrt-science/smrt-sequencing), Varlociraptor will be particularly apt for dealing with insertions and deletions within repeat regions.

Third, all quantities relating to uncertainties or spelled out probabilities that Varlociraptor (comprehensively) provides can be used further in a whole range of downstream analyses. Intriguing examples of such potential applications are the probabilistic assessment of larger somatic gains and losses, or the (partial) phasing of tumor subclones, which we will explore in future work.

Fourth, note that the ever more common use of unique molecular identifiers (UMIs) also offers statistically sound ways to address PCR stutter errors that arise when amplifying DNA molecules harboring short tandem repeat (STR) extensions prior to sequencing, which leads to ambiguities when removing duplicates [39]. Note further that without using UMIs, such ambiguities cannot be quantified in a statistically sound way. If UMIs are available, “smart deduplication” is possible in theory and already being implemented (https://github.com/rust-bio/rust-bio-tools).

Fifth, when aiming for the aggregation over calls from multiple callers that target the same type and size of variants, an important aspect is the merging of variants being called from more than one caller into a single representation. This is particularly challenging for indels and structural variants, which can, driven by alignment ambiguity, be represented in various different ways. With future releases of Varlociraptor, we aim to provide a strategy to merge such variant calls under consideration of alignment and mapping uncertainty. This will also enable a fair comparison between Varlociraptor and ensemble approaches like SmuRF [8].

Finally, it is important to note that the computational insights and the model presented in this work is, although motivated by, not at all limited to somatic variant calling. In fact, it turns out that it can be generalized towards arbitrary variant calling scenarios, where distinguishing between variants affecting the primary tumor and variants showing in metastases or relapse tumors, or distinguishing variants recurringly showing in different individual tumors from variants that do not, are immediate, relevant examples. The latest release of Varlociraptor already provides a *variant calling grammar*, as an interface for defining such scenarios (https://varlociraptor.github.io/docs/calling#generic-variant-calling) and thereby a foundation for a unifying theory of variant calling, enabling us to explore entirely new fields of applications.

## Conclusions

We have presented a statistical framework for the calling of somatic insertions and deletions from matched tumor-normal genome samples whose application has yielded substantial increases in terms of true discoveries, while safely limiting the amount of false discoveries. The framework is implemented in an easy-to-use open-source software, called Varlociraptor (https://varlociraptor.github.io). In comparison with the state of the art, we have demonstrated to double or even triple the amount of true discoveries while not increasing the false discovery rate (FDR), or suppressing it even further. Because the statistical model comprehensively addresses all relevant effects and related uncertainties, filtering the posterior probabilities derived from the model gives immediate rise to accurate and statistically sound FDR control, for the first time in the variant calling field. The model is extensible to other scenarios than somatic indel calling (any variant type, arbitrary sample relations and contaminations, other data types like RNA-seq and single-cell sequencing), ultimately yielding a unified theory of variant calling, which is already partially available in Varlociraptor and will be completed in the future.

## Methods

### Notation

We denote *observable variables* by Latin capital letters (e.g., *Z*). *Realizations* of these variables are denoted by small Latin letters (e.g., *z*). *Hidden/latent variables* are denoted by small Greek symbols. Vectors are denoted by boldface letters (e.g., ***Z***=(*Z*_1_,...,*Z*_*k*_) or ***z***=(*z*_1_,...,*z*_*k*_)). We use super-/subscripts *h* and *t* for the healthy and the tumor sample, respectively, and *c* to only refer to cancer cells within the tumor sample.

Let us fix a particular variant locus; we then denote the relevant read data in the healthy and the tumor sample by $\boldsymbol {Z}^{h}=\left (Z_{1}^{h},...,Z_{k}^{h}\right)$ and $\boldsymbol {Z}^{t}=\left (Z_{1}^{t},...,Z_{l}^{t}\right)$, respectively, where each of the $Z_{i}^{h},Z_{j}^{t}, i=1,...,k, j=1,...,l$ represents one (paired-end) read that (or parts of which) became aligned across or nearby the fixed variant locus. For selecting reads via alignments, we use BWA-Mem [23] in the following, although the choice of particular aligner is free, as long as the aligner outputs a MAPQ value, which quantifies the certainty by which the reads stem from the locus under consideration.

By *variant allele frequency (VAF)*, we refer to the fraction of genome copies in the sample affected by the variant. We denote this (unknown) frequency in the healthy and the tumor sample by *θ*_*h*_ and *θ*_*t*_, respectively. Since healthy cells are diploid, *θ*_*h*_ is either 0, 1/2, or 1 corresponding to absence, heterozygosity, and homozygosity, respectively, when dealing with a *germline variant*. It is common that healthy cells, beyond germline variation, also exhibit somatic mutations. Somatic variants that affect healthy cells usually occur at subclonal rates, i.e., are generally not characteristic in terms of giving rise to subpopulations among the healthy cells. It is therefore reasonable to assume that somatic variants affect only less than half of the cells, reflected by allowing *θ*_*h*_∈(0,0.5).

Prior to variant analysis, knowledge about *θ*_*t*_ is usually not available, because only variant analysis itself can yield insight into the clonal structure of a tumor. It is therefore reasonable to assume that *θ*_*t*_∈[0,1], that is, we allow any possible VAF to apply for a somatic variant in a tumor cell.

A tumor genome sample can still contain non-negligible amounts of healthy cells, which affects our considerations. Therefore, let *α*∈[0,1] be the *purity* of the tumor genome sample, that is, the relative amount of fragments in the sample that stem from a cancerous genome copy. Thus, 1−*α* is the relative amount of fragments that stem from a healthy genome copy from within the tumor genome sample. In addition to *θ*_*t*_ (the tumor allele frequency) and *θ*_*h*_ (the healthy allele frequency), we introduce *θ*_*c*_∈[0,1], which describes the allele frequency in the cancer cells only. We obtain the relationship:

8 $$  \theta_{t} = \alpha \cdot \theta_{c} + (1 - \alpha) \cdot \theta_{h},  $$

i.e., *θ*_*t*_ is a mixture of the frequencies among the cancer and the healthy cells that are in the tumor sample.

Finally, *strand bias* tends to introduce non-negligible issues in variant analysis. Our analyses are affected by strand bias as well. Strand bias refers to the fact that during the sequencing process, certain sequence motifs can cause the sequencing process to slow down, or even temporarily stall. If such delays occur, systematic sequencing artifacts affect the reads. Because the motif disappears when considering the complementary strand (unless the motif is a reverse complementary palindrome), artifact-inducing motifs usually affect reads from only one of the strands. This implies that the occurrence of artifacts depends on the origin of the read: artifacts can only affect reads from one of the strands, either the forward or the reverse strand, while reads sampled from the other strand are not affected by the sequencing artifact, see [40] for more details.

In summary, there are three possible cases we need to deal with: *first*, a putative variant affects only reads sampled from the reverse strand; *second*, the putative variant affects reads from forward and reverse strands at equal rates; or *third*, the putative variant only affects reads sampled from the forward strand. The exclusive support of a variant by reads from only one of the two strands is indicative of an artifact [41]. The goal is to remove such artifacts from the output.

We approach this by introducing a variable *β* taking values in $\left \{0,\frac {1}{2},1\right \}$ reflecting the three cases from above (so *β*=1 reflects that the variant only shows on reads that stem from the forward strand, and so on). In other words, *β* reflects the probability that a read that is associated with the variant stems from the forward strand.

### The model

We present a graphical model that captures all dependency relationships among the variables relating to the computation of *L*(*θ*_*h*_,*θ*_*c*_,*β*∣***Z***^*h*^,***Z***^*t*^) while taking all major uncertainties into account (see Fig. 8b).

Beyond observable variables reflecting observable read data $Z_{i}^{h},i\,=\,1,...,k, Z_{j}^{t}, j\,=\,1,...,l$ and latent variables *θ*_*h*_,*θ*_*c*_,*β* for allele frequencies and strand bias, we first introduce two additional latent variables. We denote with $\xi _{i}^{h},\xi _{j}^{t} \in \{0,1\}$ whether fragments $Z_{i}^{h},Z_{j}^{t}$ are associated with the variant $\left (\xi _{i}^{h}, \xi _{j}^{t} = 1\right)$ or not $\left (\xi _{i}^{h},\xi _{j}^{t}=0\right)$. Further, $\omega _{i}^{h},\omega _{j}^{t}\in \{0,1\}$ indicate whether reads indeed stem from the locus $\left (\omega _{i}^{h},\omega _{j}^{t}=1\right)$ or not ($\omega _{i}^{h},\omega _{j}^{t}=0$).

Variables $\omega _{i}^{h},\omega _{j}^{t},\xi _{i}^{h},\xi _{j}^{t}$ refer to the abovementioned three cases (a), (b), and (c). For example, $\xi _{i}^{h}=1,\omega _{i}^{h}=1$ represents the case that read $Z_{i}^{h}$ stems from the locus of interest and is indeed associated with the variant (case (c) above). Note that the case *ω*=0, which indicates that the read does not stem from the locus (a), renders specification of other variables obsolete, because this particular read cannot provide information about the variant. Since knowledge about realizations of the hyperparameters cannot be observed at the time of the analysis, they are latent variables.

In the following, we introduce the full model in three steps, thereby deriving the conditional dependencies between the different variables. *First*, we consider the basic model. *Second*, we introduce *impurity*, modeling the fact that the cancer genome sample also contains healthy genome copies. This implies to distinguish between the treatment of reads from healthy and tumor sample: while reads from the healthy sample still follow the basic model, processing reads from the tumor sample requires a modification to take impurity into account. *Third*, we introduce *strand bias*, which would affect both reads from the healthy and the tumor sample and thus requires to apply modifications for both parts.

**Part 1—Foundations: alignment and typing uncertainty.** We first model the two major sources of uncertainty, (a) *alignment uncertainty* and (b) *typing uncertainty*. Let $Z_{i}, i=1,\dots,k$ be the observable alignment data. *Alignment uncertainty* is handled via:

9 $$  \omega_{i} \sim \text{Bernoulli}\left(\pi_{i} \right)  $$

where *π*_*i*_ reflects the probability that the *i*th read has been aligned to the correct position in the genome. Estimates for *π*_*i*_ are provided by the aligner of choice, via the reported MAPQ values [42]. Note that for a multi-mapped read, the different *π*_*i*_ that apply for the different possible alignments of that read are supposed to sum up to 1. This is important if one read gets mapped to several putative indel loci such that the read is considered several times. For simplicity and performance reasons, our implementation of the model in Varlociraptor currently only considers the primary alignment. It is planned to optionally enable the consideration of all alternative (secondary) alignments as well. *Typing uncertainty* can be modeled as:

10 $$\begin{array}{*{20}l}  \xi_{i} &\sim \text{Bernoulli}\left(\theta \tau \right). \end{array} $$

Thereby, the allele frequency *θ*, as per its definition, reflects the probability to sample a read from a variant-affected genome copy. Further, *τ* reflects the probability that, if sampled from the variant-affected copy, the read indeed covers the variant. That is, the product *θτ* reflects to sample a fragment that is truly affected with the variant (and hence provides real evidence about the variant), see the “Sampling probability” section for how *τ* is computed. Note that *θ* and *τ* vary depending on whether *Z*_*i*_ refers to fragment data from the healthy $\left (Z_{i}^{h}\right)$ or the tumor genome ($Z_{j}^{t}$). Appropriate choices for *θ*,*τ* are specified in the paragraph about impurity below.

Whether *ξ*_*i*_=1 or *ξ*_*i*_=0 is generally not immediately evident from the observed (paired-end) read *Z*_*i*_, leading to *typing uncertainty*. We define:

11 $$  Z_{i} \mid \xi_{i}, \omega_{i} \sim \begin{cases} p_{i} &\text{ if}\ \xi_{i} = 1, \omega_{i} = 1\\ a_{i} &\text{ if}\ \xi_{i} = 0, \omega_{i} = 1\\ o_{i} &\text{ if}\ \omega_{i} = 0 \end{cases}  $$

where *a*_*i*_,*p*_*i*_, and *o*_*i*_ are the probability distributions that reflect the situation that *Z*_*i*_ stems from a genome copy in which the indel is either *p*resent or *a*bsent, or comes from an (unknown) *o*ther locus. In the last case, *Z*_*i*_ is supposed to have no influence on the posterior probability distribution of *θ*.

In order to illustrate the nature of *p*_*i*_,*a*_*i*_, and *o*_*i*_, let us consider a simplified case. The detailed definition can be found in the “Computing *a*_*i*_,*p*_*i*_, and *o*_*i*_” section. We denote two different haplotypes *H*_ref_ and *H*_var_. The former represents the reference sequence (no variant) and the latter the alternative (variant affected) sequence at the considered locus. Let *x* be a particular read. Let further:

12 $$  P_{\text{ref}}(x) \quad \text{and} \quad P_{\text{var}}(x)  $$

be the probabilities that *x* has been sampled from *H*_ref_ or from *H*_var_, respectively. Because *x* may contain errors, one needs to take the sequencing error profile of *x* into account when aiming at accurate computation of *P*_ref_(*x*) and *P*_var_(*x*). The error profile is provided via the base qualities reported by the sequencing machine. Base qualities are reported along with read alignments in BAM files (https://samtools.github.io/hts-specs/SAMv1.pdf).

As described in earlier work [43], probabilities (12) can be reliably computed by means of a Pair HMM whose parameters refer to the base quality profile of *x*, thereby appropriately accounting for the sequencing errors affecting *x*. Following this well-approved rationale, we model:

13 $$  {}a_{i}(Z_{i} = x) \stackrel{\text{def}}{=} P_{\text{ref}}(x) \quad \text{and} \quad p_{i}(Z_{i}=x) \stackrel{\text{def}}{=} P_{\text{var}}(x)  $$

Note that for the sake of a clear presentation, we have omitted the detail that *p*_*i*_,*a*_*i*_ also reflect considerations about the fragment length distributions of the involved reads, see the “Computing *a*_*i*_,*p*_*i*_, and *o*_*i*_” section for how *a*_*i*_,*p*_*i*_, and *o*_*i*_ are computed in full detail.

Finally, *o*_*i*_ reflects the case that *x* does not stem from the locus. Without further knowledge available—which is the case at the time of analysis—it is reasonable to assume that *o*_*i*_ is equal for all possible reads *x*. In other words, it is reasonable to assume that *o*_*i*_ reflects a uniform distribution. The only detail to consider (which follows from the theoretical statements listed in the “Statements” section) is that the particular value *o*(*Z*_*i*_=*x*) needs to scale right relative to *p*_*i*_(*Z*_*i*_=*x*) and *a*_*i*_(*Z*_*i*_=*x*), see again the “Computing *a*_*i*_,*p*_*i*_, and *o*_*i*_” section for the respective details.

**Part 2—Impurity.** Let *α* be the purity of the tumor genome sample. In turn, 1−*α* is the ratio of fragments stemming from a healthy genome copy, when sampled from the tumor sample. Impurity does not affect the healthy sample, so (9), (10), (11), and (13) apply without further modifications for the healthy sample: variables can be indexed with super- or subscript *h* as they appear, for example, $\omega _{i}^{h} \sim \text {Bernoulli}(\pi _{i}^{h})$ in (9) or $\xi _{i}^{h}\sim \text {Bernoulli}(\theta _{h}\tau _{h})$ in (10).

However, variables referring to the tumor sample (indexed using super- or subscript *t*) require different treatment. Let $Z_{j}^{t}, j=1,\dots,k$ be the observable read data in the tumor sample. First, note that impurity does not affect the degree of certainty of read alignments. So, (9) applies without modifications:

14 $$  \omega_{j}^{t} \sim \text{Bernoulli}\left(\pi_{j}^{t} \right)  $$

Modeling typing uncertainty however requires changes. Recalling (8), we compute:

15 $$  \xi_{j}^{t} \sim \text{Bernoulli}\left(\theta_{t}\tau_{t}\right) \stackrel{(\text{8})}{=} \text{Bernoulli}\left(\alpha \theta_{c} \tau_{t} + (1 - \alpha) \theta_{h} \tau_{t} \right)  $$

This reflects that if reads stem from a cancerous genome copy, which happens with probability *α*, variables *θ*_*c*_,*τ*_*t*_ apply, and if stemming from a healthy genome copy, which happens with probability 1−*α*, variables *θ*_*h*_,*τ*_*t*_ apply. Note that although stemming from a healthy genome copy (thus *θ*_*h*_), still *τ*_*t*_ applies, because the healthy genome copy was sampled from the tumor sample and *τ* is specific to the sample, healthy, or tumor (see the “Sampling probability” section for details).

Equations (11) and (13) remain unchanged, that is, super- and subscripts *t* can be introduced without further modifications, because impurity does not matter in these considerations.

**Part 3—Strand bias.** In the following, we will be dealing with paired-end read data. We will therefore be focusing on this case. Note however that our model also immediately applies for single-end and mate-pair read data, via some straightforward modifications.

For the sake of simplicity, let us consider reads from only the healthy sample, and for the sake of a clear presentation, we omit super- and subscripts. That is, we write $\textbf {Z}=(Z_{i})_{i=1}^{k}$ when meaning $\textbf {Z}^{h}=\left (Z_{i}^{h}\right)_{i=1}^{k}$, and we simply write *θ*,*τ* when meaning *θ*_*h*_,*τ*_*h*_. Extending the following arguments to the tumor sample is straightforward.

Following the usual protocols, in a paired-end read, one read end stems from the forward strand, while the other end stems from the reverse strand. When accounting for strand bias, it is important to track whether the forward or the reverse strand is associated with the variant, or even both of them (reflecting the situation of a self-overlapping read), because variants showing exclusively in only one of the types of ends are likely to reflect artifacts.

We model strand direction of read pairs by expanding **Z** into **Z**=(**R**,**S**), distinguishing between the read data themselves $\textbf {R}=(R_{i})_{i=1}^{k}$ and variables $\textbf {S}=(S_{i})_{i=1}^{k}$. Each of the *S*_*i*_ takes values in {−,+}, reflecting whether the reverse (−) or the forward (+) end of read pair *i* is associated with the variant. For the sake of a clear presentation, we omit the case that a paired-end read has self-overlapping ends, such that it is possible that both of its ends are associated with the variant; see the “Strand bias: technical details” section for the corresponding, straightforward modifications.

The value of *S*_*i*_ only has meaning if the *i*th read pair indeed stems from the variant haplotype, which refers to the case *ξ*_*i*_=1,*ω*_*i*_=1 in terms of the earlier latent variables. If *ω*_*i*_=0, that is the *i*th read pair does not stem from the variant locus, or if *ω*_*i*_=1,*ξ*_*i*_=0, that is, if the *i*th read pair stems from the locus but is associated with the reference haplotype, strand bias cannot affect the *i*th read pair. Realizations of *S*_*i*_ are observable: we retrieve the corresponding values from the initial standard read aligner in the obvious way. Note that it is important to retrieve the realizations from the standard alignment, but not the re-alignment, because standard, reference-based alignments are a major source of strand bias artifacts.

Integrating the *S*_*i*_, the likelihood function extends to:

$$\begin{aligned} L(\theta,\beta\mid\mathbf{Z}=(\mathbf{R},\mathbf{S})) \propto P(\mathbf{Z}=(\mathbf{R},\mathbf{S})\mid\theta,\beta) = \prod_{i=1}^{k} P(R_{i},S_{i}\mid\theta,\beta) \end{aligned} $$

So, when treating strand bias, we have to specify how to evaluate *P*(*R*_*i*_,*S*_*i*_∣*θ*,*β*). We compute:

16 $$\begin{array}{*{20}l}  P(S_{i},R_{i}\mid\theta,\beta) &= P(S_{i}\mid R_{i},\theta,\beta)\times P(R_{i}\mid\theta,\beta) \\ &= P(S_{i}\mid R_{i},\theta,\beta)\times P(R_{i}\mid\theta) \end{array} $$

where the last equality follows from the fact that only *S*_*i*_ depends on *β*, while *R*_*i*_, the observed read sequence itself, does not depend on *β*. Note that *R*_*i*_ could be identical both for (+,−)-oriented and (−,+)-oriented read pairs, while only one of those orientations gives rise to a variant artifact. Computing *P*(*R*_*i*_∣*θ*) is identical with the computations displayed for the cases not involving strand bias, see parts 1 and 2. So, it remains to consider *P*(*S*_*i*_∣*R*_*i*_,*θ*,*β*). We refer to the “Strand bias: technical details” section where we elaborate on the corresponding details; here, we conclude that (16) points out that strand bias can be handled efficiently by integrating additional factors *P*(*S*_*i*_∣*R*_*i*_,*θ*,*β*) into the overall likelihood function.

As further outlined in the last paragraph of the “Strand bias: technical details” section, the factor *P*(*S*_*i*_∣*R*_*i*_,*θ*,*β*) in (16) has no influence on the likelihood of *θ* if $\beta =\frac 12$. This is important, because only the loci where *β*=0 or 1 reflect strand bias artifacts and are to be removed from further considerations. Variants observed at the loci where $\beta =\frac 12$ are no strand bias artifacts. Therefore, the desirable scenario is to deal with them as if strand bias was not to take into account. This means that $L\left (\theta,\frac 12\mid \textbf {Z}=(\textbf {R},\textbf {S})\right)$ should be proportional to *L*(*θ*∣**Z**), as treated before introducing strand bias, see again the “Strand bias: technical details” section for further straightforward computations that prove this.

#### Computing *a*_*i*_,*p*_*i*_, and *o*_*i*_

We now specify distributions *a*_*i*_ and *p*_*i*_ for read pairs *Z*_*i*_. We note in passing that our model does in general not depend on a particular sequencing technology, so can also deal with single-end third-generation sequencing reads or other protocols, see the “Discussion” section for some final remarks on that point.

As usual, let us fix a particular locus harboring a putative variant and denote the corresponding reference haplotype of that locus by *H*_ref_ and the alternative, variant-affected haplotype of the locus by *H*_var_. We compute *H*_var_ by the application of the putative variant to the reference sequence (*H*_ref_) without any additional changes.

Let us consider a particular read pair *x*=(*x*_1_,*x*_2_), consisting of two ends *x*_1_,*x*_2_ (which in general are of equal length), that was found to align with the putative variant locus through the use of a standard read aligner (e.g., BWA-MEM, [44]). After having collected *x*, we discard all standard read alignment information about *x*, apart from one detail: we store *z*, the length of the alignment computed by the standard read aligner. Note that *z* is evaluated as the distance between the rightmost alignment coordinate of the right end (*x*_2_) and the leftmost alignment coordinate of the left end (*x*_1_), including clipped bases.

**Fig. 9:**
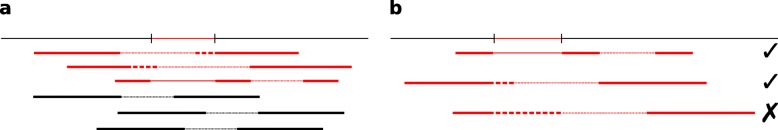
Dealing with evidence from alignments. The reference genome is displayed as a thin line at the top, with an example deletion highlighted in red. Paired-end reads are displayed as two connected thick bars. Deletions within the alignment of a read are displayed as thinner region. Soft clips in the alignment are dotted. Fragment origin is encoded by color (red = variant; black = reference). **a** Reads coming from the variant allele can be aligned with soft-clipped ends or with the variant being encoded in the alignment itself. Due to ambiguities, the positions are not necessarily perfectly aligned with the variant (see Fig. S12 (Additional file 1) for a real example). **b** Depending on the size of the variant and properties of the read mapper on a specific genome, it can become less likely to obtain fragments from the variant allele. Here, the first and the second fragments are mappable, and the third is not, because the soft clip would become too large to be considered by the read mapper

The reason to discard the standard read alignment information is as follows. Since the standard read aligner does not know the true genome underlying the sample and instead aligns against a reference genome, read alignments coming from variant alleles can be biased in multiple ways. When dealing with insertions and deletions, these biases include well-known effects referring to mistaken gap placement—note that placing gaps in alignments correctly has remained a notorious issue [20]. Incomplete gaps (soft-/hard-clipped read alignments) output by the standard aligner add to these difficulties; therefore, re-aligning clipped alignments can be of particular value, see Fig. 9a for an illustration and Fig. S12 (Additional file 1) for a real world example.

Instead of using standard read alignments, we re-align *x* with both haplotypes *H*_ref_ and *H*_var_ using a Pair HMM [45], as it was initially suggested by [43]. The Pair HMM appropriately accounts for possible sequencing errors in *x*, because its parameters reflect the sequencing error (base quality) profile of *x*, and thereby reflects that *x* has been sampled as a fragment from *H*_ref_ or *H*_var_, by providing either *x* and *H*_ref_ or *x* and *H*_var_ as input pair of sequences.

We derive values *a*_*i*_(*x*),*p*_*i*_(*x*),and *o*_*i*_(*x*) from these re-alignments, reflecting probabilities that *x* has been sampled from *H*_ref_ (*a*_*i*_) or from *H*_var_ (*p*_*i*_), while *o*_*i*_, reflecting the probability that *x* does not stem from the locus, needs to scale right with *a*_*i*_ and *p*_*i*_, in order to ensure numerical stability.

For aligning *x* with *H*_ref_ and *H*_var_ using a Pair HMM, we need to consider the issue that we need to specify exactly what *H*_ref_ and *H*_var_ should look like. Of course, *H*_ref_ and *H*_var_ cannot reflect whole-chromosome length sequences, because it is infeasible by runtime to align *x* against a full-length chromosome. Hence, we need to cut *H*_ref_ out of the full-length chromosome in an appropriate way. In particular, it is favorable that the parts of *H*_ref_ and *H*_var_ against which *x* is aligned (i.e., the parts provided as input to the Pair HMM) should be of equal length (or differ by one or two basepairs, but not differ by the length of the indel, for example), to avoid biases induced by the length of the indel under consideration.

To do this, we proceed in six steps:

1. Let *l* be the coordinate of the variant locus in the reference genome *G*. We determine *H*_ref_:=*G*[*l*−*h*,*l*+*h*], that is as a window of size 2*h* in the reference genome around the variant locus (Varlociraptor uses *h*=64 by default). We then obtain *H*_var_ by applying the variant to *H*_ref_. Note that this is not yet the input for the Pair HMM.
2. Let *x*_1_ be the left end of the read pair *x*=(*x*_1_,*x*_2_). The following computations are executed also for *x*_2_, which happens entirely analogously. Taking *x*_1_ as the pattern and *H*_ref_ or *H*_var_, respectively, as the text, we apply Myers’ bitvector algorithm [46] and obtain coordinates *a*_*r*_,*b*_*r*_ and *a*_*v*_,*b*_*v*_ determined such that, relative to edit distance, the optimal occurrence of *x*_1_ in *H*_ref_ and *H*_var_ is *H*_ref_[*a*_*r*_,*b*_*r*_] and *H*_var_[*a*_*v*_,*b*_*v*_], respectively. Note that by virtue of Myers’ bitvector algorithm, the lengths of *H*_ref_[*a*_*r*_,*b*_*r*_] and *H*_var_[*a*_*v*_,*b*_*v*_], that is, *b*_*r*_−*a*_*r*_+1 and *b*_*v*_−*a*_*v*_+1, tend to differ only by at most 1 or two basepairs, which is the desired scenario. As mentioned above, we also obtain such coordinates for the right end *x*_2_.
3. We finally specify a maximal edit distance *d*, and compute:
  17 $$\begin{array}{*{20}l} a_{i, \text{left}}(x) &:= P_{\text{HMM}}(x_{1}, H_{\text{ref}}[a_{r}-d,b_{r}+d]) \end{array} $$
  18 $$\begin{array}{*{20}l} p_{i, \text{left}}(x) &:= P_{\text{HMM}}(x_{1}, H_{\text{var}}[a_{v}-d,b_{v}+d]) \end{array} $$
  where *P*_HMM_(*x*_1_,*H*_ref_[*a*_*r*_−*d*,*b*_*r*_+*d*]) and *P*_HMM_(*x*_1_,*H*_var_[*a*_*v*_−*d*,*b*_*v*_+*d*]) are computed by means of the forward algorithm for Pair HMMs as defined by [45]. Again, probabilities *a*_*i*,right_(*x*) and *p*_*i*,right_(*x*) referring to the right read end *x*_2_ are computed in the entirely analogous way, by replacing *x*_1_ with *x*_2_ and coordinates *a*_*r*_,*b*_*r*_,*a*_*v*_,*b*_*v*_ with coordinates resulting from running Myers’ algorithm with *x*_2_ as pattern and *H*_ref_ and *H*_var_ as texts.
4. Let *f* specify the fragment length distribution referring to the sequencing library protocol in use. Here, we assume *f* to be approximately Gaussian and hence specify it by its mean *μ* and standard deviation *σ*. While, without any further adaptations, we can work with arbitrary (empirical) fragment length distributions.
  We recall that *z* is the length of the (standard) alignment of *x*. Note that *z* agrees with the length of the underlying fragment if *x* is not affected by the variant. If, however, *x* is affected by the variant, which is of length *δ*, then the length of the underlying fragment would evaluate as *z*+*δ*. Hence, we define:
  19 $$\begin{array}{*{20}l}  a_{i, \text{int}}(x) &:= f(z) \end{array} $$
  20 $$\begin{array}{*{20}l}  p_{i, \text{int}}(x) &:= f(z+\delta) \end{array} $$
  to reflect that *a*_*i*,int_ is supposed to reflect that alignment and fragment length agree, while *p*_*i*,int_ is supposed to reflect that they differ by the length of the variant.
5. We combine the evidence for the left and right read with the insert size by calculating:
  21 $$\begin{array}{*{20}l} a_{i}(x) &:= a_{i, \text{left}}(x) \times a_{i, \text{int}}(x) \times a_{i, \text{right}}(x) \end{array} $$
  22 $$\begin{array}{*{20}l} p_{i}(x) &:= p_{i, \text{left}}(x) \times p_{i, \text{int}}(x) \times p_{i, \text{right}}(x). \end{array} $$
6. Finally, we denote:
  23 $$ o_{i}(x) := \frac12(a_{i}(x) + p_{i}(x)).  $$
  The formal justification for determining *o*_*i*_(*x*) as such follows from the proofs provided in the “Statements” section, which lists the formal statements that make the theoretical foundation of our model.

Note that we discard all reads from being considered if neither the left read end (*x*_1_) nor the right read end (*x*_2_) overlap the variant locus as per their Pair HMM-based re-alignments.

Finally, let us revisit the decision to combine evidences of left and right reads with the insert size. Splitting up of *a*_*i*_,*p*_*i*_ into read end-specific factors would effectively weaken the variant-related signals from reads that can only be ambiguously placed, that is whose exact placement remains dubious even after re-alignment. To understand the advantage of combining evidences, consider a read *x* whose first end supports the variant, which yields large *p*_*i*,left_(*x*), but small *a*_*i*,left_(*x*), whereas the second end supports the reference, which yields large *a*_*i*,right_(*x*), but small *p*_*i*,right_(*x*). In consequence, overall, both *a*_*i*_(*x*) and *p*_*i*_(*x*) are approximately equal. The same argument holds, for example, if one or both reads support the variant, but the insert size does not. Following our model, reads *x* with (approximately) equal *a*_*i*_(*x*) and *p*_*i*_(*x*) yield an approximately uniform probability distribution with respect to *θ*, the allele frequency of the variant, which is just our prior distribution on *θ*. In other words, *x* does not contribute to making a statement in favor of a particular (range of) *θ*, which is the correct scenario.

#### Sampling probability

See Fig. 9b for illustrations of the following. In the initial step, we select reads using a standard read aligner, which determines placement of reads by mapping them against the reference genome. Because differences between non-variant-affected reads and the reference sequence are small, the standard read aligner will be able to align all (or at least nearly all) of such reads successfully against the putative variant locus. The situation is different for reads that are affected by the variant. Because such reads differ from the reference sequence by, in our case, an insertion or deletion of considerable length, the standard read aligner may fail to align such reads successfully. Such failure occurs in particular if variant size and placement within the read interfere unfavorably with the alignment procedure. As a consequence, there can be a bias towards reads from the reference allele.

The effect was first systematically treated in [47]. The corresponding quantification means to assume that non-variant-affected reads align against the locus with probability 1, while variant-affected reads align with *sampling probabilityτ*, which is smaller than 1. The sampling probability *τ* is a locus-, donor genome-, and sequencing-specific parameter, because *τ* depends on the sequence context, which varies relative to the locus considered and also relative to the donor genome investigated as well as the used sequencing protocol (in terms of read length and targeted insert size). So, *τ* can be different for cancer and control genome, which we make explicit by dealing with *τ*_*h*_ and *τ*_*t*_, referring to the healthy (*h*) and tumor (*t*) genome.

In the following, we briefly specify how *τ* is computed based on the considerations made by [47]. First, *τ* depends on the fragment length distribution *f*, which can be retrieved from the read aligner, see also the explanations referring to formulas (19) and (20) above. Further, *τ* depends on the number of bases *δ* the deletion or insertion covers in the donor genome (deletion: *δ*=0; insertion: *δ*= length of insertion). It also depends on the number of bases the aligner requires to be aligned with the reference genome sequence upstream or downstream of the variant, in the upstream (left) or the downstream (right) read end, respectively, which are referred to as *k*_up_ and *k*_down_. This reflects that it is critical for a read aligner to get the outer ends of a paired-end read mapped. Values *k*_up_ and *k*_down_ depend on the read aligner and the variant. In the following, *k* is one of *k*_up_ or *k*_down_, depending on whether we consider the information referring to the upstream or downstream read end; when dealing with single-end reads (see below), *k* is unique. *k* can be estimated by inspecting a representative subset of all aligned reads of a sample, and recording the maximum deletion and insertion size encoded in the CIGAR strings [48] of this subset of reads, as well as the maximum soft clip size, retrieved from partial alignments from that representative subset of reads. If the variant is smaller than the maximum deletion or insertion encoded in the CIGAR strings, we can set *k*=0 because the variant is small enough to be part of the alignment. Otherwise, *k* can be set to the read length minus the maximum soft clip size, that is, the smallest observed partial alignment.

Let further *o* denote the fragment length (which is unknown) and *f* denote the fragment length distribution (see the “Computing *a*_*i*_,*p*_*i*_, and *o*_*i*_” section). Following [47], we compute *τ* as:

24 $$\begin{array}{*{20}l} \tau_{s} = \sum_{o=1}^{\infty} f(o) \frac{o - \delta - k_{\text{up}} - k_{\text{down}}}{o - \delta},  \end{array} $$

where *s*=*h*,*t* specifies the healthy or tumor sample, see above; note that all of *f*,*k*_up_, and *k*_down_ differ relative to the sample. The formula reflects that one sums over all possible fragment lengths, weighted by the probability to sample a fragment of that length, and calculates the probability to get a fragment of that length aligned, relative to the relevant parameters *δ*,*k*_up_, and *k*_down_. Note that in case of single end reads, (24) simplifies to:

25 $$\begin{array}{*{20}l} \tau_{s} = \frac{r - \delta - k}{o - \delta},  \end{array} $$

with *r* being the read length.

#### Strand bias: technical details

In the following, observable strand bias variables *S*_*i*_ will take values in {−,+,±} to also reflect the case that a paired-end read has self-overlapping reads both of which are affected by the variant (±). Note that existence of a ± read rules out strand bias, because it means that read ends from both strands are affected by the variant.

Let us define:

26 $$\begin{array}{*{20}l} A := \{\omega_{i}=1,\xi_{i}=1\} \end{array} $$

27 $$\begin{array}{*{20}l} B := \{\omega_{i}=1,\xi_{i}=0\} \end{array} $$

28 $$\begin{array}{*{20}l} C := \{\omega_{i} = 0\} \end{array} $$

29 $$\begin{array}{*{20}l} D := B \;\dot{\cup}\; C \end{array} $$

and recall that strand bias can only occur if event *A* applies, that is, if the read is indeed associated with the variant. Note that *A* and *D* span the entire space of cases (*ω*_*i*_,*ξ*_*i*_)∈{0,1}×{0,1}, which allows to apply the law of total probability. We obtain:

30 $$  P(S_{i}\mid Z_{i},\theta,\beta) = P(S_{i},A\mid Z_{i},\theta,\beta) + P(S_{i},D\mid Z_{i},\theta,\beta)  $$

So, we will continue to put further focus on the two summands in (30). Let *z*_*i*_ be the observed realization (the read itself including its error profile) of *Z*_*i*_. For a clear presentation of what follows, we define:

31 $$\begin{array}{*{20}l} \gamma_{A}(Z_{i}) :=& \ p_{i}(z_{i})\pi_{i}\theta\tau\quad\text{and}\\ \gamma_{D}(Z_{i}) =& \ a_{i}(z_{i})(\pi_{i}(1-\theta\tau)) + o_{i}(z_{i})(1-\pi_{i}). \end{array} $$

For the first summand from (30), we compute:

32 $$ \begin{aligned} P(S_{i},A\mid Z_{i},\theta,\beta) &= P(S_{i}\mid A,Z_{i},\theta,\beta)\cdot P(A\mid Z_{i},\theta,\beta)\\ &= P(S_{i}\mid A,\beta) \cdot P(A\mid Z_{i},\theta) \end{aligned}  $$

where the last equation follows from the fact that, on the one hand, given *A* and *β*, *S*_*i*_ is independent of *Z*_*i*_ and *θ* (note that all information *S*_*i*_ depends on about *Z* and *θ* is captured by *A*) and, on the other hand, *A* is independent of *β*, the strand bias. We continue:

$$\begin{aligned} P(A\mid Z_{i},\theta) &= \frac{P(A,Z_{i}\mid \theta)}{P(Z_{i})} = \frac{1}{P(Z_{i})}P(Z_{i}\mid A,\theta)P(A\mid\theta)\\ &= \frac{1}{P(Z_{i})}P(Z_{i}\mid A)P(A\mid\theta) = \frac{1}{P(Z_{i})}\gamma_{A}(Z_{i}) \end{aligned} $$

where the second to last equation follows from the dependency structure of the model, because *Z*_*i*_ is independent of *θ* given *A*, and the last equation follows from the fundamental considerations of part 1. Note at last that it is reasonable (and common in analogous settings) to assume that *P*(*Z*_*i*_), the prior probability to observe a read is constant across all reads, so $\frac {1}{P(Z_{i})}$ turns out to be a constant. In summary, we obtain:

33 $$ P(S_{i},A\mid Z_{i},\theta,\beta) \propto \gamma_{A}(Z_{i})\cdot P(S_{i}\mid A,\beta).  $$

Recalling that read pairs were selected where at least one of the ends overlapped the locus, let *q*_1_ and *q*_2_=1−*q*_1_ be the probabilities to have one or both reads overlapping the variant locus. Probabilities *q*_1_,*q*_2_ can be determined from the insert size distribution provided by the read mapper, in combination with the length of the read ends. Note that usually *q*_1_>>*q*_2_ and that for single-end sequencing, obviously *q*_1_=1,*q*_2_=0. Our considerations are then finalized by providing the table:

34 $$ P(S_{i} \mid A,\beta) = \left\{\begin{array}{ll} 1 &\text{if }\left\{\begin{array}{ll} S_{i} = +, \beta = 1 &\\ S_{i} = -,\beta = 0& \end{array}\right.\\ \frac12 q_{1} &\text{if}\quad S_{i}\ne\pm, \beta=\frac12\\ q_{2} &\text{if }\quad S_{i} = \pm, \beta=\frac12\\ 0 &\text{if }\left\{\begin{array}{ll} S_{i} = +,\beta = 0&\\ S_{i} = -,\beta = 1&\\ S_{i} = \pm, \beta\ne\frac12.&\end{array}\right. \end{array}\right.  $$

By entirely analogous considerations, we also obtain:

35 $$ P(S_{i},D\mid Z_{i},\theta,\beta) \propto \gamma_{D}(Z_{i})\cdot P(S_{i}\mid D,\beta)  $$

where here, however:

36 $$ \begin{aligned} P(S_{i} \mid D,\beta) = \frac13 \quad\text{ for all combinations of realizations of }S_{i},\beta \end{aligned}  $$

As an exemplary case, consider *γ*_*A*_(*Z*_*i*_)=0,*γ*_*D*_(*Z*_*i*_)=1, reflecting the case that the read is not associated with the variant with probability one. Here, we obtain that $P(S_{i}\mid Z_{i},\theta,\beta)\propto \frac 13\gamma _{D}(Z_{i})$ for all choices of *β*. This means that the *i*th read assigns equal likelihood to all choices of *β*, which is the correct scenario, because the *i*th read cannot provide any information about *β*.

Consider also the opposite case, *γ*_*A*_(*Z*_*i*_)=1,*γ*_*D*_(*Z*_*i*_)=0, reflecting full certainty about the read being associated with the variant. If for example *S*_*i*_=+, the likelihood for *β*=0 is 0. Further (recalling that usually *q*_1_>>*q*_2_, meaning that *q*_1_ is close to one), the likelihood for *β*=1 is approximately double the amount as for $\beta =\frac 12$. This again is the correct scenario.

Finally, we observe that if $\beta =\frac 12$, the factor *P*(*S*_*i*_∣*Z*_*i*_,*θ*,*β*) in (16) has no influence on the likelihood of *θ*. This is important, because we will only consider the loci where $\beta =\frac 12$ with large enough likelihood, and for such loci, we will base our further decisions on the evaluation of $L(\theta,\frac 12\mid \textbf {Z},\textbf {S})$. It is therefore essential that $L(\theta,\frac 12\mid \textbf {Z},\textbf {S})$ is proportional to *L*(*θ*∣**Z**), that is when not considering strand bias. Considering strand bias should only lead to excluding variant artifacts—if variants are supposed to be real, strand bias considerations should not have an influence on decisions about whether variants are somatic or not, or, more specifically, about what their allele frequencies are.

To understand this, note that the only appearance of *θ* in the computation of *P*(*S*_*i*_∣*Z*_*i*_,*θ*,*β*) is in the factors *γ*_*A*_(*Z*_*i*_) and *γ*_*D*_(*Z*_*i*_). Note further that these factors determine to what degree the *i*th read makes a statement about *β*: the larger *γ*_*A*_(*Z*_*i*_) relative to *γ*_*D*_(*Z*_*i*_), the stronger the statement. If $\beta =\frac 12$, however, both *P*(*S*_*i*_∣*A*,*β*) and *P*(*S*_*i*_∣*D*,*β*) evaluate as $\frac 12$, independently of the particular realization of *S*_*i*_. Therefore, if $\beta =\frac 12$, varying *θ* does not vary *P*(*S*_*i*_∣*Z*_*i*_,*θ*,*β*). Of course, still, varying *θ* varies (16) because it varies *P*(*Z*_*i*_∣*θ*). So, *θ* varies $L(\theta,\frac 12\mid \textbf {Z},\textbf {S})$, just as if *β* was not considered. In summary, this is the desired scenario.

#### Statements

We finally obtain the result outlined in the “Foundation of the approach” section as a corollary to the following theorem.

##### **Theorem 3**

Let $\boldsymbol {Z}^{h}=\left (Z_{1}^{h},...,Z_{k}^{h}\right),\boldsymbol {Z}^{t}=\left (Z_{1}^{t},...,Z_{l}^{t}\right)$ be the observable read data from a healthy and a tumor sample, covering the locus of a putative variant. Then:

- (*i*) The likelihood function
  $$\begin{array}{*{20}l}  {} \begin{aligned} L(\theta_{h},\theta_{c},\beta \mid\boldsymbol{Z}^{h},\boldsymbol{Z}^{t}) =& \prod_{i=1}^{k}L\left(\theta_{h},\theta_{c},\beta \mid Z_{i}^{h}\right)\\ &\times\prod_{j=1}^{l}L\left(\theta_{h},\theta_{c},\beta \mid Z_{j}^{t}\right) \end{aligned} \end{array} $$
  factors into likelihood functions referring to individual read pairs.
- (*ii*) Let *Z*_*i*_ refer to any of the read data $Z_{1}^{h},...,Z_{k}^{h},Z_{1}^{t},...,Z_{l}^{t}$ and let *ω*_*i*_,*ξ*_*i*_ be its latent uncertainty hyperparameters. Then:
  37 $$ {}\begin{aligned}  L(\theta_{h},\theta_{c},\beta \mid Z_{i}) &= P(Z_{i}\mid\theta_{h},\theta_{c},\beta)\\ &= \int_{\xi_{i},\omega_{i}}P(Z_{i} \mid \xi_{i},\omega_{i})\times P(\xi_{i},\omega_{i}\mid \theta_{h},\theta_{c},\beta)\;d({\xi_{i}},\omega_{i}) \end{aligned}  $$

##### *Proof*

(*i*) follows immediately from the fact that the $Z_{i}^{h},Z_{j}^{t}$ are conditionally independent given *θ*_*h*_,*θ*_*c*_,*β*; see Fig. 8b. (*ii*) follows from application of the Chapman-Kolmogorov equation, in combination with the dependency relationships captured by our model, see again Fig. 8b. □

We distinguish between read data $Z_{i}^{h}$ from the healthy sample and read data $Z_{j}^{t}$ from the tumor sample. In the following, we make use of the fact that $\omega _{i}^{h}$ and *θ*_*h*_ are independent (see Fig. 8b). Let $s_{i}^{h} = P\left (S_{i}^{h} | \omega _{i}^{h}=1, \xi _{i}^{h}=1, \beta \right)$ be the likelihood of the strand bias given read pair *i* as defined in Eq. (34). We compute the likelihood function for $Z_{i}^{h} = \left (R_{i}^{h}, S_{i}^{h}\right)$, which does not depend on *θ*_*c*_, as:

38 $$ {}\begin{aligned} P\!\left(\!Z_{i}^{h}\!\mid\!\theta_{h},\theta_{c}, \beta\!\right) &=\int_{\xi_{i}^{h},\omega_{i}^{h}}P\left(\!Z_{i}^{h}\!\mid\! \xi_{i}^{h},\omega_{i}^{h}, \beta\!\right)\!\times P\left(\!\xi_{i}^{h},\omega_{i}^{h}\!\mid\! \theta_{h},\theta_{c}\!\right)\,d\left(\xi_{i}^{h},\omega_{i}^{h}\right)\\ &= \int_{\xi_{i}^{h},\omega_{i}^{h}}P\left(Z_{i}^{h}\mid \xi_{i}^{h},\omega_{i}^{h}, \beta\right)\!\times P\left(\!\xi_{i}^{h},\omega_{i}^{h}\mid \theta_{h}\right)\,d\left(\xi_{i}^{h},\omega_{i}^{h}\right)\\ &= P\left(Z_{i}^{h}\mid\omega_{i}^{h}\,=\,1,\xi_{i}^{h}=1,\beta\right)P\left(\omega_{i}^{h}\,=\,1,\xi_{i}^{h}\,=\,1\!\mid\!\theta_{h}, \beta\right) +\\ &\quad~ P\left(Z_{i}^{h}\mid\omega_{i}^{h}\,=\,1,\xi_{i}^{h}\,=\,0,\beta\right)P\left(\omega_{i}^{h}\,=\,1,\xi_{i}^{h}\,=\,0\mid\theta_{h}, \beta\right) +\\ &\quad~ P\left(Z_{i}^{h}\mid\omega_{i}^{h}=0,\beta\right)P\left(\omega_{i}^{h}=0\right)\\ &= p_{i}^{h} s_{i}^{h}\left(\theta_{h} \tau_{h} \right)\pi_{i}^{h} + a_{i}^{h} \nicefrac{1}{3}(1-\theta_{h} \tau_{h})\pi_{i}^{h} \!+ o_{i}^{h} \nicefrac{1}{3}\left(1-\pi_{i}^{h}\right)\\ &= \underbrace{\pi_{i}^{h} \left(\underbrace{\theta_{h} \tau_{h} p_{i}^{h} s_{i}^{h}}_{\text{present}} + \underbrace{(1 - \theta_{h} \tau_{h}) a_{i}^{h} \nicefrac{1}{3}}_{\text{absent}} \right)}_{\text{correctly mapped}} + \underbrace{\left(1 - \pi_{i}^{h}\right) o_{i}^{h} \nicefrac{1}{3}}_{\text{incorrectly mapped}}. \end{aligned}  $$

Analogously, while slightly more involved due to the purity considerations causing $Z_{j}^{t} = \left (R_{i}^{t}, S_{i}^{t}\right)$ to depend on both *θ*_*h*_ and *θ*_*c*_, we compute:

39 $$ {}\begin{aligned} P\!\left(Z_{i}^{t}\!\mid\!\theta_{h},\theta_{c}, \!\beta\right) &=\int_{\xi_{j}^{t},\omega_{j}^{t}}P\left(Z_{j}^{t}\mid \xi_{j}^{t},\omega_{j}^{t},\beta\right)\times P\left(\xi_{j}^{t},\omega_{j}^{t}\mid \theta_{h},\theta_{c}\right)\,d\left(\xi_{j}^{t},\omega_{j}^{t}\right)\\ &= P\left(Z_{j}^{t}\!\mid\omega_{j}^{t}\!=1,\xi_{j}^{t}=1,\beta\right)P\left(\omega_{j}^{t}=1,\xi_{j}^{t}=1\!\mid\theta_{h}, \theta_{c}\right)+\\ &\quad~ P\left(Z_{j}^{t}\!\mid\omega_{j}^{t}\!=1,\xi_{j}^{t}=0,\beta\right)P\left(\omega_{j}^{t}\!=1,\xi_{j}^{t}=0\!\mid\theta_{h}, \theta_{c}\right) + \\ &\quad~ P\left(Z_{j}^{t}\mid\omega_{j}^{t}=0,\beta\right)P\left(\omega_{j}^{t}=0\right)\\ &=\pi_{i}^{t} \left(\underbrace{ \alpha \left(\underbrace{ \theta_{c} \beta \tau_{t} p_{i}^{t} s_{i}^{t} }_{\text{present}} + \underbrace{(1 - \theta_{c} \tau_{t}) a_{i}^{t} \nicefrac{1}{3}}_{\text{absent}} \right)}_{\text{from cancer cell}} + \right.\\ &\quad\left.\underbrace{(1 \!- \alpha) \big(\underbrace{ \theta_{h} \beta \tau_{h} p_{i}^{t} s_{i}^{t} }_{\text{present}} + \underbrace{(1 \,-\, \theta_{h} \tau_{h}) a_{i}^{t} \nicefrac{1}{3}}_{\text{absent}} \!\big)}_{\text{from healthy cell}} \!\right) \,+\, \left(1 \,-\, \pi_{i}^{t}\right) \!o_{i}^{t} \nicefrac{1}{3}, \end{aligned}  $$

with $s_{i}^{t} = P\left (S_{i}^{t} \mid \omega _{i} = 1, \xi _{i}=1, \beta \right)$ analogously to the healthy case above. As can be seen, the integral turns into a sum over the three cases {*ω*_*i*_=0},{*ω*_*i*_=1,*ξ*_*i*_=0}, {*ω*_*i*_=1,*ξ*_*i*_=1}, reflecting that *Z*_*i*_ is either (1) incorrectly mapped, (2) correct and not affected by the variant, or (3) correct and affected by the variant. For computing *p*_*i*_, *a*_*i*_, and *o*_*i*_, we need a linear runtime in the length of the considered window, since we are using a banded Pair HMM (see the “Computing *a*_*i*_,*p*_*i*_, and *o*_*i*_” section). However, this only needs to be done once for all likelihood computations in the parameter space. We can therefore infer the following central corollary.

##### **Corollary 1**

*L*(*θ*_*h*_,*θ*_*c*_,*β*∣***Z***^*h*^,***Z***^*t*^) can be computed in *O*(*k*+*l*) operations.

## Supplementary information

**Additional file 1** Supplement: This contains all supplementary sections and figures referenced in the main text.

**Additional file 2** Snakemake Report: A *Snakemake report* that allows to interactively explore all figures shown in this article in the context of workflow, parameters, software versions, and code.

**Additional file 3** Review History.
