# Supplementary material for: Varlociraptor: enhancing sensitivity and controlling false discovery rate in somatic indel discovery
Source: Genome Biol. 2020 Apr 28;21:98. doi: 10.1186/s13059-020-01993-6 (PMC7187499; doi:10.1186/s13059-020-01993-6)
Supplement: Supplementary file 1 — Additional file 1 Supplement: This contains all supplementary sections and figures referenced in the main text. [file 13059_2020_1993_MOESM1_ESM.pdf]

Supplement:
Varlociraptor: Enhancing sensitivity and controlling false discovery rate in somatic indel discovery

S1 Why naive approaches to compute the likelihood function fail.

To understand why *efficient computation of equation (1) is difficult*, consider that each of the reads Z_i^h, Z_j^t could

(a) not stem from the particular variant locus,

(b) stem from the locus, but is not affected by the variant,

(c) stem from the locus, and is indeed affected by the variant.

We recall that it can be particularly difficult to be certain about (a), (b) or (c) when dealing with reads being associated with midsize indel loci (30-250 bp; sometimes termed the "NGS twilight zone"). Let $k = |\boldsymbol{Z}^t|$ and $l = |\boldsymbol{Z}^h|$ be the read coverage of the locus in the tumor and the healthy sample. Since there are 3 different possibilities—namely (a), (b) or (c)—for the overall $k + l$ reads, we obtain that there are 3^{k+l} different scenarios that could reflect the truth, all of which apply with a particular probability. For computing equation (1) following a *fully Bayesian approach to inverse uncertainty quantification* [1]—which is the approved and canonical way to quantify uncertainties in our setting—one needs to integrate over all the possible $k+l$ choices. In a naive approach, this translates into computing a sum with 3^{k+l} summands. Because $k + l$ amounts to at least 60 to 70 in standard settings, naive approaches fail to compute the integral in human feasible runtime. This is further aggravated because one usually needs to consider hundreds of thousands of putative indel loci. *So, methodical efforts are required for uncertainty quantification in our setting.*

S2 Uniqueness and computation of the maximum likelihood estimate

The likelihood function of θ_h, θ_c, and β given the data $\boldsymbol{Z}^h$ and $\boldsymbol{Z}^t$ as shown in equation (1) is a higher-order polynomial, which makes it infeasible to derive its maximum analytically. We show in this section, however, by proving Theorem 3.2 that under weak conditions the likelihood function attains a unique global maximum on the unit interval for each value of θ_h and β. We, in addition, show that the loglikelihood function is strictly concave, which simplifies the numerical maximization.

Proof. The likelihood function with θ_h and β fixed can be written in the form

$$L(\theta_h, \theta_c, \beta \mid \boldsymbol{Z}^h, \boldsymbol{Z}^c) = C \times \prod_{j=1}^{l} P\left(Z_j^t \mid \theta_h, \theta_c, \beta\right) \tag{1}$$

where C is the constant

$$C \equiv \prod_{i=1}^{k} P(Z_i^h \mid \theta_h, \beta).$$

In the case that theorem condition *1* is *not* met, $C = 0$. The likelihood $L(\theta_h, \theta_c, \beta \mid \boldsymbol{Z}^h, \boldsymbol{Z}^c)$ equals zero for all θ_c and, therefore, does not attain a unique global maximum.

Suppose theorem condition *1* is met ($C > 0$). Let us consider theorem condition *2*. Note that $L(\theta_h, \theta_c, \beta \mid \boldsymbol{Z}^h, \boldsymbol{Z}^c) = 0$ when $\theta_c \notin I$, since for those θ_c's there exists an observation for which the $P(Z_j^t \mid \theta_h, \theta_c\beta) = 0$. The likelihood L is by definition strictly larger than zero when $\theta_c \in I$. Since the function in equation (41) is an l-th order polynomial and, therefore, continuous, it must attain a global maximum on the interval I.

Suppose theorem condition *2* is met. The point $\widehat{\theta}_c$ is a maximum of $L(\theta_h, \cdot, \cdot \mid \boldsymbol{Z}^h, \boldsymbol{Z}^c)$ if and only if it is a maximum of the loglikelihood function

$$\ell\left(\theta_h, \theta_c, \beta \mid \boldsymbol{Z}^h, \boldsymbol{Z}^c\right) \equiv \log L(\theta_h, \theta_c, \beta \mid \boldsymbol{Z}^h, \boldsymbol{Z}^c) = \log C + \sum_{j=1}^{l} \log P(Z_j^t \mid \theta_h, \theta_c, \beta) \tag{2}$$

(with θ_h, β fixed and $\theta_c \in I$) since the logarithm is a monotonic transform. (Note that ℓ is only defined on the subset I). The second order derivative of the loglikelihood with respect to θ_c is found to be

$$\frac{\partial^2 \ell}{\partial \theta_c^2} = -\sum_{j=1}^{l} \left[\frac{\partial P(Z_j^t \mid \theta_h, \theta_c, \beta)/\partial \theta_c}{P(Z_j^t \mid \theta_h, \theta_c, \beta)}\right]^2 \leq 0 \tag{3}$$

indicating that the loglikelihood function is concave. Note that it is strictly concave, i.e., $\partial^2 \ell / \partial \theta_c^2 < 0$, iff there exists an observation z_j^t for which

$$\frac{\partial P(Z_j^t \mid \theta_h, \theta_c, \beta)}{\partial \theta_c} = \alpha \pi_j^t \tau_t \left[p_j^t s_j^t - a_j^t\right] \neq 0. \tag{4}$$

This inequality holds only when $\alpha \neq 0$, $\pi_j^t \neq 0$ and $p_j^t \neq a_j^t$, which constitutes theorem conditions *3* and *4*.

Suppose I is the non-empty closed set $[a, b]$ on the unit interval. Since the loglikelihood is strictly concave when theorem conditions *3* and *4* are met, it attains a unique global maximum $\widehat{\theta}_c$ on I. Because the logarithm is a monotonic transformation, $\widehat{\theta}_c$ must be a unique global maximum of the likelihood function as well.

A similar reasoning holds when I is open or half-open. The maximum must lie on the interior of I, since the likelihood function is zero for those endpoints not in I. For example, when I is the open interval (a, b), then $L(\theta_h, a, \beta \mid \boldsymbol{Z}^h, \boldsymbol{Z}^c) = L(\theta_h, b, \beta \mid \boldsymbol{Z}^h, \boldsymbol{Z}^c) = 0$ while $L(\theta_h, \theta_c, \beta \mid \boldsymbol{Z}^h, \boldsymbol{Z}^c)$ is strictly positive on I. The loglikelihood function is under theorem conditions *3* and *4* strictly concave on I, therefore, the likelihood function attains a unique global maximum. □

S3 Supplementary Figures

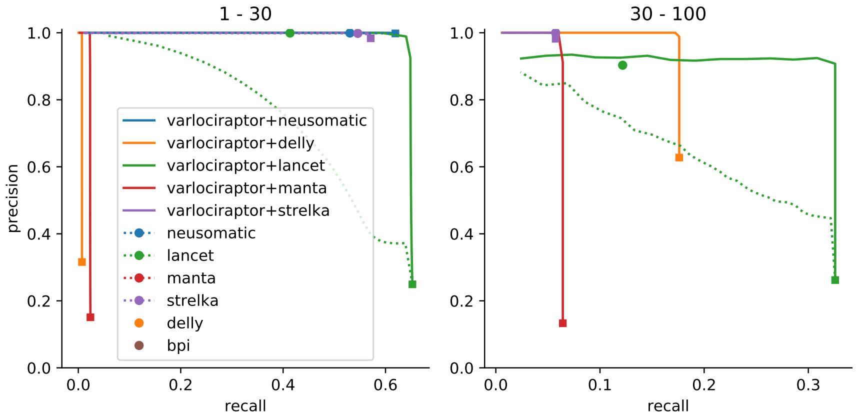

Fig. S1: Recall and precision for calling somatic insertions on simulated data. Results are grouped by deletion length, denoted as interval at the top of the plot. For our approach (Varlociraptor+*) curves are plotted by scanning over the posterior probability for having a somatic variant (for readability, each curve is terminated by a square mark). For other callers that provide a score to scan over (e.g. p-value for Lancet) we plot a dotted line. Ad-hoc results are shown as single dots. Results are shown if the prediction of the caller did provide at least 10 calls. The sharp curves for our approach reflect the favorable property of having a strong separation between the probabilities of true and false positives, see Figure **??**.

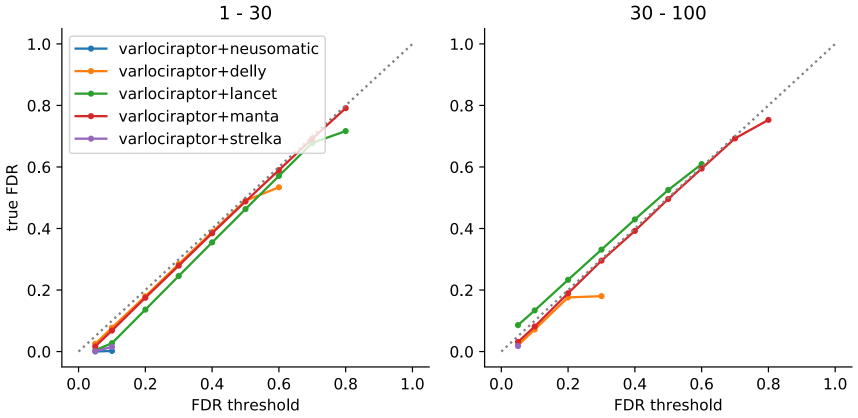

Fig. S2: FDR control for somatic insertions. Results are grouped by deletion length, denoted as interval at the top of the plot. The axes denote the desired FDR, provided by the user as input (x-axis), and the true achieved FDR (y-axis). A perfect FDR control would keep the curve exactly on the dashed diagonal. Below the diagonal, the control is conservative. Above the diagonal, the FDR would be underestimated. Importantly, points below the diagonal mean that the true FDR is smaller than the threshold provided, which means that FDR control is still established; in this sense, points below the diagonal are preferable over points above the diagonal.

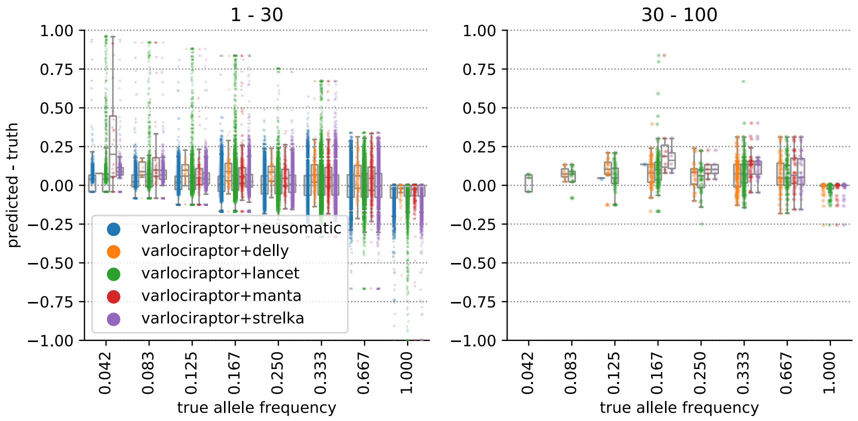

Fig. S3: Allele frequency estimation for somatic insertions. Results are grouped by deletion length, denoted as interval at the top of the plot. The horizontal axis shows the true allele frequency, the vertical axis shows the error between predicted allele frequency and truth.

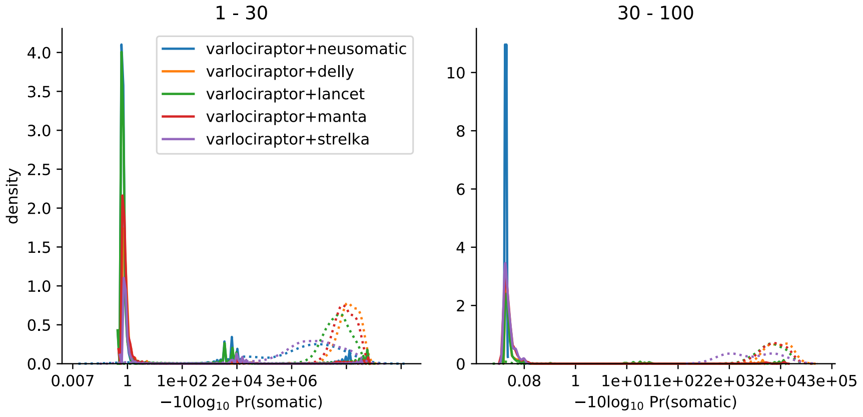

Fig. S4: Posterior probability distributions for somatic insertions. Results are grouped by deletion length, denoted as interval at the top of the plot. The x-axis indicates the (PHRED-scaled) probability, and the y-axis indicates relative amounts of calls with this probability. The distributions of posteriors for true positive calls are shown as solid lines, the distributions of posteriors for false positive calls are shown as dotted lines.

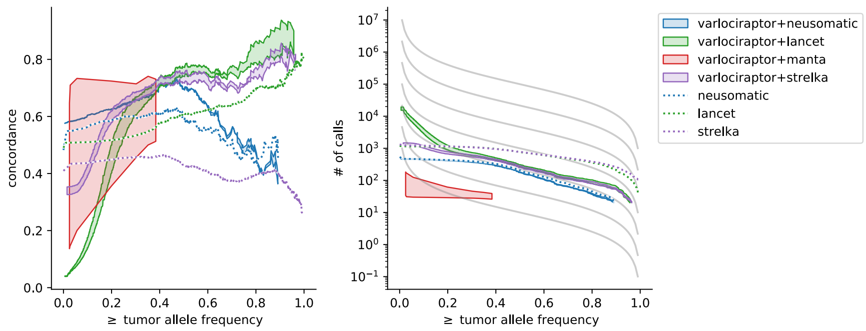

Fig. S5: Concordance of somatic insertions on real data. For Varlociraptor, the interval between all calls with a posterior probability of at least 0.9 and at least 0.99 is shown as shaded area. Left: Concordance vs. minimum allele frequency. Right: Number of calls vs. minimum allele frequency. Grey lines depict the theoretical expectation according to Williams et al. [2]

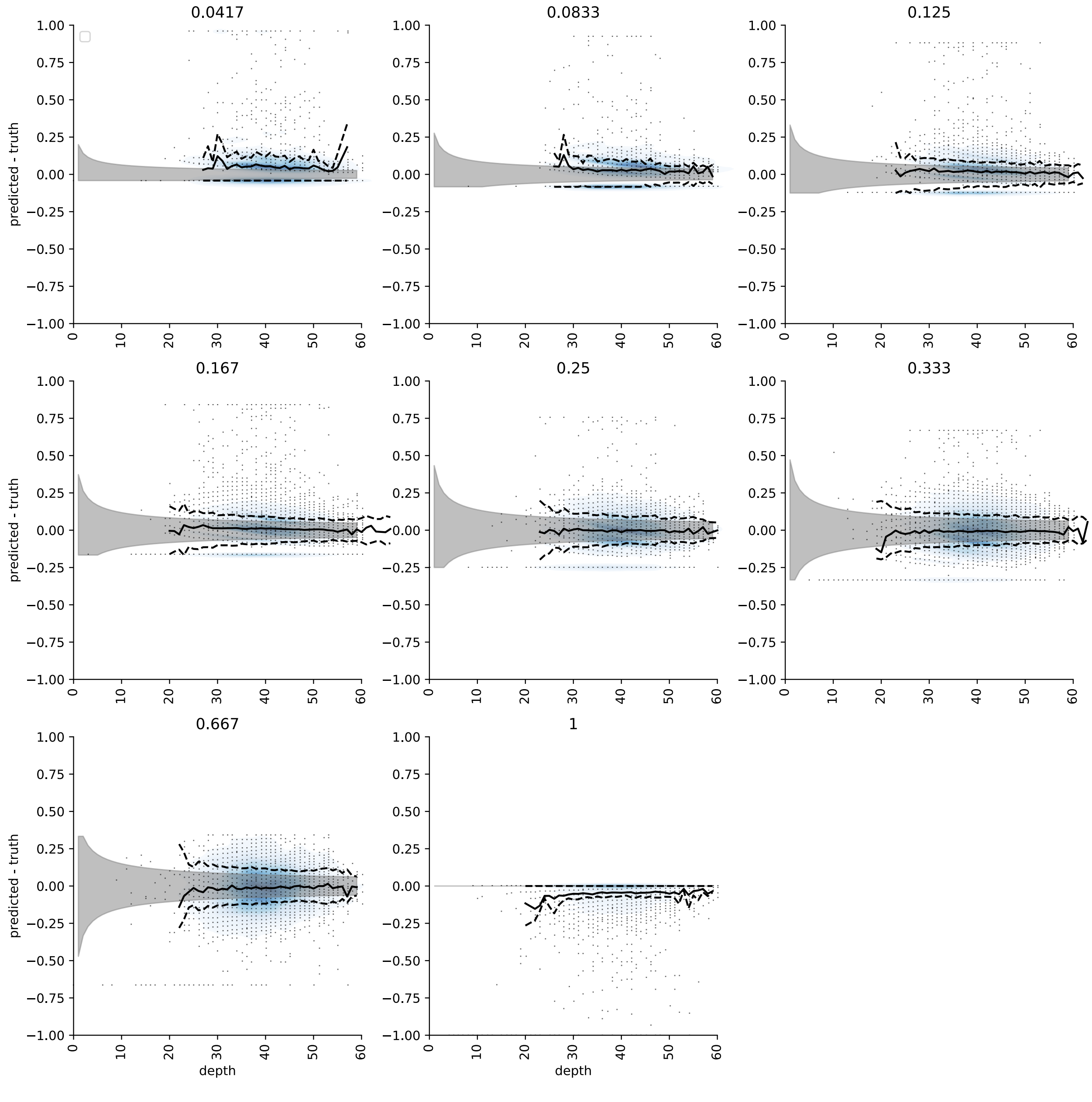

Fig. S6: Allele frequency estimation error for somatic deletions compared to sequencing depth. Each plot shows the error (predicted - truth) for a particular true allele frequency (shown above the plot). Dots represent individual predictions, the blue shading shows a corresponding density estimate. The black line shows the mean, the dashed lines depict the standard deviation. The grey area represents the theoretically expected sampling error in an experiment with no further artifacts or biases (the theoretical optimum).

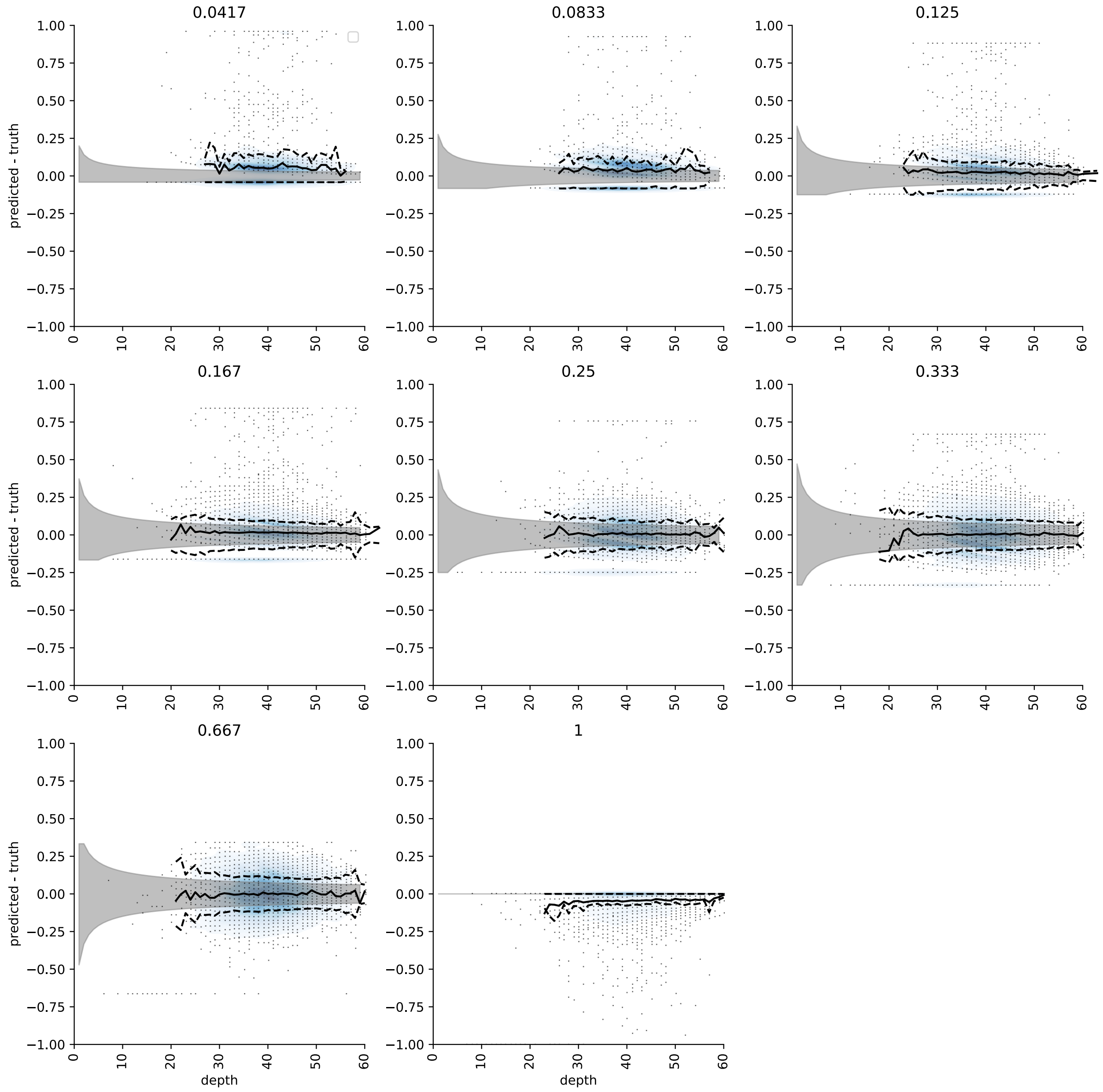

Fig. S7: Allele frequency estimation error for somatic insertions compared to sequencing depth. Each plot shows the error (predicted - truth) for a particular true allele frequency (shown above the plot). Dots represent individual predictions, the blue shading shows a corresponding density estimate. The black line shows the mean, the dashed lines depict the standard deviation. The grey area represents the theoretically expected sampling error in an experiment with no further artifacts or biases (the theoretical optimum).

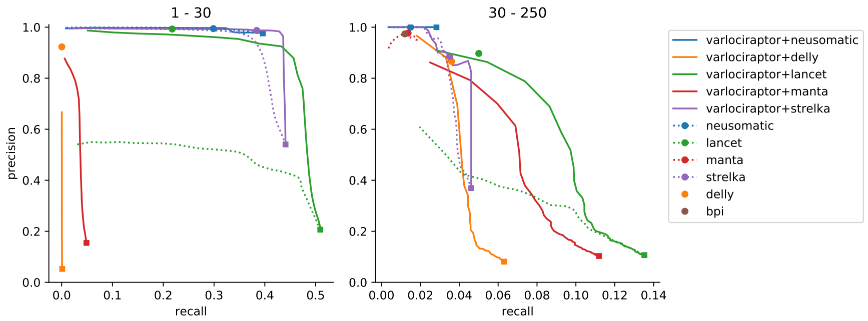

Fig. S8: Recall and precision for calling somatic deletions on synthetic data (mixture rate 20%). Results are grouped by deletion length, denoted as interval at the top of the plot. For our approach (Varlociraptor+*) curves are plotted by scanning over the posterior probability for having a somatic variant (for readability, each curve is terminated by a square mark). For other callers that provide a score to scan over (e.g. p-value for Lancet) we plot a dotted line. Ad-hoc results are shown as single dots. Results are shown if the prediction of the caller did provide at least 10 calls.

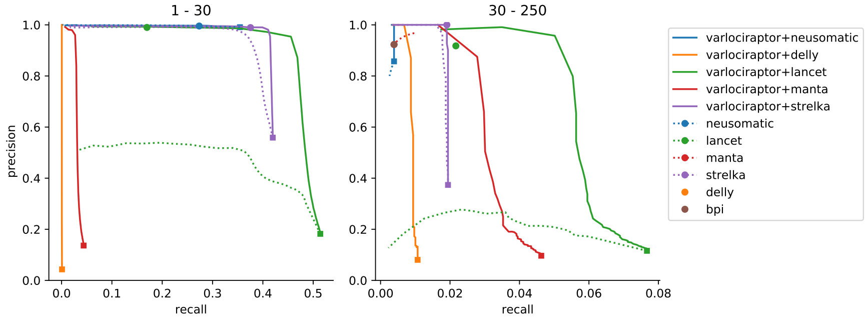

Fig. S9: Recall and precision for calling somatic insertions on synthetic data (mixture rate 20%). Results are grouped by deletion length, denoted as interval at the top of the plot. For our approach (Varlociraptor+*) curves are plotted by scanning over the posterior probability for having a somatic variant (for readability, each curve is terminated by a square mark). For other callers that provide a score to scan over (e.g. p-value for Lancet) we plot a dotted line. Ad-hoc results are shown as single dots. Results are shown if the prediction of the caller did provide at least 10 calls.

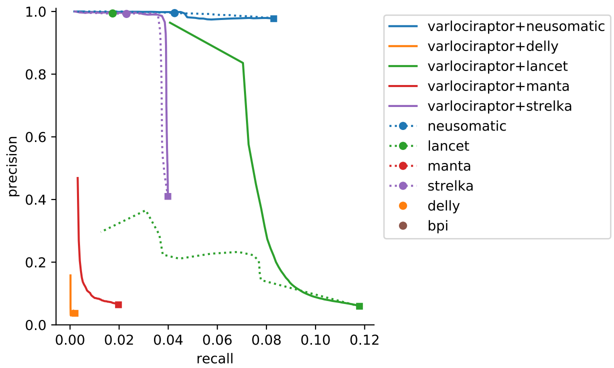

Fig. S10: Recall and precision for calling somatic deletions on synthetic data (mixture rate 5%). Results are grouped by deletion length, denoted as interval at the top of the plot. For our approach (Varlociraptor+*) curves are plotted by scanning over the posterior probability for having a somatic variant (for readability, each curve is terminated by a square mark). For other callers that provide a score to scan over (e.g. p-value for Lancet) we plot a dotted line. Ad-hoc results are shown as single dots. Results are shown if the prediction of the caller did provide at least 10 calls.

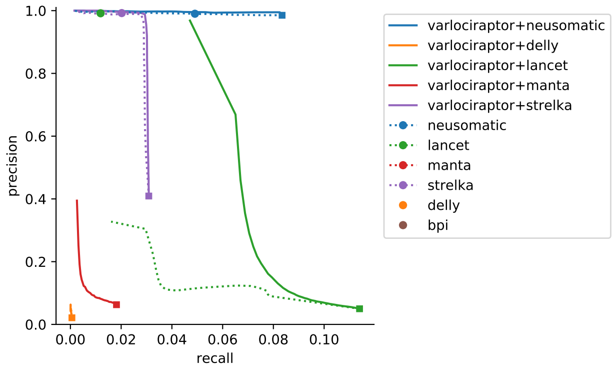

Fig. S11: Recall and precision for calling somatic insertions on synthetic data (mixture rate 5%). Results are grouped by deletion length, denoted as interval at the top of the plot. For our approach (Varlociraptor+*) curves are plotted by scanning over the posterior probability for having a somatic variant (for readability, each curve is terminated by a square mark). For other callers that provide a score to scan over (e.g. p-value for Lancet) we plot a dotted line. Ad-hoc results are shown as single dots. Results are shown if the prediction of the caller did provide at least 10 calls.

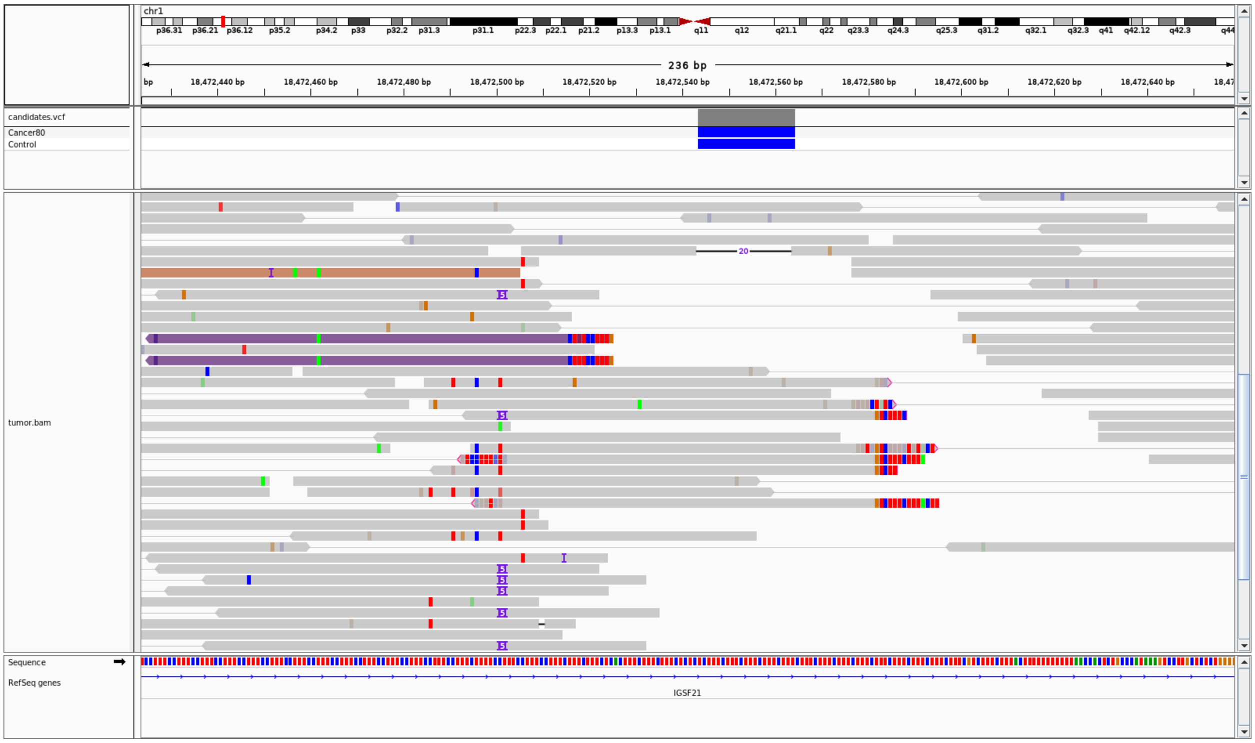

Fig. S12: Example of a variant in a repetetive region that causes misplaced softclips, highlighting the need for a realignment against the variant allele (taken from our simulated dataset (see section **??**). The clipped alignments (shown as mismatches at the read ends) should instead have a 20 bp deletion as the read at the top. Visualization was performed with IGV [3].

References

[1] F. Liu, M.J. Bayarriy, and J.O. Bergerz. Modularization in bayesian analysis, with emphasis on analysis of computer models. *Bayesian Analysis*, 4(1):119–150, 2009.

[2] Marc J Williams, Benjamin Werner, Chris P Barnes, Trevor A Graham, and Andrea Sottoriva. Identification of neutral tumor evolution across cancer types. *Nature Genetics*, 48(3):238–244, January 2016. ISSN 1061-4036. doi: 10.1038/ng.3489. URL `http://www.nature.com/doifinder/10.1038/ng.3489`.

[3] James T. Robinson, Helga Thorvaldsdttir, Aaron M. Wenger, Ahmet Zehir, and Jill P. Mesirov. Variant Review with the Integrative Genomics Viewer. *Cancer Research*, 77(21):e31–e34, November 2017. ISSN 0008-5472, 1538-7445. doi: 10.1158/0008-5472.CAN-17-0337. URL `http://cancerres.aacrjournals.org/content/77/21/e31`.
