# Supplementary material for: Varlociraptor: enhancing sensitivity and controlling false discovery rate in somatic indel discovery
Source: Genome Biol. 2020 Apr 28;21:98. doi: 10.1186/s13059-020-01993-6 (PMC7187499; doi:10.1186/s13059-020-01993-6)
Supplement: Supplementary file 2 — Additional file 2 Snakemake Report: A Snakemake report that allows to interactively explore all figures shown in this article in the context of workflow, parameters, software versions, and code. [file 13059_2020_1993_MOESM2_ESM.html]

Snakemake Report


Loading Snakemake Report...

Please enable Javascript in your browser to see this report.

Loading 6.6 MB. For large reports, this can take a while.

Snakemake Report

- Fri Jan 31 15:59:54 2020 CET
- Snakemake 5.9.1

- Workflow (current)
- Statistics
- Configuration
- Rules

##### Results

- Allele Frequency Estimation
- Concordance
- FDR Control
- Precision and Recall
- Score Distribution


## Workflow

Detailed software versions can be found under Rules.

## Results

##### Allele Frequency Estimation

Workflow resultes

| File | Size | Description | Job properties |  |
| --- | --- | --- | --- | --- |
| simulated-bwa.DEL.svg | 361.2 kB | Allele frequency estimation error vs. true allele frequency on simulated data. | Job properties  |  |  | | --- | --- | | Rule | plot\_allelefreq | | Wildcards | run=simulated-bwa, vartype=DEL | | Params | varlociraptor\_callers=['neusomatic', 'delly', 'lancet', 'manta', 'strelka'], len\_ranges=[[1, 30], [30, 50], [50, 100], [100, 250]] | |  |
| simulated-bwa.DEL.svg | 2.0 MB | Allele frequency estimation error vs. true allele frequency on simulated data. The dashed lines depict the standard deviation, solid line depicts the mean. The grey area shows the standard deviation of a corresponding binomial sampling, which marks the maximum theoretically achievable accuracy. | Job properties  |  |  | | --- | --- | | Rule | plot\_allelefreq\_scatter | | Wildcards | run=simulated-bwa, vartype=DEL | | Params | depth\_ranges=[[1, 20], [20, 40]], callers=['neusomatic', 'delly', 'lancet', 'manta', 'strelka'] | |  |
| simulated-bwa.INS.svg | 206.2 kB | Allele frequency estimation error vs. true allele frequency on simulated data. | Job properties  |  |  | | --- | --- | | Rule | plot\_allelefreq | | Wildcards | run=simulated-bwa, vartype=INS | | Params | varlociraptor\_callers=['neusomatic', 'delly', 'lancet', 'manta', 'strelka'], len\_ranges=[[1, 30], [30, 100]] | |  |
| simulated-bwa.INS.svg | 2.3 MB | Allele frequency estimation error vs. true allele frequency on simulated data. The dashed lines depict the standard deviation, solid line depicts the mean. The grey area shows the standard deviation of a corresponding binomial sampling, which marks the maximum theoretically achievable accuracy. | Job properties  |  |  | | --- | --- | | Rule | plot\_allelefreq\_scatter | | Wildcards | run=simulated-bwa, vartype=INS | | Params | depth\_ranges=[[1, 10], [10, 20], [20, 40], [40, 100]], callers=['neusomatic', 'delly', 'lancet', 'manta', 'strelka'] | |  |

##### Concordance

Workflow resultes

| File | Size | Description | Job properties |  |
| --- | --- | --- | --- | --- |
| colo1.DEL.concordance.svg | 666.8 kB | For concordance analysis, we ran varlociraptor and the other callers on four independent sequencings of the same tumor/normal pair. Called variants were matched against each other, yielding a graph. Connected components in this graph of more than one node we counted as concordant calls. Left plot shows concordance vs minimum allele frequency. Right plot shows number of calls vs minimum allele frequency. For Varlociraptor, results are shown as area between a minimum posterior probability (for somatic in tumor) of 0.9 and 0.98. For all other callers, results are shown as dotted lines (obtained with default parameters). The grey lines depict the theoretical expectation according to the model of neutral tumor evolution by Williams et al. See paper for details. | Job properties  |  |  | | --- | --- | | Rule | plot\_concordance | | Wildcards | id=colo1, vartype=DEL | | Params | callers=['neusomatic', 'delly', 'lancet', 'manta', 'strelka', 'bpi'] | |  |
| colo1.INS.concordance.svg | 500.5 kB | For concordance analysis, we ran varlociraptor and the other callers on four independent sequencings of the same tumor/normal pair. Called variants were matched against each other, yielding a graph. Connected components in this graph of more than one node we counted as concordant calls. Left plot shows concordance vs minimum allele frequency. Right plot shows number of calls vs minimum allele frequency. For Varlociraptor, results are shown as area between a minimum posterior probability (for somatic in tumor) of 0.9 and 0.98. For all other callers, results are shown as dotted lines (obtained with default parameters). The grey lines depict the theoretical expectation according to the model of neutral tumor evolution by Williams et al. See paper for details. | Job properties  |  |  | | --- | --- | | Rule | plot\_concordance | | Wildcards | id=colo1, vartype=INS | | Params | callers=['neusomatic', 'delly', 'lancet', 'manta', 'strelka', 'bpi'] | |  |

##### FDR Control

Workflow resultes

| File | Size | Description | Job properties |  |
| --- | --- | --- | --- | --- |
| simulated-bwa.DEL.svg | 81.0 kB | Performance of FDR control on simulated data. Horizontal axis shows the applied FDR threshold, vertical axis shows the true FDR of the selected set of variants. Perfect FDR control would result in a line directly on the diagonal. Conservative control would lie in the area below the diagonal. Anything above the diagonal would be overly optimistic. | Job properties  |  |  | | --- | --- | | Rule | plot\_fdr | | Wildcards | run=simulated-bwa, vartype=DEL | | Params | callers=['neusomatic', 'delly', 'lancet', 'manta', 'strelka'], purity=0.75, len\_ranges=[[1, 30], [30, 50], [50, 100], [100, 250]], fdrs=[1e-05, 0.0001, 0.001, 0.01, 0.05, 0.1, 0.2, 0.3, 0.4, 0.5, 0.6, 0.7, 0.8, 0.9] | |  |
| simulated-bwa.INS.svg | 54.8 kB | Performance of FDR control on simulated data. Horizontal axis shows the applied FDR threshold, vertical axis shows the true FDR of the selected set of variants. Perfect FDR control would result in a line directly on the diagonal. Conservative control would lie in the area below the diagonal. Anything above the diagonal would be overly optimistic. | Job properties  |  |  | | --- | --- | | Rule | plot\_fdr | | Wildcards | run=simulated-bwa, vartype=INS | | Params | callers=['neusomatic', 'delly', 'lancet', 'manta', 'strelka'], purity=0.75, len\_ranges=[[1, 30], [30, 100]], fdrs=[1e-05, 0.0001, 0.001, 0.01, 0.05, 0.1, 0.2, 0.3, 0.4, 0.5, 0.6, 0.7, 0.8, 0.9] | |  |

##### Precision and Recall

Workflow resultes

| File | Size | Description | Job properties |  |
| --- | --- | --- | --- | --- |
| simulated-bwa.DEL.nozoom.pdf | 28.1 kB | Precision and recall on simulated data. | Job properties  |  |  | | --- | --- | | Rule | plot\_precision\_recall | | Wildcards | run=simulated-bwa, vartype=DEL, zoom=nozoom | | Params | varlociraptor\_callers=['neusomatic', 'delly', 'lancet', 'manta', 'strelka'], default\_callers=['neusomatic', 'lancet', 'manta', 'strelka'], adhoc\_callers=['neusomatic', 'delly', 'lancet', 'manta', 'strelka', 'bpi'], len\_ranges=[[1, 30], [30, 50], [50, 100], [100, 250]], legend\_outside=False | |  |
| simulated-bwa.DEL.zoom.pdf | 24.6 kB | Precision and recall on simulated data. | Job properties  |  |  | | --- | --- | | Rule | plot\_precision\_recall | | Wildcards | run=simulated-bwa, vartype=DEL, zoom=zoom | | Params | varlociraptor\_callers=['neusomatic', 'delly', 'lancet', 'manta', 'strelka'], default\_callers=['neusomatic', 'lancet', 'manta', 'strelka'], adhoc\_callers=['neusomatic', 'delly', 'lancet', 'manta', 'strelka', 'bpi'], len\_ranges=[[1, 30], [30, 50], [50, 100], [100, 250]], legend\_outside=False | |  |
| simulated-bwa.INS.nozoom.pdf | 20.3 kB | Precision and recall on simulated data. | Job properties  |  |  | | --- | --- | | Rule | plot\_precision\_recall | | Wildcards | run=simulated-bwa, vartype=INS, zoom=nozoom | | Params | varlociraptor\_callers=['neusomatic', 'delly', 'lancet', 'manta', 'strelka'], default\_callers=['neusomatic', 'lancet', 'manta', 'strelka'], adhoc\_callers=['neusomatic', 'delly', 'lancet', 'manta', 'strelka', 'bpi'], len\_ranges=[[1, 30], [30, 100]], legend\_outside=False | |  |
| simulated-bwa.INS.zoom.pdf | 18.5 kB | Precision and recall on simulated data. | Job properties  |  |  | | --- | --- | | Rule | plot\_precision\_recall | | Wildcards | run=simulated-bwa, vartype=INS, zoom=zoom | | Params | varlociraptor\_callers=['neusomatic', 'delly', 'lancet', 'manta', 'strelka'], default\_callers=['neusomatic', 'lancet', 'manta', 'strelka'], adhoc\_callers=['neusomatic', 'delly', 'lancet', 'manta', 'strelka', 'bpi'], len\_ranges=[[1, 30], [30, 100]], legend\_outside=False | |  |
| synthetic-20.DEL.nozoom.pdf | 23.5 kB | Precision and recall on simulated data. | Job properties  |  |  | | --- | --- | | Rule | plot\_precision\_recall | | Wildcards | run=synthetic-20, vartype=DEL, zoom=nozoom | | Params | varlociraptor\_callers=['neusomatic', 'delly', 'lancet', 'manta', 'strelka'], default\_callers=['neusomatic', 'lancet', 'manta', 'strelka'], adhoc\_callers=['neusomatic', 'delly', 'lancet', 'manta', 'strelka', 'bpi'], len\_ranges=[[1, 30], [30, 250]], legend\_outside=True | |  |
| synthetic-20.DEL.zoom.pdf | 19.7 kB | Precision and recall on simulated data. | Job properties  |  |  | | --- | --- | | Rule | plot\_precision\_recall | | Wildcards | run=synthetic-20, vartype=DEL, zoom=zoom | | Params | varlociraptor\_callers=['neusomatic', 'delly', 'lancet', 'manta', 'strelka'], default\_callers=['neusomatic', 'lancet', 'manta', 'strelka'], adhoc\_callers=['neusomatic', 'delly', 'lancet', 'manta', 'strelka', 'bpi'], len\_ranges=[[1, 30], [30, 250]], legend\_outside=True | |  |
| synthetic-20.INS.nozoom.pdf | 22.0 kB | Precision and recall on simulated data. | Job properties  |  |  | | --- | --- | | Rule | plot\_precision\_recall | | Wildcards | run=synthetic-20, vartype=INS, zoom=nozoom | | Params | varlociraptor\_callers=['neusomatic', 'delly', 'lancet', 'manta', 'strelka'], default\_callers=['neusomatic', 'lancet', 'manta', 'strelka'], adhoc\_callers=['neusomatic', 'delly', 'lancet', 'manta', 'strelka', 'bpi'], len\_ranges=[[1, 30], [30, 250]], legend\_outside=True | |  |
| synthetic-20.INS.zoom.pdf | 18.3 kB | Precision and recall on simulated data. | Job properties  |  |  | | --- | --- | | Rule | plot\_precision\_recall | | Wildcards | run=synthetic-20, vartype=INS, zoom=zoom | | Params | varlociraptor\_callers=['neusomatic', 'delly', 'lancet', 'manta', 'strelka'], default\_callers=['neusomatic', 'lancet', 'manta', 'strelka'], adhoc\_callers=['neusomatic', 'delly', 'lancet', 'manta', 'strelka', 'bpi'], len\_ranges=[[1, 30], [30, 250]], legend\_outside=True | |  |
| synthetic-5.DEL.nozoom.pdf | 17.0 kB | Precision and recall on simulated data. | Job properties  |  |  | | --- | --- | | Rule | plot\_precision\_recall | | Wildcards | run=synthetic-5, vartype=DEL, zoom=nozoom | | Params | varlociraptor\_callers=['neusomatic', 'delly', 'lancet', 'manta', 'strelka'], default\_callers=['neusomatic', 'lancet', 'manta', 'strelka'], adhoc\_callers=['neusomatic', 'delly', 'lancet', 'manta', 'strelka', 'bpi'], len\_ranges=[[1, 250]], legend\_outside=True | |  |
| synthetic-5.DEL.zoom.pdf | 16.0 kB | Precision and recall on simulated data. | Job properties  |  |  | | --- | --- | | Rule | plot\_precision\_recall | | Wildcards | run=synthetic-5, vartype=DEL, zoom=zoom | | Params | varlociraptor\_callers=['neusomatic', 'delly', 'lancet', 'manta', 'strelka'], default\_callers=['neusomatic', 'lancet', 'manta', 'strelka'], adhoc\_callers=['neusomatic', 'delly', 'lancet', 'manta', 'strelka', 'bpi'], len\_ranges=[[1, 250]], legend\_outside=True | |  |
| synthetic-5.INS.nozoom.pdf | 17.0 kB | Precision and recall on simulated data. | Job properties  |  |  | | --- | --- | | Rule | plot\_precision\_recall | | Wildcards | run=synthetic-5, vartype=INS, zoom=nozoom | | Params | varlociraptor\_callers=['neusomatic', 'delly', 'lancet', 'manta', 'strelka'], default\_callers=['neusomatic', 'lancet', 'manta', 'strelka'], adhoc\_callers=['neusomatic', 'delly', 'lancet', 'manta', 'strelka', 'bpi'], len\_ranges=[[1, 250]], legend\_outside=True | |  |
| synthetic-5.INS.zoom.pdf | 15.3 kB | Precision and recall on simulated data. | Job properties  |  |  | | --- | --- | | Rule | plot\_precision\_recall | | Wildcards | run=synthetic-5, vartype=INS, zoom=zoom | | Params | varlociraptor\_callers=['neusomatic', 'delly', 'lancet', 'manta', 'strelka'], default\_callers=['neusomatic', 'lancet', 'manta', 'strelka'], adhoc\_callers=['neusomatic', 'delly', 'lancet', 'manta', 'strelka', 'bpi'], len\_ranges=[[1, 250]], legend\_outside=True | |  |

##### Score Distribution

Workflow resultes

| File | Size | Description | Job properties |  |
| --- | --- | --- | --- | --- |
| simulated-bwa.DEL.svg | 118.1 kB | Distribution of true and false positives depending on posterior probability for somatic variant in the tumor as given by varlociraptor. Desirable is a good separation between the two distributions. | Job properties  |  |  | | --- | --- | | Rule | plot\_score\_dist | | Wildcards | run=simulated-bwa, vartype=DEL | | Params | varlociraptor\_callers=['neusomatic', 'delly', 'lancet', 'manta', 'strelka'], len\_ranges=[[1, 30], [30, 50], [50, 100], [100, 250]] | |  |
| simulated-bwa.INS.svg | 82.0 kB | Distribution of true and false positives depending on posterior probability for somatic variant in the tumor as given by varlociraptor. Desirable is a good separation between the two distributions. | Job properties  |  |  | | --- | --- | | Rule | plot\_score\_dist | | Wildcards | run=simulated-bwa, vartype=INS | | Params | varlociraptor\_callers=['neusomatic', 'delly', 'lancet', 'manta', 'strelka'], len\_ranges=[[1, 30], [30, 100]] | |  |

## Statistics

If the workflow has been executed in cluster/cloud, runtimes include the waiting time in the queue.

## Configuration

Configuration files

| File | Code |
| --- | --- |
| config.yaml | |  |  | | --- | --- | | ```   1   2   3   4   5   6   7   8   9  10  11  12  13  14  15  16  17  18  19  20  21  22  23  24  25  26  27  28  29  30  31  32  33  34  35  36  37  38  39  40  41  42  43  44  45  46  47  48  49  50  51  52  53  54  55  56  57  58  59  60  61  62  63  64  65  66  67  68  69  70  71  72  73  74  75  76  77  78  79  80  81  82  83  84  85  86  87  88  89  90  91  92  93  94  95  96  97  98  99 100 101 102 103 104 105 106 107 108 109 110 111 112 113 114 115 116 117 118 119 120 121 122 123 124 125 126 127 128 129 130 131 132 133 134 135 136 137 138 139 140 141 142 143 144 145 146 147 148 149 150 151 152 153 154 155 156 157 158 159 160 161 162 163 164 165 166 167 168 169 170 171 172 173 174 175 176 177 178 179 180 181 182 183 184 185 186 187 188 189 190 191 192 193 194 195 196 197 198 199 200 201 202 203 204 205 206 207 208 209 210 211 212 213 214 215 216 217 218 219 220 221 222 223 224 225 226 227 228 229 230 231 232 233 234 235 236 237 238 239 240 241 242 243 ``` | ``` gdc-manifest: gdc_manifest_20171012_060907.txt  runs:   synthetic-5:     mapper: bwa     dataset: synthetic-5     ref: hg38     purity: 1.0     legend-outside: true   synthetic-20:     mapper: bwa     dataset: synthetic-20     ref: hg38     purity: 1.0     legend-outside: true   simulated-bwa:     mapper: bwa     dataset: simulated     ref: hg18     purity: 0.75   COLO_829-GSC:     dataset: COLO_829-GSC     mapper: bwa     ref: hg38     purity: 1.0   COLO_829-EBI:     dataset: COLO_829-EBI     mapper: bwa     ref: hg38     purity: 1.0   COLO_829-TGen:     dataset: COLO_829-TGen     mapper: bwa     ref: hg38     purity: 1.0   COLO_829-Ill:     dataset: COLO_829-Ill     mapper: bwa     ref: hg38     purity: 1.0   datasets:   synthetic-5:     tumor:       fq1: /vol/tiny/prosic/chm-synthetic-tumor-normal/dataset/mix5/tumor_1.fastq       fq2: /vol/tiny/prosic/chm-synthetic-tumor-normal/dataset/mix5/tumor_2.fastq       name: Cancer     normal:       fq1: /vol/tiny/prosic/chm-synthetic-tumor-normal/dataset/mix5/normal_1.fastq       fq2: /vol/tiny/prosic/chm-synthetic-tumor-normal/dataset/mix5/normal_2.fastq       name: Control     truth: /vol/tiny/prosic/chm-synthetic-tumor-normal/dataset/mix5/truth.nochr.vcf     regions: /vol/tiny/prosic/chm-synthetic-tumor-normal/dataset/mix5/confident-regions.nochr.bed   synthetic-20:     tumor:       fq1: /vol/tiny/prosic/chm-synthetic-tumor-normal/dataset/mix20/tumor_1.fastq       fq2: /vol/tiny/prosic/chm-synthetic-tumor-normal/dataset/mix20/tumor_2.fastq       name: Cancer     normal:       fq1: /vol/tiny/prosic/chm-synthetic-tumor-normal/dataset/mix20/normal_1.fastq       fq2: /vol/tiny/prosic/chm-synthetic-tumor-normal/dataset/mix20/normal_2.fastq       name: Control     truth: /vol/tiny/prosic/chm-synthetic-tumor-normal/dataset/mix20/truth.nochr.vcf     regions: /vol/tiny/prosic/chm-synthetic-tumor-normal/dataset/mix20/confident-regions.nochr.bed   simulated:     tumor:       bam: ../data/hiseq.Cancer80.wholegenome.bwamemm.cat.sorted.smtag.bam       name: Cancer80     normal:       bam: ../data/hiseq.Control.wholegenome.cov30.bwamemm.sorted.smtag.bam       name: Control     isize:       mean: 312       sd: 15     truth: ../data/simulated-truth.indels.vcf   COLO_829-GSC:     tumor:       bam: ../data/EGAD00001002142/EGAD00001002142/EGAZ00001226259_COLO_829_BCGSC_BCGSCPipe.bam       name: Cancer     normal:       bam: ../data/EGAD00001002142/EGAD00001002142/EGAZ00001226245_COLO_829BL_BCGSC_BCGSCPipe.bam       name: Control     case: COLO_829   COLO_829-EBI:     tumor:       bam: ../data/EGAD00001002142/EGAD00001002142/EGAZ00001226269_COLO_829_EPleasance_BCGSCPipe.bam       name: Cancer     normal:       bam: ../data/EGAD00001002142/EGAD00001002142/EGAZ00001226254_COLO_829BL_EPleasance_BCGSCPipe.bam       name: Control     case: COLO_829   COLO_829-Ill:     tumor:       bam: ../data/EGAD00001002142/EGAD00001002142/EGAZ00001226270_COLO_829_Illumina_BCGSCPipe.bam       name: Cancer     normal:       bam: ../data/EGAD00001002142/EGAD00001002142/EGAZ00001226256_COLO_829BL_Illumina_BCGSCPipe.bam       name: Control     case: COLO_829   COLO_829-TGen:     tumor:       bam: ../data/EGAD00001002142/EGAD00001002142/EGAZ00001229972_COLO_829_TGEN_BCGSCPipe.bam       name: Cancer     normal:       bam: ../data/EGAD00001002142/EGAD00001002142/EGAZ00001226249_COLO_829BL_TGEN_BCGSCPipe.bam       name: Control     case: COLO_829  ref:   hg18:     fasta: data/hg18.fasta     date: 2006-03     chrom_prefix: chr   hg38:     fasta: data/Homo_sapiens.GRCh38.dna.primary_assembly.fa     date: 2013-12     chrom_prefix: ""     exons: resources/hg38.exons.bed  caller:   neusomatic:     params: ""     score: SCORE     invert: true     adhoc: true     genotypes: false     info:       - SCORE   delly:     params: ""     adhoc: true     genotypes: true     info:       - END       - SOMATIC       - SVLEN       - SVTYPE   #pindel:   #  params: "-M 1 -B 10000 -H 10 -x 4 -I false -a 2"   lancet:     params: ""     score: QUAL     invert: true     adhoc: true     genotypes: true     info:       - SOMATIC       - SVLEN       - SVTYPE   manta:     params: ""     score: SOMATICSCORE     invert: true     adhoc: true     useraw: true     info:       - SOMATIC       - SOMATICSCORE       - SVLEN       - SVTYPE   strelka:     params: ""     score: QSI     invert: true     adhoc: true     info:       - SOMATIC       - QSI   bpi:     params: ""     adhoc: true     info:       - SOMATIC       - SVLEN       - SVTYPE   varlociraptor:     params: "--indel-window 64 --omit-snvs"     blacklist:       - bpi     score: PROB_SOMATIC_TUMOR     genotypes: false     info:       - PROB_SOMATIC_TUMOR       - PROB_GERMLINE_HET       - PROB_GERMLINE_HOM       - PROB_ABSENT       - PROB_SOMATIC_NORMAL       - PROB_ARTIFACT       - SVLEN     fmt:       - AF       - DP  vcf-match-params: "--max-dist 50 --max-len-diff 50"  len-ranges:   synthetic-20:     DEL:       - [1, 30]       - [30, 250]     INS:       - [1, 30]       - [30, 250]   synthetic-5:     DEL:       - [1, 250]     INS:       - [1, 250]   DEL:     - [1, 30]     - [30, 50]     - [50, 100]     - [100, 250]   INS:     - [1, 30]     - [30, 100]   depth-ranges:   DEL:     - [1, 20]     - [20, 40]   INS:     - [1, 10]     - [10, 20]     - [20, 40]     - [40, 100]   plots:   concordance: # runs where we can do concordance analysis     colo1:       - COLO_829-GSC       - COLO_829-Ill       - COLO_829-TGen       - COLO_829-EBI   known-truth: # runs with a known truth     - simulated-bwa     - synthetic-5     - synthetic-20   known-truth-full-afs: # runs where variants exhibit the full allele frequency spectrum     - simulated-bwa ``` | |

## Rules

Workflow rules

| Rule | Jobs | Output | Singularity | Conda environment | Code |
| --- | --- | --- | --- | --- | --- |
| svg2pdf | 12 | - plots/fdr-control/simulated-bwa.INS.pdf - plots/fdr-control/simulated-bwa.DEL.pdf - plots/allelefreqs/simulated-bwa.INS.pdf - plots/allelefreqs/simulated-bwa.DEL.pdf - plots/score-dist/simulated-bwa.INS.pdf - plots/score-dist/simulated-bwa.DEL.pdf - plots/allelefreq-recall/simulated-bwa.INS.pdf - plots/allelefreq-recall/simulated-bwa.DEL.pdf - plots/allelefreq-scatter/simulated-bwa.INS.pdf - plots/allelefreq-scatter/simulated-bwa.DEL.pdf - plots/concordance/colo1.INS.concordance.pdf - plots/concordance/colo1.DEL.concordance.pdf |  | - cairosvg =2.1.3 | |  |  | | --- | --- | | ``` 1 ``` | ``` cairosvg {input} -o {output} ``` | |
| plot\_precision\_recall | 12 | - plots/precision-recall/simulated-bwa.INS.zoom.pdf - plots/precision-recall/simulated-bwa.INS.nozoom.pdf - plots/precision-recall/synthetic-5.INS.zoom.pdf - plots/precision-recall/synthetic-5.INS.nozoom.pdf - plots/precision-recall/synthetic-20.INS.zoom.pdf - plots/precision-recall/synthetic-20.INS.nozoom.pdf - plots/precision-recall/simulated-bwa.DEL.zoom.pdf - plots/precision-recall/simulated-bwa.DEL.nozoom.pdf - plots/precision-recall/synthetic-5.DEL.zoom.pdf - plots/precision-recall/synthetic-5.DEL.nozoom.pdf - plots/precision-recall/synthetic-20.DEL.zoom.pdf - plots/precision-recall/synthetic-20.DEL.nozoom.pdf |  | - python =3.6 - pandas =0.23 - matplotlib =3.0 - seaborn =0.9.0 - pysam =0.13.0 - svgutils =0.2 - pybedtools =0.7.10 - networkx =2.2 | |  |  | | --- | --- | | ```   1   2   3   4   5   6   7   8   9  10  11  12  13  14  15  16  17  18  19  20  21  22  23  24  25  26  27  28  29  30  31  32  33  34  35  36  37  38  39  40  41  42  43  44  45  46  47  48  49  50  51  52  53  54  55  56  57  58  59  60  61  62  63  64  65  66  67  68  69  70  71  72  73  74  75  76  77  78  79  80  81  82  83  84  85  86  87  88  89  90  91  92  93  94  95  96  97  98  99 100 101 102 103 104 105 106 107 108 109 110 111 112 113 114 115 116 117 118 119 120 121 122 123 124 125 126 127 128 129 130 131 132 133 134 ``` | ``` from itertools import product from functools import partial import matplotlib matplotlib.use("agg") from matplotlib import pyplot as plt import seaborn as sns import pandas as pd import common import numpy as np import math from matplotlib.lines import Line2D  vartype = snakemake.wildcards.vartype colors = common.get_colors(snakemake.config)   def props(callers):     return product(callers, snakemake.params.len_ranges)   def plot_len_range(minlen, maxlen, min_precision=0.0):      truth = common.load_variants(         snakemake.input.truth, minlen, maxlen, vartype=vartype)      def plot(calls,              label,              color,              line=True,              style="-",              invert=False,              markersize=4,              endmarker=False):         calls = pd.read_table(calls, index_col=0)         if len(calls) < 10:             return         if line:             thresholds = calls.score.quantile(np.linspace(0.0, 1.0, 50))             precision = []             recall = []             for t in thresholds:                 if invert:                     c = calls[calls.score >= t]                 else:                     c = calls[calls.score <= t]                 p = common.precision(c)                 r = common.recall(c, truth)                 print(label, t, c.shape[0], p, r)                 if len(c) < 10:                     print("skipping threshold: too few calls", c)                     continue                 precision.append(p)                 recall.append(r)             if len(precision) <= 2:                 print("skipping curve because we have too few values")                 return         else:             precision = [common.precision(calls)]             recall = [common.recall(calls, truth)]             style = "."             print(label, calls.shape[0], precision, recall)          plt.plot(             recall,             precision,             style,             color=color,             label=label,             markersize=markersize         )         if endmarker:             plt.plot(recall[-1], precision[-1], "s", color=color, markersize=markersize)      handles = []     for calls, (caller,                 len_range) in zip(snakemake.input.varlociraptor_calls,                                   props(snakemake.params.varlociraptor_callers)):         if len_range[0] != minlen and len_range[1] != maxlen:             continue         label = "varlociraptor+{}".format(caller)         plot(calls, label, colors[caller], endmarker=True)         handles.append(Line2D([0], [0], color=colors[caller], label=label))      for calls, (caller,                 len_range) in zip(snakemake.input.default_calls,                                   props(snakemake.params.default_callers)):         if len_range[0] != minlen and len_range[1] != maxlen:             continue         color = colors[caller]         plot(             calls,             caller,             color,             style=":",             invert=snakemake.config["caller"][caller].get("invert", False))         if caller in snakemake.params.adhoc_callers:             handles.append(Line2D([0], [0], markersize=10, markerfacecolor=color, markeredgecolor=color, color=color, label=caller, marker=".", linestyle=":"))         else:             handles.append(Line2D([0], [0], color=color, label=caller, linestyle=":"))      for calls, (caller, len_range) in zip(snakemake.input.adhoc_calls,                              props(snakemake.params.adhoc_callers)):         if len_range[0] != minlen and len_range[1] != maxlen:             continue         color = colors[caller]         plot(calls, caller, color, markersize=10, line=False)         if caller not in snakemake.params.default_callers:             handles.append(Line2D([0], [0], markersize=10, markerfacecolor=color, markeredgecolor=color, label=caller, marker=".", lw=0))      sns.despine()     ax = plt.gca()     plt.ylim((min_precision, 1.01 if min_precision == 0.0 else 1.001))     return ax, handles   plot = plot_len_range fig_height = None legend_outside = snakemake.params.legend_outside if snakemake.wildcards.zoom == "zoom":     plot = partial(plot_len_range, min_precision=0.99 if vartype == "INS" else 0.95)     fig_height = 3     legend_outside = True   common.plot_ranges(     snakemake.params.len_ranges,     plot,     xlabel="recall",     ylabel="precision",     fig_height=fig_height,     legend_outside=legend_outside, )  plt.savefig(snakemake.output[0], bbox_inches="tight") ``` | |
| plot\_fdr | 2 | - plots/fdr-control/simulated-bwa.INS.svg - plots/fdr-control/simulated-bwa.DEL.svg |  | - python =3.6 - pandas =0.23 - matplotlib =3.0 - seaborn =0.9.0 - pysam =0.13.0 - svgutils =0.2 - pybedtools =0.7.10 - networkx =2.2 | |  |  | | --- | --- | | ```  1  2  3  4  5  6  7  8  9 10 11 12 13 14 15 16 17 18 19 20 21 22 23 24 25 26 27 28 29 30 31 32 33 34 35 36 37 38 39 40 41 42 43 44 45 46 47 48 49 50 51 52 53 54 55 56 57 58 59 60 61 62 63 64 65 66 ``` | ``` from itertools import product import matplotlib matplotlib.use("agg") from matplotlib import pyplot as plt import seaborn as sns import pandas as pd import common import numpy as np   MIN_CALLS = 100  colors = common.get_colors(snakemake.config)  props = product(snakemake.params.callers,                 snakemake.params.len_ranges, snakemake.params.fdrs)  calls = []  for _calls, (caller, len_range, fdr) in zip(snakemake.input.varlociraptor_calls, props):     calls.append({"caller": caller, "len_range": len_range, "fdr": float(fdr), "calls": _calls})  calls = pd.DataFrame(calls) calls = calls.set_index("caller", drop=False)   def plot_len_range(minlen, maxlen):      def plot(caller):         color = colors[caller]         label = "varlociraptor+{}".format(caller)         fdrs = []         alphas = []         calls_ = calls.loc[caller]         calls_ = calls_[calls_["len_range"].map(lambda r: r == [minlen, maxlen])]         calls_ = calls_.sort_values("fdr")         for e in calls_.itertuples():             c = pd.read_table(e.calls)             n = c.shape[0]             if n < MIN_CALLS:                 continue             true_fdr = 1.0 - common.precision(c)             if fdrs and fdrs[-1] == true_fdr:                 continue             fdrs.append(true_fdr)             alphas.append(e.fdr)         plt.plot(alphas, fdrs, ".-", color=color, label=label)       for caller in calls.index.unique():         plot(caller)      plt.plot([0, 1], [0, 1], ":", color="grey")      sns.despine()     ax = plt.gca()     handles, _ = ax.get_legend_handles_labels()     return ax, handles  common.plot_ranges(     snakemake.params.len_ranges,     plot_len_range,     xlabel="FDR threshold",     ylabel="true FDR")  plt.savefig(snakemake.output[0], bbox_inches="tight") ``` | |
| plot\_allelefreq | 2 | - plots/allelefreqs/simulated-bwa.INS.svg - plots/allelefreqs/simulated-bwa.DEL.svg |  | - python =3.6 - pandas =0.23 - matplotlib =3.0 - seaborn =0.9.0 - pysam =0.13.0 - svgutils =0.2 - pybedtools =0.7.10 - networkx =2.2 | |  |  | | --- | --- | | ```  1  2  3  4  5  6  7  8  9 10 11 12 13 14 15 16 17 18 19 20 21 22 23 24 25 26 27 28 29 30 31 32 33 34 35 36 37 38 39 40 41 42 43 44 45 46 47 48 49 50 51 52 53 54 55 56 57 58 59 60 61 62 63 64 65 66 67 68 69 70 71 72 73 74 75 76 77 78 79 80 81 82 83 84 85 86 ``` | ``` from itertools import product import math import matplotlib matplotlib.use("agg") from matplotlib import pyplot as plt import seaborn as sns import pandas as pd import common import numpy as np   MIN_CALLS = 10  vartype = snakemake.wildcards.vartype colors = common.get_colors(snakemake.config)  truth = common.load_variants(snakemake.input.truth, vartype=vartype)  def props(callers):     return product(callers, snakemake.params.len_ranges)  def plot_len_range(minlen, maxlen):     def plot(calls, colors):         calls = calls[calls.is_tp]         true_af = truth.loc[calls.MATCHING].reset_index().TAF         calls = calls.reset_index()         calls["error"] = calls.CASE_AF - true_af          if calls.empty:             return          calls["true_af"] = true_af         true_af = pd.Series(calls["true_af"].unique()).sort_values()         # standard deviation when sampling in binomial process from allele freq         # this is the expected sampling error within the correctly mapped fragments         # sd = true_af.apply(lambda af: 1 / 40 * math.sqrt(40 * af * (1 - af)))         # x = np.arange(len(true_af))         # offsets = [-0.5, 0.5]         # y_upper = np.array([v for v in sd for o in offsets])         #  y_lower = np.maximum(-y_upper, [-f for f in true_af for o in offsets])         # plt.fill_between([v + o for v in x for o in offsets], y_lower, y_upper, color="#EEEEEE", zorder=-5)          calls["true_af"] = calls["true_af"].apply("{:.3f}".format)          size = 1 if maxlen == 30 else 2         sns.stripplot("true_af", "error", hue="caller", data=calls, palette=colors, dodge=True, jitter=True, alpha=0.5, size=size, rasterized=True)         sns.boxplot("true_af", "error", hue="caller", data=calls, color="white", fliersize=0, linewidth=1)          handles, labels = plt.gca().get_legend_handles_labels()         n = len(calls.caller.unique())          plt.ylim((-1,1))         plt.grid(axis="y", linestyle=":", color="grey")         sns.despine()         plt.xticks(rotation="vertical")         ax = plt.gca()         ax.legend().remove()          return ax, handles[n:]      all_calls, all_colors = load_calls(minlen, maxlen)     return plot(all_calls, all_colors)  def load_calls(minlen, maxlen):     all_calls = []     all_colors = []     for calls, (caller, len_range) in zip(snakemake.input.varlociraptor_calls, props(snakemake.params.varlociraptor_callers)):         if len_range[0] != minlen and len_range[1] != maxlen:             continue         label = "varlociraptor+{}".format(caller)         calls = pd.read_table(calls)         calls["caller"] = label         if not calls.empty:             all_calls.append(calls)             all_colors.append(colors[caller])      all_calls = pd.concat(all_calls)     return all_calls, all_colors  common.plot_ranges(     snakemake.params.len_ranges,     plot_len_range,     xlabel="true allele frequency",     ylabel="predicted - truth")  plt.savefig(snakemake.output[0], bbox_inches="tight") ``` | |
| plot\_score\_dist | 2 | - plots/score-dist/simulated-bwa.INS.svg - plots/score-dist/simulated-bwa.DEL.svg |  | - python =3.6 - pandas =0.23 - matplotlib =3.0 - seaborn =0.9.0 - pysam =0.13.0 - svgutils =0.2 - pybedtools =0.7.10 - networkx =2.2 | |  |  | | --- | --- | | ```  1  2  3  4  5  6  7  8  9 10 11 12 13 14 15 16 17 18 19 20 21 22 23 24 25 26 27 28 29 30 31 32 33 34 35 36 37 38 39 40 41 42 43 44 45 46 47 ``` | ``` from itertools import product import matplotlib matplotlib.use("agg") from matplotlib import pyplot as plt import seaborn as sns import pandas as pd import common import numpy as np import math  vartype = snakemake.wildcards.vartype colors = common.get_colors(snakemake.config)  def props(callers):     return product(callers, snakemake.params.len_ranges)  phred_to_log_factor = -0.23025850929940456 log_to_phred_factor = -4.3429448190325175  def plot_len_range(minlen, maxlen):     for calls, (caller, len_range) in zip(snakemake.input.varlociraptor_calls, props(snakemake.params.varlociraptor_callers)):         if len_range[0] != minlen and len_range[1] != maxlen:             continue         label = "varlociraptor+{}".format(caller)         calls = pd.read_table(calls)         calls["caller"] = label         if not calls.empty:             color = colors[caller]             sns.kdeplot(calls[calls.is_tp].PROB_SOMATIC_TUMOR.map(np.log), color=color, label=label)             sns.kdeplot(calls[~calls.is_tp].PROB_SOMATIC_TUMOR.map(np.log), color=color, linestyle=":", label="")      ax = plt.gca()     fmt_ticks = lambda ticks: ["{:.1g}".format(np.exp(t)) for t in ticks]     ax.set_xticklabels(fmt_ticks(plt.xticks()[0]))     ax.legend().remove()     handles, _ = ax.get_legend_handles_labels()     sns.despine()      return ax, handles  common.plot_ranges(     snakemake.params.len_ranges,     plot_len_range,     xlabel=r"$-10 \log_{10}$ Pr(somatic)",     ylabel="density")  plt.savefig(snakemake.output[0], bbox_inches="tight") ``` | |
| plot\_allelefreq\_recall | 2 | - plots/allelefreq-recall/simulated-bwa.INS.svg - plots/allelefreq-recall/simulated-bwa.DEL.svg |  | - python =3.6 - pandas =0.23 - matplotlib =3.0 - seaborn =0.9.0 - pysam =0.13.0 - svgutils =0.2 - pybedtools =0.7.10 - networkx =2.2 | |  |  | | --- | --- | | ```  1  2  3  4  5  6  7  8  9 10 11 12 13 14 15 16 17 18 19 20 21 22 23 24 25 26 27 28 29 30 31 32 33 34 35 36 37 38 39 40 41 42 43 44 45 46 47 48 49 50 51 52 53 54 55 56 57 58 59 60 61 62 63 64 65 66 67 68 69 70 71 72 73 74 75 76 77 78 79 80 81 82 83 84 85 86 87 88 89 90 91 92 93 94 95 96 ``` | ``` from itertools import product import matplotlib matplotlib.use("agg") from matplotlib import pyplot as plt import seaborn as sns import pandas as pd import common import numpy as np import math from matplotlib.lines import Line2D  MIN_CALLS = 10  vartype = snakemake.wildcards.vartype colors = common.get_colors(snakemake.config)   def props(callers):     return product(callers, snakemake.params.len_ranges)   def plot_len_range(minlen, maxlen):      truth = common.load_variants(         snakemake.input.truth, minlen, maxlen, vartype=vartype)      afs = pd.Series(truth.TAF.unique()).sort_values()      def plot(calls,              label,              color,              varlociraptor=True,              style="-.",              markersize=4):         calls = pd.read_table(calls, index_col=0)         if len(calls) < 10:             return         if varlociraptor:             phred = lambda p: -10 * math.log10(p)             def calc_recall(p):                 c = calls[calls.score <= phred(p)]                 return [common.recall(c, truth[truth.TAF >= af]) for af in afs]              return plt.fill_between(                 afs,                 calc_recall(0.98 if maxlen > 30 else 0.99),                 calc_recall(0.9),                 color=color,                 label=label,                 alpha=0.6)         else:             recall = [common.recall(calls, truth[truth.TAF >= af]) for af in afs]             # plot a white background first to increase visibility             plt.plot(afs, recall, "-", color="white", alpha=0.8)             return plt.plot(                 afs,                 recall,                 style,                 color=color,                 label=label)[0]      handles = []     def register_handle(handle):         if handle is not None:             handles.append(handle)     for calls, (caller,                 len_range) in zip(snakemake.input.varlociraptor_calls,                                   props(snakemake.params.varlociraptor_callers)):         if len_range[0] != minlen and len_range[1] != maxlen:             continue         label = "varlociraptor+{}".format(caller)         handle = plot(calls, label, colors[caller], varlociraptor=True)         register_handle(handle)         #handles.append(Line2D([0], [0], color=colors[caller], label=label))      for calls, (caller, len_range) in zip(snakemake.input.adhoc_calls,                              props(snakemake.params.adhoc_callers)):         if len_range[0] != minlen and len_range[1] != maxlen:             continue         color = colors[caller]         handle = plot(calls, caller, color, style=":", varlociraptor=False)         register_handle(handle)         #handles.append(Line2D([0], [0], linestyle=":", color=color, label=caller))      sns.despine()     ax = plt.gca()     return ax, handles   common.plot_ranges(     snakemake.params.len_ranges,     plot_len_range,     xlabel="allele frequency",     ylabel="recall")  plt.savefig(snakemake.output[0], bbox_inches="tight") ``` | |
| plot\_allelefreq\_scatter | 2 | - plots/allelefreq-scatter/simulated-bwa.INS.svg - plots/allelefreq-scatter/simulated-bwa.DEL.svg |  | - python =3.6 - pandas =0.23 - matplotlib =3.0 - seaborn =0.9.0 - pysam =0.13.0 - svgutils =0.2 - pybedtools =0.7.10 - networkx =2.2 | |  |  | | --- | --- | | ```  1  2  3  4  5  6  7  8  9 10 11 12 13 14 15 16 17 18 19 20 21 22 23 24 25 26 27 28 29 30 31 32 33 34 35 36 37 38 39 40 41 42 43 44 45 46 47 48 49 50 51 52 53 54 55 56 57 58 59 60 61 62 63 64 65 66 67 68 69 70 71 ``` | ``` import math import matplotlib matplotlib.use("agg") from matplotlib import pyplot as plt import seaborn as sns import pandas as pd import common import numpy as np  MIN_COUNT = 20 MAX_DEPTH = 60  vartype = snakemake.wildcards.vartype colors = common.get_colors(snakemake.config)  truth = common.load_variants(snakemake.input.truth, vartype=vartype)  all_calls = [] for caller, calls in zip(snakemake.params.callers, snakemake.input.calls):     calls = pd.read_table(calls)     calls.loc[:, "caller"] = caller     all_calls.append(calls) all_calls = pd.concat(all_calls)  def plot(af, _):     constrain_lower = lambda error: np.maximum(error, -af)     constrain_upper = lambda error: np.minimum(error, 1.0 - af)      dp = all_calls["TUMOR_DP"]     calls = all_calls[all_calls.is_tp]     true_af = truth.loc[calls.MATCHING].reset_index().TAF     calls = calls.reset_index()     calls["true_af"] = true_af     calls = calls[calls["true_af"] == af]     calls["error"] = calls.CASE_AF - true_af      sns.kdeplot(calls["TUMOR_DP"], calls["error"], cmap="Blues", n_levels=50, shade=True, alpha=0.7, shade_lowest=False) #alpha=0.5, clip=((0.0, 1.0), (0.0, af)))     plt.plot(calls["TUMOR_DP"], calls["error"], ",", color="k", lw=0, alpha=1.0, rasterized=True)     by_depth = calls.groupby("TUMOR_DP")["error"].describe().reset_index()     by_depth["-std"] = constrain_lower(-by_depth["std"])     by_depth["std"] = constrain_upper(by_depth["std"])     by_depth = by_depth[by_depth["count"] >= MIN_COUNT]     plt.plot(by_depth.TUMOR_DP, by_depth["std"], "--", color="k")     plt.plot(by_depth.TUMOR_DP, by_depth["-std"], "--", color="k")     plt.plot(by_depth.TUMOR_DP, by_depth["mean"], "-", color="k")      depths = np.arange(0, MAX_DEPTH)     # standard deviation when sampling in binomial process from allele freq     # this is the expected sampling error within the correctly mapped fragments     sd = np.array([1.0 / depth * math.sqrt(depth * af * (1.0 - af)) for depth in depths])     plt.fill_between(depths, constrain_lower(-sd), constrain_upper(sd), color="grey", alpha=0.5)          sns.despine()     plt.xticks(rotation="vertical")     ax = plt.gca()     ax.legend().remove()     handles, labels = ax.get_legend_handles_labels()     plt.ylim((-1.0, 1.0))     plt.xlim((0, MAX_DEPTH))      return ax, []  afs = [(af, af) for af in truth.TAF.sort_values().unique()]  common.plot_ranges(     afs,     plot,     "depth",     "predicted - truth")  plt.savefig(snakemake.output[0], bbox_inches="tight") ``` | |
| obtain\_tp\_fp | 610 | - annotated-calls/varlociraptor-neusomatic/simulated-bwa.INS.1-30.1.0.tsv - annotated-calls/varlociraptor-neusomatic/simulated-bwa.INS.30-100.1.0.tsv - annotated-calls/varlociraptor-delly/simulated-bwa.INS.1-30.1.0.tsv - annotated-calls/varlociraptor-delly/simulated-bwa.INS.30-100.1.0.tsv - annotated-calls/varlociraptor-lancet/simulated-bwa.INS.1-30.1.0.tsv - annotated-calls/varlociraptor-lancet/simulated-bwa.INS.30-100.1.0.tsv - annotated-calls/varlociraptor-manta/simulated-bwa.INS.1-30.1.0.tsv - annotated-calls/varlociraptor-manta/simulated-bwa.INS.30-100.1.0.tsv - annotated-calls/varlociraptor-strelka/simulated-bwa.INS.1-30.1.0.tsv - annotated-calls/varlociraptor-strelka/simulated-bwa.INS.30-100.1.0.tsv - annotated-calls/default-neusomatic/simulated-bwa.INS.1-30.1.0.tsv - annotated-calls/default-neusomatic/simulated-bwa.INS.30-100.1.0.tsv - annotated-calls/default-lancet/simulated-bwa.INS.1-30.1.0.tsv - annotated-calls/default-lancet/simulated-bwa.INS.30-100.1.0.tsv - annotated-calls/default-manta/simulated-bwa.INS.1-30.1.0.tsv - annotated-calls/default-manta/simulated-bwa.INS.30-100.1.0.tsv - annotated-calls/default-strelka/simulated-bwa.INS.1-30.1.0.tsv - annotated-calls/default-strelka/simulated-bwa.INS.30-100.1.0.tsv - annotated-calls/adhoc-neusomatic/simulated-bwa.INS.1-30.1.0.tsv - annotated-calls/adhoc-neusomatic/simulated-bwa.INS.30-100.1.0.tsv - annotated-calls/adhoc-delly/simulated-bwa.INS.1-30.1.0.tsv - annotated-calls/adhoc-delly/simulated-bwa.INS.30-100.1.0.tsv - annotated-calls/adhoc-lancet/simulated-bwa.INS.1-30.1.0.tsv - annotated-calls/adhoc-lancet/simulated-bwa.INS.30-100.1.0.tsv - annotated-calls/adhoc-manta/simulated-bwa.INS.1-30.1.0.tsv - annotated-calls/adhoc-manta/simulated-bwa.INS.30-100.1.0.tsv - annotated-calls/adhoc-strelka/simulated-bwa.INS.1-30.1.0.tsv - annotated-calls/adhoc-strelka/simulated-bwa.INS.30-100.1.0.tsv - annotated-calls/adhoc-bpi/simulated-bwa.INS.1-30.1.0.tsv - annotated-calls/adhoc-bpi/simulated-bwa.INS.30-100.1.0.tsv - annotated-calls/varlociraptor-neusomatic/synthetic-5.INS.1-250.1.0.tsv - annotated-calls/varlociraptor-delly/synthetic-5.INS.1-250.1.0.tsv - annotated-calls/varlociraptor-lancet/synthetic-5.INS.1-250.1.0.tsv - annotated-calls/varlociraptor-manta/synthetic-5.INS.1-250.1.0.tsv - annotated-calls/varlociraptor-strelka/synthetic-5.INS.1-250.1.0.tsv - annotated-calls/default-neusomatic/synthetic-5.INS.1-250.1.0.tsv - annotated-calls/default-lancet/synthetic-5.INS.1-250.1.0.tsv - annotated-calls/default-manta/synthetic-5.INS.1-250.1.0.tsv - annotated-calls/default-strelka/synthetic-5.INS.1-250.1.0.tsv - annotated-calls/adhoc-neusomatic/synthetic-5.INS.1-250.1.0.tsv - annotated-calls/adhoc-delly/synthetic-5.INS.1-250.1.0.tsv - annotated-calls/adhoc-lancet/synthetic-5.INS.1-250.1.0.tsv - annotated-calls/adhoc-manta/synthetic-5.INS.1-250.1.0.tsv - annotated-calls/adhoc-strelka/synthetic-5.INS.1-250.1.0.tsv - annotated-calls/adhoc-bpi/synthetic-5.INS.1-250.1.0.tsv - annotated-calls/varlociraptor-neusomatic/synthetic-20.INS.1-30.1.0.tsv - annotated-calls/varlociraptor-neusomatic/synthetic-20.INS.30-250.1.0.tsv - annotated-calls/varlociraptor-delly/synthetic-20.INS.1-30.1.0.tsv - annotated-calls/varlociraptor-delly/synthetic-20.INS.30-250.1.0.tsv - annotated-calls/varlociraptor-lancet/synthetic-20.INS.1-30.1.0.tsv - annotated-calls/varlociraptor-lancet/synthetic-20.INS.30-250.1.0.tsv - annotated-calls/varlociraptor-manta/synthetic-20.INS.1-30.1.0.tsv - annotated-calls/varlociraptor-manta/synthetic-20.INS.30-250.1.0.tsv - annotated-calls/varlociraptor-strelka/synthetic-20.INS.1-30.1.0.tsv - annotated-calls/varlociraptor-strelka/synthetic-20.INS.30-250.1.0.tsv - annotated-calls/default-neusomatic/synthetic-20.INS.1-30.1.0.tsv - annotated-calls/default-neusomatic/synthetic-20.INS.30-250.1.0.tsv - annotated-calls/default-lancet/synthetic-20.INS.1-30.1.0.tsv - annotated-calls/default-lancet/synthetic-20.INS.30-250.1.0.tsv - annotated-calls/default-manta/synthetic-20.INS.1-30.1.0.tsv - annotated-calls/default-manta/synthetic-20.INS.30-250.1.0.tsv - annotated-calls/default-strelka/synthetic-20.INS.1-30.1.0.tsv - annotated-calls/default-strelka/synthetic-20.INS.30-250.1.0.tsv - annotated-calls/adhoc-neusomatic/synthetic-20.INS.1-30.1.0.tsv - annotated-calls/adhoc-neusomatic/synthetic-20.INS.30-250.1.0.tsv - annotated-calls/adhoc-delly/synthetic-20.INS.1-30.1.0.tsv - annotated-calls/adhoc-delly/synthetic-20.INS.30-250.1.0.tsv - annotated-calls/adhoc-lancet/synthetic-20.INS.1-30.1.0.tsv - annotated-calls/adhoc-lancet/synthetic-20.INS.30-250.1.0.tsv - annotated-calls/adhoc-manta/synthetic-20.INS.1-30.1.0.tsv - annotated-calls/adhoc-manta/synthetic-20.INS.30-250.1.0.tsv - annotated-calls/adhoc-strelka/synthetic-20.INS.1-30.1.0.tsv - annotated-calls/adhoc-strelka/synthetic-20.INS.30-250.1.0.tsv - annotated-calls/adhoc-bpi/synthetic-20.INS.1-30.1.0.tsv - annotated-calls/adhoc-bpi/synthetic-20.INS.30-250.1.0.tsv - annotated-calls/varlociraptor-neusomatic/simulated-bwa.DEL.1-30.1.0.tsv - annotated-calls/varlociraptor-neusomatic/simulated-bwa.DEL.30-50.1.0.tsv - annotated-calls/varlociraptor-neusomatic/simulated-bwa.DEL.50-100.1.0.tsv - annotated-calls/varlociraptor-neusomatic/simulated-bwa.DEL.100-250.1.0.tsv - annotated-calls/varlociraptor-delly/simulated-bwa.DEL.1-30.1.0.tsv - annotated-calls/varlociraptor-delly/simulated-bwa.DEL.30-50.1.0.tsv - annotated-calls/varlociraptor-delly/simulated-bwa.DEL.50-100.1.0.tsv - annotated-calls/varlociraptor-delly/simulated-bwa.DEL.100-250.1.0.tsv - annotated-calls/varlociraptor-lancet/simulated-bwa.DEL.1-30.1.0.tsv - annotated-calls/varlociraptor-lancet/simulated-bwa.DEL.30-50.1.0.tsv - annotated-calls/varlociraptor-lancet/simulated-bwa.DEL.50-100.1.0.tsv - annotated-calls/varlociraptor-lancet/simulated-bwa.DEL.100-250.1.0.tsv - annotated-calls/varlociraptor-manta/simulated-bwa.DEL.1-30.1.0.tsv - annotated-calls/varlociraptor-manta/simulated-bwa.DEL.30-50.1.0.tsv - annotated-calls/varlociraptor-manta/simulated-bwa.DEL.50-100.1.0.tsv - annotated-calls/varlociraptor-manta/simulated-bwa.DEL.100-250.1.0.tsv - annotated-calls/varlociraptor-strelka/simulated-bwa.DEL.1-30.1.0.tsv - annotated-calls/varlociraptor-strelka/simulated-bwa.DEL.30-50.1.0.tsv - annotated-calls/varlociraptor-strelka/simulated-bwa.DEL.50-100.1.0.tsv - annotated-calls/varlociraptor-strelka/simulated-bwa.DEL.100-250.1.0.tsv - annotated-calls/default-neusomatic/simulated-bwa.DEL.1-30.1.0.tsv - annotated-calls/default-neusomatic/simulated-bwa.DEL.30-50.1.0.tsv - annotated-calls/default-neusomatic/simulated-bwa.DEL.50-100.1.0.tsv - annotated-calls/default-neusomatic/simulated-bwa.DEL.100-250.1.0.tsv - annotated-calls/default-lancet/simulated-bwa.DEL.1-30.1.0.tsv - annotated-calls/default-lancet/simulated-bwa.DEL.30-50.1.0.tsv - annotated-calls/default-lancet/simulated-bwa.DEL.50-100.1.0.tsv - annotated-calls/default-lancet/simulated-bwa.DEL.100-250.1.0.tsv - annotated-calls/default-manta/simulated-bwa.DEL.1-30.1.0.tsv - annotated-calls/default-manta/simulated-bwa.DEL.30-50.1.0.tsv - annotated-calls/default-manta/simulated-bwa.DEL.50-100.1.0.tsv - annotated-calls/default-manta/simulated-bwa.DEL.100-250.1.0.tsv - annotated-calls/default-strelka/simulated-bwa.DEL.1-30.1.0.tsv - annotated-calls/default-strelka/simulated-bwa.DEL.30-50.1.0.tsv - annotated-calls/default-strelka/simulated-bwa.DEL.50-100.1.0.tsv - annotated-calls/default-strelka/simulated-bwa.DEL.100-250.1.0.tsv - annotated-calls/adhoc-neusomatic/simulated-bwa.DEL.1-30.1.0.tsv - annotated-calls/adhoc-neusomatic/simulated-bwa.DEL.30-50.1.0.tsv - annotated-calls/adhoc-neusomatic/simulated-bwa.DEL.50-100.1.0.tsv - annotated-calls/adhoc-neusomatic/simulated-bwa.DEL.100-250.1.0.tsv - annotated-calls/adhoc-delly/simulated-bwa.DEL.1-30.1.0.tsv - annotated-calls/adhoc-delly/simulated-bwa.DEL.30-50.1.0.tsv - annotated-calls/adhoc-delly/simulated-bwa.DEL.50-100.1.0.tsv - annotated-calls/adhoc-delly/simulated-bwa.DEL.100-250.1.0.tsv - annotated-calls/adhoc-lancet/simulated-bwa.DEL.1-30.1.0.tsv - annotated-calls/adhoc-lancet/simulated-bwa.DEL.30-50.1.0.tsv - annotated-calls/adhoc-lancet/simulated-bwa.DEL.50-100.1.0.tsv - annotated-calls/adhoc-lancet/simulated-bwa.DEL.100-250.1.0.tsv - annotated-calls/adhoc-manta/simulated-bwa.DEL.1-30.1.0.tsv - annotated-calls/adhoc-manta/simulated-bwa.DEL.30-50.1.0.tsv - annotated-calls/adhoc-manta/simulated-bwa.DEL.50-100.1.0.tsv - annotated-calls/adhoc-manta/simulated-bwa.DEL.100-250.1.0.tsv - annotated-calls/adhoc-strelka/simulated-bwa.DEL.1-30.1.0.tsv - annotated-calls/adhoc-strelka/simulated-bwa.DEL.30-50.1.0.tsv - annotated-calls/adhoc-strelka/simulated-bwa.DEL.50-100.1.0.tsv - annotated-calls/adhoc-strelka/simulated-bwa.DEL.100-250.1.0.tsv - annotated-calls/adhoc-bpi/simulated-bwa.DEL.1-30.1.0.tsv - annotated-calls/adhoc-bpi/simulated-bwa.DEL.30-50.1.0.tsv - annotated-calls/adhoc-bpi/simulated-bwa.DEL.50-100.1.0.tsv - annotated-calls/adhoc-bpi/simulated-bwa.DEL.100-250.1.0.tsv - annotated-calls/varlociraptor-neusomatic/synthetic-5.DEL.1-250.1.0.tsv - annotated-calls/varlociraptor-delly/synthetic-5.DEL.1-250.1.0.tsv - annotated-calls/varlociraptor-lancet/synthetic-5.DEL.1-250.1.0.tsv - annotated-calls/varlociraptor-manta/synthetic-5.DEL.1-250.1.0.tsv - annotated-calls/varlociraptor-strelka/synthetic-5.DEL.1-250.1.0.tsv - annotated-calls/default-neusomatic/synthetic-5.DEL.1-250.1.0.tsv - annotated-calls/default-lancet/synthetic-5.DEL.1-250.1.0.tsv - annotated-calls/default-manta/synthetic-5.DEL.1-250.1.0.tsv - annotated-calls/default-strelka/synthetic-5.DEL.1-250.1.0.tsv - annotated-calls/adhoc-neusomatic/synthetic-5.DEL.1-250.1.0.tsv - annotated-calls/adhoc-delly/synthetic-5.DEL.1-250.1.0.tsv - annotated-calls/adhoc-lancet/synthetic-5.DEL.1-250.1.0.tsv - annotated-calls/adhoc-manta/synthetic-5.DEL.1-250.1.0.tsv - annotated-calls/adhoc-strelka/synthetic-5.DEL.1-250.1.0.tsv - annotated-calls/adhoc-bpi/synthetic-5.DEL.1-250.1.0.tsv - annotated-calls/varlociraptor-neusomatic/synthetic-20.DEL.1-30.1.0.tsv - annotated-calls/varlociraptor-neusomatic/synthetic-20.DEL.30-250.1.0.tsv - annotated-calls/varlociraptor-delly/synthetic-20.DEL.1-30.1.0.tsv - annotated-calls/varlociraptor-delly/synthetic-20.DEL.30-250.1.0.tsv - annotated-calls/varlociraptor-lancet/synthetic-20.DEL.1-30.1.0.tsv - annotated-calls/varlociraptor-lancet/synthetic-20.DEL.30-250.1.0.tsv - annotated-calls/varlociraptor-manta/synthetic-20.DEL.1-30.1.0.tsv - annotated-calls/varlociraptor-manta/synthetic-20.DEL.30-250.1.0.tsv - annotated-calls/varlociraptor-strelka/synthetic-20.DEL.1-30.1.0.tsv - annotated-calls/varlociraptor-strelka/synthetic-20.DEL.30-250.1.0.tsv - annotated-calls/default-neusomatic/synthetic-20.DEL.1-30.1.0.tsv - annotated-calls/default-neusomatic/synthetic-20.DEL.30-250.1.0.tsv - annotated-calls/default-lancet/synthetic-20.DEL.1-30.1.0.tsv - annotated-calls/default-lancet/synthetic-20.DEL.30-250.1.0.tsv - annotated-calls/default-manta/synthetic-20.DEL.1-30.1.0.tsv - annotated-calls/default-manta/synthetic-20.DEL.30-250.1.0.tsv - annotated-calls/default-strelka/synthetic-20.DEL.1-30.1.0.tsv - annotated-calls/default-strelka/synthetic-20.DEL.30-250.1.0.tsv - annotated-calls/adhoc-neusomatic/synthetic-20.DEL.1-30.1.0.tsv - annotated-calls/adhoc-neusomatic/synthetic-20.DEL.30-250.1.0.tsv - annotated-calls/adhoc-delly/synthetic-20.DEL.1-30.1.0.tsv - annotated-calls/adhoc-delly/synthetic-20.DEL.30-250.1.0.tsv - annotated-calls/adhoc-lancet/synthetic-20.DEL.1-30.1.0.tsv - annotated-calls/adhoc-lancet/synthetic-20.DEL.30-250.1.0.tsv - annotated-calls/adhoc-manta/synthetic-20.DEL.1-30.1.0.tsv - annotated-calls/adhoc-manta/synthetic-20.DEL.30-250.1.0.tsv - annotated-calls/adhoc-strelka/synthetic-20.DEL.1-30.1.0.tsv - annotated-calls/adhoc-strelka/synthetic-20.DEL.30-250.1.0.tsv - annotated-calls/adhoc-bpi/synthetic-20.DEL.1-30.1.0.tsv - annotated-calls/adhoc-bpi/synthetic-20.DEL.30-250.1.0.tsv - annotated-calls/varlociraptor-neusomatic/simulated-bwa.INS.1-30.1e-05.tsv - annotated-calls/varlociraptor-neusomatic/simulated-bwa.INS.1-30.0.0001.tsv - annotated-calls/varlociraptor-neusomatic/simulated-bwa.INS.1-30.0.001.tsv - annotated-calls/varlociraptor-neusomatic/simulated-bwa.INS.1-30.0.01.tsv - annotated-calls/varlociraptor-neusomatic/simulated-bwa.INS.1-30.0.05.tsv - annotated-calls/varlociraptor-neusomatic/simulated-bwa.INS.1-30.0.1.tsv - annotated-calls/varlociraptor-neusomatic/simulated-bwa.INS.1-30.0.2.tsv - annotated-calls/varlociraptor-neusomatic/simulated-bwa.INS.1-30.0.3.tsv - annotated-calls/varlociraptor-neusomatic/simulated-bwa.INS.1-30.0.4.tsv - annotated-calls/varlociraptor-neusomatic/simulated-bwa.INS.1-30.0.5.tsv - annotated-calls/varlociraptor-neusomatic/simulated-bwa.INS.1-30.0.6.tsv - annotated-calls/varlociraptor-neusomatic/simulated-bwa.INS.1-30.0.7.tsv - annotated-calls/varlociraptor-neusomatic/simulated-bwa.INS.1-30.0.8.tsv - annotated-calls/varlociraptor-neusomatic/simulated-bwa.INS.1-30.0.9.tsv - annotated-calls/varlociraptor-neusomatic/simulated-bwa.INS.30-100.1e-05.tsv - annotated-calls/varlociraptor-neusomatic/simulated-bwa.INS.30-100.0.0001.tsv - annotated-calls/varlociraptor-neusomatic/simulated-bwa.INS.30-100.0.001.tsv - annotated-calls/varlociraptor-neusomatic/simulated-bwa.INS.30-100.0.01.tsv - annotated-calls/varlociraptor-neusomatic/simulated-bwa.INS.30-100.0.05.tsv - annotated-calls/varlociraptor-neusomatic/simulated-bwa.INS.30-100.0.1.tsv - annotated-calls/varlociraptor-neusomatic/simulated-bwa.INS.30-100.0.2.tsv - annotated-calls/varlociraptor-neusomatic/simulated-bwa.INS.30-100.0.3.tsv - annotated-calls/varlociraptor-neusomatic/simulated-bwa.INS.30-100.0.4.tsv - annotated-calls/varlociraptor-neusomatic/simulated-bwa.INS.30-100.0.5.tsv - annotated-calls/varlociraptor-neusomatic/simulated-bwa.INS.30-100.0.6.tsv - annotated-calls/varlociraptor-neusomatic/simulated-bwa.INS.30-100.0.7.tsv - annotated-calls/varlociraptor-neusomatic/simulated-bwa.INS.30-100.0.8.tsv - annotated-calls/varlociraptor-neusomatic/simulated-bwa.INS.30-100.0.9.tsv - annotated-calls/varlociraptor-delly/simulated-bwa.INS.1-30.1e-05.tsv - annotated-calls/varlociraptor-delly/simulated-bwa.INS.1-30.0.0001.tsv - annotated-calls/varlociraptor-delly/simulated-bwa.INS.1-30.0.001.tsv - annotated-calls/varlociraptor-delly/simulated-bwa.INS.1-30.0.01.tsv - annotated-calls/varlociraptor-delly/simulated-bwa.INS.1-30.0.05.tsv - annotated-calls/varlociraptor-delly/simulated-bwa.INS.1-30.0.1.tsv - annotated-calls/varlociraptor-delly/simulated-bwa.INS.1-30.0.2.tsv - annotated-calls/varlociraptor-delly/simulated-bwa.INS.1-30.0.3.tsv - annotated-calls/varlociraptor-delly/simulated-bwa.INS.1-30.0.4.tsv - annotated-calls/varlociraptor-delly/simulated-bwa.INS.1-30.0.5.tsv - annotated-calls/varlociraptor-delly/simulated-bwa.INS.1-30.0.6.tsv - annotated-calls/varlociraptor-delly/simulated-bwa.INS.1-30.0.7.tsv - annotated-calls/varlociraptor-delly/simulated-bwa.INS.1-30.0.8.tsv - annotated-calls/varlociraptor-delly/simulated-bwa.INS.1-30.0.9.tsv - annotated-calls/varlociraptor-delly/simulated-bwa.INS.30-100.1e-05.tsv - annotated-calls/varlociraptor-delly/simulated-bwa.INS.30-100.0.0001.tsv - annotated-calls/varlociraptor-delly/simulated-bwa.INS.30-100.0.001.tsv - annotated-calls/varlociraptor-delly/simulated-bwa.INS.30-100.0.01.tsv - annotated-calls/varlociraptor-delly/simulated-bwa.INS.30-100.0.05.tsv - annotated-calls/varlociraptor-delly/simulated-bwa.INS.30-100.0.1.tsv - annotated-calls/varlociraptor-delly/simulated-bwa.INS.30-100.0.2.tsv - annotated-calls/varlociraptor-delly/simulated-bwa.INS.30-100.0.3.tsv - annotated-calls/varlociraptor-delly/simulated-bwa.INS.30-100.0.4.tsv - annotated-calls/varlociraptor-delly/simulated-bwa.INS.30-100.0.5.tsv - annotated-calls/varlociraptor-delly/simulated-bwa.INS.30-100.0.6.tsv - annotated-calls/varlociraptor-delly/simulated-bwa.INS.30-100.0.7.tsv - annotated-calls/varlociraptor-delly/simulated-bwa.INS.30-100.0.8.tsv - annotated-calls/varlociraptor-delly/simulated-bwa.INS.30-100.0.9.tsv - annotated-calls/varlociraptor-lancet/simulated-bwa.INS.1-30.1e-05.tsv - annotated-calls/varlociraptor-lancet/simulated-bwa.INS.1-30.0.0001.tsv - annotated-calls/varlociraptor-lancet/simulated-bwa.INS.1-30.0.001.tsv - annotated-calls/varlociraptor-lancet/simulated-bwa.INS.1-30.0.01.tsv - annotated-calls/varlociraptor-lancet/simulated-bwa.INS.1-30.0.05.tsv - annotated-calls/varlociraptor-lancet/simulated-bwa.INS.1-30.0.1.tsv - annotated-calls/varlociraptor-lancet/simulated-bwa.INS.1-30.0.2.tsv - annotated-calls/varlociraptor-lancet/simulated-bwa.INS.1-30.0.3.tsv - annotated-calls/varlociraptor-lancet/simulated-bwa.INS.1-30.0.4.tsv - annotated-calls/varlociraptor-lancet/simulated-bwa.INS.1-30.0.5.tsv - annotated-calls/varlociraptor-lancet/simulated-bwa.INS.1-30.0.6.tsv - annotated-calls/varlociraptor-lancet/simulated-bwa.INS.1-30.0.7.tsv - annotated-calls/varlociraptor-lancet/simulated-bwa.INS.1-30.0.8.tsv - annotated-calls/varlociraptor-lancet/simulated-bwa.INS.1-30.0.9.tsv - annotated-calls/varlociraptor-lancet/simulated-bwa.INS.30-100.1e-05.tsv - annotated-calls/varlociraptor-lancet/simulated-bwa.INS.30-100.0.0001.tsv - annotated-calls/varlociraptor-lancet/simulated-bwa.INS.30-100.0.001.tsv - annotated-calls/varlociraptor-lancet/simulated-bwa.INS.30-100.0.01.tsv - annotated-calls/varlociraptor-lancet/simulated-bwa.INS.30-100.0.05.tsv - annotated-calls/varlociraptor-lancet/simulated-bwa.INS.30-100.0.1.tsv - annotated-calls/varlociraptor-lancet/simulated-bwa.INS.30-100.0.2.tsv - annotated-calls/varlociraptor-lancet/simulated-bwa.INS.30-100.0.3.tsv - annotated-calls/varlociraptor-lancet/simulated-bwa.INS.30-100.0.4.tsv - annotated-calls/varlociraptor-lancet/simulated-bwa.INS.30-100.0.5.tsv - annotated-calls/varlociraptor-lancet/simulated-bwa.INS.30-100.0.6.tsv - annotated-calls/varlociraptor-lancet/simulated-bwa.INS.30-100.0.7.tsv - annotated-calls/varlociraptor-lancet/simulated-bwa.INS.30-100.0.8.tsv - annotated-calls/varlociraptor-lancet/simulated-bwa.INS.30-100.0.9.tsv - annotated-calls/varlociraptor-manta/simulated-bwa.INS.1-30.1e-05.tsv - annotated-calls/varlociraptor-manta/simulated-bwa.INS.1-30.0.0001.tsv - annotated-calls/varlociraptor-manta/simulated-bwa.INS.1-30.0.001.tsv - annotated-calls/varlociraptor-manta/simulated-bwa.INS.1-30.0.01.tsv - annotated-calls/varlociraptor-manta/simulated-bwa.INS.1-30.0.05.tsv - annotated-calls/varlociraptor-manta/simulated-bwa.INS.1-30.0.1.tsv - annotated-calls/varlociraptor-manta/simulated-bwa.INS.1-30.0.2.tsv - annotated-calls/varlociraptor-manta/simulated-bwa.INS.1-30.0.3.tsv - annotated-calls/varlociraptor-manta/simulated-bwa.INS.1-30.0.4.tsv - annotated-calls/varlociraptor-manta/simulated-bwa.INS.1-30.0.5.tsv - annotated-calls/varlociraptor-manta/simulated-bwa.INS.1-30.0.6.tsv - annotated-calls/varlociraptor-manta/simulated-bwa.INS.1-30.0.7.tsv - annotated-calls/varlociraptor-manta/simulated-bwa.INS.1-30.0.8.tsv - annotated-calls/varlociraptor-manta/simulated-bwa.INS.1-30.0.9.tsv - annotated-calls/varlociraptor-manta/simulated-bwa.INS.30-100.1e-05.tsv - annotated-calls/varlociraptor-manta/simulated-bwa.INS.30-100.0.0001.tsv - annotated-calls/varlociraptor-manta/simulated-bwa.INS.30-100.0.001.tsv - annotated-calls/varlociraptor-manta/simulated-bwa.INS.30-100.0.01.tsv - annotated-calls/varlociraptor-manta/simulated-bwa.INS.30-100.0.05.tsv - annotated-calls/varlociraptor-manta/simulated-bwa.INS.30-100.0.1.tsv - annotated-calls/varlociraptor-manta/simulated-bwa.INS.30-100.0.2.tsv - annotated-calls/varlociraptor-manta/simulated-bwa.INS.30-100.0.3.tsv - annotated-calls/varlociraptor-manta/simulated-bwa.INS.30-100.0.4.tsv - annotated-calls/varlociraptor-manta/simulated-bwa.INS.30-100.0.5.tsv - annotated-calls/varlociraptor-manta/simulated-bwa.INS.30-100.0.6.tsv - annotated-calls/varlociraptor-manta/simulated-bwa.INS.30-100.0.7.tsv - annotated-calls/varlociraptor-manta/simulated-bwa.INS.30-100.0.8.tsv - annotated-calls/varlociraptor-manta/simulated-bwa.INS.30-100.0.9.tsv - annotated-calls/varlociraptor-strelka/simulated-bwa.INS.1-30.1e-05.tsv - annotated-calls/varlociraptor-strelka/simulated-bwa.INS.1-30.0.0001.tsv - annotated-calls/varlociraptor-strelka/simulated-bwa.INS.1-30.0.001.tsv - annotated-calls/varlociraptor-strelka/simulated-bwa.INS.1-30.0.01.tsv - annotated-calls/varlociraptor-strelka/simulated-bwa.INS.1-30.0.05.tsv - annotated-calls/varlociraptor-strelka/simulated-bwa.INS.1-30.0.1.tsv - annotated-calls/varlociraptor-strelka/simulated-bwa.INS.1-30.0.2.tsv - annotated-calls/varlociraptor-strelka/simulated-bwa.INS.1-30.0.3.tsv - annotated-calls/varlociraptor-strelka/simulated-bwa.INS.1-30.0.4.tsv - annotated-calls/varlociraptor-strelka/simulated-bwa.INS.1-30.0.5.tsv - annotated-calls/varlociraptor-strelka/simulated-bwa.INS.1-30.0.6.tsv - annotated-calls/varlociraptor-strelka/simulated-bwa.INS.1-30.0.7.tsv - annotated-calls/varlociraptor-strelka/simulated-bwa.INS.1-30.0.8.tsv - annotated-calls/varlociraptor-strelka/simulated-bwa.INS.1-30.0.9.tsv - annotated-calls/varlociraptor-strelka/simulated-bwa.INS.30-100.1e-05.tsv - annotated-calls/varlociraptor-strelka/simulated-bwa.INS.30-100.0.0001.tsv - annotated-calls/varlociraptor-strelka/simulated-bwa.INS.30-100.0.001.tsv - annotated-calls/varlociraptor-strelka/simulated-bwa.INS.30-100.0.01.tsv - annotated-calls/varlociraptor-strelka/simulated-bwa.INS.30-100.0.05.tsv - annotated-calls/varlociraptor-strelka/simulated-bwa.INS.30-100.0.1.tsv - annotated-calls/varlociraptor-strelka/simulated-bwa.INS.30-100.0.2.tsv - annotated-calls/varlociraptor-strelka/simulated-bwa.INS.30-100.0.3.tsv - annotated-calls/varlociraptor-strelka/simulated-bwa.INS.30-100.0.4.tsv - annotated-calls/varlociraptor-strelka/simulated-bwa.INS.30-100.0.5.tsv - annotated-calls/varlociraptor-strelka/simulated-bwa.INS.30-100.0.6.tsv - annotated-calls/varlociraptor-strelka/simulated-bwa.INS.30-100.0.7.tsv - annotated-calls/varlociraptor-strelka/simulated-bwa.INS.30-100.0.8.tsv - annotated-calls/varlociraptor-strelka/simulated-bwa.INS.30-100.0.9.tsv - annotated-calls/varlociraptor-neusomatic/simulated-bwa.DEL.1-30.1e-05.tsv - annotated-calls/varlociraptor-neusomatic/simulated-bwa.DEL.1-30.0.0001.tsv - annotated-calls/varlociraptor-neusomatic/simulated-bwa.DEL.1-30.0.001.tsv - annotated-calls/varlociraptor-neusomatic/simulated-bwa.DEL.1-30.0.01.tsv - annotated-calls/varlociraptor-neusomatic/simulated-bwa.DEL.1-30.0.05.tsv - annotated-calls/varlociraptor-neusomatic/simulated-bwa.DEL.1-30.0.1.tsv - annotated-calls/varlociraptor-neusomatic/simulated-bwa.DEL.1-30.0.2.tsv - annotated-calls/varlociraptor-neusomatic/simulated-bwa.DEL.1-30.0.3.tsv - annotated-calls/varlociraptor-neusomatic/simulated-bwa.DEL.1-30.0.4.tsv - annotated-calls/varlociraptor-neusomatic/simulated-bwa.DEL.1-30.0.5.tsv - annotated-calls/varlociraptor-neusomatic/simulated-bwa.DEL.1-30.0.6.tsv - annotated-calls/varlociraptor-neusomatic/simulated-bwa.DEL.1-30.0.7.tsv - annotated-calls/varlociraptor-neusomatic/simulated-bwa.DEL.1-30.0.8.tsv - annotated-calls/varlociraptor-neusomatic/simulated-bwa.DEL.1-30.0.9.tsv - annotated-calls/varlociraptor-neusomatic/simulated-bwa.DEL.30-50.1e-05.tsv - annotated-calls/varlociraptor-neusomatic/simulated-bwa.DEL.30-50.0.0001.tsv - annotated-calls/varlociraptor-neusomatic/simulated-bwa.DEL.30-50.0.001.tsv - annotated-calls/varlociraptor-neusomatic/simulated-bwa.DEL.30-50.0.01.tsv - annotated-calls/varlociraptor-neusomatic/simulated-bwa.DEL.30-50.0.05.tsv - annotated-calls/varlociraptor-neusomatic/simulated-bwa.DEL.30-50.0.1.tsv - annotated-calls/varlociraptor-neusomatic/simulated-bwa.DEL.30-50.0.2.tsv - annotated-calls/varlociraptor-neusomatic/simulated-bwa.DEL.30-50.0.3.tsv - annotated-calls/varlociraptor-neusomatic/simulated-bwa.DEL.30-50.0.4.tsv - annotated-calls/varlociraptor-neusomatic/simulated-bwa.DEL.30-50.0.5.tsv - annotated-calls/varlociraptor-neusomatic/simulated-bwa.DEL.30-50.0.6.tsv - annotated-calls/varlociraptor-neusomatic/simulated-bwa.DEL.30-50.0.7.tsv - annotated-calls/varlociraptor-neusomatic/simulated-bwa.DEL.30-50.0.8.tsv - annotated-calls/varlociraptor-neusomatic/simulated-bwa.DEL.30-50.0.9.tsv - annotated-calls/varlociraptor-neusomatic/simulated-bwa.DEL.50-100.1e-05.tsv - annotated-calls/varlociraptor-neusomatic/simulated-bwa.DEL.50-100.0.0001.tsv - annotated-calls/varlociraptor-neusomatic/simulated-bwa.DEL.50-100.0.001.tsv - annotated-calls/varlociraptor-neusomatic/simulated-bwa.DEL.50-100.0.01.tsv - annotated-calls/varlociraptor-neusomatic/simulated-bwa.DEL.50-100.0.05.tsv - annotated-calls/varlociraptor-neusomatic/simulated-bwa.DEL.50-100.0.1.tsv - annotated-calls/varlociraptor-neusomatic/simulated-bwa.DEL.50-100.0.2.tsv - annotated-calls/varlociraptor-neusomatic/simulated-bwa.DEL.50-100.0.3.tsv - annotated-calls/varlociraptor-neusomatic/simulated-bwa.DEL.50-100.0.4.tsv - annotated-calls/varlociraptor-neusomatic/simulated-bwa.DEL.50-100.0.5.tsv - annotated-calls/varlociraptor-neusomatic/simulated-bwa.DEL.50-100.0.6.tsv - annotated-calls/varlociraptor-neusomatic/simulated-bwa.DEL.50-100.0.7.tsv - annotated-calls/varlociraptor-neusomatic/simulated-bwa.DEL.50-100.0.8.tsv - annotated-calls/varlociraptor-neusomatic/simulated-bwa.DEL.50-100.0.9.tsv - annotated-calls/varlociraptor-neusomatic/simulated-bwa.DEL.100-250.1e-05.tsv - annotated-calls/varlociraptor-neusomatic/simulated-bwa.DEL.100-250.0.0001.tsv - annotated-calls/varlociraptor-neusomatic/simulated-bwa.DEL.100-250.0.001.tsv - annotated-calls/varlociraptor-neusomatic/simulated-bwa.DEL.100-250.0.01.tsv - annotated-calls/varlociraptor-neusomatic/simulated-bwa.DEL.100-250.0.05.tsv - annotated-calls/varlociraptor-neusomatic/simulated-bwa.DEL.100-250.0.1.tsv - annotated-calls/varlociraptor-neusomatic/simulated-bwa.DEL.100-250.0.2.tsv - annotated-calls/varlociraptor-neusomatic/simulated-bwa.DEL.100-250.0.3.tsv - annotated-calls/varlociraptor-neusomatic/simulated-bwa.DEL.100-250.0.4.tsv - annotated-calls/varlociraptor-neusomatic/simulated-bwa.DEL.100-250.0.5.tsv - annotated-calls/varlociraptor-neusomatic/simulated-bwa.DEL.100-250.0.6.tsv - annotated-calls/varlociraptor-neusomatic/simulated-bwa.DEL.100-250.0.7.tsv - annotated-calls/varlociraptor-neusomatic/simulated-bwa.DEL.100-250.0.8.tsv - annotated-calls/varlociraptor-neusomatic/simulated-bwa.DEL.100-250.0.9.tsv - annotated-calls/varlociraptor-delly/simulated-bwa.DEL.1-30.1e-05.tsv - annotated-calls/varlociraptor-delly/simulated-bwa.DEL.1-30.0.0001.tsv - annotated-calls/varlociraptor-delly/simulated-bwa.DEL.1-30.0.001.tsv - annotated-calls/varlociraptor-delly/simulated-bwa.DEL.1-30.0.01.tsv - annotated-calls/varlociraptor-delly/simulated-bwa.DEL.1-30.0.05.tsv - annotated-calls/varlociraptor-delly/simulated-bwa.DEL.1-30.0.1.tsv - annotated-calls/varlociraptor-delly/simulated-bwa.DEL.1-30.0.2.tsv - annotated-calls/varlociraptor-delly/simulated-bwa.DEL.1-30.0.3.tsv - annotated-calls/varlociraptor-delly/simulated-bwa.DEL.1-30.0.4.tsv - annotated-calls/varlociraptor-delly/simulated-bwa.DEL.1-30.0.5.tsv - annotated-calls/varlociraptor-delly/simulated-bwa.DEL.1-30.0.6.tsv - annotated-calls/varlociraptor-delly/simulated-bwa.DEL.1-30.0.7.tsv - annotated-calls/varlociraptor-delly/simulated-bwa.DEL.1-30.0.8.tsv - annotated-calls/varlociraptor-delly/simulated-bwa.DEL.1-30.0.9.tsv - annotated-calls/varlociraptor-delly/simulated-bwa.DEL.30-50.1e-05.tsv - annotated-calls/varlociraptor-delly/simulated-bwa.DEL.30-50.0.0001.tsv - annotated-calls/varlociraptor-delly/simulated-bwa.DEL.30-50.0.001.tsv - annotated-calls/varlociraptor-delly/simulated-bwa.DEL.30-50.0.01.tsv - annotated-calls/varlociraptor-delly/simulated-bwa.DEL.30-50.0.05.tsv - annotated-calls/varlociraptor-delly/simulated-bwa.DEL.30-50.0.1.tsv - annotated-calls/varlociraptor-delly/simulated-bwa.DEL.30-50.0.2.tsv - annotated-calls/varlociraptor-delly/simulated-bwa.DEL.30-50.0.3.tsv - annotated-calls/varlociraptor-delly/simulated-bwa.DEL.30-50.0.4.tsv - annotated-calls/varlociraptor-delly/simulated-bwa.DEL.30-50.0.5.tsv - annotated-calls/varlociraptor-delly/simulated-bwa.DEL.30-50.0.6.tsv - annotated-calls/varlociraptor-delly/simulated-bwa.DEL.30-50.0.7.tsv - annotated-calls/varlociraptor-delly/simulated-bwa.DEL.30-50.0.8.tsv - annotated-calls/varlociraptor-delly/simulated-bwa.DEL.30-50.0.9.tsv - annotated-calls/varlociraptor-delly/simulated-bwa.DEL.50-100.1e-05.tsv - annotated-calls/varlociraptor-delly/simulated-bwa.DEL.50-100.0.0001.tsv - annotated-calls/varlociraptor-delly/simulated-bwa.DEL.50-100.0.001.tsv - annotated-calls/varlociraptor-delly/simulated-bwa.DEL.50-100.0.01.tsv - annotated-calls/varlociraptor-delly/simulated-bwa.DEL.50-100.0.05.tsv - annotated-calls/varlociraptor-delly/simulated-bwa.DEL.50-100.0.1.tsv - annotated-calls/varlociraptor-delly/simulated-bwa.DEL.50-100.0.2.tsv - annotated-calls/varlociraptor-delly/simulated-bwa.DEL.50-100.0.3.tsv - annotated-calls/varlociraptor-delly/simulated-bwa.DEL.50-100.0.4.tsv - annotated-calls/varlociraptor-delly/simulated-bwa.DEL.50-100.0.5.tsv - annotated-calls/varlociraptor-delly/simulated-bwa.DEL.50-100.0.6.tsv - annotated-calls/varlociraptor-delly/simulated-bwa.DEL.50-100.0.7.tsv - annotated-calls/varlociraptor-delly/simulated-bwa.DEL.50-100.0.8.tsv - annotated-calls/varlociraptor-delly/simulated-bwa.DEL.50-100.0.9.tsv - annotated-calls/varlociraptor-delly/simulated-bwa.DEL.100-250.1e-05.tsv - annotated-calls/varlociraptor-delly/simulated-bwa.DEL.100-250.0.0001.tsv - annotated-calls/varlociraptor-delly/simulated-bwa.DEL.100-250.0.001.tsv - annotated-calls/varlociraptor-delly/simulated-bwa.DEL.100-250.0.01.tsv - annotated-calls/varlociraptor-delly/simulated-bwa.DEL.100-250.0.05.tsv - annotated-calls/varlociraptor-delly/simulated-bwa.DEL.100-250.0.1.tsv - annotated-calls/varlociraptor-delly/simulated-bwa.DEL.100-250.0.2.tsv - annotated-calls/varlociraptor-delly/simulated-bwa.DEL.100-250.0.3.tsv - annotated-calls/varlociraptor-delly/simulated-bwa.DEL.100-250.0.4.tsv - annotated-calls/varlociraptor-delly/simulated-bwa.DEL.100-250.0.5.tsv - annotated-calls/varlociraptor-delly/simulated-bwa.DEL.100-250.0.6.tsv - annotated-calls/varlociraptor-delly/simulated-bwa.DEL.100-250.0.7.tsv - annotated-calls/varlociraptor-delly/simulated-bwa.DEL.100-250.0.8.tsv - annotated-calls/varlociraptor-delly/simulated-bwa.DEL.100-250.0.9.tsv - annotated-calls/varlociraptor-lancet/simulated-bwa.DEL.1-30.1e-05.tsv - annotated-calls/varlociraptor-lancet/simulated-bwa.DEL.1-30.0.0001.tsv - annotated-calls/varlociraptor-lancet/simulated-bwa.DEL.1-30.0.001.tsv - annotated-calls/varlociraptor-lancet/simulated-bwa.DEL.1-30.0.01.tsv - annotated-calls/varlociraptor-lancet/simulated-bwa.DEL.1-30.0.05.tsv - annotated-calls/varlociraptor-lancet/simulated-bwa.DEL.1-30.0.1.tsv - annotated-calls/varlociraptor-lancet/simulated-bwa.DEL.1-30.0.2.tsv - annotated-calls/varlociraptor-lancet/simulated-bwa.DEL.1-30.0.3.tsv - annotated-calls/varlociraptor-lancet/simulated-bwa.DEL.1-30.0.4.tsv - annotated-calls/varlociraptor-lancet/simulated-bwa.DEL.1-30.0.5.tsv - annotated-calls/varlociraptor-lancet/simulated-bwa.DEL.1-30.0.6.tsv - annotated-calls/varlociraptor-lancet/simulated-bwa.DEL.1-30.0.7.tsv - annotated-calls/varlociraptor-lancet/simulated-bwa.DEL.1-30.0.8.tsv - annotated-calls/varlociraptor-lancet/simulated-bwa.DEL.1-30.0.9.tsv - annotated-calls/varlociraptor-lancet/simulated-bwa.DEL.30-50.1e-05.tsv - annotated-calls/varlociraptor-lancet/simulated-bwa.DEL.30-50.0.0001.tsv - annotated-calls/varlociraptor-lancet/simulated-bwa.DEL.30-50.0.001.tsv - annotated-calls/varlociraptor-lancet/simulated-bwa.DEL.30-50.0.01.tsv - annotated-calls/varlociraptor-lancet/simulated-bwa.DEL.30-50.0.05.tsv - annotated-calls/varlociraptor-lancet/simulated-bwa.DEL.30-50.0.1.tsv - annotated-calls/varlociraptor-lancet/simulated-bwa.DEL.30-50.0.2.tsv - annotated-calls/varlociraptor-lancet/simulated-bwa.DEL.30-50.0.3.tsv - annotated-calls/varlociraptor-lancet/simulated-bwa.DEL.30-50.0.4.tsv - annotated-calls/varlociraptor-lancet/simulated-bwa.DEL.30-50.0.5.tsv - annotated-calls/varlociraptor-lancet/simulated-bwa.DEL.30-50.0.6.tsv - annotated-calls/varlociraptor-lancet/simulated-bwa.DEL.30-50.0.7.tsv - annotated-calls/varlociraptor-lancet/simulated-bwa.DEL.30-50.0.8.tsv - annotated-calls/varlociraptor-lancet/simulated-bwa.DEL.30-50.0.9.tsv - annotated-calls/varlociraptor-lancet/simulated-bwa.DEL.50-100.1e-05.tsv - annotated-calls/varlociraptor-lancet/simulated-bwa.DEL.50-100.0.0001.tsv - annotated-calls/varlociraptor-lancet/simulated-bwa.DEL.50-100.0.001.tsv - annotated-calls/varlociraptor-lancet/simulated-bwa.DEL.50-100.0.01.tsv - annotated-calls/varlociraptor-lancet/simulated-bwa.DEL.50-100.0.05.tsv - annotated-calls/varlociraptor-lancet/simulated-bwa.DEL.50-100.0.1.tsv - annotated-calls/varlociraptor-lancet/simulated-bwa.DEL.50-100.0.2.tsv - annotated-calls/varlociraptor-lancet/simulated-bwa.DEL.50-100.0.3.tsv - annotated-calls/varlociraptor-lancet/simulated-bwa.DEL.50-100.0.4.tsv - annotated-calls/varlociraptor-lancet/simulated-bwa.DEL.50-100.0.5.tsv - annotated-calls/varlociraptor-lancet/simulated-bwa.DEL.50-100.0.6.tsv - annotated-calls/varlociraptor-lancet/simulated-bwa.DEL.50-100.0.7.tsv - annotated-calls/varlociraptor-lancet/simulated-bwa.DEL.50-100.0.8.tsv - annotated-calls/varlociraptor-lancet/simulated-bwa.DEL.50-100.0.9.tsv - annotated-calls/varlociraptor-lancet/simulated-bwa.DEL.100-250.1e-05.tsv - annotated-calls/varlociraptor-lancet/simulated-bwa.DEL.100-250.0.0001.tsv - annotated-calls/varlociraptor-lancet/simulated-bwa.DEL.100-250.0.001.tsv - annotated-calls/varlociraptor-lancet/simulated-bwa.DEL.100-250.0.01.tsv - annotated-calls/varlociraptor-lancet/simulated-bwa.DEL.100-250.0.05.tsv - annotated-calls/varlociraptor-lancet/simulated-bwa.DEL.100-250.0.1.tsv - annotated-calls/varlociraptor-lancet/simulated-bwa.DEL.100-250.0.2.tsv - annotated-calls/varlociraptor-lancet/simulated-bwa.DEL.100-250.0.3.tsv - annotated-calls/varlociraptor-lancet/simulated-bwa.DEL.100-250.0.4.tsv - annotated-calls/varlociraptor-lancet/simulated-bwa.DEL.100-250.0.5.tsv - annotated-calls/varlociraptor-lancet/simulated-bwa.DEL.100-250.0.6.tsv - annotated-calls/varlociraptor-lancet/simulated-bwa.DEL.100-250.0.7.tsv - annotated-calls/varlociraptor-lancet/simulated-bwa.DEL.100-250.0.8.tsv - annotated-calls/varlociraptor-lancet/simulated-bwa.DEL.100-250.0.9.tsv - annotated-calls/varlociraptor-manta/simulated-bwa.DEL.1-30.1e-05.tsv - annotated-calls/varlociraptor-manta/simulated-bwa.DEL.1-30.0.0001.tsv - annotated-calls/varlociraptor-manta/simulated-bwa.DEL.1-30.0.001.tsv - annotated-calls/varlociraptor-manta/simulated-bwa.DEL.1-30.0.01.tsv - annotated-calls/varlociraptor-manta/simulated-bwa.DEL.1-30.0.05.tsv - annotated-calls/varlociraptor-manta/simulated-bwa.DEL.1-30.0.1.tsv - annotated-calls/varlociraptor-manta/simulated-bwa.DEL.1-30.0.2.tsv - annotated-calls/varlociraptor-manta/simulated-bwa.DEL.1-30.0.3.tsv - annotated-calls/varlociraptor-manta/simulated-bwa.DEL.1-30.0.4.tsv - annotated-calls/varlociraptor-manta/simulated-bwa.DEL.1-30.0.5.tsv - annotated-calls/varlociraptor-manta/simulated-bwa.DEL.1-30.0.6.tsv - annotated-calls/varlociraptor-manta/simulated-bwa.DEL.1-30.0.7.tsv - annotated-calls/varlociraptor-manta/simulated-bwa.DEL.1-30.0.8.tsv - annotated-calls/varlociraptor-manta/simulated-bwa.DEL.1-30.0.9.tsv - annotated-calls/varlociraptor-manta/simulated-bwa.DEL.30-50.1e-05.tsv - annotated-calls/varlociraptor-manta/simulated-bwa.DEL.30-50.0.0001.tsv - annotated-calls/varlociraptor-manta/simulated-bwa.DEL.30-50.0.001.tsv - annotated-calls/varlociraptor-manta/simulated-bwa.DEL.30-50.0.01.tsv - annotated-calls/varlociraptor-manta/simulated-bwa.DEL.30-50.0.05.tsv - annotated-calls/varlociraptor-manta/simulated-bwa.DEL.30-50.0.1.tsv - annotated-calls/varlociraptor-manta/simulated-bwa.DEL.30-50.0.2.tsv - annotated-calls/varlociraptor-manta/simulated-bwa.DEL.30-50.0.3.tsv - annotated-calls/varlociraptor-manta/simulated-bwa.DEL.30-50.0.4.tsv - annotated-calls/varlociraptor-manta/simulated-bwa.DEL.30-50.0.5.tsv - annotated-calls/varlociraptor-manta/simulated-bwa.DEL.30-50.0.6.tsv - annotated-calls/varlociraptor-manta/simulated-bwa.DEL.30-50.0.7.tsv - annotated-calls/varlociraptor-manta/simulated-bwa.DEL.30-50.0.8.tsv - annotated-calls/varlociraptor-manta/simulated-bwa.DEL.30-50.0.9.tsv - annotated-calls/varlociraptor-manta/simulated-bwa.DEL.50-100.1e-05.tsv - annotated-calls/varlociraptor-manta/simulated-bwa.DEL.50-100.0.0001.tsv - annotated-calls/varlociraptor-manta/simulated-bwa.DEL.50-100.0.001.tsv - annotated-calls/varlociraptor-manta/simulated-bwa.DEL.50-100.0.01.tsv - annotated-calls/varlociraptor-manta/simulated-bwa.DEL.50-100.0.05.tsv - annotated-calls/varlociraptor-manta/simulated-bwa.DEL.50-100.0.1.tsv - annotated-calls/varlociraptor-manta/simulated-bwa.DEL.50-100.0.2.tsv - annotated-calls/varlociraptor-manta/simulated-bwa.DEL.50-100.0.3.tsv - annotated-calls/varlociraptor-manta/simulated-bwa.DEL.50-100.0.4.tsv - annotated-calls/varlociraptor-manta/simulated-bwa.DEL.50-100.0.5.tsv - annotated-calls/varlociraptor-manta/simulated-bwa.DEL.50-100.0.6.tsv - annotated-calls/varlociraptor-manta/simulated-bwa.DEL.50-100.0.7.tsv - annotated-calls/varlociraptor-manta/simulated-bwa.DEL.50-100.0.8.tsv - annotated-calls/varlociraptor-manta/simulated-bwa.DEL.50-100.0.9.tsv - annotated-calls/varlociraptor-manta/simulated-bwa.DEL.100-250.1e-05.tsv - annotated-calls/varlociraptor-manta/simulated-bwa.DEL.100-250.0.0001.tsv - annotated-calls/varlociraptor-manta/simulated-bwa.DEL.100-250.0.001.tsv - annotated-calls/varlociraptor-manta/simulated-bwa.DEL.100-250.0.01.tsv - annotated-calls/varlociraptor-manta/simulated-bwa.DEL.100-250.0.05.tsv - annotated-calls/varlociraptor-manta/simulated-bwa.DEL.100-250.0.1.tsv - annotated-calls/varlociraptor-manta/simulated-bwa.DEL.100-250.0.2.tsv - annotated-calls/varlociraptor-manta/simulated-bwa.DEL.100-250.0.3.tsv - annotated-calls/varlociraptor-manta/simulated-bwa.DEL.100-250.0.4.tsv - annotated-calls/varlociraptor-manta/simulated-bwa.DEL.100-250.0.5.tsv - annotated-calls/varlociraptor-manta/simulated-bwa.DEL.100-250.0.6.tsv - annotated-calls/varlociraptor-manta/simulated-bwa.DEL.100-250.0.7.tsv - annotated-calls/varlociraptor-manta/simulated-bwa.DEL.100-250.0.8.tsv - annotated-calls/varlociraptor-manta/simulated-bwa.DEL.100-250.0.9.tsv - annotated-calls/varlociraptor-strelka/simulated-bwa.DEL.1-30.1e-05.tsv - annotated-calls/varlociraptor-strelka/simulated-bwa.DEL.1-30.0.0001.tsv - annotated-calls/varlociraptor-strelka/simulated-bwa.DEL.1-30.0.001.tsv - annotated-calls/varlociraptor-strelka/simulated-bwa.DEL.1-30.0.01.tsv - annotated-calls/varlociraptor-strelka/simulated-bwa.DEL.1-30.0.05.tsv - annotated-calls/varlociraptor-strelka/simulated-bwa.DEL.1-30.0.1.tsv - annotated-calls/varlociraptor-strelka/simulated-bwa.DEL.1-30.0.2.tsv - annotated-calls/varlociraptor-strelka/simulated-bwa.DEL.1-30.0.3.tsv - annotated-calls/varlociraptor-strelka/simulated-bwa.DEL.1-30.0.4.tsv - annotated-calls/varlociraptor-strelka/simulated-bwa.DEL.1-30.0.5.tsv - annotated-calls/varlociraptor-strelka/simulated-bwa.DEL.1-30.0.6.tsv - annotated-calls/varlociraptor-strelka/simulated-bwa.DEL.1-30.0.7.tsv - annotated-calls/varlociraptor-strelka/simulated-bwa.DEL.1-30.0.8.tsv - annotated-calls/varlociraptor-strelka/simulated-bwa.DEL.1-30.0.9.tsv - annotated-calls/varlociraptor-strelka/simulated-bwa.DEL.30-50.1e-05.tsv - annotated-calls/varlociraptor-strelka/simulated-bwa.DEL.30-50.0.0001.tsv - annotated-calls/varlociraptor-strelka/simulated-bwa.DEL.30-50.0.001.tsv - annotated-calls/varlociraptor-strelka/simulated-bwa.DEL.30-50.0.01.tsv - annotated-calls/varlociraptor-strelka/simulated-bwa.DEL.30-50.0.05.tsv - annotated-calls/varlociraptor-strelka/simulated-bwa.DEL.30-50.0.1.tsv - annotated-calls/varlociraptor-strelka/simulated-bwa.DEL.30-50.0.2.tsv - annotated-calls/varlociraptor-strelka/simulated-bwa.DEL.30-50.0.3.tsv - annotated-calls/varlociraptor-strelka/simulated-bwa.DEL.30-50.0.4.tsv - annotated-calls/varlociraptor-strelka/simulated-bwa.DEL.30-50.0.5.tsv - annotated-calls/varlociraptor-strelka/simulated-bwa.DEL.30-50.0.6.tsv - annotated-calls/varlociraptor-strelka/simulated-bwa.DEL.30-50.0.7.tsv - annotated-calls/varlociraptor-strelka/simulated-bwa.DEL.30-50.0.8.tsv - annotated-calls/varlociraptor-strelka/simulated-bwa.DEL.30-50.0.9.tsv - annotated-calls/varlociraptor-strelka/simulated-bwa.DEL.50-100.1e-05.tsv - annotated-calls/varlociraptor-strelka/simulated-bwa.DEL.50-100.0.0001.tsv - annotated-calls/varlociraptor-strelka/simulated-bwa.DEL.50-100.0.001.tsv - annotated-calls/varlociraptor-strelka/simulated-bwa.DEL.50-100.0.01.tsv - annotated-calls/varlociraptor-strelka/simulated-bwa.DEL.50-100.0.05.tsv - annotated-calls/varlociraptor-strelka/simulated-bwa.DEL.50-100.0.1.tsv - annotated-calls/varlociraptor-strelka/simulated-bwa.DEL.50-100.0.2.tsv - annotated-calls/varlociraptor-strelka/simulated-bwa.DEL.50-100.0.3.tsv - annotated-calls/varlociraptor-strelka/simulated-bwa.DEL.50-100.0.4.tsv - annotated-calls/varlociraptor-strelka/simulated-bwa.DEL.50-100.0.5.tsv - annotated-calls/varlociraptor-strelka/simulated-bwa.DEL.50-100.0.6.tsv - annotated-calls/varlociraptor-strelka/simulated-bwa.DEL.50-100.0.7.tsv - annotated-calls/varlociraptor-strelka/simulated-bwa.DEL.50-100.0.8.tsv - annotated-calls/varlociraptor-strelka/simulated-bwa.DEL.50-100.0.9.tsv - annotated-calls/varlociraptor-strelka/simulated-bwa.DEL.100-250.1e-05.tsv - annotated-calls/varlociraptor-strelka/simulated-bwa.DEL.100-250.0.0001.tsv - annotated-calls/varlociraptor-strelka/simulated-bwa.DEL.100-250.0.001.tsv - annotated-calls/varlociraptor-strelka/simulated-bwa.DEL.100-250.0.01.tsv - annotated-calls/varlociraptor-strelka/simulated-bwa.DEL.100-250.0.05.tsv - annotated-calls/varlociraptor-strelka/simulated-bwa.DEL.100-250.0.1.tsv - annotated-calls/varlociraptor-strelka/simulated-bwa.DEL.100-250.0.2.tsv - annotated-calls/varlociraptor-strelka/simulated-bwa.DEL.100-250.0.3.tsv - annotated-calls/varlociraptor-strelka/simulated-bwa.DEL.100-250.0.4.tsv - annotated-calls/varlociraptor-strelka/simulated-bwa.DEL.100-250.0.5.tsv - annotated-calls/varlociraptor-strelka/simulated-bwa.DEL.100-250.0.6.tsv - annotated-calls/varlociraptor-strelka/simulated-bwa.DEL.100-250.0.7.tsv - annotated-calls/varlociraptor-strelka/simulated-bwa.DEL.100-250.0.8.tsv - annotated-calls/varlociraptor-strelka/simulated-bwa.DEL.100-250.0.9.tsv - annotated-calls/varlociraptor-neusomatic/simulated-bwa.INS.1-250.1.0.tsv - annotated-calls/varlociraptor-delly/simulated-bwa.INS.1-250.1.0.tsv - annotated-calls/varlociraptor-lancet/simulated-bwa.INS.1-250.1.0.tsv - annotated-calls/varlociraptor-manta/simulated-bwa.INS.1-250.1.0.tsv - annotated-calls/varlociraptor-strelka/simulated-bwa.INS.1-250.1.0.tsv - annotated-calls/varlociraptor-neusomatic/simulated-bwa.DEL.1-250.1.0.tsv - annotated-calls/varlociraptor-delly/simulated-bwa.DEL.1-250.1.0.tsv - annotated-calls/varlociraptor-lancet/simulated-bwa.DEL.1-250.1.0.tsv - annotated-calls/varlociraptor-manta/simulated-bwa.DEL.1-250.1.0.tsv - annotated-calls/varlociraptor-strelka/simulated-bwa.DEL.1-250.1.0.tsv |  | - python =3.6 - pandas =0.23 - matplotlib =3.0 - seaborn =0.9.0 - pysam =0.13.0 - svgutils =0.2 - pybedtools =0.7.10 - networkx =2.2 | |  |  | | --- | --- | | ```  1  2  3  4  5  6  7  8  9 10 11 12 13 14 15 16 17 18 19 20 21 22 23 24 25 26 ``` | ``` import pandas as pd import numpy as np from common import load_variants   minlen = int(snakemake.wildcards.minlen) maxlen = int(snakemake.wildcards.maxlen) vartype = snakemake.wildcards.vartype  if snakemake.wildcards.mode == "varlociraptor":     score = snakemake.config["caller"]["varlociraptor"]["score"]     # calls are already filtered by FDR control step     minlen = None     maxlen = None elif snakemake.wildcards.mode == "default":     score = snakemake.config["caller"][snakemake.wildcards.caller]["score"] else:     score = None  calls = load_variants(snakemake.input.calls, vartype=vartype, minlen=minlen, maxlen=maxlen)  calls["is_tp"] = calls["MATCHING"] >= 0  calls["score"] = calls[score] if score else np.nan  calls.to_csv(snakemake.output[0], sep="\t") ``` | |
| truth\_to\_tsv | 2 | - truth/synthetic-5.annotated.tsv - truth/synthetic-20.annotated.tsv |  | - rust-bio-tools =0.9.0 - bedtools =2.27.1 - bcftools =1.8 | |  |  | | --- | --- | | ``` 1 ``` | ``` rbt vcf-to-txt --genotypes --info SOMATIC SVLEN SVTYPE TAF NAF < {input} > {output} ``` | |
| plot\_concordance | 2 | - plots/concordance/colo1.INS.concordance.svg - plots/concordance/colo1.DEL.concordance.svg |  | - python =3.6 - pandas =0.23 - matplotlib =3.0 - seaborn =0.9.0 - pysam =0.13.0 - svgutils =0.2 - pybedtools =0.7.10 - networkx =2.2 | |  |  | | --- | --- | | ```   1   2   3   4   5   6   7   8   9  10  11  12  13  14  15  16  17  18  19  20  21  22  23  24  25  26  27  28  29  30  31  32  33  34  35  36  37  38  39  40  41  42  43  44  45  46  47  48  49  50  51  52  53  54  55  56  57  58  59  60  61  62  63  64  65  66  67  68  69  70  71  72  73  74  75  76  77  78  79  80  81  82  83  84  85  86  87  88  89  90  91  92  93  94  95  96  97  98  99 100 101 102 103 104 105 106 107 108 109 110 111 112 113 114 115 116 117 118 119 120 121 122 123 124 125 126 127 ``` | ``` from itertools import product import matplotlib matplotlib.use("agg") from matplotlib import pyplot as plt import seaborn as sns import pandas as pd import common import numpy as np import math from matplotlib.lines import Line2D from matplotlib.colors import to_rgba   class NotEnoughObservationsException(Exception):     pass   MIN_CALLS = 20 MAX_LEN = 1000  vartype = snakemake.wildcards.vartype colors = common.get_colors(snakemake.config)   varlociraptor_calls_low = [pd.read_table(f) for f in snakemake.input.varlociraptor_calls_low] varlociraptor_calls_high = [pd.read_table(f) for f in snakemake.input.varlociraptor_calls_high] adhoc_calls = [pd.read_table(f) for f in snakemake.input.adhoc_calls]   def expected_count(af, effective_mutation_rate):     """Calculate the expected number of somatic variants        greater than a given allele frequency given an effective mutation        rate, according to the model of Williams et al. Nature         Genetics 2016"""     return effective_mutation_rate * (1.0 / af - 1.0)   def expected_counts(afs, effective_mutation_rate):     return [expected_count(af, effective_mutation_rate) for af in afs]   def calc_concordance(calls):     n = len(calls)     return (calls["concordance_count"] > 1).sum() / n   def plot_len_range(minlen, maxlen, yfunc=None, yscale=None, upper_bound=None):     handles_varlociraptor = []     handles_adhoc = []     for i, caller in enumerate(snakemake.params.callers):         def plot_calls(calls, label, color, style, calls_lower=None):             def get_xy(calls, caseafs=None):                 svlen = calls.loc[:, calls.columns.str.startswith("SVLEN")].abs()                 # at least one of the calls has a valid svlen                 valid = ((svlen >= minlen) & (svlen <= maxlen)).sum(axis=1) >= 1                 calls = calls[valid]                 if caseafs is None:                     caseafs = calls["max_case_af"].dropna().unique()                 y = []                 _caseafs = []                 for caseaf in sorted(caseafs):                     _calls = calls[calls["max_case_af"] >= caseaf]                     if upper_bound is not None:                         _calls = _calls[_calls["max_case_af"] <= caseaf + upper_bound]                     if len(_calls) < MIN_CALLS:                         continue                     _caseafs.append(caseaf)                     y.append(yfunc(_calls))                 return _caseafs, y              x, y = get_xy(calls)             if not x:                 raise NotEnoughObservationsException()             if calls_lower is not None:                 _, y2 = get_xy(calls_lower, caseafs=x)                 return plt.fill_between(x, y, y2, label=label, edgecolor=color, facecolor=to_rgba(color, alpha=0.2))             else:                 if style != "-":                     plt.plot(x, y, "-", color="white", alpha=0.8)                 return plt.plot(x, y, style, label=label, color=color)[0]          color = colors[snakemake.params.callers[i]]         try:             handles_varlociraptor.append(                 plot_calls(                     varlociraptor_calls_high[i],                      "varlociraptor+{}".format(caller),                      color=color, style="-",                      calls_lower=varlociraptor_calls_low[i]))         except NotEnoughObservationsException:             # skip plot             pass         try:             handles_adhoc.append(plot_calls(adhoc_calls[i], caller, color=color, style=":"))         except NotEnoughObservationsException:             # skip plot             pass      handles = handles_varlociraptor + handles_adhoc     sns.despine()     ax = plt.gca()     if yscale is not None:         ax.set_yscale(yscale)     return ax, handles  plt.figure(figsize=(10, 4)) plt.subplot(121) plot_len_range(1, MAX_LEN, yfunc=calc_concordance) plt.xlabel("$\geq$ tumor allele frequency") plt.ylabel("concordance")  plt.subplot(122) for effective_mutation_rate in 10 ** np.linspace(1, 5, 7):     afs = np.linspace(0.0, 1.0, 100, endpoint=False)     plt.semilogy(afs, expected_counts(afs, effective_mutation_rate), "-", color="grey", alpha=0.4)  ax, handles = plot_len_range(1, MAX_LEN, yfunc=lambda calls: len(calls), yscale="log")  plt.xlabel("$\geq$ tumor allele frequency") plt.ylabel("# of calls")  ax.legend(handles=handles, loc="upper left", bbox_to_anchor=(1.0, 1.0))  plt.tight_layout()  plt.savefig(snakemake.output[0], bbox_inches="tight") ``` | |
| varlociraptor\_calls\_to\_tsv | 122 | - matched-calls/varlociraptor-neusomatic/simulated-bwa.INS.1-30.1.0.tsv - matched-calls/varlociraptor-neusomatic/simulated-bwa.INS.30-100.1.0.tsv - matched-calls/varlociraptor-neusomatic/synthetic-5.INS.1-250.1.0.tsv - matched-calls/varlociraptor-delly/synthetic-5.INS.1-250.1.0.tsv - matched-calls/varlociraptor-lancet/synthetic-5.INS.1-250.1.0.tsv - matched-calls/varlociraptor-manta/synthetic-5.INS.1-250.1.0.tsv - matched-calls/varlociraptor-strelka/synthetic-5.INS.1-250.1.0.tsv - matched-calls/varlociraptor-neusomatic/synthetic-20.INS.1-30.1.0.tsv - matched-calls/varlociraptor-neusomatic/synthetic-20.INS.30-250.1.0.tsv - matched-calls/varlociraptor-delly/synthetic-20.INS.1-30.1.0.tsv - matched-calls/varlociraptor-delly/synthetic-20.INS.30-250.1.0.tsv - matched-calls/varlociraptor-lancet/synthetic-20.INS.1-30.1.0.tsv - matched-calls/varlociraptor-lancet/synthetic-20.INS.30-250.1.0.tsv - matched-calls/varlociraptor-manta/synthetic-20.INS.1-30.1.0.tsv - matched-calls/varlociraptor-manta/synthetic-20.INS.30-250.1.0.tsv - matched-calls/varlociraptor-strelka/synthetic-20.INS.1-30.1.0.tsv - matched-calls/varlociraptor-strelka/synthetic-20.INS.30-250.1.0.tsv - matched-calls/varlociraptor-neusomatic/simulated-bwa.DEL.1-30.1.0.tsv - matched-calls/varlociraptor-neusomatic/simulated-bwa.DEL.30-50.1.0.tsv - matched-calls/varlociraptor-neusomatic/simulated-bwa.DEL.50-100.1.0.tsv - matched-calls/varlociraptor-neusomatic/simulated-bwa.DEL.100-250.1.0.tsv - matched-calls/varlociraptor-neusomatic/synthetic-5.DEL.1-250.1.0.tsv - matched-calls/varlociraptor-delly/synthetic-5.DEL.1-250.1.0.tsv - matched-calls/varlociraptor-lancet/synthetic-5.DEL.1-250.1.0.tsv - matched-calls/varlociraptor-manta/synthetic-5.DEL.1-250.1.0.tsv - matched-calls/varlociraptor-strelka/synthetic-5.DEL.1-250.1.0.tsv - matched-calls/varlociraptor-neusomatic/synthetic-20.DEL.1-30.1.0.tsv - matched-calls/varlociraptor-neusomatic/synthetic-20.DEL.30-250.1.0.tsv - matched-calls/varlociraptor-delly/synthetic-20.DEL.1-30.1.0.tsv - matched-calls/varlociraptor-delly/synthetic-20.DEL.30-250.1.0.tsv - matched-calls/varlociraptor-lancet/synthetic-20.DEL.1-30.1.0.tsv - matched-calls/varlociraptor-lancet/synthetic-20.DEL.30-250.1.0.tsv - matched-calls/varlociraptor-manta/synthetic-20.DEL.1-30.1.0.tsv - matched-calls/varlociraptor-manta/synthetic-20.DEL.30-250.1.0.tsv - matched-calls/varlociraptor-strelka/synthetic-20.DEL.1-30.1.0.tsv - matched-calls/varlociraptor-strelka/synthetic-20.DEL.30-250.1.0.tsv - matched-calls/varlociraptor-neusomatic/simulated-bwa.INS.1-30.1e-05.tsv - matched-calls/varlociraptor-neusomatic/simulated-bwa.INS.1-30.0.0001.tsv - matched-calls/varlociraptor-neusomatic/simulated-bwa.INS.1-30.0.001.tsv - matched-calls/varlociraptor-neusomatic/simulated-bwa.INS.1-30.0.01.tsv - matched-calls/varlociraptor-neusomatic/simulated-bwa.INS.1-30.0.05.tsv - matched-calls/varlociraptor-neusomatic/simulated-bwa.INS.1-30.0.1.tsv - matched-calls/varlociraptor-neusomatic/simulated-bwa.INS.1-30.0.2.tsv - matched-calls/varlociraptor-neusomatic/simulated-bwa.INS.1-30.0.3.tsv - matched-calls/varlociraptor-neusomatic/simulated-bwa.INS.1-30.0.4.tsv - matched-calls/varlociraptor-neusomatic/simulated-bwa.INS.1-30.0.5.tsv - matched-calls/varlociraptor-neusomatic/simulated-bwa.INS.1-30.0.6.tsv - matched-calls/varlociraptor-neusomatic/simulated-bwa.INS.1-30.0.7.tsv - matched-calls/varlociraptor-neusomatic/simulated-bwa.INS.1-30.0.8.tsv - matched-calls/varlociraptor-neusomatic/simulated-bwa.INS.1-30.0.9.tsv - matched-calls/varlociraptor-neusomatic/simulated-bwa.INS.30-100.1e-05.tsv - matched-calls/varlociraptor-neusomatic/simulated-bwa.INS.30-100.0.0001.tsv - matched-calls/varlociraptor-neusomatic/simulated-bwa.INS.30-100.0.001.tsv - matched-calls/varlociraptor-neusomatic/simulated-bwa.INS.30-100.0.01.tsv - matched-calls/varlociraptor-neusomatic/simulated-bwa.INS.30-100.0.05.tsv - matched-calls/varlociraptor-neusomatic/simulated-bwa.INS.30-100.0.1.tsv - matched-calls/varlociraptor-neusomatic/simulated-bwa.INS.30-100.0.2.tsv - matched-calls/varlociraptor-neusomatic/simulated-bwa.INS.30-100.0.3.tsv - matched-calls/varlociraptor-neusomatic/simulated-bwa.INS.30-100.0.4.tsv - matched-calls/varlociraptor-neusomatic/simulated-bwa.INS.30-100.0.5.tsv - matched-calls/varlociraptor-neusomatic/simulated-bwa.INS.30-100.0.6.tsv - matched-calls/varlociraptor-neusomatic/simulated-bwa.INS.30-100.0.7.tsv - matched-calls/varlociraptor-neusomatic/simulated-bwa.INS.30-100.0.8.tsv - matched-calls/varlociraptor-neusomatic/simulated-bwa.INS.30-100.0.9.tsv - matched-calls/varlociraptor-neusomatic/simulated-bwa.DEL.1-30.1e-05.tsv - matched-calls/varlociraptor-neusomatic/simulated-bwa.DEL.1-30.0.0001.tsv - matched-calls/varlociraptor-neusomatic/simulated-bwa.DEL.1-30.0.001.tsv - matched-calls/varlociraptor-neusomatic/simulated-bwa.DEL.1-30.0.01.tsv - matched-calls/varlociraptor-neusomatic/simulated-bwa.DEL.1-30.0.05.tsv - matched-calls/varlociraptor-neusomatic/simulated-bwa.DEL.1-30.0.1.tsv - matched-calls/varlociraptor-neusomatic/simulated-bwa.DEL.1-30.0.2.tsv - matched-calls/varlociraptor-neusomatic/simulated-bwa.DEL.1-30.0.3.tsv - matched-calls/varlociraptor-neusomatic/simulated-bwa.DEL.1-30.0.4.tsv - matched-calls/varlociraptor-neusomatic/simulated-bwa.DEL.1-30.0.5.tsv - matched-calls/varlociraptor-neusomatic/simulated-bwa.DEL.1-30.0.6.tsv - matched-calls/varlociraptor-neusomatic/simulated-bwa.DEL.1-30.0.7.tsv - matched-calls/varlociraptor-neusomatic/simulated-bwa.DEL.1-30.0.8.tsv - matched-calls/varlociraptor-neusomatic/simulated-bwa.DEL.1-30.0.9.tsv - matched-calls/varlociraptor-neusomatic/simulated-bwa.DEL.30-50.1e-05.tsv - matched-calls/varlociraptor-neusomatic/simulated-bwa.DEL.30-50.0.0001.tsv - matched-calls/varlociraptor-neusomatic/simulated-bwa.DEL.30-50.0.001.tsv - matched-calls/varlociraptor-neusomatic/simulated-bwa.DEL.30-50.0.01.tsv - matched-calls/varlociraptor-neusomatic/simulated-bwa.DEL.30-50.0.05.tsv - matched-calls/varlociraptor-neusomatic/simulated-bwa.DEL.30-50.0.1.tsv - matched-calls/varlociraptor-neusomatic/simulated-bwa.DEL.30-50.0.2.tsv - matched-calls/varlociraptor-neusomatic/simulated-bwa.DEL.30-50.0.3.tsv - matched-calls/varlociraptor-neusomatic/simulated-bwa.DEL.30-50.0.4.tsv - matched-calls/varlociraptor-neusomatic/simulated-bwa.DEL.30-50.0.5.tsv - matched-calls/varlociraptor-neusomatic/simulated-bwa.DEL.30-50.0.6.tsv - matched-calls/varlociraptor-neusomatic/simulated-bwa.DEL.30-50.0.7.tsv - matched-calls/varlociraptor-neusomatic/simulated-bwa.DEL.30-50.0.8.tsv - matched-calls/varlociraptor-neusomatic/simulated-bwa.DEL.30-50.0.9.tsv - matched-calls/varlociraptor-neusomatic/simulated-bwa.DEL.50-100.1e-05.tsv - matched-calls/varlociraptor-neusomatic/simulated-bwa.DEL.50-100.0.0001.tsv - matched-calls/varlociraptor-neusomatic/simulated-bwa.DEL.50-100.0.001.tsv - matched-calls/varlociraptor-neusomatic/simulated-bwa.DEL.50-100.0.01.tsv - matched-calls/varlociraptor-neusomatic/simulated-bwa.DEL.50-100.0.05.tsv - matched-calls/varlociraptor-neusomatic/simulated-bwa.DEL.50-100.0.1.tsv - matched-calls/varlociraptor-neusomatic/simulated-bwa.DEL.50-100.0.2.tsv - matched-calls/varlociraptor-neusomatic/simulated-bwa.DEL.50-100.0.3.tsv - matched-calls/varlociraptor-neusomatic/simulated-bwa.DEL.50-100.0.4.tsv - matched-calls/varlociraptor-neusomatic/simulated-bwa.DEL.50-100.0.5.tsv - matched-calls/varlociraptor-neusomatic/simulated-bwa.DEL.50-100.0.6.tsv - matched-calls/varlociraptor-neusomatic/simulated-bwa.DEL.50-100.0.7.tsv - matched-calls/varlociraptor-neusomatic/simulated-bwa.DEL.50-100.0.8.tsv - matched-calls/varlociraptor-neusomatic/simulated-bwa.DEL.50-100.0.9.tsv - matched-calls/varlociraptor-neusomatic/simulated-bwa.DEL.100-250.1e-05.tsv - matched-calls/varlociraptor-neusomatic/simulated-bwa.DEL.100-250.0.0001.tsv - matched-calls/varlociraptor-neusomatic/simulated-bwa.DEL.100-250.0.001.tsv - matched-calls/varlociraptor-neusomatic/simulated-bwa.DEL.100-250.0.01.tsv - matched-calls/varlociraptor-neusomatic/simulated-bwa.DEL.100-250.0.05.tsv - matched-calls/varlociraptor-neusomatic/simulated-bwa.DEL.100-250.0.1.tsv - matched-calls/varlociraptor-neusomatic/simulated-bwa.DEL.100-250.0.2.tsv - matched-calls/varlociraptor-neusomatic/simulated-bwa.DEL.100-250.0.3.tsv - matched-calls/varlociraptor-neusomatic/simulated-bwa.DEL.100-250.0.4.tsv - matched-calls/varlociraptor-neusomatic/simulated-bwa.DEL.100-250.0.5.tsv - matched-calls/varlociraptor-neusomatic/simulated-bwa.DEL.100-250.0.6.tsv - matched-calls/varlociraptor-neusomatic/simulated-bwa.DEL.100-250.0.7.tsv - matched-calls/varlociraptor-neusomatic/simulated-bwa.DEL.100-250.0.8.tsv - matched-calls/varlociraptor-neusomatic/simulated-bwa.DEL.100-250.0.9.tsv - matched-calls/varlociraptor-neusomatic/simulated-bwa.INS.1-250.1.0.tsv - matched-calls/varlociraptor-neusomatic/simulated-bwa.DEL.1-250.1.0.tsv |  | - rust-bio-tools =0.9.0 - bedtools =2.27.1 - bcftools =1.8 | |  |  | | --- | --- | | ``` 1 ``` | ``` rbt vcf-to-txt {params.gt} {params.tags} --info MATCHING < {input} > {output} ``` | |
| varlociraptor\_calls\_to\_tsv | 368 | - matched-calls/varlociraptor-delly/simulated-bwa.INS.1-30.1.0.tsv - matched-calls/varlociraptor-delly/simulated-bwa.INS.30-100.1.0.tsv - matched-calls/varlociraptor-lancet/simulated-bwa.INS.1-30.1.0.tsv - matched-calls/varlociraptor-lancet/simulated-bwa.INS.30-100.1.0.tsv - matched-calls/varlociraptor-manta/simulated-bwa.INS.1-30.1.0.tsv - matched-calls/varlociraptor-manta/simulated-bwa.INS.30-100.1.0.tsv - matched-calls/varlociraptor-strelka/simulated-bwa.INS.1-30.1.0.tsv - matched-calls/varlociraptor-strelka/simulated-bwa.INS.30-100.1.0.tsv - matched-calls/varlociraptor-delly/simulated-bwa.DEL.1-30.1.0.tsv - matched-calls/varlociraptor-delly/simulated-bwa.DEL.30-50.1.0.tsv - matched-calls/varlociraptor-delly/simulated-bwa.DEL.50-100.1.0.tsv - matched-calls/varlociraptor-delly/simulated-bwa.DEL.100-250.1.0.tsv - matched-calls/varlociraptor-lancet/simulated-bwa.DEL.1-30.1.0.tsv - matched-calls/varlociraptor-lancet/simulated-bwa.DEL.30-50.1.0.tsv - matched-calls/varlociraptor-lancet/simulated-bwa.DEL.50-100.1.0.tsv - matched-calls/varlociraptor-lancet/simulated-bwa.DEL.100-250.1.0.tsv - matched-calls/varlociraptor-manta/simulated-bwa.DEL.1-30.1.0.tsv - matched-calls/varlociraptor-manta/simulated-bwa.DEL.30-50.1.0.tsv - matched-calls/varlociraptor-manta/simulated-bwa.DEL.50-100.1.0.tsv - matched-calls/varlociraptor-manta/simulated-bwa.DEL.100-250.1.0.tsv - matched-calls/varlociraptor-strelka/simulated-bwa.DEL.1-30.1.0.tsv - matched-calls/varlociraptor-strelka/simulated-bwa.DEL.30-50.1.0.tsv - matched-calls/varlociraptor-strelka/simulated-bwa.DEL.50-100.1.0.tsv - matched-calls/varlociraptor-strelka/simulated-bwa.DEL.100-250.1.0.tsv - matched-calls/varlociraptor-delly/simulated-bwa.INS.1-30.1e-05.tsv - matched-calls/varlociraptor-delly/simulated-bwa.INS.1-30.0.0001.tsv - matched-calls/varlociraptor-delly/simulated-bwa.INS.1-30.0.001.tsv - matched-calls/varlociraptor-delly/simulated-bwa.INS.1-30.0.01.tsv - matched-calls/varlociraptor-delly/simulated-bwa.INS.1-30.0.05.tsv - matched-calls/varlociraptor-delly/simulated-bwa.INS.1-30.0.1.tsv - matched-calls/varlociraptor-delly/simulated-bwa.INS.1-30.0.2.tsv - matched-calls/varlociraptor-delly/simulated-bwa.INS.1-30.0.3.tsv - matched-calls/varlociraptor-delly/simulated-bwa.INS.1-30.0.4.tsv - matched-calls/varlociraptor-delly/simulated-bwa.INS.1-30.0.5.tsv - matched-calls/varlociraptor-delly/simulated-bwa.INS.1-30.0.6.tsv - matched-calls/varlociraptor-delly/simulated-bwa.INS.1-30.0.7.tsv - matched-calls/varlociraptor-delly/simulated-bwa.INS.1-30.0.8.tsv - matched-calls/varlociraptor-delly/simulated-bwa.INS.1-30.0.9.tsv - matched-calls/varlociraptor-delly/simulated-bwa.INS.30-100.1e-05.tsv - matched-calls/varlociraptor-delly/simulated-bwa.INS.30-100.0.0001.tsv - matched-calls/varlociraptor-delly/simulated-bwa.INS.30-100.0.001.tsv - matched-calls/varlociraptor-delly/simulated-bwa.INS.30-100.0.01.tsv - matched-calls/varlociraptor-delly/simulated-bwa.INS.30-100.0.05.tsv - matched-calls/varlociraptor-delly/simulated-bwa.INS.30-100.0.1.tsv - matched-calls/varlociraptor-delly/simulated-bwa.INS.30-100.0.2.tsv - matched-calls/varlociraptor-delly/simulated-bwa.INS.30-100.0.3.tsv - matched-calls/varlociraptor-delly/simulated-bwa.INS.30-100.0.4.tsv - matched-calls/varlociraptor-delly/simulated-bwa.INS.30-100.0.5.tsv - matched-calls/varlociraptor-delly/simulated-bwa.INS.30-100.0.6.tsv - matched-calls/varlociraptor-delly/simulated-bwa.INS.30-100.0.7.tsv - matched-calls/varlociraptor-delly/simulated-bwa.INS.30-100.0.8.tsv - matched-calls/varlociraptor-delly/simulated-bwa.INS.30-100.0.9.tsv - matched-calls/varlociraptor-lancet/simulated-bwa.INS.1-30.1e-05.tsv - matched-calls/varlociraptor-lancet/simulated-bwa.INS.1-30.0.0001.tsv - matched-calls/varlociraptor-lancet/simulated-bwa.INS.1-30.0.001.tsv - matched-calls/varlociraptor-lancet/simulated-bwa.INS.1-30.0.01.tsv - matched-calls/varlociraptor-lancet/simulated-bwa.INS.1-30.0.05.tsv - matched-calls/varlociraptor-lancet/simulated-bwa.INS.1-30.0.1.tsv - matched-calls/varlociraptor-lancet/simulated-bwa.INS.1-30.0.2.tsv - matched-calls/varlociraptor-lancet/simulated-bwa.INS.1-30.0.3.tsv - matched-calls/varlociraptor-lancet/simulated-bwa.INS.1-30.0.4.tsv - matched-calls/varlociraptor-lancet/simulated-bwa.INS.1-30.0.5.tsv - matched-calls/varlociraptor-lancet/simulated-bwa.INS.1-30.0.6.tsv - matched-calls/varlociraptor-lancet/simulated-bwa.INS.1-30.0.7.tsv - matched-calls/varlociraptor-lancet/simulated-bwa.INS.1-30.0.8.tsv - matched-calls/varlociraptor-lancet/simulated-bwa.INS.1-30.0.9.tsv - matched-calls/varlociraptor-lancet/simulated-bwa.INS.30-100.1e-05.tsv - matched-calls/varlociraptor-lancet/simulated-bwa.INS.30-100.0.0001.tsv - matched-calls/varlociraptor-lancet/simulated-bwa.INS.30-100.0.001.tsv - matched-calls/varlociraptor-lancet/simulated-bwa.INS.30-100.0.01.tsv - matched-calls/varlociraptor-lancet/simulated-bwa.INS.30-100.0.05.tsv - matched-calls/varlociraptor-lancet/simulated-bwa.INS.30-100.0.1.tsv - matched-calls/varlociraptor-lancet/simulated-bwa.INS.30-100.0.2.tsv - matched-calls/varlociraptor-lancet/simulated-bwa.INS.30-100.0.3.tsv - matched-calls/varlociraptor-lancet/simulated-bwa.INS.30-100.0.4.tsv - matched-calls/varlociraptor-lancet/simulated-bwa.INS.30-100.0.5.tsv - matched-calls/varlociraptor-lancet/simulated-bwa.INS.30-100.0.6.tsv - matched-calls/varlociraptor-lancet/simulated-bwa.INS.30-100.0.7.tsv - matched-calls/varlociraptor-lancet/simulated-bwa.INS.30-100.0.8.tsv - matched-calls/varlociraptor-lancet/simulated-bwa.INS.30-100.0.9.tsv - matched-calls/varlociraptor-manta/simulated-bwa.INS.1-30.1e-05.tsv - matched-calls/varlociraptor-manta/simulated-bwa.INS.1-30.0.0001.tsv - matched-calls/varlociraptor-manta/simulated-bwa.INS.1-30.0.001.tsv - matched-calls/varlociraptor-manta/simulated-bwa.INS.1-30.0.01.tsv - matched-calls/varlociraptor-manta/simulated-bwa.INS.1-30.0.05.tsv - matched-calls/varlociraptor-manta/simulated-bwa.INS.1-30.0.1.tsv - matched-calls/varlociraptor-manta/simulated-bwa.INS.1-30.0.2.tsv - matched-calls/varlociraptor-manta/simulated-bwa.INS.1-30.0.3.tsv - matched-calls/varlociraptor-manta/simulated-bwa.INS.1-30.0.4.tsv - matched-calls/varlociraptor-manta/simulated-bwa.INS.1-30.0.5.tsv - matched-calls/varlociraptor-manta/simulated-bwa.INS.1-30.0.6.tsv - matched-calls/varlociraptor-manta/simulated-bwa.INS.1-30.0.7.tsv - matched-calls/varlociraptor-manta/simulated-bwa.INS.1-30.0.8.tsv - matched-calls/varlociraptor-manta/simulated-bwa.INS.1-30.0.9.tsv - matched-calls/varlociraptor-manta/simulated-bwa.INS.30-100.1e-05.tsv - matched-calls/varlociraptor-manta/simulated-bwa.INS.30-100.0.0001.tsv - matched-calls/varlociraptor-manta/simulated-bwa.INS.30-100.0.001.tsv - matched-calls/varlociraptor-manta/simulated-bwa.INS.30-100.0.01.tsv - matched-calls/varlociraptor-manta/simulated-bwa.INS.30-100.0.05.tsv - matched-calls/varlociraptor-manta/simulated-bwa.INS.30-100.0.1.tsv - matched-calls/varlociraptor-manta/simulated-bwa.INS.30-100.0.2.tsv - matched-calls/varlociraptor-manta/simulated-bwa.INS.30-100.0.3.tsv - matched-calls/varlociraptor-manta/simulated-bwa.INS.30-100.0.4.tsv - matched-calls/varlociraptor-manta/simulated-bwa.INS.30-100.0.5.tsv - matched-calls/varlociraptor-manta/simulated-bwa.INS.30-100.0.6.tsv - matched-calls/varlociraptor-manta/simulated-bwa.INS.30-100.0.7.tsv - matched-calls/varlociraptor-manta/simulated-bwa.INS.30-100.0.8.tsv - matched-calls/varlociraptor-manta/simulated-bwa.INS.30-100.0.9.tsv - matched-calls/varlociraptor-strelka/simulated-bwa.INS.1-30.1e-05.tsv - matched-calls/varlociraptor-strelka/simulated-bwa.INS.1-30.0.0001.tsv - matched-calls/varlociraptor-strelka/simulated-bwa.INS.1-30.0.001.tsv - matched-calls/varlociraptor-strelka/simulated-bwa.INS.1-30.0.01.tsv - matched-calls/varlociraptor-strelka/simulated-bwa.INS.1-30.0.05.tsv - matched-calls/varlociraptor-strelka/simulated-bwa.INS.1-30.0.1.tsv - matched-calls/varlociraptor-strelka/simulated-bwa.INS.1-30.0.2.tsv - matched-calls/varlociraptor-strelka/simulated-bwa.INS.1-30.0.3.tsv - matched-calls/varlociraptor-strelka/simulated-bwa.INS.1-30.0.4.tsv - matched-calls/varlociraptor-strelka/simulated-bwa.INS.1-30.0.5.tsv - matched-calls/varlociraptor-strelka/simulated-bwa.INS.1-30.0.6.tsv - matched-calls/varlociraptor-strelka/simulated-bwa.INS.1-30.0.7.tsv - matched-calls/varlociraptor-strelka/simulated-bwa.INS.1-30.0.8.tsv - matched-calls/varlociraptor-strelka/simulated-bwa.INS.1-30.0.9.tsv - matched-calls/varlociraptor-strelka/simulated-bwa.INS.30-100.1e-05.tsv - matched-calls/varlociraptor-strelka/simulated-bwa.INS.30-100.0.0001.tsv - matched-calls/varlociraptor-strelka/simulated-bwa.INS.30-100.0.001.tsv - matched-calls/varlociraptor-strelka/simulated-bwa.INS.30-100.0.01.tsv - matched-calls/varlociraptor-strelka/simulated-bwa.INS.30-100.0.05.tsv - matched-calls/varlociraptor-strelka/simulated-bwa.INS.30-100.0.1.tsv - matched-calls/varlociraptor-strelka/simulated-bwa.INS.30-100.0.2.tsv - matched-calls/varlociraptor-strelka/simulated-bwa.INS.30-100.0.3.tsv - matched-calls/varlociraptor-strelka/simulated-bwa.INS.30-100.0.4.tsv - matched-calls/varlociraptor-strelka/simulated-bwa.INS.30-100.0.5.tsv - matched-calls/varlociraptor-strelka/simulated-bwa.INS.30-100.0.6.tsv - matched-calls/varlociraptor-strelka/simulated-bwa.INS.30-100.0.7.tsv - matched-calls/varlociraptor-strelka/simulated-bwa.INS.30-100.0.8.tsv - matched-calls/varlociraptor-strelka/simulated-bwa.INS.30-100.0.9.tsv - matched-calls/varlociraptor-delly/simulated-bwa.DEL.1-30.1e-05.tsv - matched-calls/varlociraptor-delly/simulated-bwa.DEL.1-30.0.0001.tsv - matched-calls/varlociraptor-delly/simulated-bwa.DEL.1-30.0.001.tsv - matched-calls/varlociraptor-delly/simulated-bwa.DEL.1-30.0.01.tsv - matched-calls/varlociraptor-delly/simulated-bwa.DEL.1-30.0.05.tsv - matched-calls/varlociraptor-delly/simulated-bwa.DEL.1-30.0.1.tsv - matched-calls/varlociraptor-delly/simulated-bwa.DEL.1-30.0.2.tsv - matched-calls/varlociraptor-delly/simulated-bwa.DEL.1-30.0.3.tsv - matched-calls/varlociraptor-delly/simulated-bwa.DEL.1-30.0.4.tsv - matched-calls/varlociraptor-delly/simulated-bwa.DEL.1-30.0.5.tsv - matched-calls/varlociraptor-delly/simulated-bwa.DEL.1-30.0.6.tsv - matched-calls/varlociraptor-delly/simulated-bwa.DEL.1-30.0.7.tsv - matched-calls/varlociraptor-delly/simulated-bwa.DEL.1-30.0.8.tsv - matched-calls/varlociraptor-delly/simulated-bwa.DEL.1-30.0.9.tsv - matched-calls/varlociraptor-delly/simulated-bwa.DEL.30-50.1e-05.tsv - matched-calls/varlociraptor-delly/simulated-bwa.DEL.30-50.0.0001.tsv - matched-calls/varlociraptor-delly/simulated-bwa.DEL.30-50.0.001.tsv - matched-calls/varlociraptor-delly/simulated-bwa.DEL.30-50.0.01.tsv - matched-calls/varlociraptor-delly/simulated-bwa.DEL.30-50.0.05.tsv - matched-calls/varlociraptor-delly/simulated-bwa.DEL.30-50.0.1.tsv - matched-calls/varlociraptor-delly/simulated-bwa.DEL.30-50.0.2.tsv - matched-calls/varlociraptor-delly/simulated-bwa.DEL.30-50.0.3.tsv - matched-calls/varlociraptor-delly/simulated-bwa.DEL.30-50.0.4.tsv - matched-calls/varlociraptor-delly/simulated-bwa.DEL.30-50.0.5.tsv - matched-calls/varlociraptor-delly/simulated-bwa.DEL.30-50.0.6.tsv - matched-calls/varlociraptor-delly/simulated-bwa.DEL.30-50.0.7.tsv - matched-calls/varlociraptor-delly/simulated-bwa.DEL.30-50.0.8.tsv - matched-calls/varlociraptor-delly/simulated-bwa.DEL.30-50.0.9.tsv - matched-calls/varlociraptor-delly/simulated-bwa.DEL.50-100.1e-05.tsv - matched-calls/varlociraptor-delly/simulated-bwa.DEL.50-100.0.0001.tsv - matched-calls/varlociraptor-delly/simulated-bwa.DEL.50-100.0.001.tsv - matched-calls/varlociraptor-delly/simulated-bwa.DEL.50-100.0.01.tsv - matched-calls/varlociraptor-delly/simulated-bwa.DEL.50-100.0.05.tsv - matched-calls/varlociraptor-delly/simulated-bwa.DEL.50-100.0.1.tsv - matched-calls/varlociraptor-delly/simulated-bwa.DEL.50-100.0.2.tsv - matched-calls/varlociraptor-delly/simulated-bwa.DEL.50-100.0.3.tsv - matched-calls/varlociraptor-delly/simulated-bwa.DEL.50-100.0.4.tsv - matched-calls/varlociraptor-delly/simulated-bwa.DEL.50-100.0.5.tsv - matched-calls/varlociraptor-delly/simulated-bwa.DEL.50-100.0.6.tsv - matched-calls/varlociraptor-delly/simulated-bwa.DEL.50-100.0.7.tsv - matched-calls/varlociraptor-delly/simulated-bwa.DEL.50-100.0.8.tsv - matched-calls/varlociraptor-delly/simulated-bwa.DEL.50-100.0.9.tsv - matched-calls/varlociraptor-delly/simulated-bwa.DEL.100-250.1e-05.tsv - matched-calls/varlociraptor-delly/simulated-bwa.DEL.100-250.0.0001.tsv - matched-calls/varlociraptor-delly/simulated-bwa.DEL.100-250.0.001.tsv - matched-calls/varlociraptor-delly/simulated-bwa.DEL.100-250.0.01.tsv - matched-calls/varlociraptor-delly/simulated-bwa.DEL.100-250.0.05.tsv - matched-calls/varlociraptor-delly/simulated-bwa.DEL.100-250.0.1.tsv - matched-calls/varlociraptor-delly/simulated-bwa.DEL.100-250.0.2.tsv - matched-calls/varlociraptor-delly/simulated-bwa.DEL.100-250.0.3.tsv - matched-calls/varlociraptor-delly/simulated-bwa.DEL.100-250.0.4.tsv - matched-calls/varlociraptor-delly/simulated-bwa.DEL.100-250.0.5.tsv - matched-calls/varlociraptor-delly/simulated-bwa.DEL.100-250.0.6.tsv - matched-calls/varlociraptor-delly/simulated-bwa.DEL.100-250.0.7.tsv - matched-calls/varlociraptor-delly/simulated-bwa.DEL.100-250.0.8.tsv - matched-calls/varlociraptor-delly/simulated-bwa.DEL.100-250.0.9.tsv - matched-calls/varlociraptor-lancet/simulated-bwa.DEL.1-30.1e-05.tsv - matched-calls/varlociraptor-lancet/simulated-bwa.DEL.1-30.0.0001.tsv - matched-calls/varlociraptor-lancet/simulated-bwa.DEL.1-30.0.001.tsv - matched-calls/varlociraptor-lancet/simulated-bwa.DEL.1-30.0.01.tsv - matched-calls/varlociraptor-lancet/simulated-bwa.DEL.1-30.0.05.tsv - matched-calls/varlociraptor-lancet/simulated-bwa.DEL.1-30.0.1.tsv - matched-calls/varlociraptor-lancet/simulated-bwa.DEL.1-30.0.2.tsv - matched-calls/varlociraptor-lancet/simulated-bwa.DEL.1-30.0.3.tsv - matched-calls/varlociraptor-lancet/simulated-bwa.DEL.1-30.0.4.tsv - matched-calls/varlociraptor-lancet/simulated-bwa.DEL.1-30.0.5.tsv - matched-calls/varlociraptor-lancet/simulated-bwa.DEL.1-30.0.6.tsv - matched-calls/varlociraptor-lancet/simulated-bwa.DEL.1-30.0.7.tsv - matched-calls/varlociraptor-lancet/simulated-bwa.DEL.1-30.0.8.tsv - matched-calls/varlociraptor-lancet/simulated-bwa.DEL.1-30.0.9.tsv - matched-calls/varlociraptor-lancet/simulated-bwa.DEL.30-50.1e-05.tsv - matched-calls/varlociraptor-lancet/simulated-bwa.DEL.30-50.0.0001.tsv - matched-calls/varlociraptor-lancet/simulated-bwa.DEL.30-50.0.001.tsv - matched-calls/varlociraptor-lancet/simulated-bwa.DEL.30-50.0.01.tsv - matched-calls/varlociraptor-lancet/simulated-bwa.DEL.30-50.0.05.tsv - matched-calls/varlociraptor-lancet/simulated-bwa.DEL.30-50.0.1.tsv - matched-calls/varlociraptor-lancet/simulated-bwa.DEL.30-50.0.2.tsv - matched-calls/varlociraptor-lancet/simulated-bwa.DEL.30-50.0.3.tsv - matched-calls/varlociraptor-lancet/simulated-bwa.DEL.30-50.0.4.tsv - matched-calls/varlociraptor-lancet/simulated-bwa.DEL.30-50.0.5.tsv - matched-calls/varlociraptor-lancet/simulated-bwa.DEL.30-50.0.6.tsv - matched-calls/varlociraptor-lancet/simulated-bwa.DEL.30-50.0.7.tsv - matched-calls/varlociraptor-lancet/simulated-bwa.DEL.30-50.0.8.tsv - matched-calls/varlociraptor-lancet/simulated-bwa.DEL.30-50.0.9.tsv - matched-calls/varlociraptor-lancet/simulated-bwa.DEL.50-100.1e-05.tsv - matched-calls/varlociraptor-lancet/simulated-bwa.DEL.50-100.0.0001.tsv - matched-calls/varlociraptor-lancet/simulated-bwa.DEL.50-100.0.001.tsv - matched-calls/varlociraptor-lancet/simulated-bwa.DEL.50-100.0.01.tsv - matched-calls/varlociraptor-lancet/simulated-bwa.DEL.50-100.0.05.tsv - matched-calls/varlociraptor-lancet/simulated-bwa.DEL.50-100.0.1.tsv - matched-calls/varlociraptor-lancet/simulated-bwa.DEL.50-100.0.2.tsv - matched-calls/varlociraptor-lancet/simulated-bwa.DEL.50-100.0.3.tsv - matched-calls/varlociraptor-lancet/simulated-bwa.DEL.50-100.0.4.tsv - matched-calls/varlociraptor-lancet/simulated-bwa.DEL.50-100.0.5.tsv - matched-calls/varlociraptor-lancet/simulated-bwa.DEL.50-100.0.6.tsv - matched-calls/varlociraptor-lancet/simulated-bwa.DEL.50-100.0.7.tsv - matched-calls/varlociraptor-lancet/simulated-bwa.DEL.50-100.0.8.tsv - matched-calls/varlociraptor-lancet/simulated-bwa.DEL.50-100.0.9.tsv - matched-calls/varlociraptor-lancet/simulated-bwa.DEL.100-250.1e-05.tsv - matched-calls/varlociraptor-lancet/simulated-bwa.DEL.100-250.0.0001.tsv - matched-calls/varlociraptor-lancet/simulated-bwa.DEL.100-250.0.001.tsv - matched-calls/varlociraptor-lancet/simulated-bwa.DEL.100-250.0.01.tsv - matched-calls/varlociraptor-lancet/simulated-bwa.DEL.100-250.0.05.tsv - matched-calls/varlociraptor-lancet/simulated-bwa.DEL.100-250.0.1.tsv - matched-calls/varlociraptor-lancet/simulated-bwa.DEL.100-250.0.2.tsv - matched-calls/varlociraptor-lancet/simulated-bwa.DEL.100-250.0.3.tsv - matched-calls/varlociraptor-lancet/simulated-bwa.DEL.100-250.0.4.tsv - matched-calls/varlociraptor-lancet/simulated-bwa.DEL.100-250.0.5.tsv - matched-calls/varlociraptor-lancet/simulated-bwa.DEL.100-250.0.6.tsv - matched-calls/varlociraptor-lancet/simulated-bwa.DEL.100-250.0.7.tsv - matched-calls/varlociraptor-lancet/simulated-bwa.DEL.100-250.0.8.tsv - matched-calls/varlociraptor-lancet/simulated-bwa.DEL.100-250.0.9.tsv - matched-calls/varlociraptor-manta/simulated-bwa.DEL.1-30.1e-05.tsv - matched-calls/varlociraptor-manta/simulated-bwa.DEL.1-30.0.0001.tsv - matched-calls/varlociraptor-manta/simulated-bwa.DEL.1-30.0.001.tsv - matched-calls/varlociraptor-manta/simulated-bwa.DEL.1-30.0.01.tsv - matched-calls/varlociraptor-manta/simulated-bwa.DEL.1-30.0.05.tsv - matched-calls/varlociraptor-manta/simulated-bwa.DEL.1-30.0.1.tsv - matched-calls/varlociraptor-manta/simulated-bwa.DEL.1-30.0.2.tsv - matched-calls/varlociraptor-manta/simulated-bwa.DEL.1-30.0.3.tsv - matched-calls/varlociraptor-manta/simulated-bwa.DEL.1-30.0.4.tsv - matched-calls/varlociraptor-manta/simulated-bwa.DEL.1-30.0.5.tsv - matched-calls/varlociraptor-manta/simulated-bwa.DEL.1-30.0.6.tsv - matched-calls/varlociraptor-manta/simulated-bwa.DEL.1-30.0.7.tsv - matched-calls/varlociraptor-manta/simulated-bwa.DEL.1-30.0.8.tsv - matched-calls/varlociraptor-manta/simulated-bwa.DEL.1-30.0.9.tsv - matched-calls/varlociraptor-manta/simulated-bwa.DEL.30-50.1e-05.tsv - matched-calls/varlociraptor-manta/simulated-bwa.DEL.30-50.0.0001.tsv - matched-calls/varlociraptor-manta/simulated-bwa.DEL.30-50.0.001.tsv - matched-calls/varlociraptor-manta/simulated-bwa.DEL.30-50.0.01.tsv - matched-calls/varlociraptor-manta/simulated-bwa.DEL.30-50.0.05.tsv - matched-calls/varlociraptor-manta/simulated-bwa.DEL.30-50.0.1.tsv - matched-calls/varlociraptor-manta/simulated-bwa.DEL.30-50.0.2.tsv - matched-calls/varlociraptor-manta/simulated-bwa.DEL.30-50.0.3.tsv - matched-calls/varlociraptor-manta/simulated-bwa.DEL.30-50.0.4.tsv - matched-calls/varlociraptor-manta/simulated-bwa.DEL.30-50.0.5.tsv - matched-calls/varlociraptor-manta/simulated-bwa.DEL.30-50.0.6.tsv - matched-calls/varlociraptor-manta/simulated-bwa.DEL.30-50.0.7.tsv - matched-calls/varlociraptor-manta/simulated-bwa.DEL.30-50.0.8.tsv - matched-calls/varlociraptor-manta/simulated-bwa.DEL.30-50.0.9.tsv - matched-calls/varlociraptor-manta/simulated-bwa.DEL.50-100.1e-05.tsv - matched-calls/varlociraptor-manta/simulated-bwa.DEL.50-100.0.0001.tsv - matched-calls/varlociraptor-manta/simulated-bwa.DEL.50-100.0.001.tsv - matched-calls/varlociraptor-manta/simulated-bwa.DEL.50-100.0.01.tsv - matched-calls/varlociraptor-manta/simulated-bwa.DEL.50-100.0.05.tsv - matched-calls/varlociraptor-manta/simulated-bwa.DEL.50-100.0.1.tsv - matched-calls/varlociraptor-manta/simulated-bwa.DEL.50-100.0.2.tsv - matched-calls/varlociraptor-manta/simulated-bwa.DEL.50-100.0.3.tsv - matched-calls/varlociraptor-manta/simulated-bwa.DEL.50-100.0.4.tsv - matched-calls/varlociraptor-manta/simulated-bwa.DEL.50-100.0.5.tsv - matched-calls/varlociraptor-manta/simulated-bwa.DEL.50-100.0.6.tsv - matched-calls/varlociraptor-manta/simulated-bwa.DEL.50-100.0.7.tsv - matched-calls/varlociraptor-manta/simulated-bwa.DEL.50-100.0.8.tsv - matched-calls/varlociraptor-manta/simulated-bwa.DEL.50-100.0.9.tsv - matched-calls/varlociraptor-manta/simulated-bwa.DEL.100-250.1e-05.tsv - matched-calls/varlociraptor-manta/simulated-bwa.DEL.100-250.0.0001.tsv - matched-calls/varlociraptor-manta/simulated-bwa.DEL.100-250.0.001.tsv - matched-calls/varlociraptor-manta/simulated-bwa.DEL.100-250.0.01.tsv - matched-calls/varlociraptor-manta/simulated-bwa.DEL.100-250.0.05.tsv - matched-calls/varlociraptor-manta/simulated-bwa.DEL.100-250.0.1.tsv - matched-calls/varlociraptor-manta/simulated-bwa.DEL.100-250.0.2.tsv - matched-calls/varlociraptor-manta/simulated-bwa.DEL.100-250.0.3.tsv - matched-calls/varlociraptor-manta/simulated-bwa.DEL.100-250.0.4.tsv - matched-calls/varlociraptor-manta/simulated-bwa.DEL.100-250.0.5.tsv - matched-calls/varlociraptor-manta/simulated-bwa.DEL.100-250.0.6.tsv - matched-calls/varlociraptor-manta/simulated-bwa.DEL.100-250.0.7.tsv - matched-calls/varlociraptor-manta/simulated-bwa.DEL.100-250.0.8.tsv - matched-calls/varlociraptor-manta/simulated-bwa.DEL.100-250.0.9.tsv - matched-calls/varlociraptor-strelka/simulated-bwa.DEL.1-30.1e-05.tsv - matched-calls/varlociraptor-strelka/simulated-bwa.DEL.1-30.0.0001.tsv - matched-calls/varlociraptor-strelka/simulated-bwa.DEL.1-30.0.001.tsv - matched-calls/varlociraptor-strelka/simulated-bwa.DEL.1-30.0.01.tsv - matched-calls/varlociraptor-strelka/simulated-bwa.DEL.1-30.0.05.tsv - matched-calls/varlociraptor-strelka/simulated-bwa.DEL.1-30.0.1.tsv - matched-calls/varlociraptor-strelka/simulated-bwa.DEL.1-30.0.2.tsv - matched-calls/varlociraptor-strelka/simulated-bwa.DEL.1-30.0.3.tsv - matched-calls/varlociraptor-strelka/simulated-bwa.DEL.1-30.0.4.tsv - matched-calls/varlociraptor-strelka/simulated-bwa.DEL.1-30.0.5.tsv - matched-calls/varlociraptor-strelka/simulated-bwa.DEL.1-30.0.6.tsv - matched-calls/varlociraptor-strelka/simulated-bwa.DEL.1-30.0.7.tsv - matched-calls/varlociraptor-strelka/simulated-bwa.DEL.1-30.0.8.tsv - matched-calls/varlociraptor-strelka/simulated-bwa.DEL.1-30.0.9.tsv - matched-calls/varlociraptor-strelka/simulated-bwa.DEL.30-50.1e-05.tsv - matched-calls/varlociraptor-strelka/simulated-bwa.DEL.30-50.0.0001.tsv - matched-calls/varlociraptor-strelka/simulated-bwa.DEL.30-50.0.001.tsv - matched-calls/varlociraptor-strelka/simulated-bwa.DEL.30-50.0.01.tsv - matched-calls/varlociraptor-strelka/simulated-bwa.DEL.30-50.0.05.tsv - matched-calls/varlociraptor-strelka/simulated-bwa.DEL.30-50.0.1.tsv - matched-calls/varlociraptor-strelka/simulated-bwa.DEL.30-50.0.2.tsv - matched-calls/varlociraptor-strelka/simulated-bwa.DEL.30-50.0.3.tsv - matched-calls/varlociraptor-strelka/simulated-bwa.DEL.30-50.0.4.tsv - matched-calls/varlociraptor-strelka/simulated-bwa.DEL.30-50.0.5.tsv - matched-calls/varlociraptor-strelka/simulated-bwa.DEL.30-50.0.6.tsv - matched-calls/varlociraptor-strelka/simulated-bwa.DEL.30-50.0.7.tsv - matched-calls/varlociraptor-strelka/simulated-bwa.DEL.30-50.0.8.tsv - matched-calls/varlociraptor-strelka/simulated-bwa.DEL.30-50.0.9.tsv - matched-calls/varlociraptor-strelka/simulated-bwa.DEL.50-100.1e-05.tsv - matched-calls/varlociraptor-strelka/simulated-bwa.DEL.50-100.0.0001.tsv - matched-calls/varlociraptor-strelka/simulated-bwa.DEL.50-100.0.001.tsv - matched-calls/varlociraptor-strelka/simulated-bwa.DEL.50-100.0.01.tsv - matched-calls/varlociraptor-strelka/simulated-bwa.DEL.50-100.0.05.tsv - matched-calls/varlociraptor-strelka/simulated-bwa.DEL.50-100.0.1.tsv - matched-calls/varlociraptor-strelka/simulated-bwa.DEL.50-100.0.2.tsv - matched-calls/varlociraptor-strelka/simulated-bwa.DEL.50-100.0.3.tsv - matched-calls/varlociraptor-strelka/simulated-bwa.DEL.50-100.0.4.tsv - matched-calls/varlociraptor-strelka/simulated-bwa.DEL.50-100.0.5.tsv - matched-calls/varlociraptor-strelka/simulated-bwa.DEL.50-100.0.6.tsv - matched-calls/varlociraptor-strelka/simulated-bwa.DEL.50-100.0.7.tsv - matched-calls/varlociraptor-strelka/simulated-bwa.DEL.50-100.0.8.tsv - matched-calls/varlociraptor-strelka/simulated-bwa.DEL.50-100.0.9.tsv - matched-calls/varlociraptor-strelka/simulated-bwa.DEL.100-250.1e-05.tsv - matched-calls/varlociraptor-strelka/simulated-bwa.DEL.100-250.0.0001.tsv - matched-calls/varlociraptor-strelka/simulated-bwa.DEL.100-250.0.001.tsv - matched-calls/varlociraptor-strelka/simulated-bwa.DEL.100-250.0.01.tsv - matched-calls/varlociraptor-strelka/simulated-bwa.DEL.100-250.0.05.tsv - matched-calls/varlociraptor-strelka/simulated-bwa.DEL.100-250.0.1.tsv - matched-calls/varlociraptor-strelka/simulated-bwa.DEL.100-250.0.2.tsv - matched-calls/varlociraptor-strelka/simulated-bwa.DEL.100-250.0.3.tsv - matched-calls/varlociraptor-strelka/simulated-bwa.DEL.100-250.0.4.tsv - matched-calls/varlociraptor-strelka/simulated-bwa.DEL.100-250.0.5.tsv - matched-calls/varlociraptor-strelka/simulated-bwa.DEL.100-250.0.6.tsv - matched-calls/varlociraptor-strelka/simulated-bwa.DEL.100-250.0.7.tsv - matched-calls/varlociraptor-strelka/simulated-bwa.DEL.100-250.0.8.tsv - matched-calls/varlociraptor-strelka/simulated-bwa.DEL.100-250.0.9.tsv - matched-calls/varlociraptor-delly/simulated-bwa.INS.1-250.1.0.tsv - matched-calls/varlociraptor-lancet/simulated-bwa.INS.1-250.1.0.tsv - matched-calls/varlociraptor-manta/simulated-bwa.INS.1-250.1.0.tsv - matched-calls/varlociraptor-strelka/simulated-bwa.INS.1-250.1.0.tsv - matched-calls/varlociraptor-delly/simulated-bwa.DEL.1-250.1.0.tsv - matched-calls/varlociraptor-lancet/simulated-bwa.DEL.1-250.1.0.tsv - matched-calls/varlociraptor-manta/simulated-bwa.DEL.1-250.1.0.tsv - matched-calls/varlociraptor-strelka/simulated-bwa.DEL.1-250.1.0.tsv |  | - rust-bio-tools =0.5.0 - bedtools =2.27.1 - bcftools =1.8 | |  |  | | --- | --- | | ``` 1 ``` | ``` rbt vcf-to-txt {params.gt} {params.tags} --info MATCHING < {input} > {output} ``` | |
| other\_calls\_to\_tsv | 23 | - matched-calls/default-neusomatic/simulated-bwa.all.tsv - matched-calls/adhoc-neusomatic/simulated-bwa.all.tsv - matched-calls/adhoc-delly/simulated-bwa.all.tsv - matched-calls/default-neusomatic/synthetic-5.all.tsv - matched-calls/default-lancet/synthetic-5.all.tsv - matched-calls/default-manta/synthetic-5.all.tsv - matched-calls/default-strelka/synthetic-5.all.tsv - matched-calls/adhoc-neusomatic/synthetic-5.all.tsv - matched-calls/adhoc-delly/synthetic-5.all.tsv - matched-calls/adhoc-lancet/synthetic-5.all.tsv - matched-calls/adhoc-manta/synthetic-5.all.tsv - matched-calls/adhoc-strelka/synthetic-5.all.tsv - matched-calls/adhoc-bpi/synthetic-5.all.tsv - matched-calls/default-neusomatic/synthetic-20.all.tsv - matched-calls/default-lancet/synthetic-20.all.tsv - matched-calls/default-manta/synthetic-20.all.tsv - matched-calls/default-strelka/synthetic-20.all.tsv - matched-calls/adhoc-neusomatic/synthetic-20.all.tsv - matched-calls/adhoc-delly/synthetic-20.all.tsv - matched-calls/adhoc-lancet/synthetic-20.all.tsv - matched-calls/adhoc-manta/synthetic-20.all.tsv - matched-calls/adhoc-strelka/synthetic-20.all.tsv - matched-calls/adhoc-bpi/synthetic-20.all.tsv |  | - rust-bio-tools =0.9.0 - bedtools =2.27.1 - bcftools =1.8 | |  |  | | --- | --- | | ``` 1 ``` | ``` rbt vcf-to-txt {params.gt} {params.tags} --info MATCHING < {input} > {output} ``` | |
| other\_calls\_to\_tsv | 7 | - matched-calls/default-lancet/simulated-bwa.all.tsv - matched-calls/default-manta/simulated-bwa.all.tsv - matched-calls/default-strelka/simulated-bwa.all.tsv - matched-calls/adhoc-lancet/simulated-bwa.all.tsv - matched-calls/adhoc-manta/simulated-bwa.all.tsv - matched-calls/adhoc-strelka/simulated-bwa.all.tsv - matched-calls/adhoc-bpi/simulated-bwa.all.tsv |  | - rust-bio-tools =0.2.5 - bedtools =2.27.1 - bcftools =1.8 | |  |  | | --- | --- | | ``` 1 ``` | ``` rbt vcf-to-txt {params.gt} {params.tags} --info MATCHING < {input} > {output} ``` | |
| aggregate\_concordance | 36 | - aggregated-concordance/varlociraptor-neusomatic-0.9/colo1.INS.tsv - aggregated-concordance/varlociraptor-delly-0.9/colo1.INS.tsv - aggregated-concordance/varlociraptor-lancet-0.9/colo1.INS.tsv - aggregated-concordance/varlociraptor-manta-0.9/colo1.INS.tsv - aggregated-concordance/varlociraptor-strelka-0.9/colo1.INS.tsv - aggregated-concordance/varlociraptor-bpi-0.9/colo1.INS.tsv - aggregated-concordance/varlociraptor-neusomatic-0.98/colo1.INS.tsv - aggregated-concordance/varlociraptor-delly-0.98/colo1.INS.tsv - aggregated-concordance/varlociraptor-lancet-0.98/colo1.INS.tsv - aggregated-concordance/varlociraptor-manta-0.98/colo1.INS.tsv - aggregated-concordance/varlociraptor-strelka-0.98/colo1.INS.tsv - aggregated-concordance/varlociraptor-bpi-0.98/colo1.INS.tsv - aggregated-concordance/adhoc-neusomatic-default/colo1.INS.tsv - aggregated-concordance/adhoc-delly-default/colo1.INS.tsv - aggregated-concordance/adhoc-lancet-default/colo1.INS.tsv - aggregated-concordance/adhoc-manta-default/colo1.INS.tsv - aggregated-concordance/adhoc-strelka-default/colo1.INS.tsv - aggregated-concordance/adhoc-bpi-default/colo1.INS.tsv - aggregated-concordance/varlociraptor-neusomatic-0.9/colo1.DEL.tsv - aggregated-concordance/varlociraptor-delly-0.9/colo1.DEL.tsv - aggregated-concordance/varlociraptor-lancet-0.9/colo1.DEL.tsv - aggregated-concordance/varlociraptor-manta-0.9/colo1.DEL.tsv - aggregated-concordance/varlociraptor-strelka-0.9/colo1.DEL.tsv - aggregated-concordance/varlociraptor-bpi-0.9/colo1.DEL.tsv - aggregated-concordance/varlociraptor-neusomatic-0.98/colo1.DEL.tsv - aggregated-concordance/varlociraptor-delly-0.98/colo1.DEL.tsv - aggregated-concordance/varlociraptor-lancet-0.98/colo1.DEL.tsv - aggregated-concordance/varlociraptor-manta-0.98/colo1.DEL.tsv - aggregated-concordance/varlociraptor-strelka-0.98/colo1.DEL.tsv - aggregated-concordance/varlociraptor-bpi-0.98/colo1.DEL.tsv - aggregated-concordance/adhoc-neusomatic-default/colo1.DEL.tsv - aggregated-concordance/adhoc-delly-default/colo1.DEL.tsv - aggregated-concordance/adhoc-lancet-default/colo1.DEL.tsv - aggregated-concordance/adhoc-manta-default/colo1.DEL.tsv - aggregated-concordance/adhoc-strelka-default/colo1.DEL.tsv - aggregated-concordance/adhoc-bpi-default/colo1.DEL.tsv |  | - python =3.6 - pandas =0.23 - matplotlib =3.0 - seaborn =0.9.0 - pysam =0.13.0 - svgutils =0.2 - pybedtools =0.7.10 - networkx =2.2 | |  |  | | --- | --- | | ```  1  2  3  4  5  6  7  8  9 10 11 12 13 14 15 16 17 18 19 20 21 22 23 24 25 26 27 28 29 30 31 32 33 34 35 36 37 38 39 40 41 42 43 44 45 46 47 48 49 50 51 52 53 54 55 56 57 58 59 60 61 62 63 64 65 66 67 68 69 70 71 72 73 74 75 76 77 78 79 ``` | ``` from common import load_variants import networkx as nx import pandas as pd import numpy as np  vartype = snakemake.wildcards.vartype  index_cols = ["CHROM", "POS", "SVLEN"] if vartype == "INS" or vartype == "DEL" else ["CHROM", "POS", "ALT"]  all_variants = [load_variants(f, vartype=vartype) for f in snakemake.input.calls]  G = nx.Graph() for calls, (i, j) in zip(all_variants, snakemake.params.dataset_combinations):     calls["component"] = None     for call in calls.itertuples():         a = (i, call.Index)         G.add_node(a)         if call.MATCHING >= 0:             b = (j, call.MATCHING)             G.add_node(b)             G.add_edge(a, b)  # get a set of calls for each dataset (we don't need all pairwise comparisons for that) representatives = {snakemake.params.dataset_combinations[i][0]: calls for i, calls in enumerate(all_variants)}  if snakemake.wildcards.mode != "varlociraptor":     varlociraptor_variants = [load_variants(f, vartype=vartype) for f in snakemake.input.varlociraptor_calls]     for calls in varlociraptor_variants:         calls.set_index(index_cols, inplace=True)     varlociraptor_representatives = {snakemake.params.dataset_combinations[i][0]: calls for i, calls in enumerate(varlociraptor_variants)}  # annotate calls with their component, i.e. their equivalence class for component_id, component in enumerate(nx.connected_components(G)):     for i, k in component:         representatives[i].loc[k, "component"] = component_id for calls in representatives.values():     calls["component"] = calls["component"].astype(np.float32)     calls.set_index("component", inplace=True)  # join calls based on their equivalence class aggregated = None suffix = "_{}".format dataset_name = lambda i: snakemake.params.datasets[i] is_varlociraptor = False for dataset_id, calls in representatives.items():     cols = list(index_cols)     if "CASE_AF" in calls.columns:         cols.extend(["CASE_AF", "PROB_SOMATIC_TUMOR"])         is_varlociraptor = True     calls = calls[cols]     if snakemake.wildcards.mode != "varlociraptor":         idx_calls = calls.set_index(cols, drop=False)         caseaf = idx_calls.join(varlociraptor_representatives[dataset_id][["CASE_AF"]], how="left")["CASE_AF"]         caseaf = caseaf[~caseaf.index.duplicated()]         calls = calls[~idx_calls.index.duplicated()]         calls["CASE_AF"] = caseaf.values      calls.columns = [c + suffix(dataset_name(dataset_id)) for c in calls.columns]     if aggregated is None:         aggregated = calls     else:         aggregated = aggregated.join(calls, how="outer", lsuffix="", rsuffix="")  # Forget the component id. Otherwise, we might run into errors with duplicate elements # in the index below. These can occur if there are multiple ambiguous calls. aggregated.reset_index(inplace=True, drop=True)  pos_cols = aggregated.columns[aggregated.columns.str.startswith("POS_")] is_called = (~aggregated[pos_cols].isnull()).astype(int) is_called.columns = pos_cols.str.replace("POS_", "") aggregated = aggregated.join(is_called, lsuffix="", rsuffix="")  aggregated.insert(len(aggregated.columns), "concordance_count", is_called.sum(axis=1))  aggregated["max_case_af"] = aggregated[aggregated.columns[aggregated.columns.str.startswith("CASE_AF")]].max(axis=1) if is_varlociraptor:     aggregated["max_prob_somatic_tumor"] =  aggregated[aggregated.columns[aggregated.columns.str.startswith("PROB_SOMATIC")]].min(axis=1)  aggregated.to_csv(snakemake.output[0], sep="\t", index=False) ``` | |
| match\_varlociraptor\_calls | 122 | - matched-calls/varlociraptor-neusomatic/simulated-bwa.INS.1-30.1.0.bcf - matched-calls/varlociraptor-neusomatic/simulated-bwa.INS.30-100.1.0.bcf - matched-calls/varlociraptor-neusomatic/synthetic-5.INS.1-250.1.0.bcf - matched-calls/varlociraptor-delly/synthetic-5.INS.1-250.1.0.bcf - matched-calls/varlociraptor-lancet/synthetic-5.INS.1-250.1.0.bcf - matched-calls/varlociraptor-manta/synthetic-5.INS.1-250.1.0.bcf - matched-calls/varlociraptor-strelka/synthetic-5.INS.1-250.1.0.bcf - matched-calls/varlociraptor-neusomatic/synthetic-20.INS.1-30.1.0.bcf - matched-calls/varlociraptor-neusomatic/synthetic-20.INS.30-250.1.0.bcf - matched-calls/varlociraptor-delly/synthetic-20.INS.1-30.1.0.bcf - matched-calls/varlociraptor-delly/synthetic-20.INS.30-250.1.0.bcf - matched-calls/varlociraptor-lancet/synthetic-20.INS.1-30.1.0.bcf - matched-calls/varlociraptor-lancet/synthetic-20.INS.30-250.1.0.bcf - matched-calls/varlociraptor-manta/synthetic-20.INS.1-30.1.0.bcf - matched-calls/varlociraptor-manta/synthetic-20.INS.30-250.1.0.bcf - matched-calls/varlociraptor-strelka/synthetic-20.INS.1-30.1.0.bcf - matched-calls/varlociraptor-strelka/synthetic-20.INS.30-250.1.0.bcf - matched-calls/varlociraptor-neusomatic/simulated-bwa.DEL.1-30.1.0.bcf - matched-calls/varlociraptor-neusomatic/simulated-bwa.DEL.30-50.1.0.bcf - matched-calls/varlociraptor-neusomatic/simulated-bwa.DEL.50-100.1.0.bcf - matched-calls/varlociraptor-neusomatic/simulated-bwa.DEL.100-250.1.0.bcf - matched-calls/varlociraptor-neusomatic/synthetic-5.DEL.1-250.1.0.bcf - matched-calls/varlociraptor-delly/synthetic-5.DEL.1-250.1.0.bcf - matched-calls/varlociraptor-lancet/synthetic-5.DEL.1-250.1.0.bcf - matched-calls/varlociraptor-manta/synthetic-5.DEL.1-250.1.0.bcf - matched-calls/varlociraptor-strelka/synthetic-5.DEL.1-250.1.0.bcf - matched-calls/varlociraptor-neusomatic/synthetic-20.DEL.1-30.1.0.bcf - matched-calls/varlociraptor-neusomatic/synthetic-20.DEL.30-250.1.0.bcf - matched-calls/varlociraptor-delly/synthetic-20.DEL.1-30.1.0.bcf - matched-calls/varlociraptor-delly/synthetic-20.DEL.30-250.1.0.bcf - matched-calls/varlociraptor-lancet/synthetic-20.DEL.1-30.1.0.bcf - matched-calls/varlociraptor-lancet/synthetic-20.DEL.30-250.1.0.bcf - matched-calls/varlociraptor-manta/synthetic-20.DEL.1-30.1.0.bcf - matched-calls/varlociraptor-manta/synthetic-20.DEL.30-250.1.0.bcf - matched-calls/varlociraptor-strelka/synthetic-20.DEL.1-30.1.0.bcf - matched-calls/varlociraptor-strelka/synthetic-20.DEL.30-250.1.0.bcf - matched-calls/varlociraptor-neusomatic/simulated-bwa.INS.1-30.1e-05.bcf - matched-calls/varlociraptor-neusomatic/simulated-bwa.INS.1-30.0.0001.bcf - matched-calls/varlociraptor-neusomatic/simulated-bwa.INS.1-30.0.001.bcf - matched-calls/varlociraptor-neusomatic/simulated-bwa.INS.1-30.0.01.bcf - matched-calls/varlociraptor-neusomatic/simulated-bwa.INS.1-30.0.05.bcf - matched-calls/varlociraptor-neusomatic/simulated-bwa.INS.1-30.0.1.bcf - matched-calls/varlociraptor-neusomatic/simulated-bwa.INS.1-30.0.2.bcf - matched-calls/varlociraptor-neusomatic/simulated-bwa.INS.1-30.0.3.bcf - matched-calls/varlociraptor-neusomatic/simulated-bwa.INS.1-30.0.4.bcf - matched-calls/varlociraptor-neusomatic/simulated-bwa.INS.1-30.0.5.bcf - matched-calls/varlociraptor-neusomatic/simulated-bwa.INS.1-30.0.6.bcf - matched-calls/varlociraptor-neusomatic/simulated-bwa.INS.1-30.0.7.bcf - matched-calls/varlociraptor-neusomatic/simulated-bwa.INS.1-30.0.8.bcf - matched-calls/varlociraptor-neusomatic/simulated-bwa.INS.1-30.0.9.bcf - matched-calls/varlociraptor-neusomatic/simulated-bwa.INS.30-100.1e-05.bcf - matched-calls/varlociraptor-neusomatic/simulated-bwa.INS.30-100.0.0001.bcf - matched-calls/varlociraptor-neusomatic/simulated-bwa.INS.30-100.0.001.bcf - matched-calls/varlociraptor-neusomatic/simulated-bwa.INS.30-100.0.01.bcf - matched-calls/varlociraptor-neusomatic/simulated-bwa.INS.30-100.0.05.bcf - matched-calls/varlociraptor-neusomatic/simulated-bwa.INS.30-100.0.1.bcf - matched-calls/varlociraptor-neusomatic/simulated-bwa.INS.30-100.0.2.bcf - matched-calls/varlociraptor-neusomatic/simulated-bwa.INS.30-100.0.3.bcf - matched-calls/varlociraptor-neusomatic/simulated-bwa.INS.30-100.0.4.bcf - matched-calls/varlociraptor-neusomatic/simulated-bwa.INS.30-100.0.5.bcf - matched-calls/varlociraptor-neusomatic/simulated-bwa.INS.30-100.0.6.bcf - matched-calls/varlociraptor-neusomatic/simulated-bwa.INS.30-100.0.7.bcf - matched-calls/varlociraptor-neusomatic/simulated-bwa.INS.30-100.0.8.bcf - matched-calls/varlociraptor-neusomatic/simulated-bwa.INS.30-100.0.9.bcf - matched-calls/varlociraptor-neusomatic/simulated-bwa.DEL.1-30.1e-05.bcf - matched-calls/varlociraptor-neusomatic/simulated-bwa.DEL.1-30.0.0001.bcf - matched-calls/varlociraptor-neusomatic/simulated-bwa.DEL.1-30.0.001.bcf - matched-calls/varlociraptor-neusomatic/simulated-bwa.DEL.1-30.0.01.bcf - matched-calls/varlociraptor-neusomatic/simulated-bwa.DEL.1-30.0.05.bcf - matched-calls/varlociraptor-neusomatic/simulated-bwa.DEL.1-30.0.1.bcf - matched-calls/varlociraptor-neusomatic/simulated-bwa.DEL.1-30.0.2.bcf - matched-calls/varlociraptor-neusomatic/simulated-bwa.DEL.1-30.0.3.bcf - matched-calls/varlociraptor-neusomatic/simulated-bwa.DEL.1-30.0.4.bcf - matched-calls/varlociraptor-neusomatic/simulated-bwa.DEL.1-30.0.5.bcf - matched-calls/varlociraptor-neusomatic/simulated-bwa.DEL.1-30.0.6.bcf - matched-calls/varlociraptor-neusomatic/simulated-bwa.DEL.1-30.0.7.bcf - matched-calls/varlociraptor-neusomatic/simulated-bwa.DEL.1-30.0.8.bcf - matched-calls/varlociraptor-neusomatic/simulated-bwa.DEL.1-30.0.9.bcf - matched-calls/varlociraptor-neusomatic/simulated-bwa.DEL.30-50.1e-05.bcf - matched-calls/varlociraptor-neusomatic/simulated-bwa.DEL.30-50.0.0001.bcf - matched-calls/varlociraptor-neusomatic/simulated-bwa.DEL.30-50.0.001.bcf - matched-calls/varlociraptor-neusomatic/simulated-bwa.DEL.30-50.0.01.bcf - matched-calls/varlociraptor-neusomatic/simulated-bwa.DEL.30-50.0.05.bcf - matched-calls/varlociraptor-neusomatic/simulated-bwa.DEL.30-50.0.1.bcf - matched-calls/varlociraptor-neusomatic/simulated-bwa.DEL.30-50.0.2.bcf - matched-calls/varlociraptor-neusomatic/simulated-bwa.DEL.30-50.0.3.bcf - matched-calls/varlociraptor-neusomatic/simulated-bwa.DEL.30-50.0.4.bcf - matched-calls/varlociraptor-neusomatic/simulated-bwa.DEL.30-50.0.5.bcf - matched-calls/varlociraptor-neusomatic/simulated-bwa.DEL.30-50.0.6.bcf - matched-calls/varlociraptor-neusomatic/simulated-bwa.DEL.30-50.0.7.bcf - matched-calls/varlociraptor-neusomatic/simulated-bwa.DEL.30-50.0.8.bcf - matched-calls/varlociraptor-neusomatic/simulated-bwa.DEL.30-50.0.9.bcf - matched-calls/varlociraptor-neusomatic/simulated-bwa.DEL.50-100.1e-05.bcf - matched-calls/varlociraptor-neusomatic/simulated-bwa.DEL.50-100.0.0001.bcf - matched-calls/varlociraptor-neusomatic/simulated-bwa.DEL.50-100.0.001.bcf - matched-calls/varlociraptor-neusomatic/simulated-bwa.DEL.50-100.0.01.bcf - matched-calls/varlociraptor-neusomatic/simulated-bwa.DEL.50-100.0.05.bcf - matched-calls/varlociraptor-neusomatic/simulated-bwa.DEL.50-100.0.1.bcf - matched-calls/varlociraptor-neusomatic/simulated-bwa.DEL.50-100.0.2.bcf - matched-calls/varlociraptor-neusomatic/simulated-bwa.DEL.50-100.0.3.bcf - matched-calls/varlociraptor-neusomatic/simulated-bwa.DEL.50-100.0.4.bcf - matched-calls/varlociraptor-neusomatic/simulated-bwa.DEL.50-100.0.5.bcf - matched-calls/varlociraptor-neusomatic/simulated-bwa.DEL.50-100.0.6.bcf - matched-calls/varlociraptor-neusomatic/simulated-bwa.DEL.50-100.0.7.bcf - matched-calls/varlociraptor-neusomatic/simulated-bwa.DEL.50-100.0.8.bcf - matched-calls/varlociraptor-neusomatic/simulated-bwa.DEL.50-100.0.9.bcf - matched-calls/varlociraptor-neusomatic/simulated-bwa.DEL.100-250.1e-05.bcf - matched-calls/varlociraptor-neusomatic/simulated-bwa.DEL.100-250.0.0001.bcf - matched-calls/varlociraptor-neusomatic/simulated-bwa.DEL.100-250.0.001.bcf - matched-calls/varlociraptor-neusomatic/simulated-bwa.DEL.100-250.0.01.bcf - matched-calls/varlociraptor-neusomatic/simulated-bwa.DEL.100-250.0.05.bcf - matched-calls/varlociraptor-neusomatic/simulated-bwa.DEL.100-250.0.1.bcf - matched-calls/varlociraptor-neusomatic/simulated-bwa.DEL.100-250.0.2.bcf - matched-calls/varlociraptor-neusomatic/simulated-bwa.DEL.100-250.0.3.bcf - matched-calls/varlociraptor-neusomatic/simulated-bwa.DEL.100-250.0.4.bcf - matched-calls/varlociraptor-neusomatic/simulated-bwa.DEL.100-250.0.5.bcf - matched-calls/varlociraptor-neusomatic/simulated-bwa.DEL.100-250.0.6.bcf - matched-calls/varlociraptor-neusomatic/simulated-bwa.DEL.100-250.0.7.bcf - matched-calls/varlociraptor-neusomatic/simulated-bwa.DEL.100-250.0.8.bcf - matched-calls/varlociraptor-neusomatic/simulated-bwa.DEL.100-250.0.9.bcf - matched-calls/varlociraptor-neusomatic/simulated-bwa.INS.1-250.1.0.bcf - matched-calls/varlociraptor-neusomatic/simulated-bwa.DEL.1-250.1.0.bcf |  | - rust-bio-tools =0.9.0 - bedtools =2.27.1 - bcftools =1.8 | |  |  | | --- | --- | | ``` 1 ``` | ``` bcftools view {params.regions} {input.calls} | rbt vcf-match {params.match} {input.truth} > {output} ``` | |
| match\_varlociraptor\_calls | 368 | - matched-calls/varlociraptor-delly/simulated-bwa.INS.1-30.1.0.bcf - matched-calls/varlociraptor-delly/simulated-bwa.INS.30-100.1.0.bcf - matched-calls/varlociraptor-lancet/simulated-bwa.INS.1-30.1.0.bcf - matched-calls/varlociraptor-lancet/simulated-bwa.INS.30-100.1.0.bcf - matched-calls/varlociraptor-manta/simulated-bwa.INS.1-30.1.0.bcf - matched-calls/varlociraptor-manta/simulated-bwa.INS.30-100.1.0.bcf - matched-calls/varlociraptor-strelka/simulated-bwa.INS.1-30.1.0.bcf - matched-calls/varlociraptor-strelka/simulated-bwa.INS.30-100.1.0.bcf - matched-calls/varlociraptor-delly/simulated-bwa.DEL.1-30.1.0.bcf - matched-calls/varlociraptor-delly/simulated-bwa.DEL.30-50.1.0.bcf - matched-calls/varlociraptor-delly/simulated-bwa.DEL.50-100.1.0.bcf - matched-calls/varlociraptor-delly/simulated-bwa.DEL.100-250.1.0.bcf - matched-calls/varlociraptor-lancet/simulated-bwa.DEL.1-30.1.0.bcf - matched-calls/varlociraptor-lancet/simulated-bwa.DEL.30-50.1.0.bcf - matched-calls/varlociraptor-lancet/simulated-bwa.DEL.50-100.1.0.bcf - matched-calls/varlociraptor-lancet/simulated-bwa.DEL.100-250.1.0.bcf - matched-calls/varlociraptor-manta/simulated-bwa.DEL.1-30.1.0.bcf - matched-calls/varlociraptor-manta/simulated-bwa.DEL.30-50.1.0.bcf - matched-calls/varlociraptor-manta/simulated-bwa.DEL.50-100.1.0.bcf - matched-calls/varlociraptor-manta/simulated-bwa.DEL.100-250.1.0.bcf - matched-calls/varlociraptor-strelka/simulated-bwa.DEL.1-30.1.0.bcf - matched-calls/varlociraptor-strelka/simulated-bwa.DEL.30-50.1.0.bcf - matched-calls/varlociraptor-strelka/simulated-bwa.DEL.50-100.1.0.bcf - matched-calls/varlociraptor-strelka/simulated-bwa.DEL.100-250.1.0.bcf - matched-calls/varlociraptor-delly/simulated-bwa.INS.1-30.1e-05.bcf - matched-calls/varlociraptor-delly/simulated-bwa.INS.1-30.0.0001.bcf - matched-calls/varlociraptor-delly/simulated-bwa.INS.1-30.0.001.bcf - matched-calls/varlociraptor-delly/simulated-bwa.INS.1-30.0.01.bcf - matched-calls/varlociraptor-delly/simulated-bwa.INS.1-30.0.05.bcf - matched-calls/varlociraptor-delly/simulated-bwa.INS.1-30.0.1.bcf - matched-calls/varlociraptor-delly/simulated-bwa.INS.1-30.0.2.bcf - matched-calls/varlociraptor-delly/simulated-bwa.INS.1-30.0.3.bcf - matched-calls/varlociraptor-delly/simulated-bwa.INS.1-30.0.4.bcf - matched-calls/varlociraptor-delly/simulated-bwa.INS.1-30.0.5.bcf - matched-calls/varlociraptor-delly/simulated-bwa.INS.1-30.0.6.bcf - matched-calls/varlociraptor-delly/simulated-bwa.INS.1-30.0.7.bcf - matched-calls/varlociraptor-delly/simulated-bwa.INS.1-30.0.8.bcf - matched-calls/varlociraptor-delly/simulated-bwa.INS.1-30.0.9.bcf - matched-calls/varlociraptor-delly/simulated-bwa.INS.30-100.1e-05.bcf - matched-calls/varlociraptor-delly/simulated-bwa.INS.30-100.0.0001.bcf - matched-calls/varlociraptor-delly/simulated-bwa.INS.30-100.0.001.bcf - matched-calls/varlociraptor-delly/simulated-bwa.INS.30-100.0.01.bcf - matched-calls/varlociraptor-delly/simulated-bwa.INS.30-100.0.05.bcf - matched-calls/varlociraptor-delly/simulated-bwa.INS.30-100.0.1.bcf - matched-calls/varlociraptor-delly/simulated-bwa.INS.30-100.0.2.bcf - matched-calls/varlociraptor-delly/simulated-bwa.INS.30-100.0.3.bcf - matched-calls/varlociraptor-delly/simulated-bwa.INS.30-100.0.4.bcf - matched-calls/varlociraptor-delly/simulated-bwa.INS.30-100.0.5.bcf - matched-calls/varlociraptor-delly/simulated-bwa.INS.30-100.0.6.bcf - matched-calls/varlociraptor-delly/simulated-bwa.INS.30-100.0.7.bcf - matched-calls/varlociraptor-delly/simulated-bwa.INS.30-100.0.8.bcf - matched-calls/varlociraptor-delly/simulated-bwa.INS.30-100.0.9.bcf - matched-calls/varlociraptor-lancet/simulated-bwa.INS.1-30.1e-05.bcf - matched-calls/varlociraptor-lancet/simulated-bwa.INS.1-30.0.0001.bcf - matched-calls/varlociraptor-lancet/simulated-bwa.INS.1-30.0.001.bcf - matched-calls/varlociraptor-lancet/simulated-bwa.INS.1-30.0.01.bcf - matched-calls/varlociraptor-lancet/simulated-bwa.INS.1-30.0.05.bcf - matched-calls/varlociraptor-lancet/simulated-bwa.INS.1-30.0.1.bcf - matched-calls/varlociraptor-lancet/simulated-bwa.INS.1-30.0.2.bcf - matched-calls/varlociraptor-lancet/simulated-bwa.INS.1-30.0.3.bcf - matched-calls/varlociraptor-lancet/simulated-bwa.INS.1-30.0.4.bcf - matched-calls/varlociraptor-lancet/simulated-bwa.INS.1-30.0.5.bcf - matched-calls/varlociraptor-lancet/simulated-bwa.INS.1-30.0.6.bcf - matched-calls/varlociraptor-lancet/simulated-bwa.INS.1-30.0.7.bcf - matched-calls/varlociraptor-lancet/simulated-bwa.INS.1-30.0.8.bcf - matched-calls/varlociraptor-lancet/simulated-bwa.INS.1-30.0.9.bcf - matched-calls/varlociraptor-lancet/simulated-bwa.INS.30-100.1e-05.bcf - matched-calls/varlociraptor-lancet/simulated-bwa.INS.30-100.0.0001.bcf - matched-calls/varlociraptor-lancet/simulated-bwa.INS.30-100.0.001.bcf - matched-calls/varlociraptor-lancet/simulated-bwa.INS.30-100.0.01.bcf - matched-calls/varlociraptor-lancet/simulated-bwa.INS.30-100.0.05.bcf - matched-calls/varlociraptor-lancet/simulated-bwa.INS.30-100.0.1.bcf - matched-calls/varlociraptor-lancet/simulated-bwa.INS.30-100.0.2.bcf - matched-calls/varlociraptor-lancet/simulated-bwa.INS.30-100.0.3.bcf - matched-calls/varlociraptor-lancet/simulated-bwa.INS.30-100.0.4.bcf - matched-calls/varlociraptor-lancet/simulated-bwa.INS.30-100.0.5.bcf - matched-calls/varlociraptor-lancet/simulated-bwa.INS.30-100.0.6.bcf - matched-calls/varlociraptor-lancet/simulated-bwa.INS.30-100.0.7.bcf - matched-calls/varlociraptor-lancet/simulated-bwa.INS.30-100.0.8.bcf - matched-calls/varlociraptor-lancet/simulated-bwa.INS.30-100.0.9.bcf - matched-calls/varlociraptor-manta/simulated-bwa.INS.1-30.1e-05.bcf - matched-calls/varlociraptor-manta/simulated-bwa.INS.1-30.0.0001.bcf - matched-calls/varlociraptor-manta/simulated-bwa.INS.1-30.0.001.bcf - matched-calls/varlociraptor-manta/simulated-bwa.INS.1-30.0.01.bcf - matched-calls/varlociraptor-manta/simulated-bwa.INS.1-30.0.05.bcf - matched-calls/varlociraptor-manta/simulated-bwa.INS.1-30.0.1.bcf - matched-calls/varlociraptor-manta/simulated-bwa.INS.1-30.0.2.bcf - matched-calls/varlociraptor-manta/simulated-bwa.INS.1-30.0.3.bcf - matched-calls/varlociraptor-manta/simulated-bwa.INS.1-30.0.4.bcf - matched-calls/varlociraptor-manta/simulated-bwa.INS.1-30.0.5.bcf - matched-calls/varlociraptor-manta/simulated-bwa.INS.1-30.0.6.bcf - matched-calls/varlociraptor-manta/simulated-bwa.INS.1-30.0.7.bcf - matched-calls/varlociraptor-manta/simulated-bwa.INS.1-30.0.8.bcf - matched-calls/varlociraptor-manta/simulated-bwa.INS.1-30.0.9.bcf - matched-calls/varlociraptor-manta/simulated-bwa.INS.30-100.1e-05.bcf - matched-calls/varlociraptor-manta/simulated-bwa.INS.30-100.0.0001.bcf - matched-calls/varlociraptor-manta/simulated-bwa.INS.30-100.0.001.bcf - matched-calls/varlociraptor-manta/simulated-bwa.INS.30-100.0.01.bcf - matched-calls/varlociraptor-manta/simulated-bwa.INS.30-100.0.05.bcf - matched-calls/varlociraptor-manta/simulated-bwa.INS.30-100.0.1.bcf - matched-calls/varlociraptor-manta/simulated-bwa.INS.30-100.0.2.bcf - matched-calls/varlociraptor-manta/simulated-bwa.INS.30-100.0.3.bcf - matched-calls/varlociraptor-manta/simulated-bwa.INS.30-100.0.4.bcf - matched-calls/varlociraptor-manta/simulated-bwa.INS.30-100.0.5.bcf - matched-calls/varlociraptor-manta/simulated-bwa.INS.30-100.0.6.bcf - matched-calls/varlociraptor-manta/simulated-bwa.INS.30-100.0.7.bcf - matched-calls/varlociraptor-manta/simulated-bwa.INS.30-100.0.8.bcf - matched-calls/varlociraptor-manta/simulated-bwa.INS.30-100.0.9.bcf - matched-calls/varlociraptor-strelka/simulated-bwa.INS.1-30.1e-05.bcf - matched-calls/varlociraptor-strelka/simulated-bwa.INS.1-30.0.0001.bcf - matched-calls/varlociraptor-strelka/simulated-bwa.INS.1-30.0.001.bcf - matched-calls/varlociraptor-strelka/simulated-bwa.INS.1-30.0.01.bcf - matched-calls/varlociraptor-strelka/simulated-bwa.INS.1-30.0.05.bcf - matched-calls/varlociraptor-strelka/simulated-bwa.INS.1-30.0.1.bcf - matched-calls/varlociraptor-strelka/simulated-bwa.INS.1-30.0.2.bcf - matched-calls/varlociraptor-strelka/simulated-bwa.INS.1-30.0.3.bcf - matched-calls/varlociraptor-strelka/simulated-bwa.INS.1-30.0.4.bcf - matched-calls/varlociraptor-strelka/simulated-bwa.INS.1-30.0.5.bcf - matched-calls/varlociraptor-strelka/simulated-bwa.INS.1-30.0.6.bcf - matched-calls/varlociraptor-strelka/simulated-bwa.INS.1-30.0.7.bcf - matched-calls/varlociraptor-strelka/simulated-bwa.INS.1-30.0.8.bcf - matched-calls/varlociraptor-strelka/simulated-bwa.INS.1-30.0.9.bcf - matched-calls/varlociraptor-strelka/simulated-bwa.INS.30-100.1e-05.bcf - matched-calls/varlociraptor-strelka/simulated-bwa.INS.30-100.0.0001.bcf - matched-calls/varlociraptor-strelka/simulated-bwa.INS.30-100.0.001.bcf - matched-calls/varlociraptor-strelka/simulated-bwa.INS.30-100.0.01.bcf - matched-calls/varlociraptor-strelka/simulated-bwa.INS.30-100.0.05.bcf - matched-calls/varlociraptor-strelka/simulated-bwa.INS.30-100.0.1.bcf - matched-calls/varlociraptor-strelka/simulated-bwa.INS.30-100.0.2.bcf - matched-calls/varlociraptor-strelka/simulated-bwa.INS.30-100.0.3.bcf - matched-calls/varlociraptor-strelka/simulated-bwa.INS.30-100.0.4.bcf - matched-calls/varlociraptor-strelka/simulated-bwa.INS.30-100.0.5.bcf - matched-calls/varlociraptor-strelka/simulated-bwa.INS.30-100.0.6.bcf - matched-calls/varlociraptor-strelka/simulated-bwa.INS.30-100.0.7.bcf - matched-calls/varlociraptor-strelka/simulated-bwa.INS.30-100.0.8.bcf - matched-calls/varlociraptor-strelka/simulated-bwa.INS.30-100.0.9.bcf - matched-calls/varlociraptor-delly/simulated-bwa.DEL.1-30.1e-05.bcf - matched-calls/varlociraptor-delly/simulated-bwa.DEL.1-30.0.0001.bcf - matched-calls/varlociraptor-delly/simulated-bwa.DEL.1-30.0.001.bcf - matched-calls/varlociraptor-delly/simulated-bwa.DEL.1-30.0.01.bcf - matched-calls/varlociraptor-delly/simulated-bwa.DEL.1-30.0.05.bcf - matched-calls/varlociraptor-delly/simulated-bwa.DEL.1-30.0.1.bcf - matched-calls/varlociraptor-delly/simulated-bwa.DEL.1-30.0.2.bcf - matched-calls/varlociraptor-delly/simulated-bwa.DEL.1-30.0.3.bcf - matched-calls/varlociraptor-delly/simulated-bwa.DEL.1-30.0.4.bcf - matched-calls/varlociraptor-delly/simulated-bwa.DEL.1-30.0.5.bcf - matched-calls/varlociraptor-delly/simulated-bwa.DEL.1-30.0.6.bcf - matched-calls/varlociraptor-delly/simulated-bwa.DEL.1-30.0.7.bcf - matched-calls/varlociraptor-delly/simulated-bwa.DEL.1-30.0.8.bcf - matched-calls/varlociraptor-delly/simulated-bwa.DEL.1-30.0.9.bcf - matched-calls/varlociraptor-delly/simulated-bwa.DEL.30-50.1e-05.bcf - matched-calls/varlociraptor-delly/simulated-bwa.DEL.30-50.0.0001.bcf - matched-calls/varlociraptor-delly/simulated-bwa.DEL.30-50.0.001.bcf - matched-calls/varlociraptor-delly/simulated-bwa.DEL.30-50.0.01.bcf - matched-calls/varlociraptor-delly/simulated-bwa.DEL.30-50.0.05.bcf - matched-calls/varlociraptor-delly/simulated-bwa.DEL.30-50.0.1.bcf - matched-calls/varlociraptor-delly/simulated-bwa.DEL.30-50.0.2.bcf - matched-calls/varlociraptor-delly/simulated-bwa.DEL.30-50.0.3.bcf - matched-calls/varlociraptor-delly/simulated-bwa.DEL.30-50.0.4.bcf - matched-calls/varlociraptor-delly/simulated-bwa.DEL.30-50.0.5.bcf - matched-calls/varlociraptor-delly/simulated-bwa.DEL.30-50.0.6.bcf - matched-calls/varlociraptor-delly/simulated-bwa.DEL.30-50.0.7.bcf - matched-calls/varlociraptor-delly/simulated-bwa.DEL.30-50.0.8.bcf - matched-calls/varlociraptor-delly/simulated-bwa.DEL.30-50.0.9.bcf - matched-calls/varlociraptor-delly/simulated-bwa.DEL.50-100.1e-05.bcf - matched-calls/varlociraptor-delly/simulated-bwa.DEL.50-100.0.0001.bcf - matched-calls/varlociraptor-delly/simulated-bwa.DEL.50-100.0.001.bcf - matched-calls/varlociraptor-delly/simulated-bwa.DEL.50-100.0.01.bcf - matched-calls/varlociraptor-delly/simulated-bwa.DEL.50-100.0.05.bcf - matched-calls/varlociraptor-delly/simulated-bwa.DEL.50-100.0.1.bcf - matched-calls/varlociraptor-delly/simulated-bwa.DEL.50-100.0.2.bcf - matched-calls/varlociraptor-delly/simulated-bwa.DEL.50-100.0.3.bcf - matched-calls/varlociraptor-delly/simulated-bwa.DEL.50-100.0.4.bcf - matched-calls/varlociraptor-delly/simulated-bwa.DEL.50-100.0.5.bcf - matched-calls/varlociraptor-delly/simulated-bwa.DEL.50-100.0.6.bcf - matched-calls/varlociraptor-delly/simulated-bwa.DEL.50-100.0.7.bcf - matched-calls/varlociraptor-delly/simulated-bwa.DEL.50-100.0.8.bcf - matched-calls/varlociraptor-delly/simulated-bwa.DEL.50-100.0.9.bcf - matched-calls/varlociraptor-delly/simulated-bwa.DEL.100-250.1e-05.bcf - matched-calls/varlociraptor-delly/simulated-bwa.DEL.100-250.0.0001.bcf - matched-calls/varlociraptor-delly/simulated-bwa.DEL.100-250.0.001.bcf - matched-calls/varlociraptor-delly/simulated-bwa.DEL.100-250.0.01.bcf - matched-calls/varlociraptor-delly/simulated-bwa.DEL.100-250.0.05.bcf - matched-calls/varlociraptor-delly/simulated-bwa.DEL.100-250.0.1.bcf - matched-calls/varlociraptor-delly/simulated-bwa.DEL.100-250.0.2.bcf - matched-calls/varlociraptor-delly/simulated-bwa.DEL.100-250.0.3.bcf - matched-calls/varlociraptor-delly/simulated-bwa.DEL.100-250.0.4.bcf - matched-calls/varlociraptor-delly/simulated-bwa.DEL.100-250.0.5.bcf - matched-calls/varlociraptor-delly/simulated-bwa.DEL.100-250.0.6.bcf - matched-calls/varlociraptor-delly/simulated-bwa.DEL.100-250.0.7.bcf - matched-calls/varlociraptor-delly/simulated-bwa.DEL.100-250.0.8.bcf - matched-calls/varlociraptor-delly/simulated-bwa.DEL.100-250.0.9.bcf - matched-calls/varlociraptor-lancet/simulated-bwa.DEL.1-30.1e-05.bcf - matched-calls/varlociraptor-lancet/simulated-bwa.DEL.1-30.0.0001.bcf - matched-calls/varlociraptor-lancet/simulated-bwa.DEL.1-30.0.001.bcf - matched-calls/varlociraptor-lancet/simulated-bwa.DEL.1-30.0.01.bcf - matched-calls/varlociraptor-lancet/simulated-bwa.DEL.1-30.0.05.bcf - matched-calls/varlociraptor-lancet/simulated-bwa.DEL.1-30.0.1.bcf - matched-calls/varlociraptor-lancet/simulated-bwa.DEL.1-30.0.2.bcf - matched-calls/varlociraptor-lancet/simulated-bwa.DEL.1-30.0.3.bcf - matched-calls/varlociraptor-lancet/simulated-bwa.DEL.1-30.0.4.bcf - matched-calls/varlociraptor-lancet/simulated-bwa.DEL.1-30.0.5.bcf - matched-calls/varlociraptor-lancet/simulated-bwa.DEL.1-30.0.6.bcf - matched-calls/varlociraptor-lancet/simulated-bwa.DEL.1-30.0.7.bcf - matched-calls/varlociraptor-lancet/simulated-bwa.DEL.1-30.0.8.bcf - matched-calls/varlociraptor-lancet/simulated-bwa.DEL.1-30.0.9.bcf - matched-calls/varlociraptor-lancet/simulated-bwa.DEL.30-50.1e-05.bcf - matched-calls/varlociraptor-lancet/simulated-bwa.DEL.30-50.0.0001.bcf - matched-calls/varlociraptor-lancet/simulated-bwa.DEL.30-50.0.001.bcf - matched-calls/varlociraptor-lancet/simulated-bwa.DEL.30-50.0.01.bcf - matched-calls/varlociraptor-lancet/simulated-bwa.DEL.30-50.0.05.bcf - matched-calls/varlociraptor-lancet/simulated-bwa.DEL.30-50.0.1.bcf - matched-calls/varlociraptor-lancet/simulated-bwa.DEL.30-50.0.2.bcf - matched-calls/varlociraptor-lancet/simulated-bwa.DEL.30-50.0.3.bcf - matched-calls/varlociraptor-lancet/simulated-bwa.DEL.30-50.0.4.bcf - matched-calls/varlociraptor-lancet/simulated-bwa.DEL.30-50.0.5.bcf - matched-calls/varlociraptor-lancet/simulated-bwa.DEL.30-50.0.6.bcf - matched-calls/varlociraptor-lancet/simulated-bwa.DEL.30-50.0.7.bcf - matched-calls/varlociraptor-lancet/simulated-bwa.DEL.30-50.0.8.bcf - matched-calls/varlociraptor-lancet/simulated-bwa.DEL.30-50.0.9.bcf - matched-calls/varlociraptor-lancet/simulated-bwa.DEL.50-100.1e-05.bcf - matched-calls/varlociraptor-lancet/simulated-bwa.DEL.50-100.0.0001.bcf - matched-calls/varlociraptor-lancet/simulated-bwa.DEL.50-100.0.001.bcf - matched-calls/varlociraptor-lancet/simulated-bwa.DEL.50-100.0.01.bcf - matched-calls/varlociraptor-lancet/simulated-bwa.DEL.50-100.0.05.bcf - matched-calls/varlociraptor-lancet/simulated-bwa.DEL.50-100.0.1.bcf - matched-calls/varlociraptor-lancet/simulated-bwa.DEL.50-100.0.2.bcf - matched-calls/varlociraptor-lancet/simulated-bwa.DEL.50-100.0.3.bcf - matched-calls/varlociraptor-lancet/simulated-bwa.DEL.50-100.0.4.bcf - matched-calls/varlociraptor-lancet/simulated-bwa.DEL.50-100.0.5.bcf - matched-calls/varlociraptor-lancet/simulated-bwa.DEL.50-100.0.6.bcf - matched-calls/varlociraptor-lancet/simulated-bwa.DEL.50-100.0.7.bcf - matched-calls/varlociraptor-lancet/simulated-bwa.DEL.50-100.0.8.bcf - matched-calls/varlociraptor-lancet/simulated-bwa.DEL.50-100.0.9.bcf - matched-calls/varlociraptor-lancet/simulated-bwa.DEL.100-250.1e-05.bcf - matched-calls/varlociraptor-lancet/simulated-bwa.DEL.100-250.0.0001.bcf - matched-calls/varlociraptor-lancet/simulated-bwa.DEL.100-250.0.001.bcf - matched-calls/varlociraptor-lancet/simulated-bwa.DEL.100-250.0.01.bcf - matched-calls/varlociraptor-lancet/simulated-bwa.DEL.100-250.0.05.bcf - matched-calls/varlociraptor-lancet/simulated-bwa.DEL.100-250.0.1.bcf - matched-calls/varlociraptor-lancet/simulated-bwa.DEL.100-250.0.2.bcf - matched-calls/varlociraptor-lancet/simulated-bwa.DEL.100-250.0.3.bcf - matched-calls/varlociraptor-lancet/simulated-bwa.DEL.100-250.0.4.bcf - matched-calls/varlociraptor-lancet/simulated-bwa.DEL.100-250.0.5.bcf - matched-calls/varlociraptor-lancet/simulated-bwa.DEL.100-250.0.6.bcf - matched-calls/varlociraptor-lancet/simulated-bwa.DEL.100-250.0.7.bcf - matched-calls/varlociraptor-lancet/simulated-bwa.DEL.100-250.0.8.bcf - matched-calls/varlociraptor-lancet/simulated-bwa.DEL.100-250.0.9.bcf - matched-calls/varlociraptor-manta/simulated-bwa.DEL.1-30.1e-05.bcf - matched-calls/varlociraptor-manta/simulated-bwa.DEL.1-30.0.0001.bcf - matched-calls/varlociraptor-manta/simulated-bwa.DEL.1-30.0.001.bcf - matched-calls/varlociraptor-manta/simulated-bwa.DEL.1-30.0.01.bcf - matched-calls/varlociraptor-manta/simulated-bwa.DEL.1-30.0.05.bcf - matched-calls/varlociraptor-manta/simulated-bwa.DEL.1-30.0.1.bcf - matched-calls/varlociraptor-manta/simulated-bwa.DEL.1-30.0.2.bcf - matched-calls/varlociraptor-manta/simulated-bwa.DEL.1-30.0.3.bcf - matched-calls/varlociraptor-manta/simulated-bwa.DEL.1-30.0.4.bcf - matched-calls/varlociraptor-manta/simulated-bwa.DEL.1-30.0.5.bcf - matched-calls/varlociraptor-manta/simulated-bwa.DEL.1-30.0.6.bcf - matched-calls/varlociraptor-manta/simulated-bwa.DEL.1-30.0.7.bcf - matched-calls/varlociraptor-manta/simulated-bwa.DEL.1-30.0.8.bcf - matched-calls/varlociraptor-manta/simulated-bwa.DEL.1-30.0.9.bcf - matched-calls/varlociraptor-manta/simulated-bwa.DEL.30-50.1e-05.bcf - matched-calls/varlociraptor-manta/simulated-bwa.DEL.30-50.0.0001.bcf - matched-calls/varlociraptor-manta/simulated-bwa.DEL.30-50.0.001.bcf - matched-calls/varlociraptor-manta/simulated-bwa.DEL.30-50.0.01.bcf - matched-calls/varlociraptor-manta/simulated-bwa.DEL.30-50.0.05.bcf - matched-calls/varlociraptor-manta/simulated-bwa.DEL.30-50.0.1.bcf - matched-calls/varlociraptor-manta/simulated-bwa.DEL.30-50.0.2.bcf - matched-calls/varlociraptor-manta/simulated-bwa.DEL.30-50.0.3.bcf - matched-calls/varlociraptor-manta/simulated-bwa.DEL.30-50.0.4.bcf - matched-calls/varlociraptor-manta/simulated-bwa.DEL.30-50.0.5.bcf - matched-calls/varlociraptor-manta/simulated-bwa.DEL.30-50.0.6.bcf - matched-calls/varlociraptor-manta/simulated-bwa.DEL.30-50.0.7.bcf - matched-calls/varlociraptor-manta/simulated-bwa.DEL.30-50.0.8.bcf - matched-calls/varlociraptor-manta/simulated-bwa.DEL.30-50.0.9.bcf - matched-calls/varlociraptor-manta/simulated-bwa.DEL.50-100.1e-05.bcf - matched-calls/varlociraptor-manta/simulated-bwa.DEL.50-100.0.0001.bcf - matched-calls/varlociraptor-manta/simulated-bwa.DEL.50-100.0.001.bcf - matched-calls/varlociraptor-manta/simulated-bwa.DEL.50-100.0.01.bcf - matched-calls/varlociraptor-manta/simulated-bwa.DEL.50-100.0.05.bcf - matched-calls/varlociraptor-manta/simulated-bwa.DEL.50-100.0.1.bcf - matched-calls/varlociraptor-manta/simulated-bwa.DEL.50-100.0.2.bcf - matched-calls/varlociraptor-manta/simulated-bwa.DEL.50-100.0.3.bcf - matched-calls/varlociraptor-manta/simulated-bwa.DEL.50-100.0.4.bcf - matched-calls/varlociraptor-manta/simulated-bwa.DEL.50-100.0.5.bcf - matched-calls/varlociraptor-manta/simulated-bwa.DEL.50-100.0.6.bcf - matched-calls/varlociraptor-manta/simulated-bwa.DEL.50-100.0.7.bcf - matched-calls/varlociraptor-manta/simulated-bwa.DEL.50-100.0.8.bcf - matched-calls/varlociraptor-manta/simulated-bwa.DEL.50-100.0.9.bcf - matched-calls/varlociraptor-manta/simulated-bwa.DEL.100-250.1e-05.bcf - matched-calls/varlociraptor-manta/simulated-bwa.DEL.100-250.0.0001.bcf - matched-calls/varlociraptor-manta/simulated-bwa.DEL.100-250.0.001.bcf - matched-calls/varlociraptor-manta/simulated-bwa.DEL.100-250.0.01.bcf - matched-calls/varlociraptor-manta/simulated-bwa.DEL.100-250.0.05.bcf - matched-calls/varlociraptor-manta/simulated-bwa.DEL.100-250.0.1.bcf - matched-calls/varlociraptor-manta/simulated-bwa.DEL.100-250.0.2.bcf - matched-calls/varlociraptor-manta/simulated-bwa.DEL.100-250.0.3.bcf - matched-calls/varlociraptor-manta/simulated-bwa.DEL.100-250.0.4.bcf - matched-calls/varlociraptor-manta/simulated-bwa.DEL.100-250.0.5.bcf - matched-calls/varlociraptor-manta/simulated-bwa.DEL.100-250.0.6.bcf - matched-calls/varlociraptor-manta/simulated-bwa.DEL.100-250.0.7.bcf - matched-calls/varlociraptor-manta/simulated-bwa.DEL.100-250.0.8.bcf - matched-calls/varlociraptor-manta/simulated-bwa.DEL.100-250.0.9.bcf - matched-calls/varlociraptor-strelka/simulated-bwa.DEL.1-30.1e-05.bcf - matched-calls/varlociraptor-strelka/simulated-bwa.DEL.1-30.0.0001.bcf - matched-calls/varlociraptor-strelka/simulated-bwa.DEL.1-30.0.001.bcf - matched-calls/varlociraptor-strelka/simulated-bwa.DEL.1-30.0.01.bcf - matched-calls/varlociraptor-strelka/simulated-bwa.DEL.1-30.0.05.bcf - matched-calls/varlociraptor-strelka/simulated-bwa.DEL.1-30.0.1.bcf - matched-calls/varlociraptor-strelka/simulated-bwa.DEL.1-30.0.2.bcf - matched-calls/varlociraptor-strelka/simulated-bwa.DEL.1-30.0.3.bcf - matched-calls/varlociraptor-strelka/simulated-bwa.DEL.1-30.0.4.bcf - matched-calls/varlociraptor-strelka/simulated-bwa.DEL.1-30.0.5.bcf - matched-calls/varlociraptor-strelka/simulated-bwa.DEL.1-30.0.6.bcf - matched-calls/varlociraptor-strelka/simulated-bwa.DEL.1-30.0.7.bcf - matched-calls/varlociraptor-strelka/simulated-bwa.DEL.1-30.0.8.bcf - matched-calls/varlociraptor-strelka/simulated-bwa.DEL.1-30.0.9.bcf - matched-calls/varlociraptor-strelka/simulated-bwa.DEL.30-50.1e-05.bcf - matched-calls/varlociraptor-strelka/simulated-bwa.DEL.30-50.0.0001.bcf - matched-calls/varlociraptor-strelka/simulated-bwa.DEL.30-50.0.001.bcf - matched-calls/varlociraptor-strelka/simulated-bwa.DEL.30-50.0.01.bcf - matched-calls/varlociraptor-strelka/simulated-bwa.DEL.30-50.0.05.bcf - matched-calls/varlociraptor-strelka/simulated-bwa.DEL.30-50.0.1.bcf - matched-calls/varlociraptor-strelka/simulated-bwa.DEL.30-50.0.2.bcf - matched-calls/varlociraptor-strelka/simulated-bwa.DEL.30-50.0.3.bcf - matched-calls/varlociraptor-strelka/simulated-bwa.DEL.30-50.0.4.bcf - matched-calls/varlociraptor-strelka/simulated-bwa.DEL.30-50.0.5.bcf - matched-calls/varlociraptor-strelka/simulated-bwa.DEL.30-50.0.6.bcf - matched-calls/varlociraptor-strelka/simulated-bwa.DEL.30-50.0.7.bcf - matched-calls/varlociraptor-strelka/simulated-bwa.DEL.30-50.0.8.bcf - matched-calls/varlociraptor-strelka/simulated-bwa.DEL.30-50.0.9.bcf - matched-calls/varlociraptor-strelka/simulated-bwa.DEL.50-100.1e-05.bcf - matched-calls/varlociraptor-strelka/simulated-bwa.DEL.50-100.0.0001.bcf - matched-calls/varlociraptor-strelka/simulated-bwa.DEL.50-100.0.001.bcf - matched-calls/varlociraptor-strelka/simulated-bwa.DEL.50-100.0.01.bcf - matched-calls/varlociraptor-strelka/simulated-bwa.DEL.50-100.0.05.bcf - matched-calls/varlociraptor-strelka/simulated-bwa.DEL.50-100.0.1.bcf - matched-calls/varlociraptor-strelka/simulated-bwa.DEL.50-100.0.2.bcf - matched-calls/varlociraptor-strelka/simulated-bwa.DEL.50-100.0.3.bcf - matched-calls/varlociraptor-strelka/simulated-bwa.DEL.50-100.0.4.bcf - matched-calls/varlociraptor-strelka/simulated-bwa.DEL.50-100.0.5.bcf - matched-calls/varlociraptor-strelka/simulated-bwa.DEL.50-100.0.6.bcf - matched-calls/varlociraptor-strelka/simulated-bwa.DEL.50-100.0.7.bcf - matched-calls/varlociraptor-strelka/simulated-bwa.DEL.50-100.0.8.bcf - matched-calls/varlociraptor-strelka/simulated-bwa.DEL.50-100.0.9.bcf - matched-calls/varlociraptor-strelka/simulated-bwa.DEL.100-250.1e-05.bcf - matched-calls/varlociraptor-strelka/simulated-bwa.DEL.100-250.0.0001.bcf - matched-calls/varlociraptor-strelka/simulated-bwa.DEL.100-250.0.001.bcf - matched-calls/varlociraptor-strelka/simulated-bwa.DEL.100-250.0.01.bcf - matched-calls/varlociraptor-strelka/simulated-bwa.DEL.100-250.0.05.bcf - matched-calls/varlociraptor-strelka/simulated-bwa.DEL.100-250.0.1.bcf - matched-calls/varlociraptor-strelka/simulated-bwa.DEL.100-250.0.2.bcf - matched-calls/varlociraptor-strelka/simulated-bwa.DEL.100-250.0.3.bcf - matched-calls/varlociraptor-strelka/simulated-bwa.DEL.100-250.0.4.bcf - matched-calls/varlociraptor-strelka/simulated-bwa.DEL.100-250.0.5.bcf - matched-calls/varlociraptor-strelka/simulated-bwa.DEL.100-250.0.6.bcf - matched-calls/varlociraptor-strelka/simulated-bwa.DEL.100-250.0.7.bcf - matched-calls/varlociraptor-strelka/simulated-bwa.DEL.100-250.0.8.bcf - matched-calls/varlociraptor-strelka/simulated-bwa.DEL.100-250.0.9.bcf - matched-calls/varlociraptor-delly/simulated-bwa.INS.1-250.1.0.bcf - matched-calls/varlociraptor-lancet/simulated-bwa.INS.1-250.1.0.bcf - matched-calls/varlociraptor-manta/simulated-bwa.INS.1-250.1.0.bcf - matched-calls/varlociraptor-strelka/simulated-bwa.INS.1-250.1.0.bcf - matched-calls/varlociraptor-delly/simulated-bwa.DEL.1-250.1.0.bcf - matched-calls/varlociraptor-lancet/simulated-bwa.DEL.1-250.1.0.bcf - matched-calls/varlociraptor-manta/simulated-bwa.DEL.1-250.1.0.bcf - matched-calls/varlociraptor-strelka/simulated-bwa.DEL.1-250.1.0.bcf |  | - rust-bio-tools =0.5.0 - bedtools =2.27.1 - bcftools =1.8 | |  |  | | --- | --- | | ``` 1 ``` | ``` bcftools view {params.regions} {input.calls} | rbt vcf-match {params.match} {input.truth} > {output} ``` | |
| match\_other\_calls | 23 | - matched-calls/default-neusomatic/simulated-bwa.all.bcf - matched-calls/adhoc-neusomatic/simulated-bwa.all.bcf - matched-calls/adhoc-delly/simulated-bwa.all.bcf - matched-calls/default-neusomatic/synthetic-5.all.bcf - matched-calls/default-lancet/synthetic-5.all.bcf - matched-calls/default-manta/synthetic-5.all.bcf - matched-calls/default-strelka/synthetic-5.all.bcf - matched-calls/adhoc-neusomatic/synthetic-5.all.bcf - matched-calls/adhoc-delly/synthetic-5.all.bcf - matched-calls/adhoc-lancet/synthetic-5.all.bcf - matched-calls/adhoc-manta/synthetic-5.all.bcf - matched-calls/adhoc-strelka/synthetic-5.all.bcf - matched-calls/adhoc-bpi/synthetic-5.all.bcf - matched-calls/default-neusomatic/synthetic-20.all.bcf - matched-calls/default-lancet/synthetic-20.all.bcf - matched-calls/default-manta/synthetic-20.all.bcf - matched-calls/default-strelka/synthetic-20.all.bcf - matched-calls/adhoc-neusomatic/synthetic-20.all.bcf - matched-calls/adhoc-delly/synthetic-20.all.bcf - matched-calls/adhoc-lancet/synthetic-20.all.bcf - matched-calls/adhoc-manta/synthetic-20.all.bcf - matched-calls/adhoc-strelka/synthetic-20.all.bcf - matched-calls/adhoc-bpi/synthetic-20.all.bcf |  | - rust-bio-tools =0.9.0 - bedtools =2.27.1 - bcftools =1.8 | |  |  | | --- | --- | | ``` 1 ``` | ``` bcftools view {params.regions} {input.calls} | rbt vcf-match {params.match} {input.truth} > {output} ``` | |
| match\_other\_calls | 7 | - matched-calls/default-lancet/simulated-bwa.all.bcf - matched-calls/default-manta/simulated-bwa.all.bcf - matched-calls/default-strelka/simulated-bwa.all.bcf - matched-calls/adhoc-lancet/simulated-bwa.all.bcf - matched-calls/adhoc-manta/simulated-bwa.all.bcf - matched-calls/adhoc-strelka/simulated-bwa.all.bcf - matched-calls/adhoc-bpi/simulated-bwa.all.bcf |  |  | |  |  | | --- | --- | | ``` 1 ``` | ``` bcftools view {params.regions} {input.calls} | rbt vcf-match {params.match} {input.truth} > {output} ``` | |
| concordance\_to\_tsv | 18 | - concordance/varlociraptor-neusomatic-0.9/colo1.0-vs-1.tsv - concordance/varlociraptor-neusomatic-0.9/colo1.1-vs-2.tsv - concordance/varlociraptor-neusomatic-0.9/colo1.2-vs-0.tsv - concordance/varlociraptor-neusomatic-0.9/colo1.2-vs-3.tsv - concordance/varlociraptor-neusomatic-0.9/colo1.3-vs-0.tsv - concordance/varlociraptor-neusomatic-0.9/colo1.3-vs-1.tsv - concordance/varlociraptor-neusomatic-0.98/colo1.0-vs-1.tsv - concordance/varlociraptor-neusomatic-0.98/colo1.1-vs-2.tsv - concordance/varlociraptor-neusomatic-0.98/colo1.2-vs-0.tsv - concordance/varlociraptor-neusomatic-0.98/colo1.2-vs-3.tsv - concordance/varlociraptor-neusomatic-0.98/colo1.3-vs-0.tsv - concordance/varlociraptor-neusomatic-0.98/colo1.3-vs-1.tsv - concordance/adhoc-neusomatic-default/colo1.0-vs-1.tsv - concordance/adhoc-neusomatic-default/colo1.1-vs-2.tsv - concordance/adhoc-neusomatic-default/colo1.2-vs-0.tsv - concordance/adhoc-neusomatic-default/colo1.2-vs-3.tsv - concordance/adhoc-neusomatic-default/colo1.3-vs-0.tsv - concordance/adhoc-neusomatic-default/colo1.3-vs-1.tsv |  | - rust-bio-tools =0.9.0 - bedtools =2.27.1 - bcftools =1.8 | |  |  | | --- | --- | | ``` 1 ``` | ``` rbt vcf-to-txt {params.gt} {params.tags} --info MATCHING < {input} > {output} ``` | |
| concordance\_to\_tsv | 60 | - concordance/varlociraptor-delly-0.9/colo1.0-vs-1.tsv - concordance/varlociraptor-delly-0.9/colo1.1-vs-2.tsv - concordance/varlociraptor-delly-0.9/colo1.2-vs-0.tsv - concordance/varlociraptor-delly-0.9/colo1.2-vs-3.tsv - concordance/varlociraptor-delly-0.9/colo1.3-vs-0.tsv - concordance/varlociraptor-delly-0.9/colo1.3-vs-1.tsv - concordance/varlociraptor-lancet-0.9/colo1.0-vs-1.tsv - concordance/varlociraptor-lancet-0.9/colo1.1-vs-2.tsv - concordance/varlociraptor-lancet-0.9/colo1.2-vs-0.tsv - concordance/varlociraptor-lancet-0.9/colo1.2-vs-3.tsv - concordance/varlociraptor-lancet-0.9/colo1.3-vs-0.tsv - concordance/varlociraptor-lancet-0.9/colo1.3-vs-1.tsv - concordance/varlociraptor-manta-0.9/colo1.0-vs-1.tsv - concordance/varlociraptor-manta-0.9/colo1.1-vs-2.tsv - concordance/varlociraptor-manta-0.9/colo1.2-vs-0.tsv - concordance/varlociraptor-manta-0.9/colo1.2-vs-3.tsv - concordance/varlociraptor-manta-0.9/colo1.3-vs-0.tsv - concordance/varlociraptor-manta-0.9/colo1.3-vs-1.tsv - concordance/varlociraptor-strelka-0.9/colo1.0-vs-1.tsv - concordance/varlociraptor-strelka-0.9/colo1.1-vs-2.tsv - concordance/varlociraptor-strelka-0.9/colo1.2-vs-0.tsv - concordance/varlociraptor-strelka-0.9/colo1.2-vs-3.tsv - concordance/varlociraptor-strelka-0.9/colo1.3-vs-0.tsv - concordance/varlociraptor-strelka-0.9/colo1.3-vs-1.tsv - concordance/varlociraptor-bpi-0.9/colo1.0-vs-1.tsv - concordance/varlociraptor-bpi-0.9/colo1.1-vs-2.tsv - concordance/varlociraptor-bpi-0.9/colo1.2-vs-0.tsv - concordance/varlociraptor-bpi-0.9/colo1.2-vs-3.tsv - concordance/varlociraptor-bpi-0.9/colo1.3-vs-0.tsv - concordance/varlociraptor-bpi-0.9/colo1.3-vs-1.tsv - concordance/varlociraptor-delly-0.98/colo1.0-vs-1.tsv - concordance/varlociraptor-delly-0.98/colo1.1-vs-2.tsv - concordance/varlociraptor-delly-0.98/colo1.2-vs-0.tsv - concordance/varlociraptor-delly-0.98/colo1.2-vs-3.tsv - concordance/varlociraptor-delly-0.98/colo1.3-vs-0.tsv - concordance/varlociraptor-delly-0.98/colo1.3-vs-1.tsv - concordance/varlociraptor-lancet-0.98/colo1.0-vs-1.tsv - concordance/varlociraptor-lancet-0.98/colo1.1-vs-2.tsv - concordance/varlociraptor-lancet-0.98/colo1.2-vs-0.tsv - concordance/varlociraptor-lancet-0.98/colo1.2-vs-3.tsv - concordance/varlociraptor-lancet-0.98/colo1.3-vs-0.tsv - concordance/varlociraptor-lancet-0.98/colo1.3-vs-1.tsv - concordance/varlociraptor-manta-0.98/colo1.0-vs-1.tsv - concordance/varlociraptor-manta-0.98/colo1.1-vs-2.tsv - concordance/varlociraptor-manta-0.98/colo1.2-vs-0.tsv - concordance/varlociraptor-manta-0.98/colo1.2-vs-3.tsv - concordance/varlociraptor-manta-0.98/colo1.3-vs-0.tsv - concordance/varlociraptor-manta-0.98/colo1.3-vs-1.tsv - concordance/varlociraptor-strelka-0.98/colo1.0-vs-1.tsv - concordance/varlociraptor-strelka-0.98/colo1.1-vs-2.tsv - concordance/varlociraptor-strelka-0.98/colo1.2-vs-0.tsv - concordance/varlociraptor-strelka-0.98/colo1.2-vs-3.tsv - concordance/varlociraptor-strelka-0.98/colo1.3-vs-0.tsv - concordance/varlociraptor-strelka-0.98/colo1.3-vs-1.tsv - concordance/varlociraptor-bpi-0.98/colo1.0-vs-1.tsv - concordance/varlociraptor-bpi-0.98/colo1.1-vs-2.tsv - concordance/varlociraptor-bpi-0.98/colo1.2-vs-0.tsv - concordance/varlociraptor-bpi-0.98/colo1.2-vs-3.tsv - concordance/varlociraptor-bpi-0.98/colo1.3-vs-0.tsv - concordance/varlociraptor-bpi-0.98/colo1.3-vs-1.tsv |  | - rust-bio-tools =0.5.0 - bedtools =2.27.1 - bcftools =1.8 | |  |  | | --- | --- | | ``` 1 ``` | ``` rbt vcf-to-txt {params.gt} {params.tags} --info MATCHING < {input} > {output} ``` | |
| concordance\_to\_tsv | 30 | - concordance/adhoc-delly-default/colo1.0-vs-1.tsv - concordance/adhoc-delly-default/colo1.1-vs-2.tsv - concordance/adhoc-delly-default/colo1.2-vs-0.tsv - concordance/adhoc-delly-default/colo1.2-vs-3.tsv - concordance/adhoc-delly-default/colo1.3-vs-0.tsv - concordance/adhoc-delly-default/colo1.3-vs-1.tsv - concordance/adhoc-lancet-default/colo1.0-vs-1.tsv - concordance/adhoc-lancet-default/colo1.1-vs-2.tsv - concordance/adhoc-lancet-default/colo1.2-vs-0.tsv - concordance/adhoc-lancet-default/colo1.2-vs-3.tsv - concordance/adhoc-lancet-default/colo1.3-vs-0.tsv - concordance/adhoc-lancet-default/colo1.3-vs-1.tsv - concordance/adhoc-manta-default/colo1.0-vs-1.tsv - concordance/adhoc-manta-default/colo1.1-vs-2.tsv - concordance/adhoc-manta-default/colo1.2-vs-0.tsv - concordance/adhoc-manta-default/colo1.2-vs-3.tsv - concordance/adhoc-manta-default/colo1.3-vs-0.tsv - concordance/adhoc-manta-default/colo1.3-vs-1.tsv - concordance/adhoc-strelka-default/colo1.0-vs-1.tsv - concordance/adhoc-strelka-default/colo1.1-vs-2.tsv - concordance/adhoc-strelka-default/colo1.2-vs-0.tsv - concordance/adhoc-strelka-default/colo1.2-vs-3.tsv - concordance/adhoc-strelka-default/colo1.3-vs-0.tsv - concordance/adhoc-strelka-default/colo1.3-vs-1.tsv - concordance/adhoc-bpi-default/colo1.0-vs-1.tsv - concordance/adhoc-bpi-default/colo1.1-vs-2.tsv - concordance/adhoc-bpi-default/colo1.2-vs-0.tsv - concordance/adhoc-bpi-default/colo1.2-vs-3.tsv - concordance/adhoc-bpi-default/colo1.3-vs-0.tsv - concordance/adhoc-bpi-default/colo1.3-vs-1.tsv |  | - rust-bio-tools =0.2.5 - bedtools =2.27.1 - bcftools =1.8 | |  |  | | --- | --- | | ``` 1 ``` | ``` rbt vcf-to-txt {params.gt} {params.tags} --info MATCHING < {input} > {output} ``` | |
| varlociraptor\_all\_calls\_to\_tsv | 4 | - varlociraptor-neusomatic/COLO\_829-GSC.all.tsv - varlociraptor-neusomatic/COLO\_829-Ill.all.tsv - varlociraptor-neusomatic/COLO\_829-TGen.all.tsv - varlociraptor-neusomatic/COLO\_829-EBI.all.tsv |  | - rust-bio-tools =0.9.0 - bedtools =2.27.1 - bcftools =1.8 | |  |  | | --- | --- | | ``` 1 ``` | ``` rbt vcf-to-txt {params.gt} {params.tags} < {input} > {output} ``` | |
| varlociraptor\_all\_calls\_to\_tsv | 20 | - varlociraptor-delly/COLO\_829-GSC.all.tsv - varlociraptor-delly/COLO\_829-Ill.all.tsv - varlociraptor-delly/COLO\_829-TGen.all.tsv - varlociraptor-delly/COLO\_829-EBI.all.tsv - varlociraptor-lancet/COLO\_829-GSC.all.tsv - varlociraptor-lancet/COLO\_829-Ill.all.tsv - varlociraptor-lancet/COLO\_829-TGen.all.tsv - varlociraptor-lancet/COLO\_829-EBI.all.tsv - varlociraptor-manta/COLO\_829-GSC.all.tsv - varlociraptor-manta/COLO\_829-Ill.all.tsv - varlociraptor-manta/COLO\_829-TGen.all.tsv - varlociraptor-manta/COLO\_829-EBI.all.tsv - varlociraptor-strelka/COLO\_829-GSC.all.tsv - varlociraptor-strelka/COLO\_829-Ill.all.tsv - varlociraptor-strelka/COLO\_829-TGen.all.tsv - varlociraptor-strelka/COLO\_829-EBI.all.tsv - varlociraptor-bpi/COLO\_829-GSC.all.tsv - varlociraptor-bpi/COLO\_829-Ill.all.tsv - varlociraptor-bpi/COLO\_829-TGen.all.tsv - varlociraptor-bpi/COLO\_829-EBI.all.tsv |  | - rust-bio-tools =0.5.0 - bedtools =2.27.1 - bcftools =1.8 | |  |  | | --- | --- | | ``` 1 ``` | ``` rbt vcf-to-txt {params.gt} {params.tags} < {input} > {output} ``` | |
| varlociraptor\_control\_fdr | 122 | - varlociraptor-neusomatic/simulated-bwa.INS.1-30.1.0.bcf - varlociraptor-neusomatic/simulated-bwa.INS.30-100.1.0.bcf - varlociraptor-neusomatic/synthetic-5.INS.1-250.1.0.bcf - varlociraptor-delly/synthetic-5.INS.1-250.1.0.bcf - varlociraptor-lancet/synthetic-5.INS.1-250.1.0.bcf - varlociraptor-manta/synthetic-5.INS.1-250.1.0.bcf - varlociraptor-strelka/synthetic-5.INS.1-250.1.0.bcf - varlociraptor-neusomatic/synthetic-20.INS.1-30.1.0.bcf - varlociraptor-neusomatic/synthetic-20.INS.30-250.1.0.bcf - varlociraptor-delly/synthetic-20.INS.1-30.1.0.bcf - varlociraptor-delly/synthetic-20.INS.30-250.1.0.bcf - varlociraptor-lancet/synthetic-20.INS.1-30.1.0.bcf - varlociraptor-lancet/synthetic-20.INS.30-250.1.0.bcf - varlociraptor-manta/synthetic-20.INS.1-30.1.0.bcf - varlociraptor-manta/synthetic-20.INS.30-250.1.0.bcf - varlociraptor-strelka/synthetic-20.INS.1-30.1.0.bcf - varlociraptor-strelka/synthetic-20.INS.30-250.1.0.bcf - varlociraptor-neusomatic/simulated-bwa.DEL.1-30.1.0.bcf - varlociraptor-neusomatic/simulated-bwa.DEL.30-50.1.0.bcf - varlociraptor-neusomatic/simulated-bwa.DEL.50-100.1.0.bcf - varlociraptor-neusomatic/simulated-bwa.DEL.100-250.1.0.bcf - varlociraptor-neusomatic/synthetic-5.DEL.1-250.1.0.bcf - varlociraptor-delly/synthetic-5.DEL.1-250.1.0.bcf - varlociraptor-lancet/synthetic-5.DEL.1-250.1.0.bcf - varlociraptor-manta/synthetic-5.DEL.1-250.1.0.bcf - varlociraptor-strelka/synthetic-5.DEL.1-250.1.0.bcf - varlociraptor-neusomatic/synthetic-20.DEL.1-30.1.0.bcf - varlociraptor-neusomatic/synthetic-20.DEL.30-250.1.0.bcf - varlociraptor-delly/synthetic-20.DEL.1-30.1.0.bcf - varlociraptor-delly/synthetic-20.DEL.30-250.1.0.bcf - varlociraptor-lancet/synthetic-20.DEL.1-30.1.0.bcf - varlociraptor-lancet/synthetic-20.DEL.30-250.1.0.bcf - varlociraptor-manta/synthetic-20.DEL.1-30.1.0.bcf - varlociraptor-manta/synthetic-20.DEL.30-250.1.0.bcf - varlociraptor-strelka/synthetic-20.DEL.1-30.1.0.bcf - varlociraptor-strelka/synthetic-20.DEL.30-250.1.0.bcf - varlociraptor-neusomatic/simulated-bwa.INS.1-30.1e-05.bcf - varlociraptor-neusomatic/simulated-bwa.INS.1-30.0.0001.bcf - varlociraptor-neusomatic/simulated-bwa.INS.1-30.0.001.bcf - varlociraptor-neusomatic/simulated-bwa.INS.1-30.0.01.bcf - varlociraptor-neusomatic/simulated-bwa.INS.1-30.0.05.bcf - varlociraptor-neusomatic/simulated-bwa.INS.1-30.0.1.bcf - varlociraptor-neusomatic/simulated-bwa.INS.1-30.0.2.bcf - varlociraptor-neusomatic/simulated-bwa.INS.1-30.0.3.bcf - varlociraptor-neusomatic/simulated-bwa.INS.1-30.0.4.bcf - varlociraptor-neusomatic/simulated-bwa.INS.1-30.0.5.bcf - varlociraptor-neusomatic/simulated-bwa.INS.1-30.0.6.bcf - varlociraptor-neusomatic/simulated-bwa.INS.1-30.0.7.bcf - varlociraptor-neusomatic/simulated-bwa.INS.1-30.0.8.bcf - varlociraptor-neusomatic/simulated-bwa.INS.1-30.0.9.bcf - varlociraptor-neusomatic/simulated-bwa.INS.30-100.1e-05.bcf - varlociraptor-neusomatic/simulated-bwa.INS.30-100.0.0001.bcf - varlociraptor-neusomatic/simulated-bwa.INS.30-100.0.001.bcf - varlociraptor-neusomatic/simulated-bwa.INS.30-100.0.01.bcf - varlociraptor-neusomatic/simulated-bwa.INS.30-100.0.05.bcf - varlociraptor-neusomatic/simulated-bwa.INS.30-100.0.1.bcf - varlociraptor-neusomatic/simulated-bwa.INS.30-100.0.2.bcf - varlociraptor-neusomatic/simulated-bwa.INS.30-100.0.3.bcf - varlociraptor-neusomatic/simulated-bwa.INS.30-100.0.4.bcf - varlociraptor-neusomatic/simulated-bwa.INS.30-100.0.5.bcf - varlociraptor-neusomatic/simulated-bwa.INS.30-100.0.6.bcf - varlociraptor-neusomatic/simulated-bwa.INS.30-100.0.7.bcf - varlociraptor-neusomatic/simulated-bwa.INS.30-100.0.8.bcf - varlociraptor-neusomatic/simulated-bwa.INS.30-100.0.9.bcf - varlociraptor-neusomatic/simulated-bwa.DEL.1-30.1e-05.bcf - varlociraptor-neusomatic/simulated-bwa.DEL.1-30.0.0001.bcf - varlociraptor-neusomatic/simulated-bwa.DEL.1-30.0.001.bcf - varlociraptor-neusomatic/simulated-bwa.DEL.1-30.0.01.bcf - varlociraptor-neusomatic/simulated-bwa.DEL.1-30.0.05.bcf - varlociraptor-neusomatic/simulated-bwa.DEL.1-30.0.1.bcf - varlociraptor-neusomatic/simulated-bwa.DEL.1-30.0.2.bcf - varlociraptor-neusomatic/simulated-bwa.DEL.1-30.0.3.bcf - varlociraptor-neusomatic/simulated-bwa.DEL.1-30.0.4.bcf - varlociraptor-neusomatic/simulated-bwa.DEL.1-30.0.5.bcf - varlociraptor-neusomatic/simulated-bwa.DEL.1-30.0.6.bcf - varlociraptor-neusomatic/simulated-bwa.DEL.1-30.0.7.bcf - varlociraptor-neusomatic/simulated-bwa.DEL.1-30.0.8.bcf - varlociraptor-neusomatic/simulated-bwa.DEL.1-30.0.9.bcf - varlociraptor-neusomatic/simulated-bwa.DEL.30-50.1e-05.bcf - varlociraptor-neusomatic/simulated-bwa.DEL.30-50.0.0001.bcf - varlociraptor-neusomatic/simulated-bwa.DEL.30-50.0.001.bcf - varlociraptor-neusomatic/simulated-bwa.DEL.30-50.0.01.bcf - varlociraptor-neusomatic/simulated-bwa.DEL.30-50.0.05.bcf - varlociraptor-neusomatic/simulated-bwa.DEL.30-50.0.1.bcf - varlociraptor-neusomatic/simulated-bwa.DEL.30-50.0.2.bcf - varlociraptor-neusomatic/simulated-bwa.DEL.30-50.0.3.bcf - varlociraptor-neusomatic/simulated-bwa.DEL.30-50.0.4.bcf - varlociraptor-neusomatic/simulated-bwa.DEL.30-50.0.5.bcf - varlociraptor-neusomatic/simulated-bwa.DEL.30-50.0.6.bcf - varlociraptor-neusomatic/simulated-bwa.DEL.30-50.0.7.bcf - varlociraptor-neusomatic/simulated-bwa.DEL.30-50.0.8.bcf - varlociraptor-neusomatic/simulated-bwa.DEL.30-50.0.9.bcf - varlociraptor-neusomatic/simulated-bwa.DEL.50-100.1e-05.bcf - varlociraptor-neusomatic/simulated-bwa.DEL.50-100.0.0001.bcf - varlociraptor-neusomatic/simulated-bwa.DEL.50-100.0.001.bcf - varlociraptor-neusomatic/simulated-bwa.DEL.50-100.0.01.bcf - varlociraptor-neusomatic/simulated-bwa.DEL.50-100.0.05.bcf - varlociraptor-neusomatic/simulated-bwa.DEL.50-100.0.1.bcf - varlociraptor-neusomatic/simulated-bwa.DEL.50-100.0.2.bcf - varlociraptor-neusomatic/simulated-bwa.DEL.50-100.0.3.bcf - varlociraptor-neusomatic/simulated-bwa.DEL.50-100.0.4.bcf - varlociraptor-neusomatic/simulated-bwa.DEL.50-100.0.5.bcf - varlociraptor-neusomatic/simulated-bwa.DEL.50-100.0.6.bcf - varlociraptor-neusomatic/simulated-bwa.DEL.50-100.0.7.bcf - varlociraptor-neusomatic/simulated-bwa.DEL.50-100.0.8.bcf - varlociraptor-neusomatic/simulated-bwa.DEL.50-100.0.9.bcf - varlociraptor-neusomatic/simulated-bwa.DEL.100-250.1e-05.bcf - varlociraptor-neusomatic/simulated-bwa.DEL.100-250.0.0001.bcf - varlociraptor-neusomatic/simulated-bwa.DEL.100-250.0.001.bcf - varlociraptor-neusomatic/simulated-bwa.DEL.100-250.0.01.bcf - varlociraptor-neusomatic/simulated-bwa.DEL.100-250.0.05.bcf - varlociraptor-neusomatic/simulated-bwa.DEL.100-250.0.1.bcf - varlociraptor-neusomatic/simulated-bwa.DEL.100-250.0.2.bcf - varlociraptor-neusomatic/simulated-bwa.DEL.100-250.0.3.bcf - varlociraptor-neusomatic/simulated-bwa.DEL.100-250.0.4.bcf - varlociraptor-neusomatic/simulated-bwa.DEL.100-250.0.5.bcf - varlociraptor-neusomatic/simulated-bwa.DEL.100-250.0.6.bcf - varlociraptor-neusomatic/simulated-bwa.DEL.100-250.0.7.bcf - varlociraptor-neusomatic/simulated-bwa.DEL.100-250.0.8.bcf - varlociraptor-neusomatic/simulated-bwa.DEL.100-250.0.9.bcf - varlociraptor-neusomatic/simulated-bwa.INS.1-250.1.0.bcf - varlociraptor-neusomatic/simulated-bwa.DEL.1-250.1.0.bcf |  | - varlociraptor =1.1.1 - bcftools =1.9 | |  |  | | --- | --- | | ``` 1 ``` | ``` varlociraptor filter-calls control-fdr {input} --events SOMATIC_TUMOR --var {wildcards.type} --minlen {wildcards.minlen} --maxlen {wildcards.maxlen} --fdr {wildcards.fdr} > {output} ``` | |
| varlociraptor\_control\_fdr | 368 | - varlociraptor-delly/simulated-bwa.INS.1-30.1.0.bcf - varlociraptor-delly/simulated-bwa.INS.30-100.1.0.bcf - varlociraptor-lancet/simulated-bwa.INS.1-30.1.0.bcf - varlociraptor-lancet/simulated-bwa.INS.30-100.1.0.bcf - varlociraptor-manta/simulated-bwa.INS.1-30.1.0.bcf - varlociraptor-manta/simulated-bwa.INS.30-100.1.0.bcf - varlociraptor-strelka/simulated-bwa.INS.1-30.1.0.bcf - varlociraptor-strelka/simulated-bwa.INS.30-100.1.0.bcf - varlociraptor-delly/simulated-bwa.DEL.1-30.1.0.bcf - varlociraptor-delly/simulated-bwa.DEL.30-50.1.0.bcf - varlociraptor-delly/simulated-bwa.DEL.50-100.1.0.bcf - varlociraptor-delly/simulated-bwa.DEL.100-250.1.0.bcf - varlociraptor-lancet/simulated-bwa.DEL.1-30.1.0.bcf - varlociraptor-lancet/simulated-bwa.DEL.30-50.1.0.bcf - varlociraptor-lancet/simulated-bwa.DEL.50-100.1.0.bcf - varlociraptor-lancet/simulated-bwa.DEL.100-250.1.0.bcf - varlociraptor-manta/simulated-bwa.DEL.1-30.1.0.bcf - varlociraptor-manta/simulated-bwa.DEL.30-50.1.0.bcf - varlociraptor-manta/simulated-bwa.DEL.50-100.1.0.bcf - varlociraptor-manta/simulated-bwa.DEL.100-250.1.0.bcf - varlociraptor-strelka/simulated-bwa.DEL.1-30.1.0.bcf - varlociraptor-strelka/simulated-bwa.DEL.30-50.1.0.bcf - varlociraptor-strelka/simulated-bwa.DEL.50-100.1.0.bcf - varlociraptor-strelka/simulated-bwa.DEL.100-250.1.0.bcf - varlociraptor-delly/simulated-bwa.INS.1-30.1e-05.bcf - varlociraptor-delly/simulated-bwa.INS.1-30.0.0001.bcf - varlociraptor-delly/simulated-bwa.INS.1-30.0.001.bcf - varlociraptor-delly/simulated-bwa.INS.1-30.0.01.bcf - varlociraptor-delly/simulated-bwa.INS.1-30.0.05.bcf - varlociraptor-delly/simulated-bwa.INS.1-30.0.1.bcf - varlociraptor-delly/simulated-bwa.INS.1-30.0.2.bcf - varlociraptor-delly/simulated-bwa.INS.1-30.0.3.bcf - varlociraptor-delly/simulated-bwa.INS.1-30.0.4.bcf - varlociraptor-delly/simulated-bwa.INS.1-30.0.5.bcf - varlociraptor-delly/simulated-bwa.INS.1-30.0.6.bcf - varlociraptor-delly/simulated-bwa.INS.1-30.0.7.bcf - varlociraptor-delly/simulated-bwa.INS.1-30.0.8.bcf - varlociraptor-delly/simulated-bwa.INS.1-30.0.9.bcf - varlociraptor-delly/simulated-bwa.INS.30-100.1e-05.bcf - varlociraptor-delly/simulated-bwa.INS.30-100.0.0001.bcf - varlociraptor-delly/simulated-bwa.INS.30-100.0.001.bcf - varlociraptor-delly/simulated-bwa.INS.30-100.0.01.bcf - varlociraptor-delly/simulated-bwa.INS.30-100.0.05.bcf - varlociraptor-delly/simulated-bwa.INS.30-100.0.1.bcf - varlociraptor-delly/simulated-bwa.INS.30-100.0.2.bcf - varlociraptor-delly/simulated-bwa.INS.30-100.0.3.bcf - varlociraptor-delly/simulated-bwa.INS.30-100.0.4.bcf - varlociraptor-delly/simulated-bwa.INS.30-100.0.5.bcf - varlociraptor-delly/simulated-bwa.INS.30-100.0.6.bcf - varlociraptor-delly/simulated-bwa.INS.30-100.0.7.bcf - varlociraptor-delly/simulated-bwa.INS.30-100.0.8.bcf - varlociraptor-delly/simulated-bwa.INS.30-100.0.9.bcf - varlociraptor-lancet/simulated-bwa.INS.1-30.1e-05.bcf - varlociraptor-lancet/simulated-bwa.INS.1-30.0.0001.bcf - varlociraptor-lancet/simulated-bwa.INS.1-30.0.001.bcf - varlociraptor-lancet/simulated-bwa.INS.1-30.0.01.bcf - varlociraptor-lancet/simulated-bwa.INS.1-30.0.05.bcf - varlociraptor-lancet/simulated-bwa.INS.1-30.0.1.bcf - varlociraptor-lancet/simulated-bwa.INS.1-30.0.2.bcf - varlociraptor-lancet/simulated-bwa.INS.1-30.0.3.bcf - varlociraptor-lancet/simulated-bwa.INS.1-30.0.4.bcf - varlociraptor-lancet/simulated-bwa.INS.1-30.0.5.bcf - varlociraptor-lancet/simulated-bwa.INS.1-30.0.6.bcf - varlociraptor-lancet/simulated-bwa.INS.1-30.0.7.bcf - varlociraptor-lancet/simulated-bwa.INS.1-30.0.8.bcf - varlociraptor-lancet/simulated-bwa.INS.1-30.0.9.bcf - varlociraptor-lancet/simulated-bwa.INS.30-100.1e-05.bcf - varlociraptor-lancet/simulated-bwa.INS.30-100.0.0001.bcf - varlociraptor-lancet/simulated-bwa.INS.30-100.0.001.bcf - varlociraptor-lancet/simulated-bwa.INS.30-100.0.01.bcf - varlociraptor-lancet/simulated-bwa.INS.30-100.0.05.bcf - varlociraptor-lancet/simulated-bwa.INS.30-100.0.1.bcf - varlociraptor-lancet/simulated-bwa.INS.30-100.0.2.bcf - varlociraptor-lancet/simulated-bwa.INS.30-100.0.3.bcf - varlociraptor-lancet/simulated-bwa.INS.30-100.0.4.bcf - varlociraptor-lancet/simulated-bwa.INS.30-100.0.5.bcf - varlociraptor-lancet/simulated-bwa.INS.30-100.0.6.bcf - varlociraptor-lancet/simulated-bwa.INS.30-100.0.7.bcf - varlociraptor-lancet/simulated-bwa.INS.30-100.0.8.bcf - varlociraptor-lancet/simulated-bwa.INS.30-100.0.9.bcf - varlociraptor-manta/simulated-bwa.INS.1-30.1e-05.bcf - varlociraptor-manta/simulated-bwa.INS.1-30.0.0001.bcf - varlociraptor-manta/simulated-bwa.INS.1-30.0.001.bcf - varlociraptor-manta/simulated-bwa.INS.1-30.0.01.bcf - varlociraptor-manta/simulated-bwa.INS.1-30.0.05.bcf - varlociraptor-manta/simulated-bwa.INS.1-30.0.1.bcf - varlociraptor-manta/simulated-bwa.INS.1-30.0.2.bcf - varlociraptor-manta/simulated-bwa.INS.1-30.0.3.bcf - varlociraptor-manta/simulated-bwa.INS.1-30.0.4.bcf - varlociraptor-manta/simulated-bwa.INS.1-30.0.5.bcf - varlociraptor-manta/simulated-bwa.INS.1-30.0.6.bcf - varlociraptor-manta/simulated-bwa.INS.1-30.0.7.bcf - varlociraptor-manta/simulated-bwa.INS.1-30.0.8.bcf - varlociraptor-manta/simulated-bwa.INS.1-30.0.9.bcf - varlociraptor-manta/simulated-bwa.INS.30-100.1e-05.bcf - varlociraptor-manta/simulated-bwa.INS.30-100.0.0001.bcf - varlociraptor-manta/simulated-bwa.INS.30-100.0.001.bcf - varlociraptor-manta/simulated-bwa.INS.30-100.0.01.bcf - varlociraptor-manta/simulated-bwa.INS.30-100.0.05.bcf - varlociraptor-manta/simulated-bwa.INS.30-100.0.1.bcf - varlociraptor-manta/simulated-bwa.INS.30-100.0.2.bcf - varlociraptor-manta/simulated-bwa.INS.30-100.0.3.bcf - varlociraptor-manta/simulated-bwa.INS.30-100.0.4.bcf - varlociraptor-manta/simulated-bwa.INS.30-100.0.5.bcf - varlociraptor-manta/simulated-bwa.INS.30-100.0.6.bcf - varlociraptor-manta/simulated-bwa.INS.30-100.0.7.bcf - varlociraptor-manta/simulated-bwa.INS.30-100.0.8.bcf - varlociraptor-manta/simulated-bwa.INS.30-100.0.9.bcf - varlociraptor-strelka/simulated-bwa.INS.1-30.1e-05.bcf - varlociraptor-strelka/simulated-bwa.INS.1-30.0.0001.bcf - varlociraptor-strelka/simulated-bwa.INS.1-30.0.001.bcf - varlociraptor-strelka/simulated-bwa.INS.1-30.0.01.bcf - varlociraptor-strelka/simulated-bwa.INS.1-30.0.05.bcf - varlociraptor-strelka/simulated-bwa.INS.1-30.0.1.bcf - varlociraptor-strelka/simulated-bwa.INS.1-30.0.2.bcf - varlociraptor-strelka/simulated-bwa.INS.1-30.0.3.bcf - varlociraptor-strelka/simulated-bwa.INS.1-30.0.4.bcf - varlociraptor-strelka/simulated-bwa.INS.1-30.0.5.bcf - varlociraptor-strelka/simulated-bwa.INS.1-30.0.6.bcf - varlociraptor-strelka/simulated-bwa.INS.1-30.0.7.bcf - varlociraptor-strelka/simulated-bwa.INS.1-30.0.8.bcf - varlociraptor-strelka/simulated-bwa.INS.1-30.0.9.bcf - varlociraptor-strelka/simulated-bwa.INS.30-100.1e-05.bcf - varlociraptor-strelka/simulated-bwa.INS.30-100.0.0001.bcf - varlociraptor-strelka/simulated-bwa.INS.30-100.0.001.bcf - varlociraptor-strelka/simulated-bwa.INS.30-100.0.01.bcf - varlociraptor-strelka/simulated-bwa.INS.30-100.0.05.bcf - varlociraptor-strelka/simulated-bwa.INS.30-100.0.1.bcf - varlociraptor-strelka/simulated-bwa.INS.30-100.0.2.bcf - varlociraptor-strelka/simulated-bwa.INS.30-100.0.3.bcf - varlociraptor-strelka/simulated-bwa.INS.30-100.0.4.bcf - varlociraptor-strelka/simulated-bwa.INS.30-100.0.5.bcf - varlociraptor-strelka/simulated-bwa.INS.30-100.0.6.bcf - varlociraptor-strelka/simulated-bwa.INS.30-100.0.7.bcf - varlociraptor-strelka/simulated-bwa.INS.30-100.0.8.bcf - varlociraptor-strelka/simulated-bwa.INS.30-100.0.9.bcf - varlociraptor-delly/simulated-bwa.DEL.1-30.1e-05.bcf - varlociraptor-delly/simulated-bwa.DEL.1-30.0.0001.bcf - varlociraptor-delly/simulated-bwa.DEL.1-30.0.001.bcf - varlociraptor-delly/simulated-bwa.DEL.1-30.0.01.bcf - varlociraptor-delly/simulated-bwa.DEL.1-30.0.05.bcf - varlociraptor-delly/simulated-bwa.DEL.1-30.0.1.bcf - varlociraptor-delly/simulated-bwa.DEL.1-30.0.2.bcf - varlociraptor-delly/simulated-bwa.DEL.1-30.0.3.bcf - varlociraptor-delly/simulated-bwa.DEL.1-30.0.4.bcf - varlociraptor-delly/simulated-bwa.DEL.1-30.0.5.bcf - varlociraptor-delly/simulated-bwa.DEL.1-30.0.6.bcf - varlociraptor-delly/simulated-bwa.DEL.1-30.0.7.bcf - varlociraptor-delly/simulated-bwa.DEL.1-30.0.8.bcf - varlociraptor-delly/simulated-bwa.DEL.1-30.0.9.bcf - varlociraptor-delly/simulated-bwa.DEL.30-50.1e-05.bcf - varlociraptor-delly/simulated-bwa.DEL.30-50.0.0001.bcf - varlociraptor-delly/simulated-bwa.DEL.30-50.0.001.bcf - varlociraptor-delly/simulated-bwa.DEL.30-50.0.01.bcf - varlociraptor-delly/simulated-bwa.DEL.30-50.0.05.bcf - varlociraptor-delly/simulated-bwa.DEL.30-50.0.1.bcf - varlociraptor-delly/simulated-bwa.DEL.30-50.0.2.bcf - varlociraptor-delly/simulated-bwa.DEL.30-50.0.3.bcf - varlociraptor-delly/simulated-bwa.DEL.30-50.0.4.bcf - varlociraptor-delly/simulated-bwa.DEL.30-50.0.5.bcf - varlociraptor-delly/simulated-bwa.DEL.30-50.0.6.bcf - varlociraptor-delly/simulated-bwa.DEL.30-50.0.7.bcf - varlociraptor-delly/simulated-bwa.DEL.30-50.0.8.bcf - varlociraptor-delly/simulated-bwa.DEL.30-50.0.9.bcf - varlociraptor-delly/simulated-bwa.DEL.50-100.1e-05.bcf - varlociraptor-delly/simulated-bwa.DEL.50-100.0.0001.bcf - varlociraptor-delly/simulated-bwa.DEL.50-100.0.001.bcf - varlociraptor-delly/simulated-bwa.DEL.50-100.0.01.bcf - varlociraptor-delly/simulated-bwa.DEL.50-100.0.05.bcf - varlociraptor-delly/simulated-bwa.DEL.50-100.0.1.bcf - varlociraptor-delly/simulated-bwa.DEL.50-100.0.2.bcf - varlociraptor-delly/simulated-bwa.DEL.50-100.0.3.bcf - varlociraptor-delly/simulated-bwa.DEL.50-100.0.4.bcf - varlociraptor-delly/simulated-bwa.DEL.50-100.0.5.bcf - varlociraptor-delly/simulated-bwa.DEL.50-100.0.6.bcf - varlociraptor-delly/simulated-bwa.DEL.50-100.0.7.bcf - varlociraptor-delly/simulated-bwa.DEL.50-100.0.8.bcf - varlociraptor-delly/simulated-bwa.DEL.50-100.0.9.bcf - varlociraptor-delly/simulated-bwa.DEL.100-250.1e-05.bcf - varlociraptor-delly/simulated-bwa.DEL.100-250.0.0001.bcf - varlociraptor-delly/simulated-bwa.DEL.100-250.0.001.bcf - varlociraptor-delly/simulated-bwa.DEL.100-250.0.01.bcf - varlociraptor-delly/simulated-bwa.DEL.100-250.0.05.bcf - varlociraptor-delly/simulated-bwa.DEL.100-250.0.1.bcf - varlociraptor-delly/simulated-bwa.DEL.100-250.0.2.bcf - varlociraptor-delly/simulated-bwa.DEL.100-250.0.3.bcf - varlociraptor-delly/simulated-bwa.DEL.100-250.0.4.bcf - varlociraptor-delly/simulated-bwa.DEL.100-250.0.5.bcf - varlociraptor-delly/simulated-bwa.DEL.100-250.0.6.bcf - varlociraptor-delly/simulated-bwa.DEL.100-250.0.7.bcf - varlociraptor-delly/simulated-bwa.DEL.100-250.0.8.bcf - varlociraptor-delly/simulated-bwa.DEL.100-250.0.9.bcf - varlociraptor-lancet/simulated-bwa.DEL.1-30.1e-05.bcf - varlociraptor-lancet/simulated-bwa.DEL.1-30.0.0001.bcf - varlociraptor-lancet/simulated-bwa.DEL.1-30.0.001.bcf - varlociraptor-lancet/simulated-bwa.DEL.1-30.0.01.bcf - varlociraptor-lancet/simulated-bwa.DEL.1-30.0.05.bcf - varlociraptor-lancet/simulated-bwa.DEL.1-30.0.1.bcf - varlociraptor-lancet/simulated-bwa.DEL.1-30.0.2.bcf - varlociraptor-lancet/simulated-bwa.DEL.1-30.0.3.bcf - varlociraptor-lancet/simulated-bwa.DEL.1-30.0.4.bcf - varlociraptor-lancet/simulated-bwa.DEL.1-30.0.5.bcf - varlociraptor-lancet/simulated-bwa.DEL.1-30.0.6.bcf - varlociraptor-lancet/simulated-bwa.DEL.1-30.0.7.bcf - varlociraptor-lancet/simulated-bwa.DEL.1-30.0.8.bcf - varlociraptor-lancet/simulated-bwa.DEL.1-30.0.9.bcf - varlociraptor-lancet/simulated-bwa.DEL.30-50.1e-05.bcf - varlociraptor-lancet/simulated-bwa.DEL.30-50.0.0001.bcf - varlociraptor-lancet/simulated-bwa.DEL.30-50.0.001.bcf - varlociraptor-lancet/simulated-bwa.DEL.30-50.0.01.bcf - varlociraptor-lancet/simulated-bwa.DEL.30-50.0.05.bcf - varlociraptor-lancet/simulated-bwa.DEL.30-50.0.1.bcf - varlociraptor-lancet/simulated-bwa.DEL.30-50.0.2.bcf - varlociraptor-lancet/simulated-bwa.DEL.30-50.0.3.bcf - varlociraptor-lancet/simulated-bwa.DEL.30-50.0.4.bcf - varlociraptor-lancet/simulated-bwa.DEL.30-50.0.5.bcf - varlociraptor-lancet/simulated-bwa.DEL.30-50.0.6.bcf - varlociraptor-lancet/simulated-bwa.DEL.30-50.0.7.bcf - varlociraptor-lancet/simulated-bwa.DEL.30-50.0.8.bcf - varlociraptor-lancet/simulated-bwa.DEL.30-50.0.9.bcf - varlociraptor-lancet/simulated-bwa.DEL.50-100.1e-05.bcf - varlociraptor-lancet/simulated-bwa.DEL.50-100.0.0001.bcf - varlociraptor-lancet/simulated-bwa.DEL.50-100.0.001.bcf - varlociraptor-lancet/simulated-bwa.DEL.50-100.0.01.bcf - varlociraptor-lancet/simulated-bwa.DEL.50-100.0.05.bcf - varlociraptor-lancet/simulated-bwa.DEL.50-100.0.1.bcf - varlociraptor-lancet/simulated-bwa.DEL.50-100.0.2.bcf - varlociraptor-lancet/simulated-bwa.DEL.50-100.0.3.bcf - varlociraptor-lancet/simulated-bwa.DEL.50-100.0.4.bcf - varlociraptor-lancet/simulated-bwa.DEL.50-100.0.5.bcf - varlociraptor-lancet/simulated-bwa.DEL.50-100.0.6.bcf - varlociraptor-lancet/simulated-bwa.DEL.50-100.0.7.bcf - varlociraptor-lancet/simulated-bwa.DEL.50-100.0.8.bcf - varlociraptor-lancet/simulated-bwa.DEL.50-100.0.9.bcf - varlociraptor-lancet/simulated-bwa.DEL.100-250.1e-05.bcf - varlociraptor-lancet/simulated-bwa.DEL.100-250.0.0001.bcf - varlociraptor-lancet/simulated-bwa.DEL.100-250.0.001.bcf - varlociraptor-lancet/simulated-bwa.DEL.100-250.0.01.bcf - varlociraptor-lancet/simulated-bwa.DEL.100-250.0.05.bcf - varlociraptor-lancet/simulated-bwa.DEL.100-250.0.1.bcf - varlociraptor-lancet/simulated-bwa.DEL.100-250.0.2.bcf - varlociraptor-lancet/simulated-bwa.DEL.100-250.0.3.bcf - varlociraptor-lancet/simulated-bwa.DEL.100-250.0.4.bcf - varlociraptor-lancet/simulated-bwa.DEL.100-250.0.5.bcf - varlociraptor-lancet/simulated-bwa.DEL.100-250.0.6.bcf - varlociraptor-lancet/simulated-bwa.DEL.100-250.0.7.bcf - varlociraptor-lancet/simulated-bwa.DEL.100-250.0.8.bcf - varlociraptor-lancet/simulated-bwa.DEL.100-250.0.9.bcf - varlociraptor-manta/simulated-bwa.DEL.1-30.1e-05.bcf - varlociraptor-manta/simulated-bwa.DEL.1-30.0.0001.bcf - varlociraptor-manta/simulated-bwa.DEL.1-30.0.001.bcf - varlociraptor-manta/simulated-bwa.DEL.1-30.0.01.bcf - varlociraptor-manta/simulated-bwa.DEL.1-30.0.05.bcf - varlociraptor-manta/simulated-bwa.DEL.1-30.0.1.bcf - varlociraptor-manta/simulated-bwa.DEL.1-30.0.2.bcf - varlociraptor-manta/simulated-bwa.DEL.1-30.0.3.bcf - varlociraptor-manta/simulated-bwa.DEL.1-30.0.4.bcf - varlociraptor-manta/simulated-bwa.DEL.1-30.0.5.bcf - varlociraptor-manta/simulated-bwa.DEL.1-30.0.6.bcf - varlociraptor-manta/simulated-bwa.DEL.1-30.0.7.bcf - varlociraptor-manta/simulated-bwa.DEL.1-30.0.8.bcf - varlociraptor-manta/simulated-bwa.DEL.1-30.0.9.bcf - varlociraptor-manta/simulated-bwa.DEL.30-50.1e-05.bcf - varlociraptor-manta/simulated-bwa.DEL.30-50.0.0001.bcf - varlociraptor-manta/simulated-bwa.DEL.30-50.0.001.bcf - varlociraptor-manta/simulated-bwa.DEL.30-50.0.01.bcf - varlociraptor-manta/simulated-bwa.DEL.30-50.0.05.bcf - varlociraptor-manta/simulated-bwa.DEL.30-50.0.1.bcf - varlociraptor-manta/simulated-bwa.DEL.30-50.0.2.bcf - varlociraptor-manta/simulated-bwa.DEL.30-50.0.3.bcf - varlociraptor-manta/simulated-bwa.DEL.30-50.0.4.bcf - varlociraptor-manta/simulated-bwa.DEL.30-50.0.5.bcf - varlociraptor-manta/simulated-bwa.DEL.30-50.0.6.bcf - varlociraptor-manta/simulated-bwa.DEL.30-50.0.7.bcf - varlociraptor-manta/simulated-bwa.DEL.30-50.0.8.bcf - varlociraptor-manta/simulated-bwa.DEL.30-50.0.9.bcf - varlociraptor-manta/simulated-bwa.DEL.50-100.1e-05.bcf - varlociraptor-manta/simulated-bwa.DEL.50-100.0.0001.bcf - varlociraptor-manta/simulated-bwa.DEL.50-100.0.001.bcf - varlociraptor-manta/simulated-bwa.DEL.50-100.0.01.bcf - varlociraptor-manta/simulated-bwa.DEL.50-100.0.05.bcf - varlociraptor-manta/simulated-bwa.DEL.50-100.0.1.bcf - varlociraptor-manta/simulated-bwa.DEL.50-100.0.2.bcf - varlociraptor-manta/simulated-bwa.DEL.50-100.0.3.bcf - varlociraptor-manta/simulated-bwa.DEL.50-100.0.4.bcf - varlociraptor-manta/simulated-bwa.DEL.50-100.0.5.bcf - varlociraptor-manta/simulated-bwa.DEL.50-100.0.6.bcf - varlociraptor-manta/simulated-bwa.DEL.50-100.0.7.bcf - varlociraptor-manta/simulated-bwa.DEL.50-100.0.8.bcf - varlociraptor-manta/simulated-bwa.DEL.50-100.0.9.bcf - varlociraptor-manta/simulated-bwa.DEL.100-250.1e-05.bcf - varlociraptor-manta/simulated-bwa.DEL.100-250.0.0001.bcf - varlociraptor-manta/simulated-bwa.DEL.100-250.0.001.bcf - varlociraptor-manta/simulated-bwa.DEL.100-250.0.01.bcf - varlociraptor-manta/simulated-bwa.DEL.100-250.0.05.bcf - varlociraptor-manta/simulated-bwa.DEL.100-250.0.1.bcf - varlociraptor-manta/simulated-bwa.DEL.100-250.0.2.bcf - varlociraptor-manta/simulated-bwa.DEL.100-250.0.3.bcf - varlociraptor-manta/simulated-bwa.DEL.100-250.0.4.bcf - varlociraptor-manta/simulated-bwa.DEL.100-250.0.5.bcf - varlociraptor-manta/simulated-bwa.DEL.100-250.0.6.bcf - varlociraptor-manta/simulated-bwa.DEL.100-250.0.7.bcf - varlociraptor-manta/simulated-bwa.DEL.100-250.0.8.bcf - varlociraptor-manta/simulated-bwa.DEL.100-250.0.9.bcf - varlociraptor-strelka/simulated-bwa.DEL.1-30.1e-05.bcf - varlociraptor-strelka/simulated-bwa.DEL.1-30.0.0001.bcf - varlociraptor-strelka/simulated-bwa.DEL.1-30.0.001.bcf - varlociraptor-strelka/simulated-bwa.DEL.1-30.0.01.bcf - varlociraptor-strelka/simulated-bwa.DEL.1-30.0.05.bcf - varlociraptor-strelka/simulated-bwa.DEL.1-30.0.1.bcf - varlociraptor-strelka/simulated-bwa.DEL.1-30.0.2.bcf - varlociraptor-strelka/simulated-bwa.DEL.1-30.0.3.bcf - varlociraptor-strelka/simulated-bwa.DEL.1-30.0.4.bcf - varlociraptor-strelka/simulated-bwa.DEL.1-30.0.5.bcf - varlociraptor-strelka/simulated-bwa.DEL.1-30.0.6.bcf - varlociraptor-strelka/simulated-bwa.DEL.1-30.0.7.bcf - varlociraptor-strelka/simulated-bwa.DEL.1-30.0.8.bcf - varlociraptor-strelka/simulated-bwa.DEL.1-30.0.9.bcf - varlociraptor-strelka/simulated-bwa.DEL.30-50.1e-05.bcf - varlociraptor-strelka/simulated-bwa.DEL.30-50.0.0001.bcf - varlociraptor-strelka/simulated-bwa.DEL.30-50.0.001.bcf - varlociraptor-strelka/simulated-bwa.DEL.30-50.0.01.bcf - varlociraptor-strelka/simulated-bwa.DEL.30-50.0.05.bcf - varlociraptor-strelka/simulated-bwa.DEL.30-50.0.1.bcf - varlociraptor-strelka/simulated-bwa.DEL.30-50.0.2.bcf - varlociraptor-strelka/simulated-bwa.DEL.30-50.0.3.bcf - varlociraptor-strelka/simulated-bwa.DEL.30-50.0.4.bcf - varlociraptor-strelka/simulated-bwa.DEL.30-50.0.5.bcf - varlociraptor-strelka/simulated-bwa.DEL.30-50.0.6.bcf - varlociraptor-strelka/simulated-bwa.DEL.30-50.0.7.bcf - varlociraptor-strelka/simulated-bwa.DEL.30-50.0.8.bcf - varlociraptor-strelka/simulated-bwa.DEL.30-50.0.9.bcf - varlociraptor-strelka/simulated-bwa.DEL.50-100.1e-05.bcf - varlociraptor-strelka/simulated-bwa.DEL.50-100.0.0001.bcf - varlociraptor-strelka/simulated-bwa.DEL.50-100.0.001.bcf - varlociraptor-strelka/simulated-bwa.DEL.50-100.0.01.bcf - varlociraptor-strelka/simulated-bwa.DEL.50-100.0.05.bcf - varlociraptor-strelka/simulated-bwa.DEL.50-100.0.1.bcf - varlociraptor-strelka/simulated-bwa.DEL.50-100.0.2.bcf - varlociraptor-strelka/simulated-bwa.DEL.50-100.0.3.bcf - varlociraptor-strelka/simulated-bwa.DEL.50-100.0.4.bcf - varlociraptor-strelka/simulated-bwa.DEL.50-100.0.5.bcf - varlociraptor-strelka/simulated-bwa.DEL.50-100.0.6.bcf - varlociraptor-strelka/simulated-bwa.DEL.50-100.0.7.bcf - varlociraptor-strelka/simulated-bwa.DEL.50-100.0.8.bcf - varlociraptor-strelka/simulated-bwa.DEL.50-100.0.9.bcf - varlociraptor-strelka/simulated-bwa.DEL.100-250.1e-05.bcf - varlociraptor-strelka/simulated-bwa.DEL.100-250.0.0001.bcf - varlociraptor-strelka/simulated-bwa.DEL.100-250.0.001.bcf - varlociraptor-strelka/simulated-bwa.DEL.100-250.0.01.bcf - varlociraptor-strelka/simulated-bwa.DEL.100-250.0.05.bcf - varlociraptor-strelka/simulated-bwa.DEL.100-250.0.1.bcf - varlociraptor-strelka/simulated-bwa.DEL.100-250.0.2.bcf - varlociraptor-strelka/simulated-bwa.DEL.100-250.0.3.bcf - varlociraptor-strelka/simulated-bwa.DEL.100-250.0.4.bcf - varlociraptor-strelka/simulated-bwa.DEL.100-250.0.5.bcf - varlociraptor-strelka/simulated-bwa.DEL.100-250.0.6.bcf - varlociraptor-strelka/simulated-bwa.DEL.100-250.0.7.bcf - varlociraptor-strelka/simulated-bwa.DEL.100-250.0.8.bcf - varlociraptor-strelka/simulated-bwa.DEL.100-250.0.9.bcf - varlociraptor-delly/simulated-bwa.INS.1-250.1.0.bcf - varlociraptor-lancet/simulated-bwa.INS.1-250.1.0.bcf - varlociraptor-manta/simulated-bwa.INS.1-250.1.0.bcf - varlociraptor-strelka/simulated-bwa.INS.1-250.1.0.bcf - varlociraptor-delly/simulated-bwa.DEL.1-250.1.0.bcf - varlociraptor-lancet/simulated-bwa.DEL.1-250.1.0.bcf - varlociraptor-manta/simulated-bwa.DEL.1-250.1.0.bcf - varlociraptor-strelka/simulated-bwa.DEL.1-250.1.0.bcf |  |  | |  |  | | --- | --- | | ``` 1 ``` | ``` varlociraptor filter-calls control-fdr {input} --events SOMATIC_TUMOR --var {wildcards.type} --minlen {wildcards.minlen} --maxlen {wildcards.maxlen} --fdr {wildcards.fdr} > {output} ``` | |
| fix\_neusomatic | 7 | - default-neusomatic/simulated-bwa.all.bcf - default-neusomatic/synthetic-5.all.bcf - default-neusomatic/synthetic-20.all.bcf - default-neusomatic/COLO\_829-GSC.all.bcf - default-neusomatic/COLO\_829-Ill.all.bcf - default-neusomatic/COLO\_829-TGen.all.bcf - default-neusomatic/COLO\_829-EBI.all.bcf |  | - bcftools =1.6 - samtools =1.6 | |  |  | | --- | --- | | ``` 1 ``` | ``` bcftools annotate -Ob -o {output} -h {input.header} {input.vcf} ``` | |
| index\_bcf | 80 | - default-neusomatic/simulated-bwa.all.bcf.csi - adhoc-delly/simulated-bwa.all.bcf.csi - varlociraptor-neusomatic/synthetic-5.INS.1-250.1.0.bcf.csi - varlociraptor-delly/synthetic-5.INS.1-250.1.0.bcf.csi - varlociraptor-lancet/synthetic-5.INS.1-250.1.0.bcf.csi - varlociraptor-manta/synthetic-5.INS.1-250.1.0.bcf.csi - varlociraptor-strelka/synthetic-5.INS.1-250.1.0.bcf.csi - default-neusomatic/synthetic-5.all.bcf.csi - default-lancet/synthetic-5.all.bcf.csi - default-manta/synthetic-5.all.bcf.csi - default-strelka/synthetic-5.all.bcf.csi - adhoc-neusomatic/synthetic-5.all.bcf.csi - adhoc-delly/synthetic-5.all.bcf.csi - adhoc-lancet/synthetic-5.all.bcf.csi - adhoc-manta/synthetic-5.all.bcf.csi - adhoc-strelka/synthetic-5.all.bcf.csi - adhoc-bpi/synthetic-5.all.bcf.csi - varlociraptor-neusomatic/synthetic-20.INS.1-30.1.0.bcf.csi - varlociraptor-neusomatic/synthetic-20.INS.30-250.1.0.bcf.csi - varlociraptor-delly/synthetic-20.INS.1-30.1.0.bcf.csi - varlociraptor-delly/synthetic-20.INS.30-250.1.0.bcf.csi - varlociraptor-lancet/synthetic-20.INS.1-30.1.0.bcf.csi - varlociraptor-lancet/synthetic-20.INS.30-250.1.0.bcf.csi - varlociraptor-manta/synthetic-20.INS.1-30.1.0.bcf.csi - varlociraptor-manta/synthetic-20.INS.30-250.1.0.bcf.csi - varlociraptor-strelka/synthetic-20.INS.1-30.1.0.bcf.csi - varlociraptor-strelka/synthetic-20.INS.30-250.1.0.bcf.csi - default-neusomatic/synthetic-20.all.bcf.csi - default-lancet/synthetic-20.all.bcf.csi - default-manta/synthetic-20.all.bcf.csi - default-strelka/synthetic-20.all.bcf.csi - adhoc-neusomatic/synthetic-20.all.bcf.csi - adhoc-delly/synthetic-20.all.bcf.csi - adhoc-lancet/synthetic-20.all.bcf.csi - adhoc-manta/synthetic-20.all.bcf.csi - adhoc-strelka/synthetic-20.all.bcf.csi - adhoc-bpi/synthetic-20.all.bcf.csi - varlociraptor-neusomatic/synthetic-5.DEL.1-250.1.0.bcf.csi - varlociraptor-delly/synthetic-5.DEL.1-250.1.0.bcf.csi - varlociraptor-lancet/synthetic-5.DEL.1-250.1.0.bcf.csi - varlociraptor-manta/synthetic-5.DEL.1-250.1.0.bcf.csi - varlociraptor-strelka/synthetic-5.DEL.1-250.1.0.bcf.csi - varlociraptor-neusomatic/synthetic-20.DEL.1-30.1.0.bcf.csi - varlociraptor-neusomatic/synthetic-20.DEL.30-250.1.0.bcf.csi - varlociraptor-delly/synthetic-20.DEL.1-30.1.0.bcf.csi - varlociraptor-delly/synthetic-20.DEL.30-250.1.0.bcf.csi - varlociraptor-lancet/synthetic-20.DEL.1-30.1.0.bcf.csi - varlociraptor-lancet/synthetic-20.DEL.30-250.1.0.bcf.csi - varlociraptor-manta/synthetic-20.DEL.1-30.1.0.bcf.csi - varlociraptor-manta/synthetic-20.DEL.30-250.1.0.bcf.csi - varlociraptor-strelka/synthetic-20.DEL.1-30.1.0.bcf.csi - varlociraptor-strelka/synthetic-20.DEL.30-250.1.0.bcf.csi - delly/synthetic-5.all.bcf.csi - delly/synthetic-20.all.bcf.csi - default-neusomatic/COLO\_829-GSC.all.bcf.csi - default-neusomatic/COLO\_829-Ill.all.bcf.csi - default-neusomatic/COLO\_829-TGen.all.bcf.csi - default-neusomatic/COLO\_829-EBI.all.bcf.csi - delly/COLO\_829-GSC.all.bcf.csi - delly/COLO\_829-Ill.all.bcf.csi - delly/COLO\_829-TGen.all.bcf.csi - delly/COLO\_829-EBI.all.bcf.csi - default-lancet/COLO\_829-GSC.all.bcf.csi - default-lancet/COLO\_829-Ill.all.bcf.csi - default-lancet/COLO\_829-TGen.all.bcf.csi - default-lancet/COLO\_829-EBI.all.bcf.csi - manta/COLO\_829-GSC.all.bcf.csi - manta/COLO\_829-Ill.all.bcf.csi - manta/COLO\_829-TGen.all.bcf.csi - manta/COLO\_829-EBI.all.bcf.csi - default-strelka/COLO\_829-GSC.all.bcf.csi - default-strelka/COLO\_829-Ill.all.bcf.csi - default-strelka/COLO\_829-TGen.all.bcf.csi - default-strelka/COLO\_829-EBI.all.bcf.csi - bpi/COLO\_829-GSC.all.bcf.csi - bpi/COLO\_829-Ill.all.bcf.csi - bpi/COLO\_829-TGen.all.bcf.csi - bpi/COLO\_829-EBI.all.bcf.csi - manta/synthetic-5.all.bcf.csi - manta/synthetic-20.all.bcf.csi |  | - bcftools =1.6 - samtools =1.6 | |  |  | | --- | --- | | ``` 1 ``` | ``` bcftools index {input} ``` | |
| neusomatic\_adhoc | 7 | - adhoc-neusomatic/simulated-bwa.all.bcf - adhoc-neusomatic/synthetic-5.all.bcf - adhoc-neusomatic/synthetic-20.all.bcf - adhoc-neusomatic/COLO\_829-GSC.all.bcf - adhoc-neusomatic/COLO\_829-Ill.all.bcf - adhoc-neusomatic/COLO\_829-TGen.all.bcf - adhoc-neusomatic/COLO\_829-EBI.all.bcf |  | - bcftools ==1.5 | |  |  | | --- | --- | | ```  1  2  3  4  5  6  7  8  9 10 11 12 ``` | ``` __author__ = "Johannes Köster" __copyright__ = "Copyright 2016, Johannes Köster" __email__ = "koester@jimmy.harvard.edu" __license__ = "MIT"   from snakemake.shell import shell   shell(     "bcftools view {snakemake.params} {snakemake.input[0]} "     "-o {snakemake.output[0]}") ``` | |
| annotate\_truth | 2 | - truth/synthetic-5.annotated.vcf - truth/synthetic-20.annotated.vcf |  | - cyvcf2 =0.11.5 - python =3.7 | |  |  | | --- | --- | | ```  1  2  3  4  5  6  7  8  9 10 11 12 13 14 15 16 17 18 19 20 21 22 23 24 25 26 27 28 29 30 31 32 33 34 35 36 37 38 39 40 41 42 43 44 45 46 47 48 49 50 51 52 53 54 55 56 57 58 59 ``` | ``` from cyvcf2 import VCF, Writer import numpy as np   def subclone_vaf(gt):     """Calculate subclone allele frequency"""     if np.all(gt[:2] == [1, 1]):         return 1.0     elif (np.all(gt[:2] == [0, 1]) or np.all(gt[:2] == [1, 0]) or           np.all(gt[:2] == [-1, 1]) or np.all(gt[:2] == [1, -1])):         return 0.5     else:         return 0.0   # Reader vcf_in = VCF(snakemake.input[0])  # Setup subclone information subclones = ["Som{}".format(i) for i in range(1, 5)] fractions = [1/3, 1/3, 1/4, 1/12]   # Prepare writer vcf_in.add_info_to_header({"ID": "TAF",                            "Number": "1",                            "Description": "True tumor allele frequency",                            "Type": "Float"}) vcf_in.add_info_to_header({"ID": "NAF",                            "Number": "1",                            "Description": "True normal allele frequency",                            "Type": "Float"}) bcf_out = Writer(snakemake.output[0], vcf_in)  for rec in vcf_in:     if len(rec.ALT) > 1:         raise ValueError("multiallelic sites are not supported at the moment")      try:         # get VAFs from VCF         tumor_vaf = rec.INFO["TAF"]         normal_vaf = rec.INFO["NAF"]     except KeyError:         # calculate VAFs         subclone_idx = [vcf_in.samples.index(s) for s in subclones]         control_idx = vcf_in.samples.index("Control")          tumor_vaf = sum(fraction * subclone_vaf(rec.genotypes[idx])                         for idx, fraction in zip(subclone_idx, fractions))         normal_vaf = subclone_vaf(rec.genotypes[control_idx])          rec.INFO["TAF"] = tumor_vaf         rec.INFO["NAF"] = normal_vaf              # only keep somatic variants     if normal_vaf == 0.0 and tumor_vaf > 0.0:         bcf_out.write_record(rec)  bcf_out.close() ``` | |
| merge\_lancet | 6 | - default-lancet/synthetic-5.all.bcf - default-lancet/synthetic-20.all.bcf - default-lancet/COLO\_829-GSC.all.bcf - default-lancet/COLO\_829-Ill.all.bcf - default-lancet/COLO\_829-TGen.all.bcf - default-lancet/COLO\_829-EBI.all.bcf |  | - bcftools =1.6 - samtools =1.6 | |  |  | | --- | --- | | ``` 1 ``` | ``` bcftools concat -Ob {input} > {output} ``` | |
| manta\_default | 6 | - default-manta/synthetic-5.all.bcf - default-manta/synthetic-20.all.bcf - default-manta/COLO\_829-GSC.all.bcf - default-manta/COLO\_829-Ill.all.bcf - default-manta/COLO\_829-TGen.all.bcf - default-manta/COLO\_829-EBI.all.bcf |  | - bcftools ==1.5 | |  |  | | --- | --- | | ```  1  2  3  4  5  6  7  8  9 10 11 12 ``` | ``` __author__ = "Johannes Köster" __copyright__ = "Copyright 2016, Johannes Köster" __email__ = "koester@jimmy.harvard.edu" __license__ = "MIT"   from snakemake.shell import shell   shell(     "bcftools view {snakemake.params} {snakemake.input[0]} "     "-o {snakemake.output[0]}") ``` | |
| strelka\_default | 6 | - default-strelka/synthetic-5.all.bcf - default-strelka/synthetic-20.all.bcf - default-strelka/COLO\_829-GSC.all.bcf - default-strelka/COLO\_829-Ill.all.bcf - default-strelka/COLO\_829-TGen.all.bcf - default-strelka/COLO\_829-EBI.all.bcf |  | - bcftools ==1.6 | |  |  | | --- | --- | | ```  1  2  3  4  5  6  7  8  9 10 11 12 ``` | ``` __author__ = "Johannes Köster" __copyright__ = "Copyright 2016, Johannes Köster" __email__ = "koester@jimmy.harvard.edu" __license__ = "MIT"   from snakemake.shell import shell   shell(     "bcftools concat {snakemake.params} -o {snakemake.output[0]} "     "{snakemake.input.calls}") ``` | |
| delly\_adhoc | 6 | - adhoc-delly/synthetic-5.all.bcf - adhoc-delly/synthetic-20.all.bcf - adhoc-delly/COLO\_829-GSC.all.bcf - adhoc-delly/COLO\_829-Ill.all.bcf - adhoc-delly/COLO\_829-TGen.all.bcf - adhoc-delly/COLO\_829-EBI.all.bcf |  | - delly =0.7.7 - bcftools =1.6 | |  |  | | --- | --- | | ``` 1 ``` | ``` delly filter -m 0 -r 1.0 --samples {input.samples} -o {params.tmp} {input.bcf}; bcftools view -i INFO/SOMATIC -f PASS -Ob {params.tmp} > {output} ``` | |
| lancet\_adhoc | 6 | - adhoc-lancet/synthetic-5.all.bcf - adhoc-lancet/synthetic-20.all.bcf - adhoc-lancet/COLO\_829-GSC.all.bcf - adhoc-lancet/COLO\_829-Ill.all.bcf - adhoc-lancet/COLO\_829-TGen.all.bcf - adhoc-lancet/COLO\_829-EBI.all.bcf |  | - bcftools ==1.5 | |  |  | | --- | --- | | ```  1  2  3  4  5  6  7  8  9 10 11 12 ``` | ``` __author__ = "Johannes Köster" __copyright__ = "Copyright 2016, Johannes Köster" __email__ = "koester@jimmy.harvard.edu" __license__ = "MIT"   from snakemake.shell import shell   shell(     "bcftools view {snakemake.params} {snakemake.input[0]} "     "-o {snakemake.output[0]}") ``` | |
| manta\_adhoc | 6 | - adhoc-manta/synthetic-5.all.bcf - adhoc-manta/synthetic-20.all.bcf - adhoc-manta/COLO\_829-GSC.all.bcf - adhoc-manta/COLO\_829-Ill.all.bcf - adhoc-manta/COLO\_829-TGen.all.bcf - adhoc-manta/COLO\_829-EBI.all.bcf |  | - bcftools ==1.5 | |  |  | | --- | --- | | ```  1  2  3  4  5  6  7  8  9 10 11 12 ``` | ``` __author__ = "Johannes Köster" __copyright__ = "Copyright 2016, Johannes Köster" __email__ = "koester@jimmy.harvard.edu" __license__ = "MIT"   from snakemake.shell import shell   shell(     "bcftools view {snakemake.params} {snakemake.input[0]} "     "-o {snakemake.output[0]}") ``` | |
| strelka\_adhoc | 6 | - adhoc-strelka/synthetic-5.all.bcf - adhoc-strelka/synthetic-20.all.bcf - adhoc-strelka/COLO\_829-GSC.all.bcf - adhoc-strelka/COLO\_829-Ill.all.bcf - adhoc-strelka/COLO\_829-TGen.all.bcf - adhoc-strelka/COLO\_829-EBI.all.bcf |  | - bcftools ==1.5 | |  |  | | --- | --- | | ```  1  2  3  4  5  6  7  8  9 10 11 12 ``` | ``` __author__ = "Johannes Köster" __copyright__ = "Copyright 2016, Johannes Köster" __email__ = "koester@jimmy.harvard.edu" __license__ = "MIT"   from snakemake.shell import shell   shell(     "bcftools view {snakemake.params} {snakemake.input[0]} "     "-o {snakemake.output[0]}") ``` | |
| bpi\_adhoc | 6 | - adhoc-bpi/synthetic-5.all.bcf - adhoc-bpi/synthetic-20.all.bcf - adhoc-bpi/COLO\_829-GSC.all.bcf - adhoc-bpi/COLO\_829-Ill.all.bcf - adhoc-bpi/COLO\_829-TGen.all.bcf - adhoc-bpi/COLO\_829-EBI.all.bcf |  | - bcftools ==1.5 | |  |  | | --- | --- | | ```  1  2  3  4  5  6  7  8  9 10 11 12 ``` | ``` __author__ = "Johannes Köster" __copyright__ = "Copyright 2016, Johannes Köster" __email__ = "koester@jimmy.harvard.edu" __license__ = "MIT"   from snakemake.shell import shell   shell(     "bcftools view {snakemake.params} {snakemake.input[0]} "     "-o {snakemake.output[0]}") ``` | |
| concordance\_match | 18 | - concordance/varlociraptor-neusomatic-0.9/colo1.0-vs-1.bcf - concordance/varlociraptor-neusomatic-0.9/colo1.1-vs-2.bcf - concordance/varlociraptor-neusomatic-0.9/colo1.2-vs-0.bcf - concordance/varlociraptor-neusomatic-0.9/colo1.2-vs-3.bcf - concordance/varlociraptor-neusomatic-0.9/colo1.3-vs-0.bcf - concordance/varlociraptor-neusomatic-0.9/colo1.3-vs-1.bcf - concordance/varlociraptor-neusomatic-0.98/colo1.0-vs-1.bcf - concordance/varlociraptor-neusomatic-0.98/colo1.1-vs-2.bcf - concordance/varlociraptor-neusomatic-0.98/colo1.2-vs-0.bcf - concordance/varlociraptor-neusomatic-0.98/colo1.2-vs-3.bcf - concordance/varlociraptor-neusomatic-0.98/colo1.3-vs-0.bcf - concordance/varlociraptor-neusomatic-0.98/colo1.3-vs-1.bcf - concordance/adhoc-neusomatic-default/colo1.0-vs-1.bcf - concordance/adhoc-neusomatic-default/colo1.1-vs-2.bcf - concordance/adhoc-neusomatic-default/colo1.2-vs-0.bcf - concordance/adhoc-neusomatic-default/colo1.2-vs-3.bcf - concordance/adhoc-neusomatic-default/colo1.3-vs-0.bcf - concordance/adhoc-neusomatic-default/colo1.3-vs-1.bcf |  | - rust-bio-tools =0.9.0 - bedtools =2.27.1 - bcftools =1.8 | |  |  | | --- | --- | | ``` 1 ``` | ``` rbt vcf-match {params.match} {params.bcfs[1]} < {params.bcfs[0]} > {output} ``` | |
| concordance\_match | 60 | - concordance/varlociraptor-delly-0.9/colo1.0-vs-1.bcf - concordance/varlociraptor-delly-0.9/colo1.1-vs-2.bcf - concordance/varlociraptor-delly-0.9/colo1.2-vs-0.bcf - concordance/varlociraptor-delly-0.9/colo1.2-vs-3.bcf - concordance/varlociraptor-delly-0.9/colo1.3-vs-0.bcf - concordance/varlociraptor-delly-0.9/colo1.3-vs-1.bcf - concordance/varlociraptor-lancet-0.9/colo1.0-vs-1.bcf - concordance/varlociraptor-lancet-0.9/colo1.1-vs-2.bcf - concordance/varlociraptor-lancet-0.9/colo1.2-vs-0.bcf - concordance/varlociraptor-lancet-0.9/colo1.2-vs-3.bcf - concordance/varlociraptor-lancet-0.9/colo1.3-vs-0.bcf - concordance/varlociraptor-lancet-0.9/colo1.3-vs-1.bcf - concordance/varlociraptor-manta-0.9/colo1.0-vs-1.bcf - concordance/varlociraptor-manta-0.9/colo1.1-vs-2.bcf - concordance/varlociraptor-manta-0.9/colo1.2-vs-0.bcf - concordance/varlociraptor-manta-0.9/colo1.2-vs-3.bcf - concordance/varlociraptor-manta-0.9/colo1.3-vs-0.bcf - concordance/varlociraptor-manta-0.9/colo1.3-vs-1.bcf - concordance/varlociraptor-strelka-0.9/colo1.0-vs-1.bcf - concordance/varlociraptor-strelka-0.9/colo1.1-vs-2.bcf - concordance/varlociraptor-strelka-0.9/colo1.2-vs-0.bcf - concordance/varlociraptor-strelka-0.9/colo1.2-vs-3.bcf - concordance/varlociraptor-strelka-0.9/colo1.3-vs-0.bcf - concordance/varlociraptor-strelka-0.9/colo1.3-vs-1.bcf - concordance/varlociraptor-bpi-0.9/colo1.0-vs-1.bcf - concordance/varlociraptor-bpi-0.9/colo1.1-vs-2.bcf - concordance/varlociraptor-bpi-0.9/colo1.2-vs-0.bcf - concordance/varlociraptor-bpi-0.9/colo1.2-vs-3.bcf - concordance/varlociraptor-bpi-0.9/colo1.3-vs-0.bcf - concordance/varlociraptor-bpi-0.9/colo1.3-vs-1.bcf - concordance/varlociraptor-delly-0.98/colo1.0-vs-1.bcf - concordance/varlociraptor-delly-0.98/colo1.1-vs-2.bcf - concordance/varlociraptor-delly-0.98/colo1.2-vs-0.bcf - concordance/varlociraptor-delly-0.98/colo1.2-vs-3.bcf - concordance/varlociraptor-delly-0.98/colo1.3-vs-0.bcf - concordance/varlociraptor-delly-0.98/colo1.3-vs-1.bcf - concordance/varlociraptor-lancet-0.98/colo1.0-vs-1.bcf - concordance/varlociraptor-lancet-0.98/colo1.1-vs-2.bcf - concordance/varlociraptor-lancet-0.98/colo1.2-vs-0.bcf - concordance/varlociraptor-lancet-0.98/colo1.2-vs-3.bcf - concordance/varlociraptor-lancet-0.98/colo1.3-vs-0.bcf - concordance/varlociraptor-lancet-0.98/colo1.3-vs-1.bcf - concordance/varlociraptor-manta-0.98/colo1.0-vs-1.bcf - concordance/varlociraptor-manta-0.98/colo1.1-vs-2.bcf - concordance/varlociraptor-manta-0.98/colo1.2-vs-0.bcf - concordance/varlociraptor-manta-0.98/colo1.2-vs-3.bcf - concordance/varlociraptor-manta-0.98/colo1.3-vs-0.bcf - concordance/varlociraptor-manta-0.98/colo1.3-vs-1.bcf - concordance/varlociraptor-strelka-0.98/colo1.0-vs-1.bcf - concordance/varlociraptor-strelka-0.98/colo1.1-vs-2.bcf - concordance/varlociraptor-strelka-0.98/colo1.2-vs-0.bcf - concordance/varlociraptor-strelka-0.98/colo1.2-vs-3.bcf - concordance/varlociraptor-strelka-0.98/colo1.3-vs-0.bcf - concordance/varlociraptor-strelka-0.98/colo1.3-vs-1.bcf - concordance/varlociraptor-bpi-0.98/colo1.0-vs-1.bcf - concordance/varlociraptor-bpi-0.98/colo1.1-vs-2.bcf - concordance/varlociraptor-bpi-0.98/colo1.2-vs-0.bcf - concordance/varlociraptor-bpi-0.98/colo1.2-vs-3.bcf - concordance/varlociraptor-bpi-0.98/colo1.3-vs-0.bcf - concordance/varlociraptor-bpi-0.98/colo1.3-vs-1.bcf |  | - rust-bio-tools =0.5.0 - bedtools =2.27.1 - bcftools =1.8 | |  |  | | --- | --- | | ``` 1 ``` | ``` rbt vcf-match {params.match} {params.bcfs[1]} < {params.bcfs[0]} > {output} ``` | |
| concordance\_match | 30 | - concordance/adhoc-delly-default/colo1.0-vs-1.bcf - concordance/adhoc-delly-default/colo1.1-vs-2.bcf - concordance/adhoc-delly-default/colo1.2-vs-0.bcf - concordance/adhoc-delly-default/colo1.2-vs-3.bcf - concordance/adhoc-delly-default/colo1.3-vs-0.bcf - concordance/adhoc-delly-default/colo1.3-vs-1.bcf - concordance/adhoc-lancet-default/colo1.0-vs-1.bcf - concordance/adhoc-lancet-default/colo1.1-vs-2.bcf - concordance/adhoc-lancet-default/colo1.2-vs-0.bcf - concordance/adhoc-lancet-default/colo1.2-vs-3.bcf - concordance/adhoc-lancet-default/colo1.3-vs-0.bcf - concordance/adhoc-lancet-default/colo1.3-vs-1.bcf - concordance/adhoc-manta-default/colo1.0-vs-1.bcf - concordance/adhoc-manta-default/colo1.1-vs-2.bcf - concordance/adhoc-manta-default/colo1.2-vs-0.bcf - concordance/adhoc-manta-default/colo1.2-vs-3.bcf - concordance/adhoc-manta-default/colo1.3-vs-0.bcf - concordance/adhoc-manta-default/colo1.3-vs-1.bcf - concordance/adhoc-strelka-default/colo1.0-vs-1.bcf - concordance/adhoc-strelka-default/colo1.1-vs-2.bcf - concordance/adhoc-strelka-default/colo1.2-vs-0.bcf - concordance/adhoc-strelka-default/colo1.2-vs-3.bcf - concordance/adhoc-strelka-default/colo1.3-vs-0.bcf - concordance/adhoc-strelka-default/colo1.3-vs-1.bcf - concordance/adhoc-bpi-default/colo1.0-vs-1.bcf - concordance/adhoc-bpi-default/colo1.1-vs-2.bcf - concordance/adhoc-bpi-default/colo1.2-vs-0.bcf - concordance/adhoc-bpi-default/colo1.2-vs-3.bcf - concordance/adhoc-bpi-default/colo1.3-vs-0.bcf - concordance/adhoc-bpi-default/colo1.3-vs-1.bcf |  | - rust-bio-tools =0.2.5 - bedtools =2.27.1 - bcftools =1.8 | |  |  | | --- | --- | | ``` 1 ``` | ``` rbt vcf-match {params.match} {params.bcfs[1]} < {params.bcfs[0]} > {output} ``` | |
| varlociraptor\_merge | 39 | - varlociraptor-neusomatic/COLO\_829-GSC.all.bcf - varlociraptor-neusomatic/COLO\_829-Ill.all.bcf - varlociraptor-neusomatic/COLO\_829-TGen.all.bcf - varlociraptor-neusomatic/COLO\_829-EBI.all.bcf - varlociraptor-delly/COLO\_829-GSC.all.bcf - varlociraptor-delly/COLO\_829-Ill.all.bcf - varlociraptor-delly/COLO\_829-TGen.all.bcf - varlociraptor-delly/COLO\_829-EBI.all.bcf - varlociraptor-lancet/COLO\_829-GSC.all.bcf - varlociraptor-lancet/COLO\_829-Ill.all.bcf - varlociraptor-lancet/COLO\_829-TGen.all.bcf - varlociraptor-lancet/COLO\_829-EBI.all.bcf - varlociraptor-manta/COLO\_829-GSC.all.bcf - varlociraptor-manta/COLO\_829-Ill.all.bcf - varlociraptor-manta/COLO\_829-TGen.all.bcf - varlociraptor-manta/COLO\_829-EBI.all.bcf - varlociraptor-strelka/COLO\_829-GSC.all.bcf - varlociraptor-strelka/COLO\_829-Ill.all.bcf - varlociraptor-strelka/COLO\_829-TGen.all.bcf - varlociraptor-strelka/COLO\_829-EBI.all.bcf - varlociraptor-bpi/COLO\_829-GSC.all.bcf - varlociraptor-bpi/COLO\_829-Ill.all.bcf - varlociraptor-bpi/COLO\_829-TGen.all.bcf - varlociraptor-bpi/COLO\_829-EBI.all.bcf - varlociraptor-neusomatic/simulated-bwa.all.bcf - varlociraptor-delly/simulated-bwa.all.bcf - varlociraptor-lancet/simulated-bwa.all.bcf - varlociraptor-manta/simulated-bwa.all.bcf - varlociraptor-strelka/simulated-bwa.all.bcf - varlociraptor-neusomatic/synthetic-5.all.bcf - varlociraptor-delly/synthetic-5.all.bcf - varlociraptor-lancet/synthetic-5.all.bcf - varlociraptor-manta/synthetic-5.all.bcf - varlociraptor-strelka/synthetic-5.all.bcf - varlociraptor-neusomatic/synthetic-20.all.bcf - varlociraptor-delly/synthetic-20.all.bcf - varlociraptor-lancet/synthetic-20.all.bcf - varlociraptor-manta/synthetic-20.all.bcf - varlociraptor-strelka/synthetic-20.all.bcf |  | - bcftools ==1.5 | |  |  | | --- | --- | | ```  1  2  3  4  5  6  7  8  9 10 11 12 ``` | ``` __author__ = "Johannes Köster" __copyright__ = "Copyright 2016, Johannes Köster" __email__ = "koester@jimmy.harvard.edu" __license__ = "MIT"   from snakemake.shell import shell   shell(     "bcftools concat {snakemake.params} -o {snakemake.output[0]} "     "{snakemake.input}") ``` | |
| neusomatic | 7 | - neusomatic/simulated-bwa - neusomatic/simulated-bwa.all.vcf - neusomatic/synthetic-5 - neusomatic/synthetic-5.all.vcf - neusomatic/synthetic-20 - neusomatic/synthetic-20.all.vcf - neusomatic/COLO\_829-GSC - neusomatic/COLO\_829-GSC.all.vcf - neusomatic/COLO\_829-Ill - neusomatic/COLO\_829-Ill.all.vcf - neusomatic/COLO\_829-TGen - neusomatic/COLO\_829-TGen.all.vcf - neusomatic/COLO\_829-EBI - neusomatic/COLO\_829-EBI.all.vcf | docker://msahraeian/neusomatic:0.2.1 |  | |  |  | | --- | --- | | ``` 1 2 3 4 5 6 ``` | ```         (preprocess.py --mode call --reference {input.ref}                       --region_bed {input.bed} --tumor_bam {input.bams[0]}                       --normal_bam {input.bams[1]} --work {output.workdir}                       --min_mapq 10 --num_threads {threads}                       --scan_alignments_binary /opt/neusomatic/neusomatic/bin/scan_alignments          call.py --candidates_tsv {output.workdir}/dataset/*/candidates*.tsv                 --reference {input.ref} --out {output.workdir}                 --checkpoint /opt/neusomatic/neusomatic/models/NeuSomatic_v0.1.4_standalone_SEQC-WGS-Spike.pth                 --num_threads {threads}                 --batch_size 100          python `which postprocess.py` --reference {input.ref} --tumor_bam {input.bams[0]}                        --pred_vcf {output.workdir}/pred.vcf                        --candidates_vcf {output.workdir}/work_tumor/filtered_candidates.vcf                        --output_vcf {output.vcf}                        --work {output.workdir}) 2> {log} ``` | |
| fix\_lancet | 150 | - lancet/synthetic-5/chr1.fixed.vcf - lancet/synthetic-5/chr2.fixed.vcf - lancet/synthetic-5/chr3.fixed.vcf - lancet/synthetic-5/chr4.fixed.vcf - lancet/synthetic-5/chr5.fixed.vcf - lancet/synthetic-5/chr6.fixed.vcf - lancet/synthetic-5/chr7.fixed.vcf - lancet/synthetic-5/chr8.fixed.vcf - lancet/synthetic-5/chr9.fixed.vcf - lancet/synthetic-5/chr10.fixed.vcf - lancet/synthetic-5/chr11.fixed.vcf - lancet/synthetic-5/chr12.fixed.vcf - lancet/synthetic-5/chr13.fixed.vcf - lancet/synthetic-5/chr14.fixed.vcf - lancet/synthetic-5/chr15.fixed.vcf - lancet/synthetic-5/chr16.fixed.vcf - lancet/synthetic-5/chr17.fixed.vcf - lancet/synthetic-5/chr18.fixed.vcf - lancet/synthetic-5/chr19.fixed.vcf - lancet/synthetic-5/chr20.fixed.vcf - lancet/synthetic-5/chr21.fixed.vcf - lancet/synthetic-5/chr22.fixed.vcf - lancet/synthetic-5/chrM.fixed.vcf - lancet/synthetic-5/chrX.fixed.vcf - lancet/synthetic-5/chrY.fixed.vcf - lancet/synthetic-20/chr1.fixed.vcf - lancet/synthetic-20/chr2.fixed.vcf - lancet/synthetic-20/chr3.fixed.vcf - lancet/synthetic-20/chr4.fixed.vcf - lancet/synthetic-20/chr5.fixed.vcf - lancet/synthetic-20/chr6.fixed.vcf - lancet/synthetic-20/chr7.fixed.vcf - lancet/synthetic-20/chr8.fixed.vcf - lancet/synthetic-20/chr9.fixed.vcf - lancet/synthetic-20/chr10.fixed.vcf - lancet/synthetic-20/chr11.fixed.vcf - lancet/synthetic-20/chr12.fixed.vcf - lancet/synthetic-20/chr13.fixed.vcf - lancet/synthetic-20/chr14.fixed.vcf - lancet/synthetic-20/chr15.fixed.vcf - lancet/synthetic-20/chr16.fixed.vcf - lancet/synthetic-20/chr17.fixed.vcf - lancet/synthetic-20/chr18.fixed.vcf - lancet/synthetic-20/chr19.fixed.vcf - lancet/synthetic-20/chr20.fixed.vcf - lancet/synthetic-20/chr21.fixed.vcf - lancet/synthetic-20/chr22.fixed.vcf - lancet/synthetic-20/chrM.fixed.vcf - lancet/synthetic-20/chrX.fixed.vcf - lancet/synthetic-20/chrY.fixed.vcf - lancet/COLO\_829-GSC/chr1.fixed.vcf - lancet/COLO\_829-GSC/chr2.fixed.vcf - lancet/COLO\_829-GSC/chr3.fixed.vcf - lancet/COLO\_829-GSC/chr4.fixed.vcf - lancet/COLO\_829-GSC/chr5.fixed.vcf - lancet/COLO\_829-GSC/chr6.fixed.vcf - lancet/COLO\_829-GSC/chr7.fixed.vcf - lancet/COLO\_829-GSC/chr8.fixed.vcf - lancet/COLO\_829-GSC/chr9.fixed.vcf - lancet/COLO\_829-GSC/chr10.fixed.vcf - lancet/COLO\_829-GSC/chr11.fixed.vcf - lancet/COLO\_829-GSC/chr12.fixed.vcf - lancet/COLO\_829-GSC/chr13.fixed.vcf - lancet/COLO\_829-GSC/chr14.fixed.vcf - lancet/COLO\_829-GSC/chr15.fixed.vcf - lancet/COLO\_829-GSC/chr16.fixed.vcf - lancet/COLO\_829-GSC/chr17.fixed.vcf - lancet/COLO\_829-GSC/chr18.fixed.vcf - lancet/COLO\_829-GSC/chr19.fixed.vcf - lancet/COLO\_829-GSC/chr20.fixed.vcf - lancet/COLO\_829-GSC/chr21.fixed.vcf - lancet/COLO\_829-GSC/chr22.fixed.vcf - lancet/COLO\_829-GSC/chrM.fixed.vcf - lancet/COLO\_829-GSC/chrX.fixed.vcf - lancet/COLO\_829-GSC/chrY.fixed.vcf - lancet/COLO\_829-Ill/chr1.fixed.vcf - lancet/COLO\_829-Ill/chr2.fixed.vcf - lancet/COLO\_829-Ill/chr3.fixed.vcf - lancet/COLO\_829-Ill/chr4.fixed.vcf - lancet/COLO\_829-Ill/chr5.fixed.vcf - lancet/COLO\_829-Ill/chr6.fixed.vcf - lancet/COLO\_829-Ill/chr7.fixed.vcf - lancet/COLO\_829-Ill/chr8.fixed.vcf - lancet/COLO\_829-Ill/chr9.fixed.vcf - lancet/COLO\_829-Ill/chr10.fixed.vcf - lancet/COLO\_829-Ill/chr11.fixed.vcf - lancet/COLO\_829-Ill/chr12.fixed.vcf - lancet/COLO\_829-Ill/chr13.fixed.vcf - lancet/COLO\_829-Ill/chr14.fixed.vcf - lancet/COLO\_829-Ill/chr15.fixed.vcf - lancet/COLO\_829-Ill/chr16.fixed.vcf - lancet/COLO\_829-Ill/chr17.fixed.vcf - lancet/COLO\_829-Ill/chr18.fixed.vcf - lancet/COLO\_829-Ill/chr19.fixed.vcf - lancet/COLO\_829-Ill/chr20.fixed.vcf - lancet/COLO\_829-Ill/chr21.fixed.vcf - lancet/COLO\_829-Ill/chr22.fixed.vcf - lancet/COLO\_829-Ill/chrM.fixed.vcf - lancet/COLO\_829-Ill/chrX.fixed.vcf - lancet/COLO\_829-Ill/chrY.fixed.vcf - lancet/COLO\_829-TGen/chr1.fixed.vcf - lancet/COLO\_829-TGen/chr2.fixed.vcf - lancet/COLO\_829-TGen/chr3.fixed.vcf - lancet/COLO\_829-TGen/chr4.fixed.vcf - lancet/COLO\_829-TGen/chr5.fixed.vcf - lancet/COLO\_829-TGen/chr6.fixed.vcf - lancet/COLO\_829-TGen/chr7.fixed.vcf - lancet/COLO\_829-TGen/chr8.fixed.vcf - lancet/COLO\_829-TGen/chr9.fixed.vcf - lancet/COLO\_829-TGen/chr10.fixed.vcf - lancet/COLO\_829-TGen/chr11.fixed.vcf - lancet/COLO\_829-TGen/chr12.fixed.vcf - lancet/COLO\_829-TGen/chr13.fixed.vcf - lancet/COLO\_829-TGen/chr14.fixed.vcf - lancet/COLO\_829-TGen/chr15.fixed.vcf - lancet/COLO\_829-TGen/chr16.fixed.vcf - lancet/COLO\_829-TGen/chr17.fixed.vcf - lancet/COLO\_829-TGen/chr18.fixed.vcf - lancet/COLO\_829-TGen/chr19.fixed.vcf - lancet/COLO\_829-TGen/chr20.fixed.vcf - lancet/COLO\_829-TGen/chr21.fixed.vcf - lancet/COLO\_829-TGen/chr22.fixed.vcf - lancet/COLO\_829-TGen/chrM.fixed.vcf - lancet/COLO\_829-TGen/chrX.fixed.vcf - lancet/COLO\_829-TGen/chrY.fixed.vcf - lancet/COLO\_829-EBI/chr1.fixed.vcf - lancet/COLO\_829-EBI/chr2.fixed.vcf - lancet/COLO\_829-EBI/chr3.fixed.vcf - lancet/COLO\_829-EBI/chr4.fixed.vcf - lancet/COLO\_829-EBI/chr5.fixed.vcf - lancet/COLO\_829-EBI/chr6.fixed.vcf - lancet/COLO\_829-EBI/chr7.fixed.vcf - lancet/COLO\_829-EBI/chr8.fixed.vcf - lancet/COLO\_829-EBI/chr9.fixed.vcf - lancet/COLO\_829-EBI/chr10.fixed.vcf - lancet/COLO\_829-EBI/chr11.fixed.vcf - lancet/COLO\_829-EBI/chr12.fixed.vcf - lancet/COLO\_829-EBI/chr13.fixed.vcf - lancet/COLO\_829-EBI/chr14.fixed.vcf - lancet/COLO\_829-EBI/chr15.fixed.vcf - lancet/COLO\_829-EBI/chr16.fixed.vcf - lancet/COLO\_829-EBI/chr17.fixed.vcf - lancet/COLO\_829-EBI/chr18.fixed.vcf - lancet/COLO\_829-EBI/chr19.fixed.vcf - lancet/COLO\_829-EBI/chr20.fixed.vcf - lancet/COLO\_829-EBI/chr21.fixed.vcf - lancet/COLO\_829-EBI/chr22.fixed.vcf - lancet/COLO\_829-EBI/chrM.fixed.vcf - lancet/COLO\_829-EBI/chrX.fixed.vcf - lancet/COLO\_829-EBI/chrY.fixed.vcf |  | - bcftools =1.6 - samtools =1.6 | |  |  | | --- | --- | | ``` 1 ``` | ``` sed -r 's/MS\=[0-9]+[ACGT]+/MS/g' {input.vcf} | bcftools annotate -o {output} -h {input.header} - ``` | |
| manta | 6 | - manta/synthetic-5/results/variants/candidateSV.vcf.gz - manta/synthetic-5/results/variants/somaticSV.vcf.gz - manta/synthetic-5/results/variants/candidateSmallIndels.vcf.gz - manta/synthetic-20/results/variants/candidateSV.vcf.gz - manta/synthetic-20/results/variants/somaticSV.vcf.gz - manta/synthetic-20/results/variants/candidateSmallIndels.vcf.gz - manta/COLO\_829-GSC/results/variants/candidateSV.vcf.gz - manta/COLO\_829-GSC/results/variants/somaticSV.vcf.gz - manta/COLO\_829-GSC/results/variants/candidateSmallIndels.vcf.gz - manta/COLO\_829-Ill/results/variants/candidateSV.vcf.gz - manta/COLO\_829-Ill/results/variants/somaticSV.vcf.gz - manta/COLO\_829-Ill/results/variants/candidateSmallIndels.vcf.gz - manta/COLO\_829-TGen/results/variants/candidateSV.vcf.gz - manta/COLO\_829-TGen/results/variants/somaticSV.vcf.gz - manta/COLO\_829-TGen/results/variants/candidateSmallIndels.vcf.gz - manta/COLO\_829-EBI/results/variants/candidateSV.vcf.gz - manta/COLO\_829-EBI/results/variants/somaticSV.vcf.gz - manta/COLO\_829-EBI/results/variants/candidateSmallIndels.vcf.gz |  | - manta =1.3.0 | |  |  | | --- | --- | | ``` 1 ``` | ``` rm -rf {params.dir}; (configManta.py {params.extra} --tumorBam {input.samples[0]} --normalBam {input.samples[1]} --referenceFasta {input.ref} --runDir {params.dir}; {params.dir}/runWorkflow.py -m local -j {threads}) > {log} 2>&1 ``` | |
| strelka | 6 | - strelka/synthetic-5/results/variants/somatic.snvs.vcf.gz - strelka/synthetic-5/results/variants/somatic.indels.vcf.gz - strelka/synthetic-20/results/variants/somatic.snvs.vcf.gz - strelka/synthetic-20/results/variants/somatic.indels.vcf.gz - strelka/COLO\_829-GSC/results/variants/somatic.snvs.vcf.gz - strelka/COLO\_829-GSC/results/variants/somatic.indels.vcf.gz - strelka/COLO\_829-Ill/results/variants/somatic.snvs.vcf.gz - strelka/COLO\_829-Ill/results/variants/somatic.indels.vcf.gz - strelka/COLO\_829-TGen/results/variants/somatic.snvs.vcf.gz - strelka/COLO\_829-TGen/results/variants/somatic.indels.vcf.gz - strelka/COLO\_829-EBI/results/variants/somatic.snvs.vcf.gz - strelka/COLO\_829-EBI/results/variants/somatic.indels.vcf.gz |  | - strelka =2.8.4 | |  |  | | --- | --- | | ``` 1 ``` | ``` rm -rf {params.dir}; (configureStrelkaSomaticWorkflow.py {params.extra} --tumorBam {input.samples[0]} --normalBam {input.samples[1]} --referenceFasta {input.ref} --runDir {params.dir} --indelCandidates {input.manta}; {params.dir}/runWorkflow.py -m local -j {threads}) > {log} 2>&1 ``` | |
| delly\_concat | 6 | - delly/synthetic-5.all.bcf - delly/synthetic-20.all.bcf - delly/COLO\_829-GSC.all.bcf - delly/COLO\_829-Ill.all.bcf - delly/COLO\_829-TGen.all.bcf - delly/COLO\_829-EBI.all.bcf |  | - bcftools ==1.5 | |  |  | | --- | --- | | ```  1  2  3  4  5  6  7  8  9 10 11 12 ``` | ``` __author__ = "Johannes Köster" __copyright__ = "Copyright 2016, Johannes Köster" __email__ = "koester@jimmy.harvard.edu" __license__ = "MIT"   from snakemake.shell import shell   shell(     "bcftools concat {snakemake.params} -o {snakemake.output[0]} "     "{snakemake.input}") ``` | |
| bpi\_convert | 6 | - bpi/synthetic-5.all.bcf - bpi/synthetic-20.all.bcf - bpi/COLO\_829-GSC.all.bcf - bpi/COLO\_829-Ill.all.bcf - bpi/COLO\_829-TGen.all.bcf - bpi/COLO\_829-EBI.all.bcf |  | - bcftools ==1.5 | |  |  | | --- | --- | | ```  1  2  3  4  5  6  7  8  9 10 11 12 ``` | ``` __author__ = "Johannes Köster" __copyright__ = "Copyright 2016, Johannes Köster" __email__ = "koester@jimmy.harvard.edu" __license__ = "MIT"   from snakemake.shell import shell   shell(     "bcftools view {snakemake.params} {snakemake.input[0]} "     "-o {snakemake.output[0]}") ``` | |
| adhoc\_varlociraptor | 48 | - varlociraptor-neusomatic/COLO\_829-GSC.adhoc.0.9.bcf - varlociraptor-neusomatic/COLO\_829-Ill.adhoc.0.9.bcf - varlociraptor-neusomatic/COLO\_829-TGen.adhoc.0.9.bcf - varlociraptor-neusomatic/COLO\_829-EBI.adhoc.0.9.bcf - varlociraptor-delly/COLO\_829-GSC.adhoc.0.9.bcf - varlociraptor-delly/COLO\_829-Ill.adhoc.0.9.bcf - varlociraptor-delly/COLO\_829-TGen.adhoc.0.9.bcf - varlociraptor-delly/COLO\_829-EBI.adhoc.0.9.bcf - varlociraptor-lancet/COLO\_829-GSC.adhoc.0.9.bcf - varlociraptor-lancet/COLO\_829-Ill.adhoc.0.9.bcf - varlociraptor-lancet/COLO\_829-TGen.adhoc.0.9.bcf - varlociraptor-lancet/COLO\_829-EBI.adhoc.0.9.bcf - varlociraptor-manta/COLO\_829-GSC.adhoc.0.9.bcf - varlociraptor-manta/COLO\_829-Ill.adhoc.0.9.bcf - varlociraptor-manta/COLO\_829-TGen.adhoc.0.9.bcf - varlociraptor-manta/COLO\_829-EBI.adhoc.0.9.bcf - varlociraptor-strelka/COLO\_829-GSC.adhoc.0.9.bcf - varlociraptor-strelka/COLO\_829-Ill.adhoc.0.9.bcf - varlociraptor-strelka/COLO\_829-TGen.adhoc.0.9.bcf - varlociraptor-strelka/COLO\_829-EBI.adhoc.0.9.bcf - varlociraptor-bpi/COLO\_829-GSC.adhoc.0.9.bcf - varlociraptor-bpi/COLO\_829-Ill.adhoc.0.9.bcf - varlociraptor-bpi/COLO\_829-TGen.adhoc.0.9.bcf - varlociraptor-bpi/COLO\_829-EBI.adhoc.0.9.bcf - varlociraptor-neusomatic/COLO\_829-GSC.adhoc.0.98.bcf - varlociraptor-neusomatic/COLO\_829-Ill.adhoc.0.98.bcf - varlociraptor-neusomatic/COLO\_829-TGen.adhoc.0.98.bcf - varlociraptor-neusomatic/COLO\_829-EBI.adhoc.0.98.bcf - varlociraptor-delly/COLO\_829-GSC.adhoc.0.98.bcf - varlociraptor-delly/COLO\_829-Ill.adhoc.0.98.bcf - varlociraptor-delly/COLO\_829-TGen.adhoc.0.98.bcf - varlociraptor-delly/COLO\_829-EBI.adhoc.0.98.bcf - varlociraptor-lancet/COLO\_829-GSC.adhoc.0.98.bcf - varlociraptor-lancet/COLO\_829-Ill.adhoc.0.98.bcf - varlociraptor-lancet/COLO\_829-TGen.adhoc.0.98.bcf - varlociraptor-lancet/COLO\_829-EBI.adhoc.0.98.bcf - varlociraptor-manta/COLO\_829-GSC.adhoc.0.98.bcf - varlociraptor-manta/COLO\_829-Ill.adhoc.0.98.bcf - varlociraptor-manta/COLO\_829-TGen.adhoc.0.98.bcf - varlociraptor-manta/COLO\_829-EBI.adhoc.0.98.bcf - varlociraptor-strelka/COLO\_829-GSC.adhoc.0.98.bcf - varlociraptor-strelka/COLO\_829-Ill.adhoc.0.98.bcf - varlociraptor-strelka/COLO\_829-TGen.adhoc.0.98.bcf - varlociraptor-strelka/COLO\_829-EBI.adhoc.0.98.bcf - varlociraptor-bpi/COLO\_829-GSC.adhoc.0.98.bcf - varlociraptor-bpi/COLO\_829-Ill.adhoc.0.98.bcf - varlociraptor-bpi/COLO\_829-TGen.adhoc.0.98.bcf - varlociraptor-bpi/COLO\_829-EBI.adhoc.0.98.bcf |  | - bcftools ==1.5 | |  |  | | --- | --- | | ```  1  2  3  4  5  6  7  8  9 10 11 12 ``` | ``` __author__ = "Johannes Köster" __copyright__ = "Copyright 2016, Johannes Köster" __email__ = "koester@jimmy.harvard.edu" __license__ = "MIT"   from snakemake.shell import shell   shell(     "bcftools view {snakemake.params} {snakemake.input[0]} "     "-o {snakemake.output[0]}") ``` | |
| varlociraptor\_call | 375 | - varlociraptor-neusomatic/COLO\_829-GSC.1.bcf - varlociraptor-neusomatic/COLO\_829-GSC.2.bcf - varlociraptor-neusomatic/COLO\_829-GSC.3.bcf - varlociraptor-neusomatic/COLO\_829-GSC.4.bcf - varlociraptor-neusomatic/COLO\_829-GSC.5.bcf - varlociraptor-neusomatic/COLO\_829-GSC.6.bcf - varlociraptor-neusomatic/COLO\_829-GSC.7.bcf - varlociraptor-neusomatic/COLO\_829-GSC.8.bcf - varlociraptor-neusomatic/COLO\_829-GSC.9.bcf - varlociraptor-neusomatic/COLO\_829-GSC.10.bcf - varlociraptor-neusomatic/COLO\_829-GSC.11.bcf - varlociraptor-neusomatic/COLO\_829-GSC.12.bcf - varlociraptor-neusomatic/COLO\_829-GSC.13.bcf - varlociraptor-neusomatic/COLO\_829-GSC.14.bcf - varlociraptor-neusomatic/COLO\_829-GSC.15.bcf - varlociraptor-neusomatic/COLO\_829-GSC.16.bcf - varlociraptor-neusomatic/COLO\_829-GSC.17.bcf - varlociraptor-neusomatic/COLO\_829-GSC.18.bcf - varlociraptor-neusomatic/COLO\_829-GSC.19.bcf - varlociraptor-neusomatic/COLO\_829-GSC.20.bcf - varlociraptor-neusomatic/COLO\_829-GSC.21.bcf - varlociraptor-neusomatic/COLO\_829-GSC.22.bcf - varlociraptor-neusomatic/COLO\_829-GSC.M.bcf - varlociraptor-neusomatic/COLO\_829-GSC.X.bcf - varlociraptor-neusomatic/COLO\_829-GSC.Y.bcf - varlociraptor-neusomatic/COLO\_829-Ill.1.bcf - varlociraptor-neusomatic/COLO\_829-Ill.2.bcf - varlociraptor-neusomatic/COLO\_829-Ill.3.bcf - varlociraptor-neusomatic/COLO\_829-Ill.4.bcf - varlociraptor-neusomatic/COLO\_829-Ill.5.bcf - varlociraptor-neusomatic/COLO\_829-Ill.6.bcf - varlociraptor-neusomatic/COLO\_829-Ill.7.bcf - varlociraptor-neusomatic/COLO\_829-Ill.8.bcf - varlociraptor-neusomatic/COLO\_829-Ill.9.bcf - varlociraptor-neusomatic/COLO\_829-Ill.10.bcf - varlociraptor-neusomatic/COLO\_829-Ill.11.bcf - varlociraptor-neusomatic/COLO\_829-Ill.12.bcf - varlociraptor-neusomatic/COLO\_829-Ill.13.bcf - varlociraptor-neusomatic/COLO\_829-Ill.14.bcf - varlociraptor-neusomatic/COLO\_829-Ill.15.bcf - varlociraptor-neusomatic/COLO\_829-Ill.16.bcf - varlociraptor-neusomatic/COLO\_829-Ill.17.bcf - varlociraptor-neusomatic/COLO\_829-Ill.18.bcf - varlociraptor-neusomatic/COLO\_829-Ill.19.bcf - varlociraptor-neusomatic/COLO\_829-Ill.20.bcf - varlociraptor-neusomatic/COLO\_829-Ill.21.bcf - varlociraptor-neusomatic/COLO\_829-Ill.22.bcf - varlociraptor-neusomatic/COLO\_829-Ill.M.bcf - varlociraptor-neusomatic/COLO\_829-Ill.X.bcf - varlociraptor-neusomatic/COLO\_829-Ill.Y.bcf - varlociraptor-neusomatic/COLO\_829-TGen.1.bcf - varlociraptor-neusomatic/COLO\_829-TGen.2.bcf - varlociraptor-neusomatic/COLO\_829-TGen.3.bcf - varlociraptor-neusomatic/COLO\_829-TGen.4.bcf - varlociraptor-neusomatic/COLO\_829-TGen.5.bcf - varlociraptor-neusomatic/COLO\_829-TGen.6.bcf - varlociraptor-neusomatic/COLO\_829-TGen.7.bcf - varlociraptor-neusomatic/COLO\_829-TGen.8.bcf - varlociraptor-neusomatic/COLO\_829-TGen.9.bcf - varlociraptor-neusomatic/COLO\_829-TGen.10.bcf - varlociraptor-neusomatic/COLO\_829-TGen.11.bcf - varlociraptor-neusomatic/COLO\_829-TGen.12.bcf - varlociraptor-neusomatic/COLO\_829-TGen.13.bcf - varlociraptor-neusomatic/COLO\_829-TGen.14.bcf - varlociraptor-neusomatic/COLO\_829-TGen.15.bcf - varlociraptor-neusomatic/COLO\_829-TGen.16.bcf - varlociraptor-neusomatic/COLO\_829-TGen.17.bcf - varlociraptor-neusomatic/COLO\_829-TGen.18.bcf - varlociraptor-neusomatic/COLO\_829-TGen.19.bcf - varlociraptor-neusomatic/COLO\_829-TGen.20.bcf - varlociraptor-neusomatic/COLO\_829-TGen.21.bcf - varlociraptor-neusomatic/COLO\_829-TGen.22.bcf - varlociraptor-neusomatic/COLO\_829-TGen.M.bcf - varlociraptor-neusomatic/COLO\_829-TGen.X.bcf - varlociraptor-neusomatic/COLO\_829-TGen.Y.bcf - varlociraptor-neusomatic/COLO\_829-EBI.1.bcf - varlociraptor-neusomatic/COLO\_829-EBI.2.bcf - varlociraptor-neusomatic/COLO\_829-EBI.3.bcf - varlociraptor-neusomatic/COLO\_829-EBI.4.bcf - varlociraptor-neusomatic/COLO\_829-EBI.5.bcf - varlociraptor-neusomatic/COLO\_829-EBI.6.bcf - varlociraptor-neusomatic/COLO\_829-EBI.7.bcf - varlociraptor-neusomatic/COLO\_829-EBI.8.bcf - varlociraptor-neusomatic/COLO\_829-EBI.9.bcf - varlociraptor-neusomatic/COLO\_829-EBI.10.bcf - varlociraptor-neusomatic/COLO\_829-EBI.11.bcf - varlociraptor-neusomatic/COLO\_829-EBI.12.bcf - varlociraptor-neusomatic/COLO\_829-EBI.13.bcf - varlociraptor-neusomatic/COLO\_829-EBI.14.bcf - varlociraptor-neusomatic/COLO\_829-EBI.15.bcf - varlociraptor-neusomatic/COLO\_829-EBI.16.bcf - varlociraptor-neusomatic/COLO\_829-EBI.17.bcf - varlociraptor-neusomatic/COLO\_829-EBI.18.bcf - varlociraptor-neusomatic/COLO\_829-EBI.19.bcf - varlociraptor-neusomatic/COLO\_829-EBI.20.bcf - varlociraptor-neusomatic/COLO\_829-EBI.21.bcf - varlociraptor-neusomatic/COLO\_829-EBI.22.bcf - varlociraptor-neusomatic/COLO\_829-EBI.M.bcf - varlociraptor-neusomatic/COLO\_829-EBI.X.bcf - varlociraptor-neusomatic/COLO\_829-EBI.Y.bcf - varlociraptor-neusomatic/simulated-bwa.1.bcf - varlociraptor-neusomatic/simulated-bwa.2.bcf - varlociraptor-neusomatic/simulated-bwa.3.bcf - varlociraptor-neusomatic/simulated-bwa.4.bcf - varlociraptor-neusomatic/simulated-bwa.5.bcf - varlociraptor-neusomatic/simulated-bwa.6.bcf - varlociraptor-neusomatic/simulated-bwa.7.bcf - varlociraptor-neusomatic/simulated-bwa.8.bcf - varlociraptor-neusomatic/simulated-bwa.9.bcf - varlociraptor-neusomatic/simulated-bwa.10.bcf - varlociraptor-neusomatic/simulated-bwa.11.bcf - varlociraptor-neusomatic/simulated-bwa.12.bcf - varlociraptor-neusomatic/simulated-bwa.13.bcf - varlociraptor-neusomatic/simulated-bwa.14.bcf - varlociraptor-neusomatic/simulated-bwa.15.bcf - varlociraptor-neusomatic/simulated-bwa.16.bcf - varlociraptor-neusomatic/simulated-bwa.17.bcf - varlociraptor-neusomatic/simulated-bwa.18.bcf - varlociraptor-neusomatic/simulated-bwa.19.bcf - varlociraptor-neusomatic/simulated-bwa.20.bcf - varlociraptor-neusomatic/simulated-bwa.21.bcf - varlociraptor-neusomatic/simulated-bwa.22.bcf - varlociraptor-neusomatic/simulated-bwa.M.bcf - varlociraptor-neusomatic/simulated-bwa.X.bcf - varlociraptor-neusomatic/simulated-bwa.Y.bcf - varlociraptor-neusomatic/synthetic-5.1.bcf - varlociraptor-neusomatic/synthetic-5.2.bcf - varlociraptor-neusomatic/synthetic-5.3.bcf - varlociraptor-neusomatic/synthetic-5.4.bcf - varlociraptor-neusomatic/synthetic-5.5.bcf - varlociraptor-neusomatic/synthetic-5.6.bcf - varlociraptor-neusomatic/synthetic-5.7.bcf - varlociraptor-neusomatic/synthetic-5.8.bcf - varlociraptor-neusomatic/synthetic-5.9.bcf - varlociraptor-neusomatic/synthetic-5.10.bcf - varlociraptor-neusomatic/synthetic-5.11.bcf - varlociraptor-neusomatic/synthetic-5.12.bcf - varlociraptor-neusomatic/synthetic-5.13.bcf - varlociraptor-neusomatic/synthetic-5.14.bcf - varlociraptor-neusomatic/synthetic-5.15.bcf - varlociraptor-neusomatic/synthetic-5.16.bcf - varlociraptor-neusomatic/synthetic-5.17.bcf - varlociraptor-neusomatic/synthetic-5.18.bcf - varlociraptor-neusomatic/synthetic-5.19.bcf - varlociraptor-neusomatic/synthetic-5.20.bcf - varlociraptor-neusomatic/synthetic-5.21.bcf - varlociraptor-neusomatic/synthetic-5.22.bcf - varlociraptor-neusomatic/synthetic-5.M.bcf - varlociraptor-neusomatic/synthetic-5.X.bcf - varlociraptor-neusomatic/synthetic-5.Y.bcf - varlociraptor-delly/synthetic-5.1.bcf - varlociraptor-delly/synthetic-5.2.bcf - varlociraptor-delly/synthetic-5.3.bcf - varlociraptor-delly/synthetic-5.4.bcf - varlociraptor-delly/synthetic-5.5.bcf - varlociraptor-delly/synthetic-5.6.bcf - varlociraptor-delly/synthetic-5.7.bcf - varlociraptor-delly/synthetic-5.8.bcf - varlociraptor-delly/synthetic-5.9.bcf - varlociraptor-delly/synthetic-5.10.bcf - varlociraptor-delly/synthetic-5.11.bcf - varlociraptor-delly/synthetic-5.12.bcf - varlociraptor-delly/synthetic-5.13.bcf - varlociraptor-delly/synthetic-5.14.bcf - varlociraptor-delly/synthetic-5.15.bcf - varlociraptor-delly/synthetic-5.16.bcf - varlociraptor-delly/synthetic-5.17.bcf - varlociraptor-delly/synthetic-5.18.bcf - varlociraptor-delly/synthetic-5.19.bcf - varlociraptor-delly/synthetic-5.20.bcf - varlociraptor-delly/synthetic-5.21.bcf - varlociraptor-delly/synthetic-5.22.bcf - varlociraptor-delly/synthetic-5.M.bcf - varlociraptor-delly/synthetic-5.X.bcf - varlociraptor-delly/synthetic-5.Y.bcf - varlociraptor-lancet/synthetic-5.1.bcf - varlociraptor-lancet/synthetic-5.2.bcf - varlociraptor-lancet/synthetic-5.3.bcf - varlociraptor-lancet/synthetic-5.4.bcf - varlociraptor-lancet/synthetic-5.5.bcf - varlociraptor-lancet/synthetic-5.6.bcf - varlociraptor-lancet/synthetic-5.7.bcf - varlociraptor-lancet/synthetic-5.8.bcf - varlociraptor-lancet/synthetic-5.9.bcf - varlociraptor-lancet/synthetic-5.10.bcf - varlociraptor-lancet/synthetic-5.11.bcf - varlociraptor-lancet/synthetic-5.12.bcf - varlociraptor-lancet/synthetic-5.13.bcf - varlociraptor-lancet/synthetic-5.14.bcf - varlociraptor-lancet/synthetic-5.15.bcf - varlociraptor-lancet/synthetic-5.16.bcf - varlociraptor-lancet/synthetic-5.17.bcf - varlociraptor-lancet/synthetic-5.18.bcf - varlociraptor-lancet/synthetic-5.19.bcf - varlociraptor-lancet/synthetic-5.20.bcf - varlociraptor-lancet/synthetic-5.21.bcf - varlociraptor-lancet/synthetic-5.22.bcf - varlociraptor-lancet/synthetic-5.M.bcf - varlociraptor-lancet/synthetic-5.X.bcf - varlociraptor-lancet/synthetic-5.Y.bcf - varlociraptor-manta/synthetic-5.1.bcf - varlociraptor-manta/synthetic-5.2.bcf - varlociraptor-manta/synthetic-5.3.bcf - varlociraptor-manta/synthetic-5.4.bcf - varlociraptor-manta/synthetic-5.5.bcf - varlociraptor-manta/synthetic-5.6.bcf - varlociraptor-manta/synthetic-5.7.bcf - varlociraptor-manta/synthetic-5.8.bcf - varlociraptor-manta/synthetic-5.9.bcf - varlociraptor-manta/synthetic-5.10.bcf - varlociraptor-manta/synthetic-5.11.bcf - varlociraptor-manta/synthetic-5.12.bcf - varlociraptor-manta/synthetic-5.13.bcf - varlociraptor-manta/synthetic-5.14.bcf - varlociraptor-manta/synthetic-5.15.bcf - varlociraptor-manta/synthetic-5.16.bcf - varlociraptor-manta/synthetic-5.17.bcf - varlociraptor-manta/synthetic-5.18.bcf - varlociraptor-manta/synthetic-5.19.bcf - varlociraptor-manta/synthetic-5.20.bcf - varlociraptor-manta/synthetic-5.21.bcf - varlociraptor-manta/synthetic-5.22.bcf - varlociraptor-manta/synthetic-5.M.bcf - varlociraptor-manta/synthetic-5.X.bcf - varlociraptor-manta/synthetic-5.Y.bcf - varlociraptor-strelka/synthetic-5.1.bcf - varlociraptor-strelka/synthetic-5.2.bcf - varlociraptor-strelka/synthetic-5.3.bcf - varlociraptor-strelka/synthetic-5.4.bcf - varlociraptor-strelka/synthetic-5.5.bcf - varlociraptor-strelka/synthetic-5.6.bcf - varlociraptor-strelka/synthetic-5.7.bcf - varlociraptor-strelka/synthetic-5.8.bcf - varlociraptor-strelka/synthetic-5.9.bcf - varlociraptor-strelka/synthetic-5.10.bcf - varlociraptor-strelka/synthetic-5.11.bcf - varlociraptor-strelka/synthetic-5.12.bcf - varlociraptor-strelka/synthetic-5.13.bcf - varlociraptor-strelka/synthetic-5.14.bcf - varlociraptor-strelka/synthetic-5.15.bcf - varlociraptor-strelka/synthetic-5.16.bcf - varlociraptor-strelka/synthetic-5.17.bcf - varlociraptor-strelka/synthetic-5.18.bcf - varlociraptor-strelka/synthetic-5.19.bcf - varlociraptor-strelka/synthetic-5.20.bcf - varlociraptor-strelka/synthetic-5.21.bcf - varlociraptor-strelka/synthetic-5.22.bcf - varlociraptor-strelka/synthetic-5.M.bcf - varlociraptor-strelka/synthetic-5.X.bcf - varlociraptor-strelka/synthetic-5.Y.bcf - varlociraptor-neusomatic/synthetic-20.1.bcf - varlociraptor-neusomatic/synthetic-20.2.bcf - varlociraptor-neusomatic/synthetic-20.3.bcf - varlociraptor-neusomatic/synthetic-20.4.bcf - varlociraptor-neusomatic/synthetic-20.5.bcf - varlociraptor-neusomatic/synthetic-20.6.bcf - varlociraptor-neusomatic/synthetic-20.7.bcf - varlociraptor-neusomatic/synthetic-20.8.bcf - varlociraptor-neusomatic/synthetic-20.9.bcf - varlociraptor-neusomatic/synthetic-20.10.bcf - varlociraptor-neusomatic/synthetic-20.11.bcf - varlociraptor-neusomatic/synthetic-20.12.bcf - varlociraptor-neusomatic/synthetic-20.13.bcf - varlociraptor-neusomatic/synthetic-20.14.bcf - varlociraptor-neusomatic/synthetic-20.15.bcf - varlociraptor-neusomatic/synthetic-20.16.bcf - varlociraptor-neusomatic/synthetic-20.17.bcf - varlociraptor-neusomatic/synthetic-20.18.bcf - varlociraptor-neusomatic/synthetic-20.19.bcf - varlociraptor-neusomatic/synthetic-20.20.bcf - varlociraptor-neusomatic/synthetic-20.21.bcf - varlociraptor-neusomatic/synthetic-20.22.bcf - varlociraptor-neusomatic/synthetic-20.M.bcf - varlociraptor-neusomatic/synthetic-20.X.bcf - varlociraptor-neusomatic/synthetic-20.Y.bcf - varlociraptor-delly/synthetic-20.1.bcf - varlociraptor-delly/synthetic-20.2.bcf - varlociraptor-delly/synthetic-20.3.bcf - varlociraptor-delly/synthetic-20.4.bcf - varlociraptor-delly/synthetic-20.5.bcf - varlociraptor-delly/synthetic-20.6.bcf - varlociraptor-delly/synthetic-20.7.bcf - varlociraptor-delly/synthetic-20.8.bcf - varlociraptor-delly/synthetic-20.9.bcf - varlociraptor-delly/synthetic-20.10.bcf - varlociraptor-delly/synthetic-20.11.bcf - varlociraptor-delly/synthetic-20.12.bcf - varlociraptor-delly/synthetic-20.13.bcf - varlociraptor-delly/synthetic-20.14.bcf - varlociraptor-delly/synthetic-20.15.bcf - varlociraptor-delly/synthetic-20.16.bcf - varlociraptor-delly/synthetic-20.17.bcf - varlociraptor-delly/synthetic-20.18.bcf - varlociraptor-delly/synthetic-20.19.bcf - varlociraptor-delly/synthetic-20.20.bcf - varlociraptor-delly/synthetic-20.21.bcf - varlociraptor-delly/synthetic-20.22.bcf - varlociraptor-delly/synthetic-20.M.bcf - varlociraptor-delly/synthetic-20.X.bcf - varlociraptor-delly/synthetic-20.Y.bcf - varlociraptor-lancet/synthetic-20.1.bcf - varlociraptor-lancet/synthetic-20.2.bcf - varlociraptor-lancet/synthetic-20.3.bcf - varlociraptor-lancet/synthetic-20.4.bcf - varlociraptor-lancet/synthetic-20.5.bcf - varlociraptor-lancet/synthetic-20.6.bcf - varlociraptor-lancet/synthetic-20.7.bcf - varlociraptor-lancet/synthetic-20.8.bcf - varlociraptor-lancet/synthetic-20.9.bcf - varlociraptor-lancet/synthetic-20.10.bcf - varlociraptor-lancet/synthetic-20.11.bcf - varlociraptor-lancet/synthetic-20.12.bcf - varlociraptor-lancet/synthetic-20.13.bcf - varlociraptor-lancet/synthetic-20.14.bcf - varlociraptor-lancet/synthetic-20.15.bcf - varlociraptor-lancet/synthetic-20.16.bcf - varlociraptor-lancet/synthetic-20.17.bcf - varlociraptor-lancet/synthetic-20.18.bcf - varlociraptor-lancet/synthetic-20.19.bcf - varlociraptor-lancet/synthetic-20.20.bcf - varlociraptor-lancet/synthetic-20.21.bcf - varlociraptor-lancet/synthetic-20.22.bcf - varlociraptor-lancet/synthetic-20.M.bcf - varlociraptor-lancet/synthetic-20.X.bcf - varlociraptor-lancet/synthetic-20.Y.bcf - varlociraptor-manta/synthetic-20.1.bcf - varlociraptor-manta/synthetic-20.2.bcf - varlociraptor-manta/synthetic-20.3.bcf - varlociraptor-manta/synthetic-20.4.bcf - varlociraptor-manta/synthetic-20.5.bcf - varlociraptor-manta/synthetic-20.6.bcf - varlociraptor-manta/synthetic-20.7.bcf - varlociraptor-manta/synthetic-20.8.bcf - varlociraptor-manta/synthetic-20.9.bcf - varlociraptor-manta/synthetic-20.10.bcf - varlociraptor-manta/synthetic-20.11.bcf - varlociraptor-manta/synthetic-20.12.bcf - varlociraptor-manta/synthetic-20.13.bcf - varlociraptor-manta/synthetic-20.14.bcf - varlociraptor-manta/synthetic-20.15.bcf - varlociraptor-manta/synthetic-20.16.bcf - varlociraptor-manta/synthetic-20.17.bcf - varlociraptor-manta/synthetic-20.18.bcf - varlociraptor-manta/synthetic-20.19.bcf - varlociraptor-manta/synthetic-20.20.bcf - varlociraptor-manta/synthetic-20.21.bcf - varlociraptor-manta/synthetic-20.22.bcf - varlociraptor-manta/synthetic-20.M.bcf - varlociraptor-manta/synthetic-20.X.bcf - varlociraptor-manta/synthetic-20.Y.bcf - varlociraptor-strelka/synthetic-20.1.bcf - varlociraptor-strelka/synthetic-20.2.bcf - varlociraptor-strelka/synthetic-20.3.bcf - varlociraptor-strelka/synthetic-20.4.bcf - varlociraptor-strelka/synthetic-20.5.bcf - varlociraptor-strelka/synthetic-20.6.bcf - varlociraptor-strelka/synthetic-20.7.bcf - varlociraptor-strelka/synthetic-20.8.bcf - varlociraptor-strelka/synthetic-20.9.bcf - varlociraptor-strelka/synthetic-20.10.bcf - varlociraptor-strelka/synthetic-20.11.bcf - varlociraptor-strelka/synthetic-20.12.bcf - varlociraptor-strelka/synthetic-20.13.bcf - varlociraptor-strelka/synthetic-20.14.bcf - varlociraptor-strelka/synthetic-20.15.bcf - varlociraptor-strelka/synthetic-20.16.bcf - varlociraptor-strelka/synthetic-20.17.bcf - varlociraptor-strelka/synthetic-20.18.bcf - varlociraptor-strelka/synthetic-20.19.bcf - varlociraptor-strelka/synthetic-20.20.bcf - varlociraptor-strelka/synthetic-20.21.bcf - varlociraptor-strelka/synthetic-20.22.bcf - varlociraptor-strelka/synthetic-20.M.bcf - varlociraptor-strelka/synthetic-20.X.bcf - varlociraptor-strelka/synthetic-20.Y.bcf |  | - varlociraptor =1.1.1 - bcftools =1.9 | |  |  | | --- | --- | | ``` 1 ``` | ``` bcftools view -Ou {input.calls} {params.chrom_prefix} | varlociraptor call variants {input.ref} {config[caller][varlociraptor][params]} {params.caller} tumor-normal {input.bams} --purity {params.purity} > {output} 2> {log} ``` | |
| varlociraptor\_call | 600 | - varlociraptor-delly/COLO\_829-GSC.1.bcf - varlociraptor-delly/COLO\_829-GSC.2.bcf - varlociraptor-delly/COLO\_829-GSC.3.bcf - varlociraptor-delly/COLO\_829-GSC.4.bcf - varlociraptor-delly/COLO\_829-GSC.5.bcf - varlociraptor-delly/COLO\_829-GSC.6.bcf - varlociraptor-delly/COLO\_829-GSC.7.bcf - varlociraptor-delly/COLO\_829-GSC.8.bcf - varlociraptor-delly/COLO\_829-GSC.9.bcf - varlociraptor-delly/COLO\_829-GSC.10.bcf - varlociraptor-delly/COLO\_829-GSC.11.bcf - varlociraptor-delly/COLO\_829-GSC.12.bcf - varlociraptor-delly/COLO\_829-GSC.13.bcf - varlociraptor-delly/COLO\_829-GSC.14.bcf - varlociraptor-delly/COLO\_829-GSC.15.bcf - varlociraptor-delly/COLO\_829-GSC.16.bcf - varlociraptor-delly/COLO\_829-GSC.17.bcf - varlociraptor-delly/COLO\_829-GSC.18.bcf - varlociraptor-delly/COLO\_829-GSC.19.bcf - varlociraptor-delly/COLO\_829-GSC.20.bcf - varlociraptor-delly/COLO\_829-GSC.21.bcf - varlociraptor-delly/COLO\_829-GSC.22.bcf - varlociraptor-delly/COLO\_829-GSC.M.bcf - varlociraptor-delly/COLO\_829-GSC.X.bcf - varlociraptor-delly/COLO\_829-GSC.Y.bcf - varlociraptor-delly/COLO\_829-Ill.1.bcf - varlociraptor-delly/COLO\_829-Ill.2.bcf - varlociraptor-delly/COLO\_829-Ill.3.bcf - varlociraptor-delly/COLO\_829-Ill.4.bcf - varlociraptor-delly/COLO\_829-Ill.5.bcf - varlociraptor-delly/COLO\_829-Ill.6.bcf - varlociraptor-delly/COLO\_829-Ill.7.bcf - varlociraptor-delly/COLO\_829-Ill.8.bcf - varlociraptor-delly/COLO\_829-Ill.9.bcf - varlociraptor-delly/COLO\_829-Ill.10.bcf - varlociraptor-delly/COLO\_829-Ill.11.bcf - varlociraptor-delly/COLO\_829-Ill.12.bcf - varlociraptor-delly/COLO\_829-Ill.13.bcf - varlociraptor-delly/COLO\_829-Ill.14.bcf - varlociraptor-delly/COLO\_829-Ill.15.bcf - varlociraptor-delly/COLO\_829-Ill.16.bcf - varlociraptor-delly/COLO\_829-Ill.17.bcf - varlociraptor-delly/COLO\_829-Ill.18.bcf - varlociraptor-delly/COLO\_829-Ill.19.bcf - varlociraptor-delly/COLO\_829-Ill.20.bcf - varlociraptor-delly/COLO\_829-Ill.21.bcf - varlociraptor-delly/COLO\_829-Ill.22.bcf - varlociraptor-delly/COLO\_829-Ill.M.bcf - varlociraptor-delly/COLO\_829-Ill.X.bcf - varlociraptor-delly/COLO\_829-Ill.Y.bcf - varlociraptor-delly/COLO\_829-TGen.1.bcf - varlociraptor-delly/COLO\_829-TGen.2.bcf - varlociraptor-delly/COLO\_829-TGen.3.bcf - varlociraptor-delly/COLO\_829-TGen.4.bcf - varlociraptor-delly/COLO\_829-TGen.5.bcf - varlociraptor-delly/COLO\_829-TGen.6.bcf - varlociraptor-delly/COLO\_829-TGen.7.bcf - varlociraptor-delly/COLO\_829-TGen.8.bcf - varlociraptor-delly/COLO\_829-TGen.9.bcf - varlociraptor-delly/COLO\_829-TGen.10.bcf - varlociraptor-delly/COLO\_829-TGen.11.bcf - varlociraptor-delly/COLO\_829-TGen.12.bcf - varlociraptor-delly/COLO\_829-TGen.13.bcf - varlociraptor-delly/COLO\_829-TGen.14.bcf - varlociraptor-delly/COLO\_829-TGen.15.bcf - varlociraptor-delly/COLO\_829-TGen.16.bcf - varlociraptor-delly/COLO\_829-TGen.17.bcf - varlociraptor-delly/COLO\_829-TGen.18.bcf - varlociraptor-delly/COLO\_829-TGen.19.bcf - varlociraptor-delly/COLO\_829-TGen.20.bcf - varlociraptor-delly/COLO\_829-TGen.21.bcf - varlociraptor-delly/COLO\_829-TGen.22.bcf - varlociraptor-delly/COLO\_829-TGen.M.bcf - varlociraptor-delly/COLO\_829-TGen.X.bcf - varlociraptor-delly/COLO\_829-TGen.Y.bcf - varlociraptor-delly/COLO\_829-EBI.1.bcf - varlociraptor-delly/COLO\_829-EBI.2.bcf - varlociraptor-delly/COLO\_829-EBI.3.bcf - varlociraptor-delly/COLO\_829-EBI.4.bcf - varlociraptor-delly/COLO\_829-EBI.5.bcf - varlociraptor-delly/COLO\_829-EBI.6.bcf - varlociraptor-delly/COLO\_829-EBI.7.bcf - varlociraptor-delly/COLO\_829-EBI.8.bcf - varlociraptor-delly/COLO\_829-EBI.9.bcf - varlociraptor-delly/COLO\_829-EBI.10.bcf - varlociraptor-delly/COLO\_829-EBI.11.bcf - varlociraptor-delly/COLO\_829-EBI.12.bcf - varlociraptor-delly/COLO\_829-EBI.13.bcf - varlociraptor-delly/COLO\_829-EBI.14.bcf - varlociraptor-delly/COLO\_829-EBI.15.bcf - varlociraptor-delly/COLO\_829-EBI.16.bcf - varlociraptor-delly/COLO\_829-EBI.17.bcf - varlociraptor-delly/COLO\_829-EBI.18.bcf - varlociraptor-delly/COLO\_829-EBI.19.bcf - varlociraptor-delly/COLO\_829-EBI.20.bcf - varlociraptor-delly/COLO\_829-EBI.21.bcf - varlociraptor-delly/COLO\_829-EBI.22.bcf - varlociraptor-delly/COLO\_829-EBI.M.bcf - varlociraptor-delly/COLO\_829-EBI.X.bcf - varlociraptor-delly/COLO\_829-EBI.Y.bcf - varlociraptor-lancet/COLO\_829-GSC.1.bcf - varlociraptor-lancet/COLO\_829-GSC.2.bcf - varlociraptor-lancet/COLO\_829-GSC.3.bcf - varlociraptor-lancet/COLO\_829-GSC.4.bcf - varlociraptor-lancet/COLO\_829-GSC.5.bcf - varlociraptor-lancet/COLO\_829-GSC.6.bcf - varlociraptor-lancet/COLO\_829-GSC.7.bcf - varlociraptor-lancet/COLO\_829-GSC.8.bcf - varlociraptor-lancet/COLO\_829-GSC.9.bcf - varlociraptor-lancet/COLO\_829-GSC.10.bcf - varlociraptor-lancet/COLO\_829-GSC.11.bcf - varlociraptor-lancet/COLO\_829-GSC.12.bcf - varlociraptor-lancet/COLO\_829-GSC.13.bcf - varlociraptor-lancet/COLO\_829-GSC.14.bcf - varlociraptor-lancet/COLO\_829-GSC.15.bcf - varlociraptor-lancet/COLO\_829-GSC.16.bcf - varlociraptor-lancet/COLO\_829-GSC.17.bcf - varlociraptor-lancet/COLO\_829-GSC.18.bcf - varlociraptor-lancet/COLO\_829-GSC.19.bcf - varlociraptor-lancet/COLO\_829-GSC.20.bcf - varlociraptor-lancet/COLO\_829-GSC.21.bcf - varlociraptor-lancet/COLO\_829-GSC.22.bcf - varlociraptor-lancet/COLO\_829-GSC.M.bcf - varlociraptor-lancet/COLO\_829-GSC.X.bcf - varlociraptor-lancet/COLO\_829-GSC.Y.bcf - varlociraptor-lancet/COLO\_829-Ill.1.bcf - varlociraptor-lancet/COLO\_829-Ill.2.bcf - varlociraptor-lancet/COLO\_829-Ill.3.bcf - varlociraptor-lancet/COLO\_829-Ill.4.bcf - varlociraptor-lancet/COLO\_829-Ill.5.bcf - varlociraptor-lancet/COLO\_829-Ill.6.bcf - varlociraptor-lancet/COLO\_829-Ill.7.bcf - varlociraptor-lancet/COLO\_829-Ill.8.bcf - varlociraptor-lancet/COLO\_829-Ill.9.bcf - varlociraptor-lancet/COLO\_829-Ill.10.bcf - varlociraptor-lancet/COLO\_829-Ill.11.bcf - varlociraptor-lancet/COLO\_829-Ill.12.bcf - varlociraptor-lancet/COLO\_829-Ill.13.bcf - varlociraptor-lancet/COLO\_829-Ill.14.bcf - varlociraptor-lancet/COLO\_829-Ill.15.bcf - varlociraptor-lancet/COLO\_829-Ill.16.bcf - varlociraptor-lancet/COLO\_829-Ill.17.bcf - varlociraptor-lancet/COLO\_829-Ill.18.bcf - varlociraptor-lancet/COLO\_829-Ill.19.bcf - varlociraptor-lancet/COLO\_829-Ill.20.bcf - varlociraptor-lancet/COLO\_829-Ill.21.bcf - varlociraptor-lancet/COLO\_829-Ill.22.bcf - varlociraptor-lancet/COLO\_829-Ill.M.bcf - varlociraptor-lancet/COLO\_829-Ill.X.bcf - varlociraptor-lancet/COLO\_829-Ill.Y.bcf - varlociraptor-lancet/COLO\_829-TGen.1.bcf - varlociraptor-lancet/COLO\_829-TGen.2.bcf - varlociraptor-lancet/COLO\_829-TGen.3.bcf - varlociraptor-lancet/COLO\_829-TGen.4.bcf - varlociraptor-lancet/COLO\_829-TGen.5.bcf - varlociraptor-lancet/COLO\_829-TGen.6.bcf - varlociraptor-lancet/COLO\_829-TGen.7.bcf - varlociraptor-lancet/COLO\_829-TGen.8.bcf - varlociraptor-lancet/COLO\_829-TGen.9.bcf - varlociraptor-lancet/COLO\_829-TGen.10.bcf - varlociraptor-lancet/COLO\_829-TGen.11.bcf - varlociraptor-lancet/COLO\_829-TGen.12.bcf - varlociraptor-lancet/COLO\_829-TGen.13.bcf - varlociraptor-lancet/COLO\_829-TGen.14.bcf - varlociraptor-lancet/COLO\_829-TGen.15.bcf - varlociraptor-lancet/COLO\_829-TGen.16.bcf - varlociraptor-lancet/COLO\_829-TGen.17.bcf - varlociraptor-lancet/COLO\_829-TGen.18.bcf - varlociraptor-lancet/COLO\_829-TGen.19.bcf - varlociraptor-lancet/COLO\_829-TGen.20.bcf - varlociraptor-lancet/COLO\_829-TGen.21.bcf - varlociraptor-lancet/COLO\_829-TGen.22.bcf - varlociraptor-lancet/COLO\_829-TGen.M.bcf - varlociraptor-lancet/COLO\_829-TGen.X.bcf - varlociraptor-lancet/COLO\_829-TGen.Y.bcf - varlociraptor-lancet/COLO\_829-EBI.1.bcf - varlociraptor-lancet/COLO\_829-EBI.2.bcf - varlociraptor-lancet/COLO\_829-EBI.3.bcf - varlociraptor-lancet/COLO\_829-EBI.4.bcf - varlociraptor-lancet/COLO\_829-EBI.5.bcf - varlociraptor-lancet/COLO\_829-EBI.6.bcf - varlociraptor-lancet/COLO\_829-EBI.7.bcf - varlociraptor-lancet/COLO\_829-EBI.8.bcf - varlociraptor-lancet/COLO\_829-EBI.9.bcf - varlociraptor-lancet/COLO\_829-EBI.10.bcf - varlociraptor-lancet/COLO\_829-EBI.11.bcf - varlociraptor-lancet/COLO\_829-EBI.12.bcf - varlociraptor-lancet/COLO\_829-EBI.13.bcf - varlociraptor-lancet/COLO\_829-EBI.14.bcf - varlociraptor-lancet/COLO\_829-EBI.15.bcf - varlociraptor-lancet/COLO\_829-EBI.16.bcf - varlociraptor-lancet/COLO\_829-EBI.17.bcf - varlociraptor-lancet/COLO\_829-EBI.18.bcf - varlociraptor-lancet/COLO\_829-EBI.19.bcf - varlociraptor-lancet/COLO\_829-EBI.20.bcf - varlociraptor-lancet/COLO\_829-EBI.21.bcf - varlociraptor-lancet/COLO\_829-EBI.22.bcf - varlociraptor-lancet/COLO\_829-EBI.M.bcf - varlociraptor-lancet/COLO\_829-EBI.X.bcf - varlociraptor-lancet/COLO\_829-EBI.Y.bcf - varlociraptor-manta/COLO\_829-GSC.1.bcf - varlociraptor-manta/COLO\_829-GSC.2.bcf - varlociraptor-manta/COLO\_829-GSC.3.bcf - varlociraptor-manta/COLO\_829-GSC.4.bcf - varlociraptor-manta/COLO\_829-GSC.5.bcf - varlociraptor-manta/COLO\_829-GSC.6.bcf - varlociraptor-manta/COLO\_829-GSC.7.bcf - varlociraptor-manta/COLO\_829-GSC.8.bcf - varlociraptor-manta/COLO\_829-GSC.9.bcf - varlociraptor-manta/COLO\_829-GSC.10.bcf - varlociraptor-manta/COLO\_829-GSC.11.bcf - varlociraptor-manta/COLO\_829-GSC.12.bcf - varlociraptor-manta/COLO\_829-GSC.13.bcf - varlociraptor-manta/COLO\_829-GSC.14.bcf - varlociraptor-manta/COLO\_829-GSC.15.bcf - varlociraptor-manta/COLO\_829-GSC.16.bcf - varlociraptor-manta/COLO\_829-GSC.17.bcf - varlociraptor-manta/COLO\_829-GSC.18.bcf - varlociraptor-manta/COLO\_829-GSC.19.bcf - varlociraptor-manta/COLO\_829-GSC.20.bcf - varlociraptor-manta/COLO\_829-GSC.21.bcf - varlociraptor-manta/COLO\_829-GSC.22.bcf - varlociraptor-manta/COLO\_829-GSC.M.bcf - varlociraptor-manta/COLO\_829-GSC.X.bcf - varlociraptor-manta/COLO\_829-GSC.Y.bcf - varlociraptor-manta/COLO\_829-Ill.1.bcf - varlociraptor-manta/COLO\_829-Ill.2.bcf - varlociraptor-manta/COLO\_829-Ill.3.bcf - varlociraptor-manta/COLO\_829-Ill.4.bcf - varlociraptor-manta/COLO\_829-Ill.5.bcf - varlociraptor-manta/COLO\_829-Ill.6.bcf - varlociraptor-manta/COLO\_829-Ill.7.bcf - varlociraptor-manta/COLO\_829-Ill.8.bcf - varlociraptor-manta/COLO\_829-Ill.9.bcf - varlociraptor-manta/COLO\_829-Ill.10.bcf - varlociraptor-manta/COLO\_829-Ill.11.bcf - varlociraptor-manta/COLO\_829-Ill.12.bcf - varlociraptor-manta/COLO\_829-Ill.13.bcf - varlociraptor-manta/COLO\_829-Ill.14.bcf - varlociraptor-manta/COLO\_829-Ill.15.bcf - varlociraptor-manta/COLO\_829-Ill.16.bcf - varlociraptor-manta/COLO\_829-Ill.17.bcf - varlociraptor-manta/COLO\_829-Ill.18.bcf - varlociraptor-manta/COLO\_829-Ill.19.bcf - varlociraptor-manta/COLO\_829-Ill.20.bcf - varlociraptor-manta/COLO\_829-Ill.21.bcf - varlociraptor-manta/COLO\_829-Ill.22.bcf - varlociraptor-manta/COLO\_829-Ill.M.bcf - varlociraptor-manta/COLO\_829-Ill.X.bcf - varlociraptor-manta/COLO\_829-Ill.Y.bcf - varlociraptor-manta/COLO\_829-TGen.1.bcf - varlociraptor-manta/COLO\_829-TGen.2.bcf - varlociraptor-manta/COLO\_829-TGen.3.bcf - varlociraptor-manta/COLO\_829-TGen.4.bcf - varlociraptor-manta/COLO\_829-TGen.5.bcf - varlociraptor-manta/COLO\_829-TGen.6.bcf - varlociraptor-manta/COLO\_829-TGen.7.bcf - varlociraptor-manta/COLO\_829-TGen.8.bcf - varlociraptor-manta/COLO\_829-TGen.9.bcf - varlociraptor-manta/COLO\_829-TGen.10.bcf - varlociraptor-manta/COLO\_829-TGen.11.bcf - varlociraptor-manta/COLO\_829-TGen.12.bcf - varlociraptor-manta/COLO\_829-TGen.13.bcf - varlociraptor-manta/COLO\_829-TGen.14.bcf - varlociraptor-manta/COLO\_829-TGen.15.bcf - varlociraptor-manta/COLO\_829-TGen.16.bcf - varlociraptor-manta/COLO\_829-TGen.17.bcf - varlociraptor-manta/COLO\_829-TGen.18.bcf - varlociraptor-manta/COLO\_829-TGen.19.bcf - varlociraptor-manta/COLO\_829-TGen.20.bcf - varlociraptor-manta/COLO\_829-TGen.21.bcf - varlociraptor-manta/COLO\_829-TGen.22.bcf - varlociraptor-manta/COLO\_829-TGen.M.bcf - varlociraptor-manta/COLO\_829-TGen.X.bcf - varlociraptor-manta/COLO\_829-TGen.Y.bcf - varlociraptor-manta/COLO\_829-EBI.1.bcf - varlociraptor-manta/COLO\_829-EBI.2.bcf - varlociraptor-manta/COLO\_829-EBI.3.bcf - varlociraptor-manta/COLO\_829-EBI.4.bcf - varlociraptor-manta/COLO\_829-EBI.5.bcf - varlociraptor-manta/COLO\_829-EBI.6.bcf - varlociraptor-manta/COLO\_829-EBI.7.bcf - varlociraptor-manta/COLO\_829-EBI.8.bcf - varlociraptor-manta/COLO\_829-EBI.9.bcf - varlociraptor-manta/COLO\_829-EBI.10.bcf - varlociraptor-manta/COLO\_829-EBI.11.bcf - varlociraptor-manta/COLO\_829-EBI.12.bcf - varlociraptor-manta/COLO\_829-EBI.13.bcf - varlociraptor-manta/COLO\_829-EBI.14.bcf - varlociraptor-manta/COLO\_829-EBI.15.bcf - varlociraptor-manta/COLO\_829-EBI.16.bcf - varlociraptor-manta/COLO\_829-EBI.17.bcf - varlociraptor-manta/COLO\_829-EBI.18.bcf - varlociraptor-manta/COLO\_829-EBI.19.bcf - varlociraptor-manta/COLO\_829-EBI.20.bcf - varlociraptor-manta/COLO\_829-EBI.21.bcf - varlociraptor-manta/COLO\_829-EBI.22.bcf - varlociraptor-manta/COLO\_829-EBI.M.bcf - varlociraptor-manta/COLO\_829-EBI.X.bcf - varlociraptor-manta/COLO\_829-EBI.Y.bcf - varlociraptor-strelka/COLO\_829-GSC.1.bcf - varlociraptor-strelka/COLO\_829-GSC.2.bcf - varlociraptor-strelka/COLO\_829-GSC.3.bcf - varlociraptor-strelka/COLO\_829-GSC.4.bcf - varlociraptor-strelka/COLO\_829-GSC.5.bcf - varlociraptor-strelka/COLO\_829-GSC.6.bcf - varlociraptor-strelka/COLO\_829-GSC.7.bcf - varlociraptor-strelka/COLO\_829-GSC.8.bcf - varlociraptor-strelka/COLO\_829-GSC.9.bcf - varlociraptor-strelka/COLO\_829-GSC.10.bcf - varlociraptor-strelka/COLO\_829-GSC.11.bcf - varlociraptor-strelka/COLO\_829-GSC.12.bcf - varlociraptor-strelka/COLO\_829-GSC.13.bcf - varlociraptor-strelka/COLO\_829-GSC.14.bcf - varlociraptor-strelka/COLO\_829-GSC.15.bcf - varlociraptor-strelka/COLO\_829-GSC.16.bcf - varlociraptor-strelka/COLO\_829-GSC.17.bcf - varlociraptor-strelka/COLO\_829-GSC.18.bcf - varlociraptor-strelka/COLO\_829-GSC.19.bcf - varlociraptor-strelka/COLO\_829-GSC.20.bcf - varlociraptor-strelka/COLO\_829-GSC.21.bcf - varlociraptor-strelka/COLO\_829-GSC.22.bcf - varlociraptor-strelka/COLO\_829-GSC.M.bcf - varlociraptor-strelka/COLO\_829-GSC.X.bcf - varlociraptor-strelka/COLO\_829-GSC.Y.bcf - varlociraptor-strelka/COLO\_829-Ill.1.bcf - varlociraptor-strelka/COLO\_829-Ill.2.bcf - varlociraptor-strelka/COLO\_829-Ill.3.bcf - varlociraptor-strelka/COLO\_829-Ill.4.bcf - varlociraptor-strelka/COLO\_829-Ill.5.bcf - varlociraptor-strelka/COLO\_829-Ill.6.bcf - varlociraptor-strelka/COLO\_829-Ill.7.bcf - varlociraptor-strelka/COLO\_829-Ill.8.bcf - varlociraptor-strelka/COLO\_829-Ill.9.bcf - varlociraptor-strelka/COLO\_829-Ill.10.bcf - varlociraptor-strelka/COLO\_829-Ill.11.bcf - varlociraptor-strelka/COLO\_829-Ill.12.bcf - varlociraptor-strelka/COLO\_829-Ill.13.bcf - varlociraptor-strelka/COLO\_829-Ill.14.bcf - varlociraptor-strelka/COLO\_829-Ill.15.bcf - varlociraptor-strelka/COLO\_829-Ill.16.bcf - varlociraptor-strelka/COLO\_829-Ill.17.bcf - varlociraptor-strelka/COLO\_829-Ill.18.bcf - varlociraptor-strelka/COLO\_829-Ill.19.bcf - varlociraptor-strelka/COLO\_829-Ill.20.bcf - varlociraptor-strelka/COLO\_829-Ill.21.bcf - varlociraptor-strelka/COLO\_829-Ill.22.bcf - varlociraptor-strelka/COLO\_829-Ill.M.bcf - varlociraptor-strelka/COLO\_829-Ill.X.bcf - varlociraptor-strelka/COLO\_829-Ill.Y.bcf - varlociraptor-strelka/COLO\_829-TGen.1.bcf - varlociraptor-strelka/COLO\_829-TGen.2.bcf - varlociraptor-strelka/COLO\_829-TGen.3.bcf - varlociraptor-strelka/COLO\_829-TGen.4.bcf - varlociraptor-strelka/COLO\_829-TGen.5.bcf - varlociraptor-strelka/COLO\_829-TGen.6.bcf - varlociraptor-strelka/COLO\_829-TGen.7.bcf - varlociraptor-strelka/COLO\_829-TGen.8.bcf - varlociraptor-strelka/COLO\_829-TGen.9.bcf - varlociraptor-strelka/COLO\_829-TGen.10.bcf - varlociraptor-strelka/COLO\_829-TGen.11.bcf - varlociraptor-strelka/COLO\_829-TGen.12.bcf - varlociraptor-strelka/COLO\_829-TGen.13.bcf - varlociraptor-strelka/COLO\_829-TGen.14.bcf - varlociraptor-strelka/COLO\_829-TGen.15.bcf - varlociraptor-strelka/COLO\_829-TGen.16.bcf - varlociraptor-strelka/COLO\_829-TGen.17.bcf - varlociraptor-strelka/COLO\_829-TGen.18.bcf - varlociraptor-strelka/COLO\_829-TGen.19.bcf - varlociraptor-strelka/COLO\_829-TGen.20.bcf - varlociraptor-strelka/COLO\_829-TGen.21.bcf - varlociraptor-strelka/COLO\_829-TGen.22.bcf - varlociraptor-strelka/COLO\_829-TGen.M.bcf - varlociraptor-strelka/COLO\_829-TGen.X.bcf - varlociraptor-strelka/COLO\_829-TGen.Y.bcf - varlociraptor-strelka/COLO\_829-EBI.1.bcf - varlociraptor-strelka/COLO\_829-EBI.2.bcf - varlociraptor-strelka/COLO\_829-EBI.3.bcf - varlociraptor-strelka/COLO\_829-EBI.4.bcf - varlociraptor-strelka/COLO\_829-EBI.5.bcf - varlociraptor-strelka/COLO\_829-EBI.6.bcf - varlociraptor-strelka/COLO\_829-EBI.7.bcf - varlociraptor-strelka/COLO\_829-EBI.8.bcf - varlociraptor-strelka/COLO\_829-EBI.9.bcf - varlociraptor-strelka/COLO\_829-EBI.10.bcf - varlociraptor-strelka/COLO\_829-EBI.11.bcf - varlociraptor-strelka/COLO\_829-EBI.12.bcf - varlociraptor-strelka/COLO\_829-EBI.13.bcf - varlociraptor-strelka/COLO\_829-EBI.14.bcf - varlociraptor-strelka/COLO\_829-EBI.15.bcf - varlociraptor-strelka/COLO\_829-EBI.16.bcf - varlociraptor-strelka/COLO\_829-EBI.17.bcf - varlociraptor-strelka/COLO\_829-EBI.18.bcf - varlociraptor-strelka/COLO\_829-EBI.19.bcf - varlociraptor-strelka/COLO\_829-EBI.20.bcf - varlociraptor-strelka/COLO\_829-EBI.21.bcf - varlociraptor-strelka/COLO\_829-EBI.22.bcf - varlociraptor-strelka/COLO\_829-EBI.M.bcf - varlociraptor-strelka/COLO\_829-EBI.X.bcf - varlociraptor-strelka/COLO\_829-EBI.Y.bcf - varlociraptor-bpi/COLO\_829-GSC.1.bcf - varlociraptor-bpi/COLO\_829-GSC.2.bcf - varlociraptor-bpi/COLO\_829-GSC.3.bcf - varlociraptor-bpi/COLO\_829-GSC.4.bcf - varlociraptor-bpi/COLO\_829-GSC.5.bcf - varlociraptor-bpi/COLO\_829-GSC.6.bcf - varlociraptor-bpi/COLO\_829-GSC.7.bcf - varlociraptor-bpi/COLO\_829-GSC.8.bcf - varlociraptor-bpi/COLO\_829-GSC.9.bcf - varlociraptor-bpi/COLO\_829-GSC.10.bcf - varlociraptor-bpi/COLO\_829-GSC.11.bcf - varlociraptor-bpi/COLO\_829-GSC.12.bcf - varlociraptor-bpi/COLO\_829-GSC.13.bcf - varlociraptor-bpi/COLO\_829-GSC.14.bcf - varlociraptor-bpi/COLO\_829-GSC.15.bcf - varlociraptor-bpi/COLO\_829-GSC.16.bcf - varlociraptor-bpi/COLO\_829-GSC.17.bcf - varlociraptor-bpi/COLO\_829-GSC.18.bcf - varlociraptor-bpi/COLO\_829-GSC.19.bcf - varlociraptor-bpi/COLO\_829-GSC.20.bcf - varlociraptor-bpi/COLO\_829-GSC.21.bcf - varlociraptor-bpi/COLO\_829-GSC.22.bcf - varlociraptor-bpi/COLO\_829-GSC.M.bcf - varlociraptor-bpi/COLO\_829-GSC.X.bcf - varlociraptor-bpi/COLO\_829-GSC.Y.bcf - varlociraptor-bpi/COLO\_829-Ill.1.bcf - varlociraptor-bpi/COLO\_829-Ill.2.bcf - varlociraptor-bpi/COLO\_829-Ill.3.bcf - varlociraptor-bpi/COLO\_829-Ill.4.bcf - varlociraptor-bpi/COLO\_829-Ill.5.bcf - varlociraptor-bpi/COLO\_829-Ill.6.bcf - varlociraptor-bpi/COLO\_829-Ill.7.bcf - varlociraptor-bpi/COLO\_829-Ill.8.bcf - varlociraptor-bpi/COLO\_829-Ill.9.bcf - varlociraptor-bpi/COLO\_829-Ill.10.bcf - varlociraptor-bpi/COLO\_829-Ill.11.bcf - varlociraptor-bpi/COLO\_829-Ill.12.bcf - varlociraptor-bpi/COLO\_829-Ill.13.bcf - varlociraptor-bpi/COLO\_829-Ill.14.bcf - varlociraptor-bpi/COLO\_829-Ill.15.bcf - varlociraptor-bpi/COLO\_829-Ill.16.bcf - varlociraptor-bpi/COLO\_829-Ill.17.bcf - varlociraptor-bpi/COLO\_829-Ill.18.bcf - varlociraptor-bpi/COLO\_829-Ill.19.bcf - varlociraptor-bpi/COLO\_829-Ill.20.bcf - varlociraptor-bpi/COLO\_829-Ill.21.bcf - varlociraptor-bpi/COLO\_829-Ill.22.bcf - varlociraptor-bpi/COLO\_829-Ill.M.bcf - varlociraptor-bpi/COLO\_829-Ill.X.bcf - varlociraptor-bpi/COLO\_829-Ill.Y.bcf - varlociraptor-bpi/COLO\_829-TGen.1.bcf - varlociraptor-bpi/COLO\_829-TGen.2.bcf - varlociraptor-bpi/COLO\_829-TGen.3.bcf - varlociraptor-bpi/COLO\_829-TGen.4.bcf - varlociraptor-bpi/COLO\_829-TGen.5.bcf - varlociraptor-bpi/COLO\_829-TGen.6.bcf - varlociraptor-bpi/COLO\_829-TGen.7.bcf - varlociraptor-bpi/COLO\_829-TGen.8.bcf - varlociraptor-bpi/COLO\_829-TGen.9.bcf - varlociraptor-bpi/COLO\_829-TGen.10.bcf - varlociraptor-bpi/COLO\_829-TGen.11.bcf - varlociraptor-bpi/COLO\_829-TGen.12.bcf - varlociraptor-bpi/COLO\_829-TGen.13.bcf - varlociraptor-bpi/COLO\_829-TGen.14.bcf - varlociraptor-bpi/COLO\_829-TGen.15.bcf - varlociraptor-bpi/COLO\_829-TGen.16.bcf - varlociraptor-bpi/COLO\_829-TGen.17.bcf - varlociraptor-bpi/COLO\_829-TGen.18.bcf - varlociraptor-bpi/COLO\_829-TGen.19.bcf - varlociraptor-bpi/COLO\_829-TGen.20.bcf - varlociraptor-bpi/COLO\_829-TGen.21.bcf - varlociraptor-bpi/COLO\_829-TGen.22.bcf - varlociraptor-bpi/COLO\_829-TGen.M.bcf - varlociraptor-bpi/COLO\_829-TGen.X.bcf - varlociraptor-bpi/COLO\_829-TGen.Y.bcf - varlociraptor-bpi/COLO\_829-EBI.1.bcf - varlociraptor-bpi/COLO\_829-EBI.2.bcf - varlociraptor-bpi/COLO\_829-EBI.3.bcf - varlociraptor-bpi/COLO\_829-EBI.4.bcf - varlociraptor-bpi/COLO\_829-EBI.5.bcf - varlociraptor-bpi/COLO\_829-EBI.6.bcf - varlociraptor-bpi/COLO\_829-EBI.7.bcf - varlociraptor-bpi/COLO\_829-EBI.8.bcf - varlociraptor-bpi/COLO\_829-EBI.9.bcf - varlociraptor-bpi/COLO\_829-EBI.10.bcf - varlociraptor-bpi/COLO\_829-EBI.11.bcf - varlociraptor-bpi/COLO\_829-EBI.12.bcf - varlociraptor-bpi/COLO\_829-EBI.13.bcf - varlociraptor-bpi/COLO\_829-EBI.14.bcf - varlociraptor-bpi/COLO\_829-EBI.15.bcf - varlociraptor-bpi/COLO\_829-EBI.16.bcf - varlociraptor-bpi/COLO\_829-EBI.17.bcf - varlociraptor-bpi/COLO\_829-EBI.18.bcf - varlociraptor-bpi/COLO\_829-EBI.19.bcf - varlociraptor-bpi/COLO\_829-EBI.20.bcf - varlociraptor-bpi/COLO\_829-EBI.21.bcf - varlociraptor-bpi/COLO\_829-EBI.22.bcf - varlociraptor-bpi/COLO\_829-EBI.M.bcf - varlociraptor-bpi/COLO\_829-EBI.X.bcf - varlociraptor-bpi/COLO\_829-EBI.Y.bcf - varlociraptor-delly/simulated-bwa.1.bcf - varlociraptor-delly/simulated-bwa.2.bcf - varlociraptor-delly/simulated-bwa.3.bcf - varlociraptor-delly/simulated-bwa.4.bcf - varlociraptor-delly/simulated-bwa.5.bcf - varlociraptor-delly/simulated-bwa.6.bcf - varlociraptor-delly/simulated-bwa.7.bcf - varlociraptor-delly/simulated-bwa.8.bcf - varlociraptor-delly/simulated-bwa.9.bcf - varlociraptor-delly/simulated-bwa.10.bcf - varlociraptor-delly/simulated-bwa.11.bcf - varlociraptor-delly/simulated-bwa.12.bcf - varlociraptor-delly/simulated-bwa.13.bcf - varlociraptor-delly/simulated-bwa.14.bcf - varlociraptor-delly/simulated-bwa.15.bcf - varlociraptor-delly/simulated-bwa.16.bcf - varlociraptor-delly/simulated-bwa.17.bcf - varlociraptor-delly/simulated-bwa.18.bcf - varlociraptor-delly/simulated-bwa.19.bcf - varlociraptor-delly/simulated-bwa.20.bcf - varlociraptor-delly/simulated-bwa.21.bcf - varlociraptor-delly/simulated-bwa.22.bcf - varlociraptor-delly/simulated-bwa.M.bcf - varlociraptor-delly/simulated-bwa.X.bcf - varlociraptor-delly/simulated-bwa.Y.bcf - varlociraptor-lancet/simulated-bwa.1.bcf - varlociraptor-lancet/simulated-bwa.2.bcf - varlociraptor-lancet/simulated-bwa.3.bcf - varlociraptor-lancet/simulated-bwa.4.bcf - varlociraptor-lancet/simulated-bwa.5.bcf - varlociraptor-lancet/simulated-bwa.6.bcf - varlociraptor-lancet/simulated-bwa.7.bcf - varlociraptor-lancet/simulated-bwa.8.bcf - varlociraptor-lancet/simulated-bwa.9.bcf - varlociraptor-lancet/simulated-bwa.10.bcf - varlociraptor-lancet/simulated-bwa.11.bcf - varlociraptor-lancet/simulated-bwa.12.bcf - varlociraptor-lancet/simulated-bwa.13.bcf - varlociraptor-lancet/simulated-bwa.14.bcf - varlociraptor-lancet/simulated-bwa.15.bcf - varlociraptor-lancet/simulated-bwa.16.bcf - varlociraptor-lancet/simulated-bwa.17.bcf - varlociraptor-lancet/simulated-bwa.18.bcf - varlociraptor-lancet/simulated-bwa.19.bcf - varlociraptor-lancet/simulated-bwa.20.bcf - varlociraptor-lancet/simulated-bwa.21.bcf - varlociraptor-lancet/simulated-bwa.22.bcf - varlociraptor-lancet/simulated-bwa.M.bcf - varlociraptor-lancet/simulated-bwa.X.bcf - varlociraptor-lancet/simulated-bwa.Y.bcf - varlociraptor-manta/simulated-bwa.1.bcf - varlociraptor-manta/simulated-bwa.2.bcf - varlociraptor-manta/simulated-bwa.3.bcf - varlociraptor-manta/simulated-bwa.4.bcf - varlociraptor-manta/simulated-bwa.5.bcf - varlociraptor-manta/simulated-bwa.6.bcf - varlociraptor-manta/simulated-bwa.7.bcf - varlociraptor-manta/simulated-bwa.8.bcf - varlociraptor-manta/simulated-bwa.9.bcf - varlociraptor-manta/simulated-bwa.10.bcf - varlociraptor-manta/simulated-bwa.11.bcf - varlociraptor-manta/simulated-bwa.12.bcf - varlociraptor-manta/simulated-bwa.13.bcf - varlociraptor-manta/simulated-bwa.14.bcf - varlociraptor-manta/simulated-bwa.15.bcf - varlociraptor-manta/simulated-bwa.16.bcf - varlociraptor-manta/simulated-bwa.17.bcf - varlociraptor-manta/simulated-bwa.18.bcf - varlociraptor-manta/simulated-bwa.19.bcf - varlociraptor-manta/simulated-bwa.20.bcf - varlociraptor-manta/simulated-bwa.21.bcf - varlociraptor-manta/simulated-bwa.22.bcf - varlociraptor-manta/simulated-bwa.M.bcf - varlociraptor-manta/simulated-bwa.X.bcf - varlociraptor-manta/simulated-bwa.Y.bcf - varlociraptor-strelka/simulated-bwa.1.bcf - varlociraptor-strelka/simulated-bwa.2.bcf - varlociraptor-strelka/simulated-bwa.3.bcf - varlociraptor-strelka/simulated-bwa.4.bcf - varlociraptor-strelka/simulated-bwa.5.bcf - varlociraptor-strelka/simulated-bwa.6.bcf - varlociraptor-strelka/simulated-bwa.7.bcf - varlociraptor-strelka/simulated-bwa.8.bcf - varlociraptor-strelka/simulated-bwa.9.bcf - varlociraptor-strelka/simulated-bwa.10.bcf - varlociraptor-strelka/simulated-bwa.11.bcf - varlociraptor-strelka/simulated-bwa.12.bcf - varlociraptor-strelka/simulated-bwa.13.bcf - varlociraptor-strelka/simulated-bwa.14.bcf - varlociraptor-strelka/simulated-bwa.15.bcf - varlociraptor-strelka/simulated-bwa.16.bcf - varlociraptor-strelka/simulated-bwa.17.bcf - varlociraptor-strelka/simulated-bwa.18.bcf - varlociraptor-strelka/simulated-bwa.19.bcf - varlociraptor-strelka/simulated-bwa.20.bcf - varlociraptor-strelka/simulated-bwa.21.bcf - varlociraptor-strelka/simulated-bwa.22.bcf - varlociraptor-strelka/simulated-bwa.M.bcf - varlociraptor-strelka/simulated-bwa.X.bcf - varlociraptor-strelka/simulated-bwa.Y.bcf |  |  | |  |  | | --- | --- | | ``` 1 ``` | ``` bcftools view -Ou {input.calls} {params.chrom_prefix} | varlociraptor call variants {input.ref} {config[caller][varlociraptor][params]} {params.caller} tumor-normal {input.bams} --purity {params.purity} > {output} 2> {log} ``` | |
| get\_region\_bed | 7 | - neusomatic/simulated-bwa.region.bed - neusomatic/synthetic-5.region.bed - neusomatic/synthetic-20.region.bed - neusomatic/COLO\_829-GSC.region.bed - neusomatic/COLO\_829-Ill.region.bed - neusomatic/COLO\_829-TGen.region.bed - neusomatic/COLO\_829-EBI.region.bed |  | - pyfaidx =0.5.7 | |  |  | | --- | --- | | ``` 1 ``` | ``` faidx --transform bed {input} > {output} ``` | |
| mark\_duplicates | 12 | - mapped-bwa/synthetic-5.tumor.hg38.sorted.bam - mapped-bwa/synthetic-5.tumor.hg38.markdup.metrics.txt - mapped-bwa/synthetic-5.normal.hg38.sorted.bam - mapped-bwa/synthetic-5.normal.hg38.markdup.metrics.txt - mapped-bwa/synthetic-20.tumor.hg38.sorted.bam - mapped-bwa/synthetic-20.tumor.hg38.markdup.metrics.txt - mapped-bwa/synthetic-20.normal.hg38.sorted.bam - mapped-bwa/synthetic-20.normal.hg38.markdup.metrics.txt - mapped-bwa/COLO\_829-GSC.tumor.hg38.sorted.bam - mapped-bwa/COLO\_829-GSC.tumor.hg38.markdup.metrics.txt - mapped-bwa/COLO\_829-GSC.normal.hg38.sorted.bam - mapped-bwa/COLO\_829-GSC.normal.hg38.markdup.metrics.txt - mapped-bwa/COLO\_829-Ill.tumor.hg38.sorted.bam - mapped-bwa/COLO\_829-Ill.tumor.hg38.markdup.metrics.txt - mapped-bwa/COLO\_829-Ill.normal.hg38.sorted.bam - mapped-bwa/COLO\_829-Ill.normal.hg38.markdup.metrics.txt - mapped-bwa/COLO\_829-TGen.tumor.hg38.sorted.bam - mapped-bwa/COLO\_829-TGen.tumor.hg38.markdup.metrics.txt - mapped-bwa/COLO\_829-TGen.normal.hg38.sorted.bam - mapped-bwa/COLO\_829-TGen.normal.hg38.markdup.metrics.txt - mapped-bwa/COLO\_829-EBI.tumor.hg38.sorted.bam - mapped-bwa/COLO\_829-EBI.tumor.hg38.markdup.metrics.txt - mapped-bwa/COLO\_829-EBI.normal.hg38.sorted.bam - mapped-bwa/COLO\_829-EBI.normal.hg38.markdup.metrics.txt |  | - picard ==2.9.2 | |  |  | | --- | --- | | ```  1  2  3  4  5  6  7  8  9 10 11 12 ``` | ``` __author__ = "Johannes Köster" __copyright__ = "Copyright 2016, Johannes Köster" __email__ = "koester@jimmy.harvard.edu" __license__ = "MIT"   from snakemake.shell import shell   shell("picard MarkDuplicates {snakemake.params} INPUT={snakemake.input} "       "OUTPUT={snakemake.output.bam} METRICS_FILE={snakemake.output.metrics} "       "&> {snakemake.log}") ``` | |
| samtools\_index | 12 | - mapped-bwa/synthetic-5.tumor.hg38.sorted.bam.bai - mapped-bwa/synthetic-5.normal.hg38.sorted.bam.bai - mapped-bwa/synthetic-20.tumor.hg38.sorted.bam.bai - mapped-bwa/synthetic-20.normal.hg38.sorted.bam.bai - mapped-bwa/COLO\_829-GSC.tumor.hg38.sorted.bam.bai - mapped-bwa/COLO\_829-GSC.normal.hg38.sorted.bam.bai - mapped-bwa/COLO\_829-Ill.tumor.hg38.sorted.bam.bai - mapped-bwa/COLO\_829-Ill.normal.hg38.sorted.bam.bai - mapped-bwa/COLO\_829-TGen.tumor.hg38.sorted.bam.bai - mapped-bwa/COLO\_829-TGen.normal.hg38.sorted.bam.bai - mapped-bwa/COLO\_829-EBI.tumor.hg38.sorted.bam.bai - mapped-bwa/COLO\_829-EBI.normal.hg38.sorted.bam.bai |  | - samtools ==1.6 | |  |  | | --- | --- | | ```  1  2  3  4  5  6  7  8  9 10 ``` | ``` __author__ = "Johannes Köster" __copyright__ = "Copyright 2016, Johannes Köster" __email__ = "koester@jimmy.harvard.edu" __license__ = "MIT"   from snakemake.shell import shell   shell("samtools index {snakemake.params} {snakemake.input[0]} {snakemake.output[0]}") ``` | |
| lancet | 50 | - lancet/synthetic-5/chr1.vcf - lancet/synthetic-5/chr2.vcf - lancet/synthetic-5/chr3.vcf - lancet/synthetic-5/chr4.vcf - lancet/synthetic-5/chr5.vcf - lancet/synthetic-5/chr6.vcf - lancet/synthetic-5/chr7.vcf - lancet/synthetic-5/chr8.vcf - lancet/synthetic-5/chr9.vcf - lancet/synthetic-5/chr10.vcf - lancet/synthetic-5/chr11.vcf - lancet/synthetic-5/chr12.vcf - lancet/synthetic-5/chr13.vcf - lancet/synthetic-5/chr14.vcf - lancet/synthetic-5/chr15.vcf - lancet/synthetic-5/chr16.vcf - lancet/synthetic-5/chr17.vcf - lancet/synthetic-5/chr18.vcf - lancet/synthetic-5/chr19.vcf - lancet/synthetic-5/chr20.vcf - lancet/synthetic-5/chr21.vcf - lancet/synthetic-5/chr22.vcf - lancet/synthetic-5/chrM.vcf - lancet/synthetic-5/chrX.vcf - lancet/synthetic-5/chrY.vcf - lancet/synthetic-20/chr1.vcf - lancet/synthetic-20/chr2.vcf - lancet/synthetic-20/chr3.vcf - lancet/synthetic-20/chr4.vcf - lancet/synthetic-20/chr5.vcf - lancet/synthetic-20/chr6.vcf - lancet/synthetic-20/chr7.vcf - lancet/synthetic-20/chr8.vcf - lancet/synthetic-20/chr9.vcf - lancet/synthetic-20/chr10.vcf - lancet/synthetic-20/chr11.vcf - lancet/synthetic-20/chr12.vcf - lancet/synthetic-20/chr13.vcf - lancet/synthetic-20/chr14.vcf - lancet/synthetic-20/chr15.vcf - lancet/synthetic-20/chr16.vcf - lancet/synthetic-20/chr17.vcf - lancet/synthetic-20/chr18.vcf - lancet/synthetic-20/chr19.vcf - lancet/synthetic-20/chr20.vcf - lancet/synthetic-20/chr21.vcf - lancet/synthetic-20/chr22.vcf - lancet/synthetic-20/chrM.vcf - lancet/synthetic-20/chrX.vcf - lancet/synthetic-20/chrY.vcf |  | - gxx\_linux-64 - make - cmake - bamtools =2.5.1 - htslib =1.3 | |  |  | | --- | --- | | ``` 1 ``` | ``` LD_LIBRARY_PATH=$CONDA_PREFIX/lib resources/lancet --tumor {input.bams[0]} --normal {input.bams[1]} --ref {input.ref} --reg {params.region} --num-threads {threads} {params.extra} > {output} 2> {log} ``` | |
| lancet | 75 | - lancet/COLO\_829-GSC/chr1.vcf - lancet/COLO\_829-GSC/chr2.vcf - lancet/COLO\_829-GSC/chr3.vcf - lancet/COLO\_829-GSC/chr4.vcf - lancet/COLO\_829-GSC/chr5.vcf - lancet/COLO\_829-GSC/chr6.vcf - lancet/COLO\_829-GSC/chr7.vcf - lancet/COLO\_829-GSC/chr8.vcf - lancet/COLO\_829-GSC/chr9.vcf - lancet/COLO\_829-GSC/chr10.vcf - lancet/COLO\_829-GSC/chr11.vcf - lancet/COLO\_829-GSC/chr12.vcf - lancet/COLO\_829-GSC/chr13.vcf - lancet/COLO\_829-GSC/chr14.vcf - lancet/COLO\_829-GSC/chr15.vcf - lancet/COLO\_829-GSC/chr16.vcf - lancet/COLO\_829-GSC/chr17.vcf - lancet/COLO\_829-GSC/chr18.vcf - lancet/COLO\_829-GSC/chr19.vcf - lancet/COLO\_829-GSC/chr20.vcf - lancet/COLO\_829-GSC/chr21.vcf - lancet/COLO\_829-GSC/chr22.vcf - lancet/COLO\_829-GSC/chrM.vcf - lancet/COLO\_829-GSC/chrX.vcf - lancet/COLO\_829-GSC/chrY.vcf - lancet/COLO\_829-Ill/chr1.vcf - lancet/COLO\_829-Ill/chr2.vcf - lancet/COLO\_829-Ill/chr3.vcf - lancet/COLO\_829-Ill/chr4.vcf - lancet/COLO\_829-Ill/chr5.vcf - lancet/COLO\_829-Ill/chr6.vcf - lancet/COLO\_829-Ill/chr7.vcf - lancet/COLO\_829-Ill/chr8.vcf - lancet/COLO\_829-Ill/chr9.vcf - lancet/COLO\_829-Ill/chr10.vcf - lancet/COLO\_829-Ill/chr11.vcf - lancet/COLO\_829-Ill/chr12.vcf - lancet/COLO\_829-Ill/chr13.vcf - lancet/COLO\_829-Ill/chr14.vcf - lancet/COLO\_829-Ill/chr15.vcf - lancet/COLO\_829-Ill/chr16.vcf - lancet/COLO\_829-Ill/chr17.vcf - lancet/COLO\_829-Ill/chr18.vcf - lancet/COLO\_829-Ill/chr19.vcf - lancet/COLO\_829-Ill/chr20.vcf - lancet/COLO\_829-Ill/chr21.vcf - lancet/COLO\_829-Ill/chr22.vcf - lancet/COLO\_829-Ill/chrM.vcf - lancet/COLO\_829-Ill/chrX.vcf - lancet/COLO\_829-Ill/chrY.vcf - lancet/COLO\_829-TGen/chr1.vcf - lancet/COLO\_829-TGen/chr2.vcf - lancet/COLO\_829-TGen/chr3.vcf - lancet/COLO\_829-TGen/chr4.vcf - lancet/COLO\_829-TGen/chr5.vcf - lancet/COLO\_829-TGen/chr6.vcf - lancet/COLO\_829-TGen/chr7.vcf - lancet/COLO\_829-TGen/chr8.vcf - lancet/COLO\_829-TGen/chr9.vcf - lancet/COLO\_829-TGen/chr10.vcf - lancet/COLO\_829-TGen/chr11.vcf - lancet/COLO\_829-TGen/chr12.vcf - lancet/COLO\_829-TGen/chr13.vcf - lancet/COLO\_829-TGen/chr14.vcf - lancet/COLO\_829-TGen/chr15.vcf - lancet/COLO\_829-TGen/chr16.vcf - lancet/COLO\_829-TGen/chr17.vcf - lancet/COLO\_829-TGen/chr18.vcf - lancet/COLO\_829-TGen/chr19.vcf - lancet/COLO\_829-TGen/chr20.vcf - lancet/COLO\_829-TGen/chr21.vcf - lancet/COLO\_829-TGen/chr22.vcf - lancet/COLO\_829-TGen/chrM.vcf - lancet/COLO\_829-TGen/chrX.vcf - lancet/COLO\_829-TGen/chrY.vcf |  | - bamtools =2.3.0 | |  |  | | --- | --- | | ``` 1 ``` | ``` LD_LIBRARY_PATH=$CONDA_PREFIX/lib resources/lancet --tumor {input.bams[0]} --normal {input.bams[1]} --ref {input.ref} --reg {params.region} --num-threads {threads} {params.extra} > {output} 2> {log} ``` | |
| lancet | 25 | - lancet/COLO\_829-EBI/chr1.vcf - lancet/COLO\_829-EBI/chr2.vcf - lancet/COLO\_829-EBI/chr3.vcf - lancet/COLO\_829-EBI/chr4.vcf - lancet/COLO\_829-EBI/chr5.vcf - lancet/COLO\_829-EBI/chr6.vcf - lancet/COLO\_829-EBI/chr7.vcf - lancet/COLO\_829-EBI/chr8.vcf - lancet/COLO\_829-EBI/chr9.vcf - lancet/COLO\_829-EBI/chr10.vcf - lancet/COLO\_829-EBI/chr11.vcf - lancet/COLO\_829-EBI/chr12.vcf - lancet/COLO\_829-EBI/chr13.vcf - lancet/COLO\_829-EBI/chr14.vcf - lancet/COLO\_829-EBI/chr15.vcf - lancet/COLO\_829-EBI/chr16.vcf - lancet/COLO\_829-EBI/chr17.vcf - lancet/COLO\_829-EBI/chr18.vcf - lancet/COLO\_829-EBI/chr19.vcf - lancet/COLO\_829-EBI/chr20.vcf - lancet/COLO\_829-EBI/chr21.vcf - lancet/COLO\_829-EBI/chr22.vcf - lancet/COLO\_829-EBI/chrM.vcf - lancet/COLO\_829-EBI/chrX.vcf - lancet/COLO\_829-EBI/chrY.vcf |  |  | |  |  | | --- | --- | | ``` 1 ``` | ``` LD_LIBRARY_PATH=$CONDA_PREFIX/lib resources/lancet --tumor {input.bams[0]} --normal {input.bams[1]} --ref {input.ref} --reg {params.region} --num-threads {threads} {params.extra} > {output} 2> {log} ``` | |
| delly | 12 | - delly/synthetic-5.DEL.bcf - delly/synthetic-5.INS.bcf - delly/synthetic-20.DEL.bcf - delly/synthetic-20.INS.bcf - delly/COLO\_829-GSC.DEL.bcf - delly/COLO\_829-GSC.INS.bcf - delly/COLO\_829-Ill.DEL.bcf - delly/COLO\_829-Ill.INS.bcf - delly/COLO\_829-TGen.DEL.bcf - delly/COLO\_829-TGen.INS.bcf - delly/COLO\_829-EBI.DEL.bcf - delly/COLO\_829-EBI.INS.bcf |  | - delly ==0.7.7 | |  |  | | --- | --- | | ```  1  2  3  4  5  6  7  8  9 10 11 12 13 14 15 16 17 18 19 20 21 22 ``` | ``` __author__ = "Johannes Köster" __copyright__ = "Copyright 2016, Johannes Köster" __email__ = "koester@jimmy.harvard.edu" __license__ = "MIT"   from snakemake.shell import shell   try:     exclude = "-x " + snakemake.input.exclude except AttributeError:     exclude = ""   extra = snakemake.params.get("extra", "") log = snakemake.log_fmt_shell(stdout=True, stderr=True)  shell(     "OMP_NUM_THREADS={snakemake.threads} delly call {extra} "     "{exclude} -t {snakemake.params.vartype} -g {snakemake.input.ref} "     "-o {snakemake.output[0]} {snakemake.input.samples} {log}") ``` | |
| bpi | 6 | - bpi/synthetic-5.all.vcf - bpi/synthetic-20.all.vcf - bpi/COLO\_829-GSC.all.vcf - bpi/COLO\_829-Ill.all.vcf - bpi/COLO\_829-TGen.all.vcf - bpi/COLO\_829-EBI.all.vcf |  | - break-point-inspector =1.5 | |  |  | | --- | --- | | ``` 1 ``` | ``` (break-point-inspector -vcf {input.manta} -ref {input.samples[1]} -tumor {input.samples[0]} -output_vcf {output}) > {log} 2>&1 ``` | |
| manta\_raw | 6 | - manta/COLO\_829-GSC.all.bcf - manta/COLO\_829-Ill.all.bcf - manta/COLO\_829-TGen.all.bcf - manta/COLO\_829-EBI.all.bcf - manta/synthetic-5.all.bcf - manta/synthetic-20.all.bcf |  | - bcftools ==1.5 | |  |  | | --- | --- | | ```  1  2  3  4  5  6  7  8  9 10 11 12 ``` | ``` __author__ = "Johannes Köster" __copyright__ = "Copyright 2016, Johannes Köster" __email__ = "koester@jimmy.harvard.edu" __license__ = "MIT"   from snakemake.shell import shell   shell(     "bcftools view {snakemake.params} {snakemake.input[0]} "     "-o {snakemake.output[0]}") ``` | |
| samtools\_sort | 12 | - mapped-bwa/synthetic-5.tumor.hg38.sorted.pre.bam - mapped-bwa/synthetic-5.normal.hg38.sorted.pre.bam - mapped-bwa/synthetic-20.tumor.hg38.sorted.pre.bam - mapped-bwa/synthetic-20.normal.hg38.sorted.pre.bam - mapped-bwa/COLO\_829-GSC.tumor.hg38.sorted.pre.bam - mapped-bwa/COLO\_829-GSC.normal.hg38.sorted.pre.bam - mapped-bwa/COLO\_829-Ill.tumor.hg38.sorted.pre.bam - mapped-bwa/COLO\_829-Ill.normal.hg38.sorted.pre.bam - mapped-bwa/COLO\_829-TGen.tumor.hg38.sorted.pre.bam - mapped-bwa/COLO\_829-TGen.normal.hg38.sorted.pre.bam - mapped-bwa/COLO\_829-EBI.tumor.hg38.sorted.pre.bam - mapped-bwa/COLO\_829-EBI.normal.hg38.sorted.pre.bam |  | - samtools ==1.6 | |  |  | | --- | --- | | ```  1  2  3  4  5  6  7  8  9 10 11 12 13 14 15 ``` | ``` __author__ = "Johannes Köster" __copyright__ = "Copyright 2016, Johannes Köster" __email__ = "koester@jimmy.harvard.edu" __license__ = "MIT"   import os from snakemake.shell import shell   prefix = os.path.splitext(snakemake.output[0])[0]  shell(     "samtools sort {snakemake.params} -@ {snakemake.threads} -o {snakemake.output[0]} "     "-T {prefix} {snakemake.input[0]}") ``` | |
| bwa | 4 | - mapped-bwa/synthetic-5.tumor.hg38.bam - mapped-bwa/synthetic-5.normal.hg38.bam - mapped-bwa/synthetic-20.tumor.hg38.bam - mapped-bwa/synthetic-20.normal.hg38.bam |  | - bwa =0.7.17 - samtools =1.10 | |  |  | | --- | --- | | ``` 1 ``` | ``` (resources/bwa mem -t {threads} {params.extra} {params.index} {input.sample} | samtools view -Sb - > {output}) 2> {log} ``` | |
| bwa | 8 | - mapped-bwa/COLO\_829-GSC.tumor.hg38.bam - mapped-bwa/COLO\_829-GSC.normal.hg38.bam - mapped-bwa/COLO\_829-Ill.tumor.hg38.bam - mapped-bwa/COLO\_829-Ill.normal.hg38.bam - mapped-bwa/COLO\_829-TGen.tumor.hg38.bam - mapped-bwa/COLO\_829-TGen.normal.hg38.bam - mapped-bwa/COLO\_829-EBI.tumor.hg38.bam - mapped-bwa/COLO\_829-EBI.normal.hg38.bam |  | - python - samtools =1.6 - numpy - scikit-learn - pandas - setuptools | |  |  | | --- | --- | | ``` 1 ``` | ``` (resources/bwa mem -t {threads} {params.extra} {params.index} {input.sample} | samtools view -Sb - > {output}) 2> {log} ``` | |
