# Supplementary material for: Varlociraptor: enhancing sensitivity and controlling false discovery rate in somatic indel discovery
Source: Genome Biol. 2020 Apr 28;21:98. doi: 10.1186/s13059-020-01993-6 (PMC7187499; doi:10.1186/s13059-020-01993-6)
Supplement: Supplementary file 3 — Additional file 3 Review History. [file 13059_2020_1993_MOESM3_ESM.docx]

Review History

**First round of review**

**Reviewer 1**

**Comments to author:**

The Koster et al. present a novel model Varlociraptor for improving our ability for predicting the indels in cancer tumors. It models the intrinsic issues of short read mapping to calculate the FDR for somatic indels predicted. This is a very important problem and in practice can have a great impact in studying indels in tumor. I enjoyed the authors approaches in solving the problem and I think this paper would be of high interest and impact. I have several comments about this paper as stated below: 

1. Methods: Going over the method it seems to me that the probabilities calculated in equations (2), (3), (4) can be extended to consider the potential of multiple mapping of the reads (i.e., when one read is supporting more than one of the indels). Currently, it seems the authors only consider the aligner uncertainty as \pi_i as calculated by MAPQ score. Can the authors please discuss this issue in the paper more clearly? 

2. Methods: Can the proposed approach also work for STR extensions? As it was shown a very large fraction of the indels are in reality STR extensions. However, STR extension have their own issues for prediction such as PCR stutter errors (Willems et al.). Can the method be extended to consider these issues too?  

3. Simulations: The results provided in the simulation are quite interesting. However, the authors can try another set of simulations which they create an artificial somatic and tumor WGS by sampling from two genome with known indels predicted using long read (for example CHM1 and CHM13 haploid samples). The authors can consider the WGS from CHM1 is the normal samples and the WGS from tumor is CHM1 and some fraction of reads from CHM13 (the fraction of reads used will be the VAF). Then the somatic variants are indels predicted in CHM13 samples.  

4. Real data: The authors need to compare to some of the tools developed specifically somatic indel discovery. Please also compare the results against these three tools if possible SmuRF, NeuSomatic and novoBreak.

**Reviewer 2**

**Comments to author:**

The authors introduce a new method for detecting structural variants in the case of samples taken from tumors etc, where we can expect the aberrations are in hard-to-control allele frequencies due to tumor heterogeneity, and the reference data is from the same individual as the  tumor. The method is statistically sound and great effort is made to manage sources of noice and have control the error rates. I think the method is exemplary in providing detail: it made me feel that I would be able to implement the method myself (with extensive effort, but without ambiguity).

I am overall very happy with the manuscript. It was an interesting read and a solid contribution to the field. I have a lot of comments on the actual text, but that should be easy to fix.

I believe this paper presents a significant step forward for somatic variant discovery.

Major comments

I think the introduction is not introducing the field properly. There is a lot of work on methods for recognising structural variants, but the field is quite summarily described. I realise there is less work on somatic variants, but even so, a reader deserves a little more guidance to the present literature.

Theorem 2.2 is listed in section 2.2 without all details needed, which is mentioned below: "a list of extra conditions for this theorem necessary to hold" is referred to in the Appendix. I don't this is OK. A theorem should stand by itself, not have hidden conditions. I realise the conditions are technical and boring, but they need to be codified somehow and made explicit in the theorem.

Figure 6 seems to indicate that variant allele frequency is harder to predict for short indels. Is this true? It is surprising because it goes against some of premises of the "twilight zone" being for indels larger than 30 bp.

I think Section 4 Declarations should have clear instructions on how to find the software, even if the link is in the abstract. In principle a section like this should support automatic parsing of download instructions.

Minor issues

Language and style issues in the introduction:
* First sentence, "Cancer is a genetic disorder in the first place:", sounds strange to me. Don't you mean "Cancer is primarily a genetic disorder:"? It is very unfortunate if the very first sentence causes friction for even a fraction of readers.
* "The fraction of somatic variants discovered… has left room for improvement…" should probably be something like "Given the fraction of somatic variants discovered, there is room for improvement…"
* "Thereby, somatic insertions…"  The word "thereby" does not belong here.
* "in other contexts", with references to Mandoiu and Zelikovsky, and others: what other contexts?
* "In this paper, we suggest such a sensitive strategy, and prove that with it we can make substantial progress in terms of eliminating the somatic indel discovery blind spot." -> "In this paper, we suggest such a sensitive strategy, and prove that it substantially reduces the somatic indel discovery blind spot."
* The sentence "In the second step—which is unique to somatic variant discovery— one runs a differential analysis that classifies putative variants into somatic, because they only appear in the cancer genome, germline and healthy somatic, because variants appear in the control genome (where healthy somatic appear at subclonal levels), or just noise." is difficult to parse and should be rewritten.
* Lines 43—50 on page 1: repeated use of "dealing" and "deal"
* What is referred to with "generic settings" on line 48/49?
* Page 2, first paragraph: "The purpose of this paper is to do this: we would like to fathom the corresponding limits theoretically and to push them considerably in practice." I suggest: "The purpose of this paper is to understand theoretical limits of somatic variant discovery and improve practical limits substantially." At any rate, the word "fathom" is awkard here.
* I think the paragraph starting with "Key to success" can be reduced close to 50% without loss of meaning and with improved readability.
* "Nowadays, $n\le 60$ is not uncommon." I think "Nowadays, $n$ around 60 is not uncommon." is better describing what you mean.

Later sections:
* "We will use the indels that are output by the competitor" — which competitor is that, please be specific.
* You reference Sarek in footnote 9, but Sarek seems to have a DOI one can cite as well.
* "to computing (1)…"  should probably be "to compute (1)" or "for computing (1)".
* "in short runtime" -> "fast"
* Section 2.4, Simulated data: I don't think you mean that Venter's genome was "approved" for benchmarking purposes. Maybe you were thinking of "applied"?
* The allele frequencies 0.042 to 0.667 looked arbitrarily chosen, but I eventually figured out the reason for them. Please be more explicit of their source to help the reader, perhaps by referring to figure 2 and in its caption pointing out the allele frequencies.
* I don't think you point out where the mutations are created. Are they just inserted into the sub clones I - IV, or also in the intermediate sub clones and undergoing simulated evolution?
* Subsection "Real data": "has been approved earlier" -> "has been used previously"
* Figure 4: I find the scaling of the x-axis strange and hard to read. Instead of writing "Pr(somatic) (PHRED)" in the subfigures, write "-10 log Pr(somatic)".
* I don't think the phrase "as we pointed out so far" is proper english.
* The sentence "In other words, an optimal scenario is to employ a statistically sound routine that establishes false discovery rate (FDR) control" is better expressed as "In other words, we would like to control the false discovery rate (FDR)."
* Similarly, the phrase "a desired level of FDR control" should be "desired FDR".
* "An explanation for this to happen" -> "An explanation for this"?
* Last line of page 12: "raise only very little" -> "raise only very few".
* In Discussion: "it is able to raise call sheets of superior quality" -> "it is able to call structural somatic variants better than alternative method".
* In Declarations, Availability: "and and" -> "and"
* In methods, around equation (7): You write "For $\theta_c$, …, we obtain the relationship $$\theta_t =…$$ which, just as $\theta_t$ can take values in all of the unit interval [0,1] for a somatic variant." This sentence is strange. The word "which" must (?) refer to equation (7), but the RHS there is of course simply $\theta_t$.
* I think section 5.2 can be deleted because you made this point already.
* In section 5.3 you refer to "Figure 8(b)", but figure 8 does not have labeled panels (only "left" and "right"). Also, in my humble opinion the left panel does not contribute much. I was just confused and more happy about the parameter descriptions in the text.
* Page 19: "In a final remark, note that for that sake" -> "… note that for the sake"
* On page 20 you refer to "re-alignment" before that concept is introduced. It was quite confusing.
* On page 22 you state that the fragment length distribution is often approximately Gaussian. I think that is only in a loose sense of the word "approximately". Given the statistic rigour you want in this paper, I think you should rather state that you _model_ the fragment length with a Gaussian. That way, you will not need to discuss the statement with folks who have actually looked at fragment length distributions.

References
* Allhoff et al: Poor Alexander's last name is mistreated. And the journal's name should be properly capitalised.
* Grüning et al: First author's first name and second author's first name are not correct.
* Shockingly: The title of the Snakemake paper is not correct.

**Authors Response**

**Point-by-point responses to the reviewers’ comments:**

We thank both reviewers for the comprehensive and positive reviews. In the following, we answer each comment in detail.

Reviewer 1

General comments The Koster et al. present a novel model Varlociraptor for im- proving our ability for predicting the indels in cancer tumors. It models the intrinsic issues of short read mapping to calculate the FDR for somatic indels predicted. This is a very important problem and in practice can have a great impact in studying indels in tumor. I enjoyed the authors approaches in solving the problem and I think this paper would be of high interest and impact.

*Response We are pleased to hear that and thank the reviewer for this positive assessment!*

Comment 1 Methods: Going over the method it seems to me that the probabilities calculated in equations (2), (3), (4) can be extended to consider the potential of multiple mapping of the reads (i.e., when one read is supporting more than one of the indels). Currently, it seems the authors only consider the aligner uncertainty as π_i as calculated_ by MAPQ score. Can the authors please discuss this issue in the paper more clearly?

*Response Indeed, the implementation of our model (Varlociraptor) currently only considers the primary alignment. This is however not a limitation of the model, and was done for simplicity and performance reasons. In fact it is at the core of the method to be able to consider multiply mapped reads in a statistically appropriate way. For this, it is required that the π_i, as derived from the MAPQ scores that express the alignment_ uncertainty of a read at a particular location sum up to at most 1 for one read across the different locations where it maps* *to. Future versions of Varlociraptor will exploit this as well. Thanks for pointing out that this point was not entirely clear. We have extended the text accordingly.*

Comment 2 Methods: Can the proposed approach also work for STR extensions? As it was shown a very large fraction of the indels are in reality STR extensions. However, STR extension have their own issues for prediction such as PCR stutter errors (Willems et al.). Can the method be extended to consider these issues too?

*Response Thanks for this very interesting comment. In general, Varlociraptor deals with STR extensions in the exact same way as it deals with any other indel: if suggested by a caller that specializes in STRs (or any other tool that is able to call STRs), Varloci- raptor re-evaluates the putative STR extension in the suggested (statistically rigorous) way, thoroughly examining all reads that provide support for it and also all reads that provide evidence against it. Finally one can apply false discovery control also to puta- tive STR extensions, which, just as for other classes of indels, limits the amount of false positives among such calls, without suffering from drastic losses in sensitivity.*

*PCR stutter errors add extra complications that cannot be considered at this point in time. This is not Varlociraptor’s fault, because Varlociraptor could easily consider them, if error rates for PCR stutter could be provided, as part of the sequencing library prepa- ration, which however involves to be able to quantify PCR amplification in that respect. Because this is not standard, statistical uncertainties emerging from PCR stutter cannot be addressed, neither by Varlociraptor nor by anyone else who aims at addressing this.*

*Therefore, we prefer to handle PCR stutter errors by making use of UMI’s, if such are available. UMI’s have proven to become more routine in the recent past (certainly boosted by single cell transcriptomics) also for dealing with PCR stutter, abecause they offer a very clean way (”smart deduplication”) to get rid of such errors; see for example Sena et al. [1]. We are in the process of providing an algorithm to compute consensus reads for cases where the molecular copies referring to one UMI deliver ambiguous signals, indicating the occurrence of PCR stutter errors, in other software of ours (see Rust-Bio- Tools, https://github.com/rust-bio/rust-bio-tools).*

*Again, note that currently there is no way to handle PCR stutter in a sufficiently sound way without making use of UMI’s, because the necessary parameters, unlike parame- ters referring to alignment, typing or strand bias uncertainty, are not delivered by the sequencing protocols.*

*We have added a remark in the Discussion pointing out that PCR stutter errors can be appropriately addressed, as long as UMI’s are available, while they cannot be addressed in a statistically sound way as long as they are not available.*

Comment 3 Simulations: The results provided in the simulation are quite interesting. However, the authors can try another set of simulations which they create an artificial somatic and tumor WGS by sampling from two genome with known indels predicted using long read (for example CHM1 and CHM13 haploid samples). The authors can consider the WGS from CHM1 is the normal samples and the WGS from tumor is CHM1 and some fraction of reads from CHM13 (the fraction of reads used will be the VAF). Then the somatic variants are indels predicted in CHM13 samples.

*Response Thanks a lot for this interesting suggestion. We have done this as proposed (with the minor change to take CHM13 as tumor and CHM1 as normal), see section 2.4, paragraph “synthetic data”. For two mixture rates (5% and 20% of CHM13 and the rest of CHM1), we now show results in the supplement (Figure S8-S11) and discuss them along with precision and recall from simulated data in the results section. In short, the picture is the same as with simulated data, albeit being more noisy due to the caveats mentioned in section 2.4.*

Comment 4 Real data: The authors need to compare to some of the tools developed specifically somatic indel discovery. Please also compare the results against these three tools if possible SmuRF, NeuSomatic and novoBreak.

*Response We thank the reviewer for pointing out additional tools to compare against. We have added NeuSomatic to the comparison; Varlociraptor improves the recall while maintaining precision similar to the other callers.*

*We have tried to add novoBreak, but were not able to execute it without a crash, which seems to stem from bugs in the main novoBreak script (see error log). The first row of errors points to a line in the run_ssake.pl script that tries to load a file *.ssake.asm.contigs. Since this is not an input file to novoBreak, we assume that a bug before that line causes it to be missing. Given that novoBreak development seems to have stopped 4 years ago^1^ we have therefore decided to keep it out of the comparison.*

*SmuRF is a consensus caller that takes the output of four variant callers addressing the same variant types and length ranges (SNVs and small indels) as input. Currently, Varlociraptor does not yet provide a strategy to deduplicate calls for the same variant by multiple callers into a single unified representation (which can be* *particularly challenging for larger variants). Hence, there is currently no way for a fair comparison between the two, such that we decided to skip SmuRF for now. However, extending Varlociraptor towards taking the output of multiple callers as input and automatically merging them together under consideration of all involved uncertainties is indeed a nice feature for the future. We have added a paragraph about this to the Discussion, also pointing to SmuRF as a tool to compare against once the feature is implemented.*

**Reviewer 2**

General comments The authors introduce a new method for detecting structural variants in the case of samples taken from tumors etc, where we can expect the aber- rations are in hard-to-control allele frequencies due to tumor heterogeneity, and the reference data is from the same individual as the tumor. The method is statistically sound and great effort is made to manage sources of noice and have control the error rates. I think the method is exemplary in providing detail: it made me feel that I would be able to implement the method myself (with extensive effort, but without ambiguity). I am overall very happy with the manuscript. It was an interesting read and a solid contribution to the field. I have a lot of comments on the actual text, but that should be easy to fix. I believe this paper presents a significant step forward for somatic variant discovery.

*Response We thank the reviewer for this positive assessment of our work! Given our investments in terms of presentation and readability, we are particularly happy to hear that the chosen way of presenting the model is appreciated.*

Comment 1 I think the introduction is not introducing the field properly. There is a lot of work on methods for recognising structural variants, but the field is quite summarily described. I realise there is less work on somatic variants, but even so, a reader deserves a little more guidance to the present literature.

*Response Thanks for pointing out that we should stock up on literature in this re- spect. We added two paragraphs right after the initial paragraph to review the current situation with respect to (indel, structural) variant discovery in general, and somatic variant discovery in particular. We hope that pointing out the lack of comprehensive statistical models in general combining with the fact that indels of 30-250 bp are particularly hard to discover already as germline indels adds to explaining the basic motivation behind our paper.*

Comment 2 Theorem 2.2 is listed in section 2.2 without all details needed, which is mentioned below: ”a list of extra conditions for this theorem necessary to hold” is referred to in the Appendix. I don’t this is OK. A theorem should stand by itself, not have hidden conditions. I realise the conditions are technical and boring, but they need to be codified somehow and made explicit in the theorem.

*Response We agree. We decided to follow the ever more routine practice to push technical details to appendices, but we actually appreciate ourselves that it is deemed better to list the details of the theorem already in the main text (note however that we left the proof in the appendix, because it is really lengthy and technical). Thanks for pointing this out.*

Comment 3 Figure 6 seems to indicate that variant allele frequency is harder to predict for short indels. Is this true? It is surprising because it goes against some of premises of the ”twilight zone” being for indels larger than 30 bp.

*Response The 1-30bp subplot contains significantly more data points than those for larger variant sizes. In consequence, outliers become more visible (or become evident at all only then), probably generating the impression that the prediction is less accurate. However, when looking at the error bars of the box plots, they are approximately the same for allele frequencies ≥ 0.125. For lower frequencies, there are indeed larger error bars in the 1-30bp subplot. However, for such low frequencies, there are hardly any data points in the other plots, rendering their error bars less reliable and in general more narrow. This is also indicated by the jumping nature of those box plots in the subplots for the larger variant sizes. We have added a clarifying statement to the caption.*

Comment 4 I think Section 4 Declarations should have clear instructions on how to find the software, even if the link is in the abstract. In principle a section like this should support automatic parsing of download instructions.

*Response We have extended the section to include the requested information.*

Comment 5 Language and style issues in the introduction:

1. First sentence, ”Cancer is a genetic disorder in the first place:”, sounds strange to me. Don’t you mean ”Cancer is primarily a genetic disorder:”? It is very unfortunate if the very first sentence causes friction for even a fraction of readers.

2. ”The fractio,n of somatic variants discovered. . . has left room for improvement. . . ” should probably be something like ”Given the fraction of somatic variants discov- ered, there is room for improvement. . . ”

3. ”Thereby, somatic insertions...” The word ”thereby” does not belong here.

4. ”in other contexts”, with references to Mandoiu and Zelikovsky, and others: what

other contexts?

5. ”In this paper, we suggest such a sensitive strategy, and prove that with it we can make substantial progress in terms of eliminating the somatic indel discovery blind spot.” → ”In this paper, we suggest such a sensitive strategy, and prove that it substantially reduces the somatic indel discovery blind spot.”

6. The sentence ”In the second step—which is unique to somatic variant discov- ery— one runs a differential analysis that classifies putative variants into somatic, because they only appear in the cancer genome, germline and healthy somatic, because variants appear in the control genome (where healthy somatic appear at subclonal levels), or just noise.” is difficult to parse and should be rewritten.

7. Lines 43—50 on page 1: repeated use of ”dealing” and ”deal”

8. What is referred to with ”generic settings” on line 48/49?

9. Page 2, first paragraph: ”The purpose of this paper is to do this: we would like to fathom the corresponding limits theoretically and to push them considerably in practice.” I suggest: ”The purpose of this paper is to understand theoretical limits of somatic variant discovery and improve practical limits substantially.” At any rate, the word ”fathom” is awkard here.

10. I think the paragraph starting with ”Key to success” can be reduced close to 50%

without loss of meaning and with improved readability.

11. ”Nowadays, n ≤ 60 is not uncommon.” I think ”Nowadays, n around 60 is not

uncommon.” is better describing what you mean.

*Response We thank the reviewer for the thorough reading of the introduction. Please find responses to specific items below. All other items have been fixed as suggested.*

1*. Thanks for this—in fact on a side remark very intriguing—comment! The comment caused us to re-think how to optimally formulate what we want to say in the first sentence. Given the latest developments, and the new insight into the role of epigenetics and mitochondrial health in cancer onset and progression (with GP’s recommending to avoid carbohydrate intake during therapies in the clinic in the meantime), it seems no longer be really clear that cancer is ’primarily’ a genetic disorder, which was indeed what we wanted to express when we started to launch the study. Now, to the best of our understanding, it seems that the current working hypotheses rather are that somatic genetic variants are a precondition, while not necessarily the real reason for developing cancer. So, by serendipity, ’in the first place’ may indeed be the better match in the meantime. While then, still, ’in the first place’ – we agree – sounds awkward, and is hard to follow, a bad choice; thanks for pointing this out! Based on the latest advances in cancer research, we opted for”The foundation of cancer are modifications of the genome.”, which should be true regardless of the choice of actually available working hypotheses on why there is cancer.*

4*. We meant ”in contexts where no somatic variants were considered”, and have*

*reformulated the corresponding part.*

*9. Thanks for pointing out. We opted for ”... is to understand the limits of somatic*

*variant discovery in theory, and considerably push them in practice.”*

Comment 6 Later sections:

1. ”We will use the indels that are output by the competitor” — which competitor

is that, please be specific.

2. You reference Sarek in footnote 9, but Sarek seems to have a DOI one can cite as

well.

3. ”to computing (1)...” should probably be ”to compute (1)” or ”for computing

(1)”.

4. ”in short runtime” → ”fast”

5. Section 2.4, Simulated data: I don’t think you mean that Venter’s genome was

”approved” for benchmarking purposes. Maybe you were thinking of ”applied”?

6. The allele frequencies 0.042 to 0.667 looked arbitrarily chosen, but I eventually figured out the reason for them. Please be more explicit of their source to help the reader, perhaps by referring to figure 2 and in its caption pointing out the allele frequencies.

7. I don’t think you point out where the mutations are created. Are they just inserted into the sub clones I - IV, or also in the intermediate sub clones and undergoing simulated evolution?

8. Subsection ”Real data”: ”has been approved earlier” → ”has been used previously”

9. Figure 4: I find the scaling of the x-axis strange and hard to read. Instead of

writing ”Pr(somatic) (PHRED)” in the subfigures, write ”-10 log Pr(somatic)”.

10. I don’t think the phrase ”as we pointed out so far” is proper english.

11. The sentence ”In other words, an optimal scenario is to employ a statistically sound routine that establishes false discovery rate (FDR) control” is better expressed as ”In other words, we would like to control the false discovery rate (FDR).”

12. Similarly, the phrase ”a desired level of FDR control” should be ”desired FDR”.

13. ”An explanation for this to happen” → ”An explanation for this”?

14. Last line of page 12: ”raise only very little” → ”raise only very few”.

15. In Discussion: ”it is able to raise call sheets of superior quality” → ”it is able to

call structural somatic variants better than alternative method”.

16. In Declarations, Availability: ”and and” → ”and”

17. In methods, around equation (7): You write ”For θ_c, . . . ,_ we obtain the relationship

θ_t = ..._

which, just as θ_t_ can take values in all of the unit interval [0,1] for a somatic variant.” This sentence is strange. The word ”which” must (?) refer to equation (7), but the RHS there is of course simply θ_t._

18. I think section 5.2 can be deleted because you made this point already.

19. In section 5.3 you refer to ”Figure 8(b)”, but figure 8 does not have labeled pan- els (only ”left” and ”right”). Also, in my humble opinion the left panel does not contribute much. I was just confused and more happy about the parameter descriptions in the text.

20. Page 19: ”In a final remark, note that for that sake” → ”... note that for the

sake”

21. On page 20 you refer to ”re-alignment” before that concept is introduced. It was

quite confusing.

22. On page 22 you state that the fragment length distribution is often approximately Gaussian. I think that is only in a loose sense of the word ”approximately”. Given the statistic rigour you want in this paper, I think you should rather state that you model the fragment length with a Gaussian. That way, you will not need to discuss the statement with folks who have actually looked at fragment length distributions.

*Response Thanks a lot for the detailed assessment! All mentioned issues have been fixed as suggested. Where necessary we have provided a specific response below:*

*1. with competitor we meant the respective tool to compare against. This has now*

*been clarified in the text.*

*7. Thank you for spotting this. We have added this information to the text. In short: they are inserted into the subclones, but the topology defines which variants are shared between subclones.*

*17. Indeed, the sentence was a bit broken. We have separated the statements into*

*multiple sentences that hopefully make it more readable.*

*18. We agree that this point was already made before (in a less detailed form). We*

*have now moved the section into the appendix.*

*19. Actually, in our version of the manuscript there are labels a and b for the left and the right panel of Figure 8. Maybe there is something broken in the rendered version you received. We will check this carefully upon resubmission, thank you!*

*21. Indeed! We have reordered the paragraphs.*

7 References

1. Allhoff et al: Poor Alexander’s last name is mistreated. And the journal’s name

should be properly capitalised.

2. Grüning et al: First author’s first name and second author’s first name are not

correct.

3. Shockingly: The title of the Snakemake paper is not correct.

*Response Thanks a lot :-). All fixed.*

References

[1] J. A. Sena, G. Galotto, N. P. Devitt, M. C. Connick, J. L. Jacobi, P. E. Umale, L. Vidali, and C. J. Bell. “Unique Molecular Identifiers reveal a novel sequencing artefact with implications for RNA-Seq based gene expression analysis”. en. In: Scientific Reports 8.1 (2018), pp. 1–13._9_

**Second round of review**

**Reviewer 1**

The reviewers have adequately answered all my concerns.
